# An Asymmetric Approach toward the *Aristotelia* Alkaloid (−)-Penduncularine

**DOI:** 10.1021/acs.orglett.5c02062

**Published:** 2025-07-14

**Authors:** Guoduan Liang, Kirsten E. Christensen, Edward A. Anderson

**Affiliations:** Chemistry Research Laboratory, Department of Chemistry, 6396 University of Oxford, 12 Mansfield Road, Oxford OX1 3TA, U.K.

## Abstract

We report a catalytic enantioselective total synthesis of an endocyclic alkene regioisomer of (−)-peduncularine, termed (−)-pseudo-peduncularine, and also a synthesis of its C7 epimer. Highlights of the syntheses include a new strategy for the construction of the peduncularine framework by palladium-catalyzed ynamide cycloisomerization/enamide reduction, which could be performed on a multigram scale. By introducing the indole side chain as a substituent on the ynamide, this approach contrasts with previous strategies that have relied on late-stage Fischer indole synthesis. Inversion of the C7 stereocenter installed in the cycloisomerization process could be readily achieved by temporary azabicycle ring opening, using an enamide hydrolysis/ketone reduction/Mitsunobu cyclization sequence. A catalytic asymmetric sulfonamidation of a racemic allylic benzoate enabled an enantioselective synthesis of (−)-pseudo-peduncularine.

## Graphical abstract

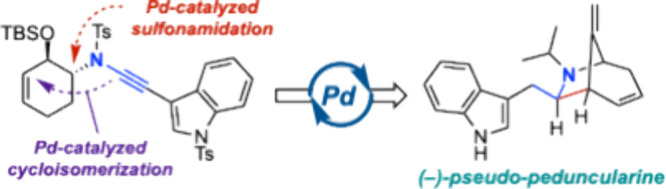

The *Aristotelia* alkaloid (−)-penduncularine (**1** (Scheme 1a)) was first isolated from Tasmanian shrub *Aristotelia peduncularis* by Bick and co-workers in 1971,^1,2^ along with a number of structurally related alkaloids, including aristoteline and sorelline. Despite its relatively low molecular weight, peduncularine features an array of interesting and synthetically challenging structural features, including an unusual 6-azabicyclo[3.2.1]­octene motif, a C7-*exo* (indol-3-yl)­methylene side chain, a C3=C4 bond, a C8 exocyclic methylene group (on the one-carbon bridge), and three stereocenters.

**Scheme 1:**
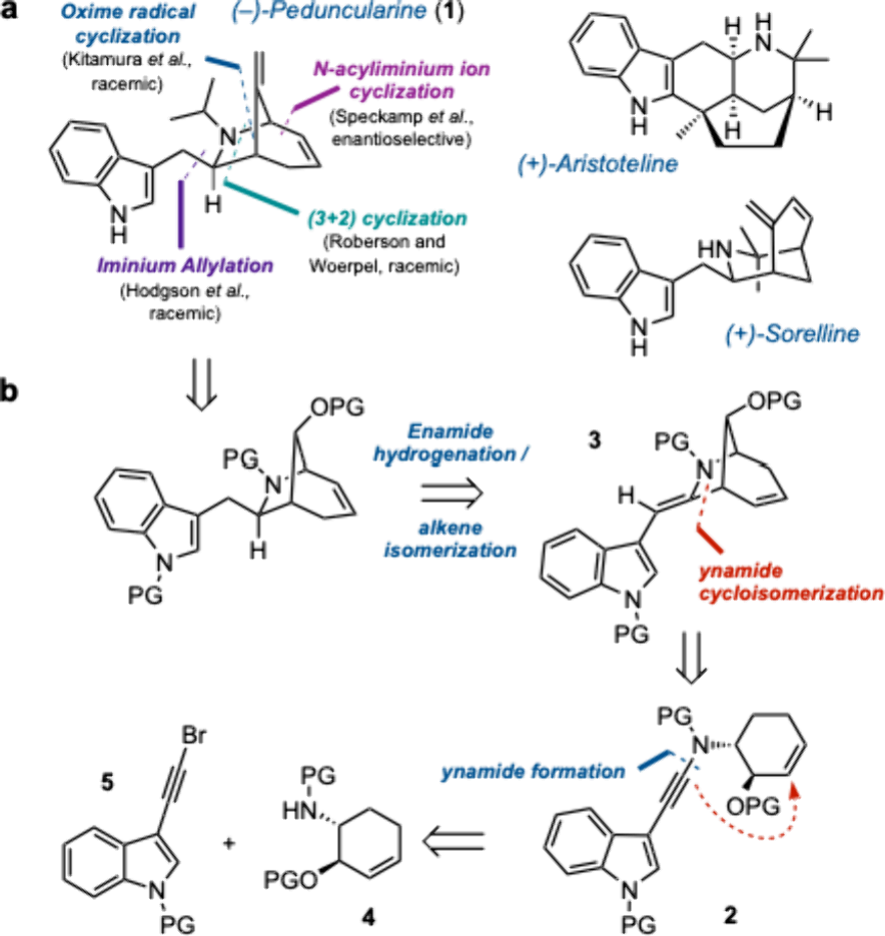
(a) Representative Family Members of the *Aristotelia* Alkaloids and Previous Strategies toward Peduncularine and (b) Our Planned Enantioselective Approach, Centered on Pd-Catalyzed Enynamide Cycloisomerization to Construct the Azabicyclic Core (PG = protecting group)

Due to these structural features, and its anticancer properties,^3^ peduncularine has drawn much attention from synthetic chemists. To date, four total syntheses (but only one asymmetric approach) have been reported, each employing different strategies to construct the unique 6-azabicyclo[3.2.1]­oct-3-ene core. Speckamp and co-workers implemented a silicon-assisted *N*-acyliminium ion cyclization strategy and completed the first and only enantioselective total synthesis to date in 18 steps from (*S*)-malic acid (0.7% overall yield).^4^ While confirming the absolute configuration, control over the C7 stereocenter proved to be challenging. Roberson and Woerpel used a (3+2) annulation between a silyl-substituted cyclohexadiene and an isocyanate to generate the 6-azabicyclo[3.2.1]­oct-3-ene skeleton, subsequently completing the total synthesis of (±)-peduncularine with a longest linear sequence (LLS) of 13 steps in an overall yield of 3.1%.^5^ Kitamura and co-workers employed a radical cyclization of an oxime, followed by reductive ring opening to construct the core framework; the natural product was obtained in a LLS of 13 steps with a 5.5% overall yield, albeit again facing challenges over control of stereochemistry at C7.^6^ Finally, Hodgson and co-workers described a TMSOTf-induced rearrangement cascade to give a 7-allylated 6-azabicyclo[3.2.1]­octan-3-one; this approach led to (±)-peduncularine in 1.2% overall yield via a 17 step LLS.^7^ Aside from these approaches, four formal syntheses^8–11^ have been reported (from the groups of Rigby,^8^ Woerpel,^9^ Weinreb,^10^ and Martin^11^), all targeting an intermediate in the Speckamp route.

Our group has developed a variety of metal-catalyzed cycloisomerizations of alkenyl ynamides to generate azacyclic products that are useful for total synthesis endeavors as well as medicinal chemistry applications.^12–15^ Building on the use of palladium-catalyzed enynamide cycloisomerizations to construct fused and spirocyclic azacycles in a synthesis of the core of the natural product gelsemine,^16^ we questioned whether we could apply ynamide cycloisomerization to the synthesis of a bridged bicyclic azacycle, a transform that has not been previously exploited in total synthesis contexts. Specifically, we hypothesized that from a suitable cyclohexenyl ynamide **2** (Scheme 1b), we might generate unusual 6-azabicyclo[3.2.1]­octene framework **3** of peduncularine in a single step, while simultaneously installing its (indol-3-yl)­methylene side chain. This strategy differs from all previous reports in that it does not rely on a late-stage Fischer indole synthesis to install the indole ring; rather, the indole moiety would be introduced early in the synthesis, thereby improving convergency. Cyclohexene **2** would in turn be derived from ynamide formation^17–19^ between cyclohexenyl sulfonamide **4** and (for example) bromoalkyne **5**. Here we report the realization of this plan for the assembly of the peduncularine framework and its elaboration toward peduncularine itself. These studies resulted in an enantioselective synthesis of (−)-pseudo-peduncularine (the C2–C3 alkene regioisomer of (−)-peduncularine), further demonstrating the potential of ynamide cycloisomerizations to assemble complex alkaloid frameworks.

Before embarking on a synthesis of the fully functionalized peduncularine skeleton, we first chose to study the feasibility of bridged azacycle formation via Pd-catalyzed cycloisomerization in a system lacking the indole side chain, namely cyclohexenyl ynamide **6** (Scheme 2). Toward this end, enone **7** was first prepared from cyclohexadiene by copper-catalyzed aziridination to give monoaziridine **8** ^20^ (59% yield on a 10 mmol scale), followed by oxidative ring opening with DMSO at 60 °C (92%).^21^ Stereoselective reduction of enone **7** (NaBH_4_) followed by TBS protection of the resulting alcohol provided **9** in 89% yield over two steps. Alternatively, **9** could be accessed by direct hydrolytic ring opening (aqueous NaHSO_3_)^22^ and TBS protection; both routes afforded **9** as a single diastereomer. CuI-catalyzed coupling^17–19^ of **9** with bromoalkyne **10** generated **6** in a nonoptimized 44% yield. To our delight, ynamide cycloisomerization catalyzed by Pd­(OAc)_2_/bbeda^12^ generated azabicyclo[3.2.1]­octene skeleton **11** in 77% yield, thus demonstrating the viability of cycloisomerization to assemble the core carbon framework of peduncularine. As the bridge silyl ether in **11** was designed as a precursor to the C8 *exo*-methylene motif required in peduncularine, we questioned whether this exocyclic alkene might be tolerated in the cycloisomerization step. We therefore carried out methylenation^23^ of aziridine **8** to give diene **12**; however, attempted ynamide formation using bromoalkyne **10** failed. After significant effort,^24^ we discovered that only a terminal ynamide **13** (prepared via a dichloroenamide intermediate **14** as developed by our group)^25^ was tolerated in this more ambitious cycloisomerization but delivered product bicycle **15** in only 20% yield and required a high catalyst loading. We therefore elected to retain the C8 silyl ether as a latent alkene.

**Scheme 2:**
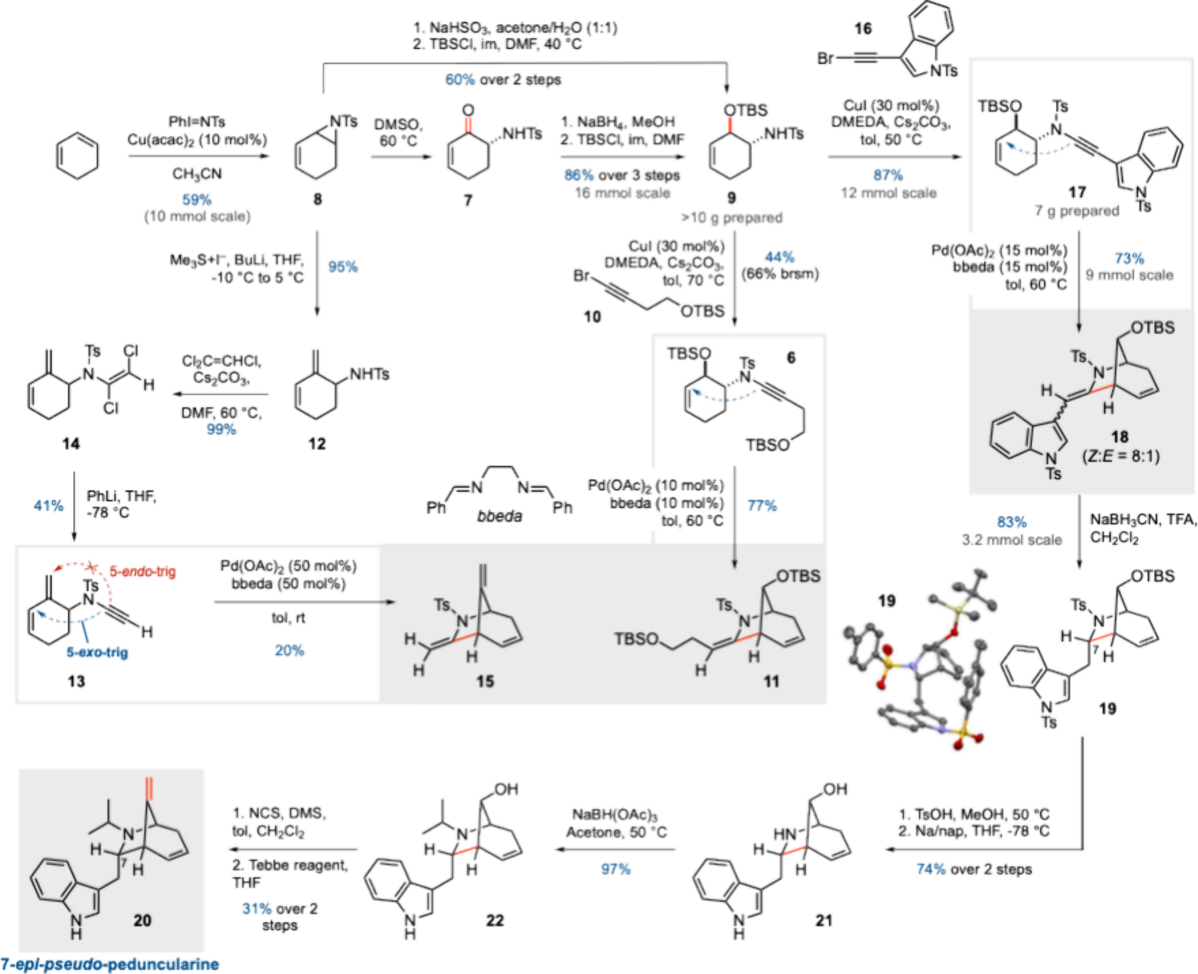
Establishing the Cycloisomerization Strategy, Determination of the Stereochemical Outcome of Cycloisomerization, and Elaboration to (±)-7-*epi*-Pseudo-peduncularine (**20**)

We next turned our attention to the formation and cyclization of an indole-substituted ynamide. Cu-catalyzed coupling^17–19^ between **9** and indole-substituted bromoalkyne **16**, which was obtained from commercially available indole-3-carboxaldehyde in three steps on a >10 g scale,^24^ generated the desired ynamide **17** in 82% yield (4.8 g scale). To our delight, Pd­(OAc)_2_/bbeda-catalyzed ynamide cycloisomerization generated indole-substituted azabicyclo[3.2.1]­octene skeleton **18** in 76% yield (73% yield on a 6.3 g scale) as an inconsequential mixture of enamide stereoisomers (*Z*:*E* = 8:1), thus providing the required carbon framework of peduncularine. Elaboration of **18** toward peduncularine first required reduction of the enamide formed in the cycloisomerization step. Treatment of **18** with AcOH and NaBH_3_CN in methanol at reflux did not give the expected reduction product, instead only effecting isomerization of the enamide *Z* isomer to the *E* isomer. Extensive screening of acids, hydride sources, and solvents eventually revealed that the combination of TFA and NaBH_3_CN in CH_2_Cl_2_ afforded reduction product **19** in 83% yield on a 2.2 g scale;^24^ a one-pot cycloisomerization/reduction sequence could also be carried out, which delivered **19** in 52% yield from **17**. However, while the X-ray crystal structure of **19** confirmed the correct skeletal connectivity required for peduncularine,^26^ it also revealed the opposite stereochemistry at C7 to that needed for the natural product. This is presumably due to enamide protonation/reduction on the less hindered *exo*-face of the bridged bicyclic scaffold.

Before the revision of the stereochemistry at C7 was addressed, a total synthesis of 7-*epi*-pseudo-peduncularine (**20**) was first carried out. Deprotection of the TBS ether and detosylation gave **21** (74% yield over two steps) on a gram scale. Reductive amination installed the isopropyl group on the nitrogen atom in 97% yield (**22**). A number of methods were screened for surprisingly challenging oxidation of the hindered C8 alcohol, from which the Corey–Kim oxidation emerged as being the most suitable.^24^ Tebbe olefination^27,28^ of **28** furnished 7-*epi*-pseudo-peduncularine **20** (31% yield over two steps).

Having completed a synthesis of 7-*epi*-pseudo-peduncularine, we turned our attention to revision of the C7 stereochemistry and one-bond migration of the endocyclic alkene (Scheme 3). After exploring a number of strategies, we found that the former goal could be achieved by initial hydrolysis of enamide **18** with aqueous TFA to give ketone **23** in 97% yield. NaBH_4_ reduction provided alcohol **24** as a single diastereomer (70%), with the stereoselectivity of the reduction possibly dictated by a conformationally constraining hydrogen bond between the carbonyl oxygen and sulfonamide NH. Treatment of **24** under Mitsunobu conditions afforded **25** with the correct (inverted) C7 stereochemistry. The stereochemistry of **25** was unequivocally determined by X-ray crystallographic analysis of alcohol **26**,^26^ formed from TBS deprotection of **25**.

**Scheme 3:**
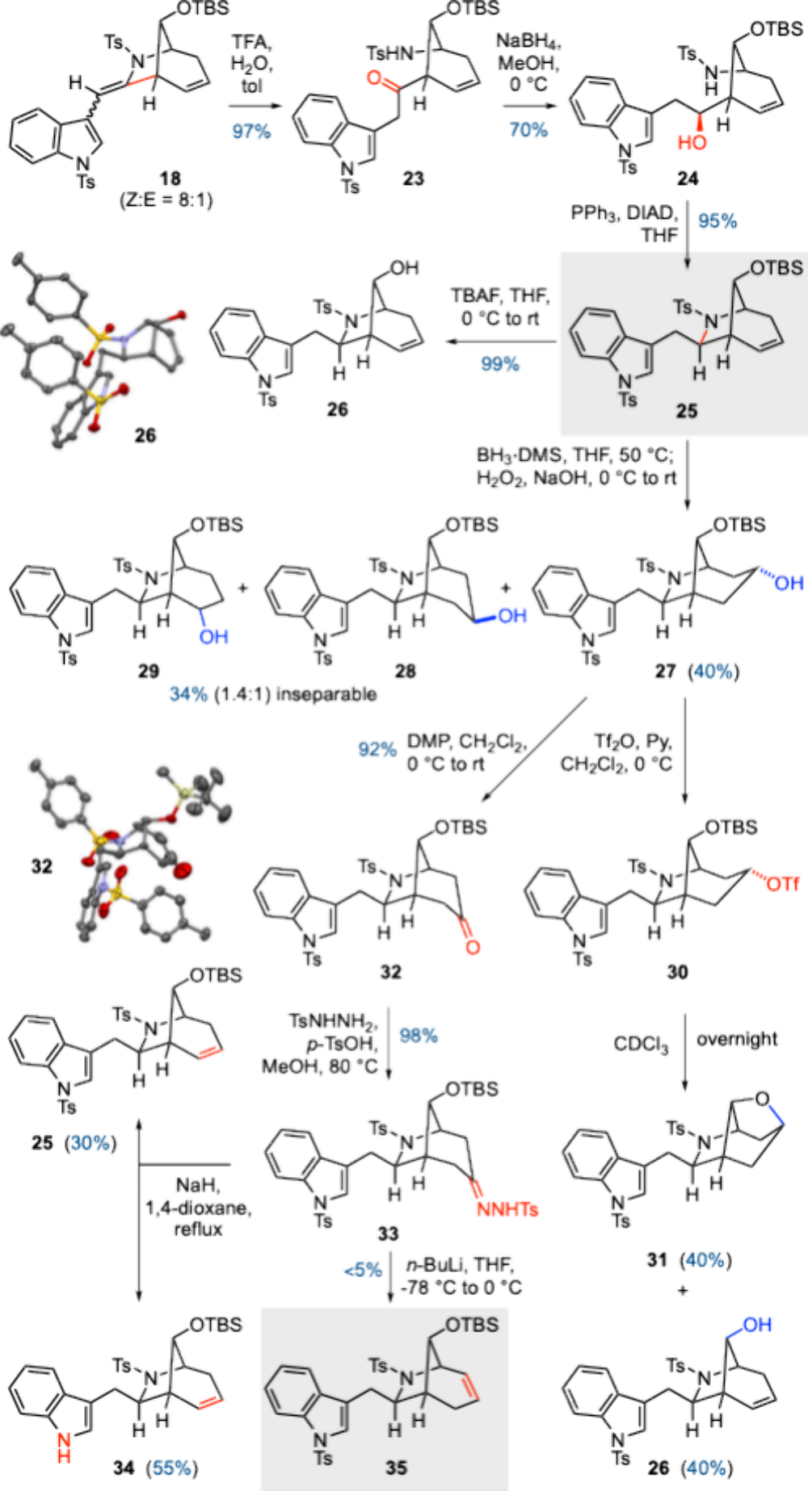
Inversion of C7 Stereochemistry and Selected Attempts to Achieve Endocyclic Alkene Migration

A number of tactics were pursued in attempts to achieve isomerization of the endocyclic double bond.^24^ Hydroboration/oxidation gave **27** as the predominant regio- and diastereoisomer (40%), along with its epimer **28** and a small amount of regioisomer **29** (1.4:1, 34% combined; the stereochemistry of **29** was not assigned, and **28** and **29** were inseparable). Direct dehydration of **27** or **28** was not successful, and mesylation/elimination also proved to be unfruitful. Conversion of **27** to triflate **30** led to an interesting outcome. Upon standing in CDCl_3_, **30** was transformed into tetrahydrofuran **31** (40% yield from **27**) and the undesired alkene regioisomer **26** (40% yield from **27**). These products may arise from S_N_1 and E1 pathways, where an intermediate carbocation generated from the loss of CF_3_SO_3_^–^ either is captured by the proximal silyl ether oxygen atom to generate **31** or undergoes loss of a proton to give **32**. Attempted elimination of THF **31** under basic conditions was unsuccessful.

We considered that it may also be possible to instead effect alkene installation from the ketone oxidation state. Dess-Martin oxidation of **27** gave ketone **32** in excellent yield, whose structure was confirmed by X-ray crystallographic analysis.^26^ Enol triflate formation (with a view to Pd-catalyzed reduction) was not successful, although tosylhydrazone **33** could be formed in 98% yield, in readiness for Bamford–Stevens elimination.^15^ To our surprise, treatment of **33** with NaH in 1,4-dioxane at reflux gave solely the undesired alkene product **25** in 30% yield and elimination/detosylation product **34** in 55% yield; none of the desired alkene isomer was observed. Only a Shapiro reaction (using *n*-BuLi) gave trace amounts of a product tentatively assigned as the desired regioisomer **35** (<5%), with most of the substrate decomposing under these conditions. Comparison of the ^1^H NMR spectra of **25** and **35** revealed distinct differences, with the alkene protons in particular becoming significantly more deshielded in **35** (5.60 and 5.49 ppm) compared to **25** (5.30 and 5.22 pm) due to inductive electron withdrawal by the adjacent sulfonamide.^24^

In light of these challenges, we chose to instead address an asymmetric synthesis of the double bond isomer of peduncularine itself, dubbed “pseudo-peduncularine”. This required assembly of aziridine **8** in the enantioenriched form (Scheme 4). To achieve this, we employed the elegant tactic of Taguchi and co-workers for the synthesis of iodoaziridine **36**.^29^ Specifically, cyclohexanol benzoyl ester **37** was subjected to a Trost asymmetric sulfonamidation reaction, which delivered cyclohexenyl sulfonamide (+)-**38** in 95% ee (70% yield). Iodoaziridination^30^ of **38** afforded product (−)-**36**, which upon treatment with KO*t*-Bu underwent elimination to give enantioenriched allylic aziridine (+)-**8** (53% yield, 95% ee). From here, the same pathway as executed previously (Scheme 2) was employed to reach (−)-**25**, namely, aziridine ring opening (**9**), ynamide formation (**17**), and cycloisomerization (**18**); enamide reduction; and inversion of C7 stereochemistry (**25**). We found that end-game conditions equivalent to those applied to **19** for the synthesis of 7-*epi*-pseudo-peduncularine translated well to epimer (−)-**25**. Desilylation and detosylation afforded alcohol (+)-**39**, which underwent smooth reductive amination with acetone to afford (+)-**40** in 96% yield. Finally, application of the Corey–Kim oxidation/Tebbe olefination sequence gave (−)-pseudo-peduncularine (−)-**41** ([α]_D_^25^ = −4.6 (*c* 0.13 in MeOH)).

**Scheme 4:**
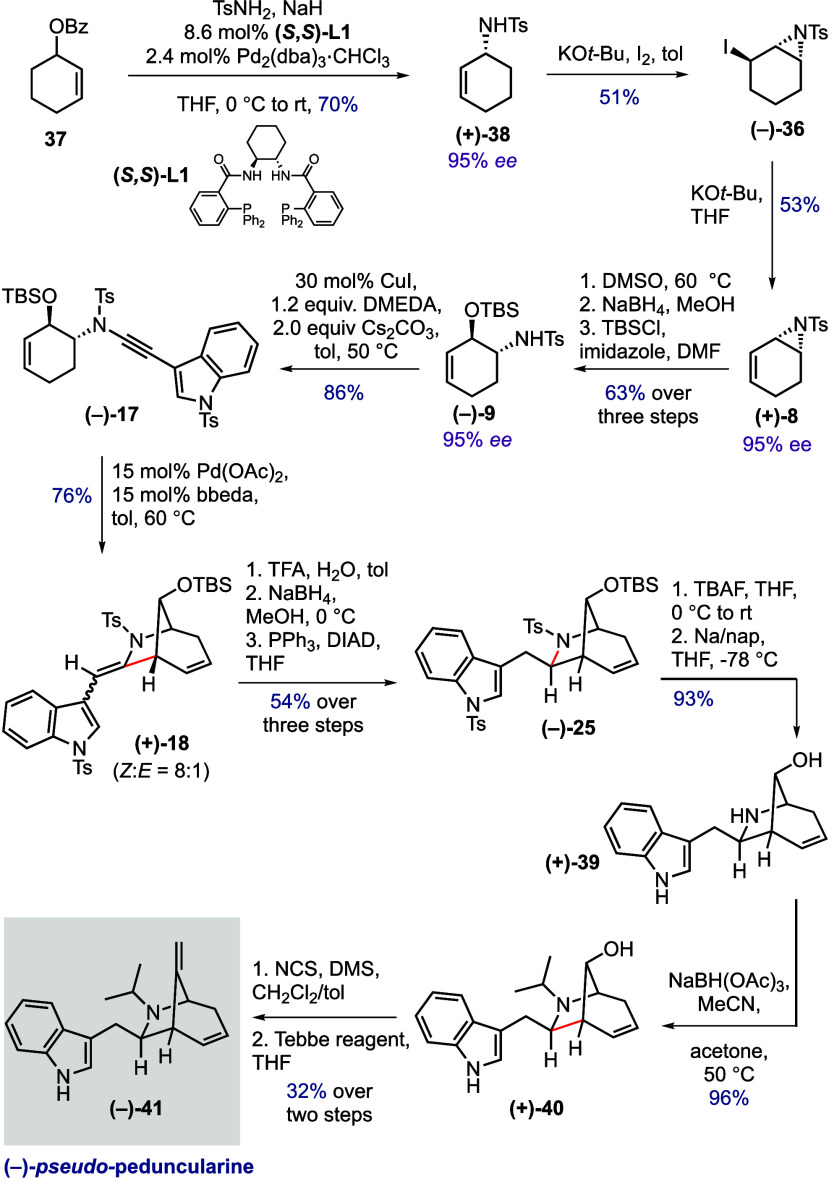
Asymmetric Synthesis of (−)-Pseudo-peduncularine

In summary, we have completed a synthesis of (±)-7-*epi*-pseudo-peduncularine in 10 steps from cyclohexa-1,3-diene and an enantioselective total synthesis of (−)-pseudo-peduncularine in 17 steps from cyclohexenol. Key transformations included a palladium-catalyzed asymmetric allylic sulfonamidation to control enantioselectivity and a palladium-catalyzed cycloisomerization of a cyclohexenyl ynamide to afford the unusual 6-azabicyclo[3.2.1]­oct-3-ene peduncularine core, while simultaneously installing the required indole-bearing side chain. This work further underlines the utility of ynamide cycloisomerizations in natural product synthesis, in particular, for the construction of complex 3D architectures.

## Supplementary Material



## Data Availability

The data underlying this study are available in the published article and its Supporting Information.

## Notes

Experimental procedures, details of reaction optimization, and copies of ^1^H and ^13^C NMR spectra (PDF)
