# Supplementary material for: An Asymmetric Approach toward the Aristotelia Alkaloid (−)-Penduncularine
Source: Org Lett. 2025 Jul 14;27(29):7798–803. doi: 10.1021/acs.orglett.5c02062 (PMC12305644; doi:10.1021/acs.orglett.5c02062)

Supporting Information

An asymmetric approach towards the *Aristotelia* alkaloid (−)-penduncularine

Guoduan Liang, Kirsten E. Christensen and Edward A. Anderson*

Chemistry Research Laboratory, Department of Chemistry, University of Oxford, 12 Mansfield Road, Oxford OX1 3TA, United Kingdom

Contents

1. General experimental considerations 2
2. Additional strategies explored for ynamide synthesis 4
3. Additional strategies explored to invert the stereochemistry at C7 5
4. Additional strategies explored for migration of the endocyclic double bond 7
5. Additional strategies explored for asymmetric synthesis 13
6. Reaction optimisation tables 15
7. Characterization of compounds 17
8. X-ray crystallographic data 72
9. References 78
10. Copies of NMR spectra 80

1. General experimental considerations

Regents, solvents and reaction conditions: All reactions were performed in oven-dried glassware under a nitrogen atmosphere unless otherwise stated. For all reactions which needed heating, an oil bath was used as the heating source. Solvents and commercially available reagents were dried and purified before use where appropriate using standard procedures. Tetrahydrofuran (THF), dichloromethane (DCM), dimethylformamide (DMF), dioxane, toluene and triethylamine (TEA) were obtained anhydrous from solvent dispenser units having been passed through an activated alumina column under nitrogen. Anhydrous 1,2-dichloroethane (DCE) was distilled from calcium hydride. Anhydrous methanol (MeOH) and dimethylsulfoxide (DMSO) were obtained commercially and used without further purification. Brine refers to a saturated aqueous solution of NaCl.

NMR Spectra: Proton (^{1}H) and carbon (^{13}C) NMR spectra were recorded on a Bruker AVII500 (500/125 MHz), Bruker DPX400 (400/100 MHz) or Bruker AVF400 (400/100 MHz). Proton and carbon chemical shifts (δ_H, δ_C) are quoted in ppm and referenced to tetramethylsilane. ^{1}H NMR spectra were recorded using an internal deuterium lock for the residual protons in $CDCl_3$ (δ 7.26) and C_6D_6 (δ 7.16). ^{13}C NMR Spectra were recorded using an internal deuterium lock using solvents $CDCl_3$ (δ 77.0) and C_6D_6 (δ 128.06). Assignments were made on the basis of chemical shifts, coupling constants, COSY, HSQC, HMBC, nOe, NOESY data and comparison with spectra of related compounds. Resonances are described using the following abbreviations: s (singlet), d (doublet), t (triplet), q (quartet), sept. (septet), m (multiplet), br. (broad), ap. (apparent), dd (double doublet) and so on. Coupling constants (*J*) are given in Hz and are rounded to the nearest 0.1 Hz. H and H' refer to diastereotopic protons attached to the same carbon and imply no particular stereochemistry.

Mass Spectra: Low resolution mass spectra were recorded on a Micromass LCT Premier spectrometer (ESI). High resolution mass spectra were recorded by the Mass Spectrometry service of the Chemistry Research Laboratory, University of Oxford, using a Bruker Daltronics microTOF spectrometer (ESI). *m/z* values are reported in Daltons with their percentage abundances and relevant fragment ions in parentheses. High resolution values are calculated to four decimal places from the molecular formula, all found values being within a tolerance of 5 ppm.

Enantiomeric excess: Enantiomeric excess (*ee*) was determined by HPLC on an Agilent 1200 series running in normal phase under UV (210-254 nm) detection using a ZORBAX RX-SIL (150 mm × 4.6 mm ID) as the analytical column. Chiral analysis was carried out on a DAICEL CHIRALPAK®-IB or IC (250 mm × 4.6 mm ID) or using Chiral column Lux 5 µm i-Amylose-1-00G-4762-E0 [250 x 4.6 mm ID].

Optical rotations were recorded on an Anton Paar polarimeter (instrument type: MCP 150) with a 50 mm path cell length (using the sodium D line, 589 nm). Specific rotations ($[\alpha]_D^{25}$) are given in deg dm^2 g^{-1}. Concentration (c) is reported in g/100 mL. For simplicity, the numbering of the carbon atoms of a given structure does not follow IUPAC rules.

Infrared Spectra: Infrared spectra were recorded on a Bruker Tensor 27 Fourier transform spectrometer, as a thin film on NaCl plates or a diamond ATR module. Absorption maxima (ν_{max}) are quoted in wavenumbers (cm^{-1}).

Melting Points: Melting points were recorded on a Leica Galen III Compound Microscope and are uncorrected.

Chromatography: TLC was performed on Merck Keiselgel 60 F254 0.2 mm precoated plates and visualized using basic potassium permanganate dip, acidic vanillin dip or ultraviolet light. Retention factors are reported with the solvent system in parentheses. Column chromatography was performed on Merck Keiselgel 60 SiO_2 (40-63 µm) and the solvent system used is recorded in parentheses.

2. Additional strategies explored for ynamide synthesis

Ynamide formation for substrates **7** and **12** failed under the following conditions (Scheme S1): Hsung conditions, using cat. $CuSO_4{\cdot}5H_2O$, K_3PO_4, 1,10-phenanthroline; Urabe conditions, using cat. CuI, DMEDA, Cs_2CO_3; Stang conditions, using alkynyliodonium salt **S1**.

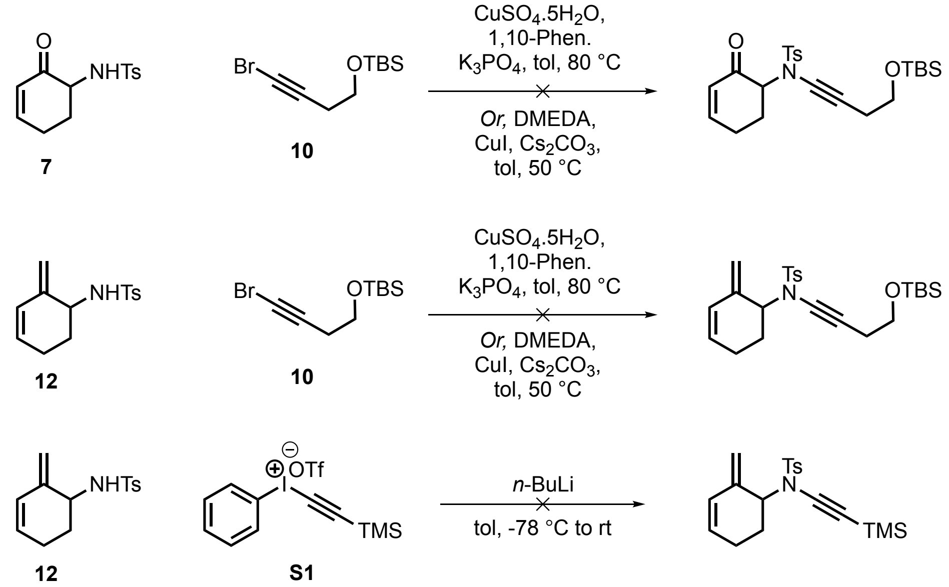

Scheme S1. Preliminary attempts for ynamide formation

Treatment of **14** (Scheme S2) with PhLi gave the intermediate alkynyllithium **S2**, however attempted alkylation with α-bromoacetal **S3** or iodide **S4** to install suitable ynamide side chains failed; **13** was the only isolable product. Only alkylation with the less-hindered MeI proved successful, giving methyl-substituted ynamide **S5** in 82% yield. However, **S5** failed to undergo productive cycloisomerisation.

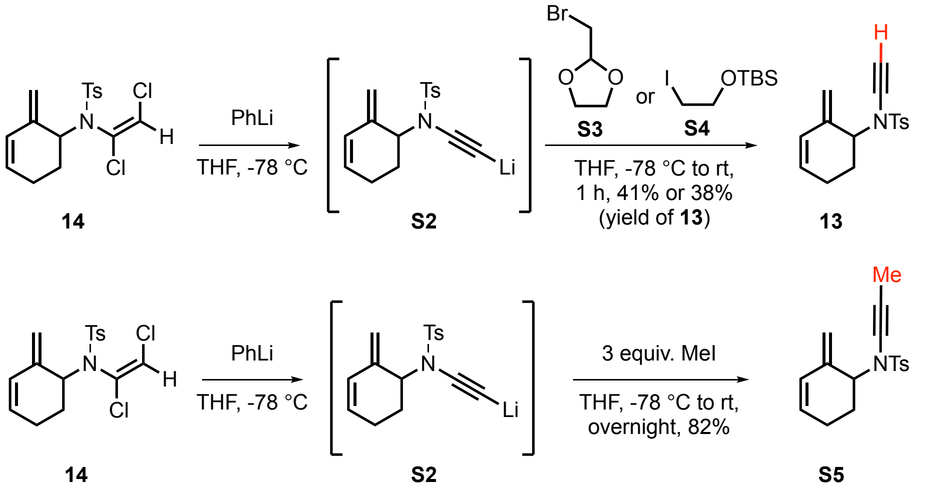

Scheme S2. Ynamide formation studies from dichloroenamide **14**.

3. Additional strategies explored to invert the stereochemistry at C7

We hypothesized that inversion of the C8 bridge alcohol / silyl ether substituent (such that it is oriented on the same side of the bicycle as the enamide) would block the undesired approach of the reducing agent to the *exo*-face in the reduction of the cycloisomerisation enamide product, hence allowing reduction from the bottom face (as drawn). Inversion was attempted as follows:

3.1 Luche reduction

We first attempted to invert the hydroxyl stereochemistry at the stage of enone **7**, planning to obtain the *syn* reduction product **S7** (Table 3.1). However, to our dismay and despite testing various reducing reagents, the best result that could be achieved was a 1:0.8 ratio of undesired *anti* (**S6**) and desired *syn* products (**S7**) as an inseparable mixture (under Luche conditions, entry 1). Other conditions attempted are illustrated in the Table.

Table 3.1 Screening of reaction condition for Luche reduction of **7**

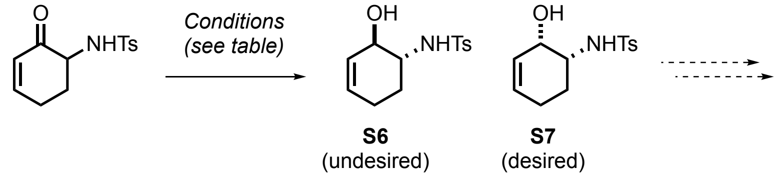

Entry	Reagent	Solvent	Temp. (°C)	Time (h)	Result (**S6**:**S7**)
1	$NaBH_4/CeCl_3{\cdot}7H_2O$	MeOH	0	1 h	1:0.8
2	$NaBH_4/CeCl_3{\cdot}7H_2O$	MeOH	-50	1 h	2:1
3	$LiAlH_4$	Et_2O	-78 to rt	12 h	1:0.4
4	DIBAL-H	THF	-78 to rt	12 h	No reaction
5	L-selectride	THF	-78 to rt	12 h	complex mixture

3.2 Mitsunobu reaction

We next subjected amino alcohol **S6** to Mitsunobu conditions in an attempt to invert the alcohol stereochemistry. However, this did not give the desired product, instead affording aziridine **8.** An attempted Mitsunobu reaction on cyclization product **S8** failed as well (Scheme 3.1)

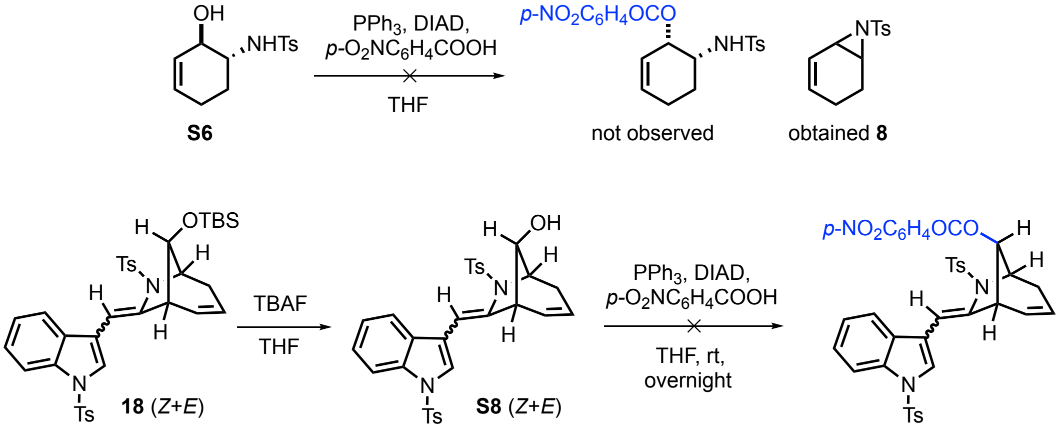

Scheme 3.1. Additional attempts at inversion of C8 stereochemistry by Mitsunobu reactions.

3.3 Retro-Mannich / Mannich reaction

We considered that inversion of C7 stereochemistry might be achieved by epimerization of **S9** through retro-Mannich ring-opening / Mannich cyclization (Scheme 3.2).

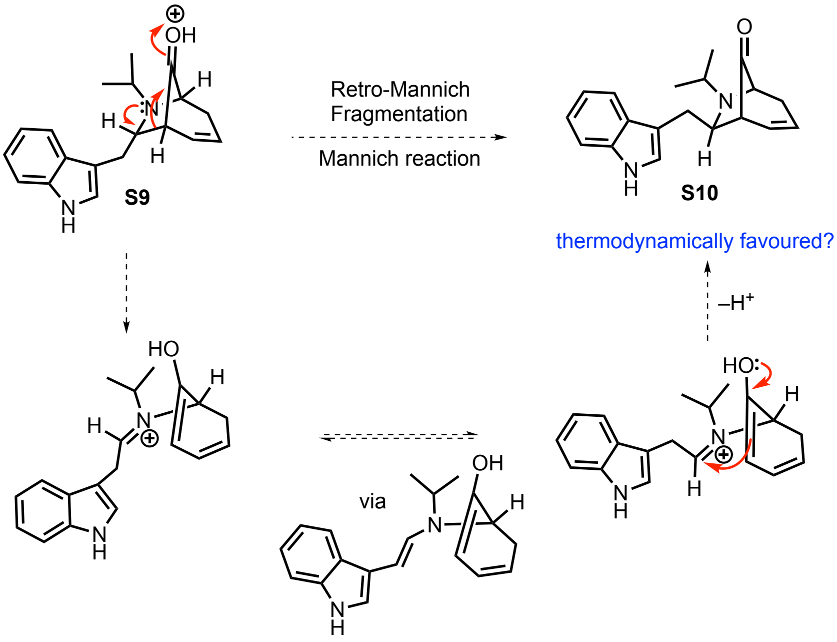

Scheme 3.2. Planned epimerization through retro-Mannich fragmentation / Mannich reaction

To our disappointment, either no reaction or decomposition was observed under various conditions (Table 3.2).

Table 3.2 Screening of reaction condition for Retro-Mannich / Mannich reaction of **S9**

Entry	Acid (5 equiv.)	Temp. (°C)	Time (h)	Results
1	PTSA	70	12 h	No reaction
2	MsOH	rt	12 h	No reaction
3	TFA	70	12 h	No reaction
4	AcOH	70	12 h	No reaction
5	CSA	70	12 h	No reaction
7	BF_3•Et_2O	70	12 h	Decomposed
8	TfOH	rt	12 h	No reaction
9	TMSOTf	rt	12 h	Decomposed

4. Additional strategies explored for migration of the endocyclic double bond

In addition to approaches outlined in the main text, the following strategies were tested for alkene isomerization.

1. Pd-catalyzed chain-walking: We tested a one-pot palladium-catalyzed cycloisomerization of enynamide **17**, with in situ palladium-mediated 'chain-walking' to give **S11**, using more forcing conditions that those required for cycloisomerization alone. Unfortunately, alkene isomerization did not occur as targeted, and we were only able to obtain cycloisomerization product **18** (Table 4.1).

Table 4.1 Attempted migration of double bond under cycloisomerization conditions.

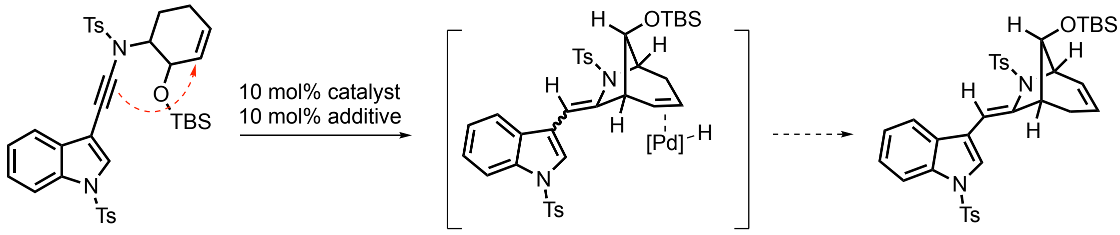

17 **18** **S11**

Entry	Catalyst	Ligand	Temp. (°C)	Time (h)	Results
1	$Pd(OAc)_2$	bbeda	85	12 h	Product **18**
2	$Pd(OAc)_2$	bbeda	110	12 h	Product **18**
3	$Pd(OAc)_2$	PPh_3	85	12 h	Product **18**
4	$Pd(OAc)_2$	PPh_3	110	12 h	Product **18**
5	$Pd(OAc)_2$	-	60	2 h	Decomposition
6	$[Pd_2dba_3]$•$CHCl_3$	-	85	12 h	No reaction
7	$[Pd_2dba_3]$•$CHCl_3$	-	110	12 h	No reaction

2. Hydroxyl-directed isomerization. We hypothesized that the C8 bridge hydroxyl group may be able to direct alkene isomerization, but despite extensive investigation, no conditions tested (such as use of rhodium catalysts that are known to promote hydroxyl-directed alkene migration),[1-2] proved successful. To our surprise, treatment of **S12** with 3 equivalents of *t*-BuOK in DMF at 60 °C afforded the O-Ts migration product **S14**.

Table 4.2 Attempted hydroxyl-direct double bond isomerization.

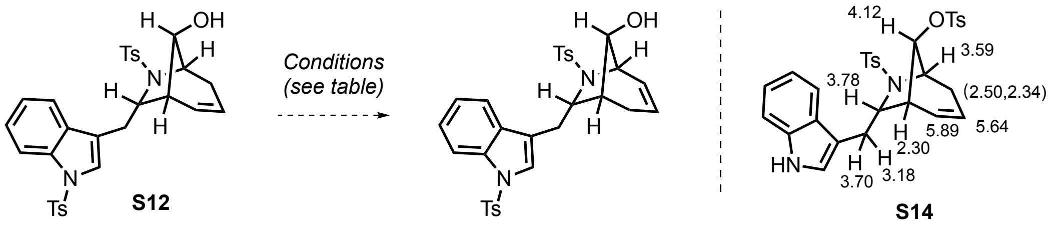

Entry	Catalyst (10 mol%)	Solvent	Temp. (°C)	Time (h)	Results
1	$RhCl_3 \cdot 3H_2O$	EtOH	105	12 h	No reaction
2	Wilkinson's catalyst	EtOH	105	12 h	No reaction
3	*t*-BuOK[a]	DMF	60	1 h	Ts migration (75%)
4	TfOH[a]	1,4-Dioxane	90	12 h	No reaction
5	Pd/C	EtOH	80	12 h	Decomposed
7	$Pd(OAc)_2$/bbeda	Tol	105	12 h	No reaction
8	$[Pd_2dba_3] \cdot CHCl_3$	DCE	85	12 h	No reaction

a) 3 equivalents

3. Reductive transposition strategy. A reductive transposition strategy was planned,[3-6] which would allow us to access known intermediate **iso-40** from a previous synthesis[7] (Scheme 4.1). This relied on allylic oxidation followed by diazene formation.

Treatment of **25** with SeO_2 and TBHP in DCM at room temperature resulted in no product formation (Scheme 4.2), while heating **25** with SeO_2 alone overnight in refluxing dioxane instead gave the benzylic oxidation product **S17** in 71% yield. Hypothesizing that the TBS ether may be too bulky such that it blocked oxidation at the desired allylic position, the TBS group was deprotected, and product **26** was tested under the same conditions, but to our dismay, neither gave the desired allylic oxidation product.

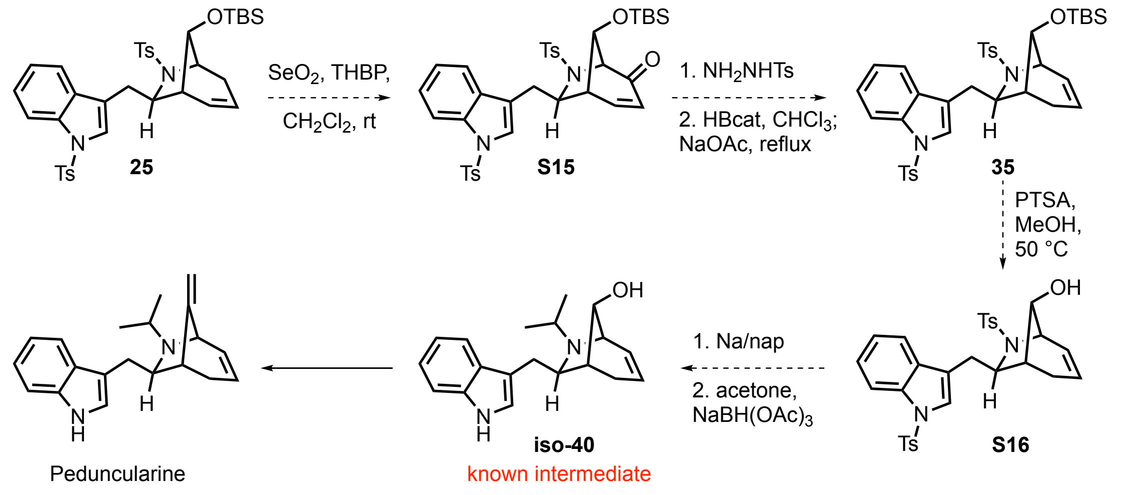

Scheme 4.1. Planned strategy for double bond isomerization using diazene rearrangement

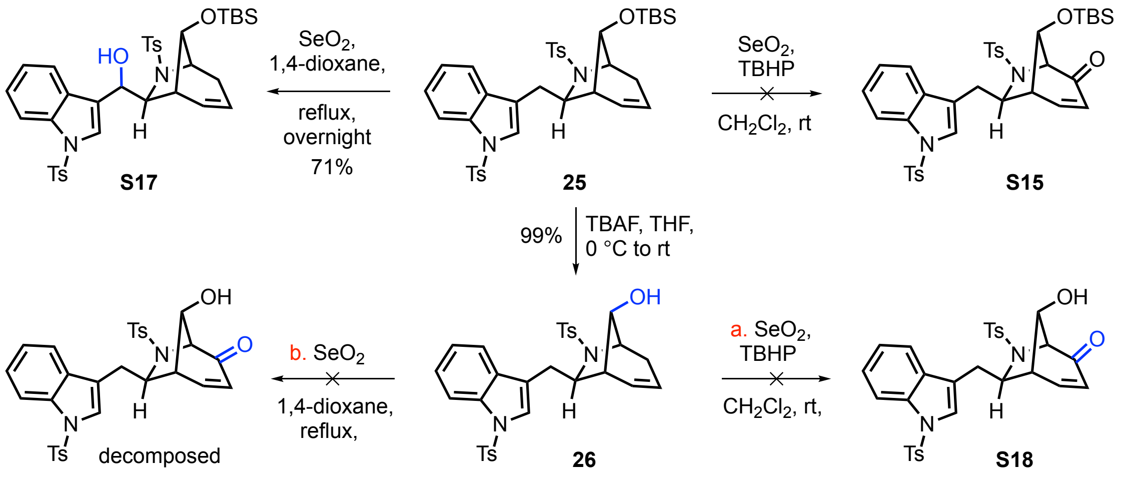

Scheme 4.2. Experimental results on attempted allylic oxidation of **25**

4. Hydroboration-oxidation / elimination. Expanding on the hydroboration / oxidation studies described in the main text, a variety of conditions were tested for dehydration of alcohol **27**. Despite a thorough screen of reaction conditions (See Table 4.3), we did not observe the desired elimination product **35** at all. Reagents/catalysts tested include TsOH, Burgess reagent, Martin sulfurane, $SOCl_2$, BF_3•OEt_2, $POCl_3$, PPh_3/DIAD.

Table 4.3 Screening of reaction condition for dehydration of **27**

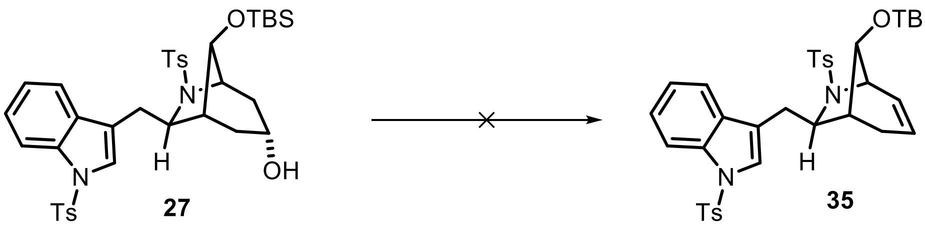

Entry	Reagent (1.2 equiv.)	Temp. (°C)	Solvent	Time (h)	Results
1	TsOH	70	Tol	12 h	Decomposed
2	Burgess reagent	70	Tol	1 h	No reaction
3	Burgess reagent	70	Tol	12 h	No reaction
4	Burgess reagent	100	Tol	12 h	Decomposed
5	Burgess reagent	80	Tol	5 h	Decomposed
6	Martin sulfurane	80	Tol	3 h	No reaction
7	$SOCl_2$	rt	CH_2Cl_2	12 h	No reaction
8	$SOCl_2$	40	CH_2Cl_2	12 h	No reaction
9	BF_3•OEt_2	0 to rt	CH_2Cl_2	12 h	Decomposed
10	$POCl_3$	60	Pyridine	2 h	No reaction
11	PPh_3/DIAD	rt	THF	12 h	No reaction
12	PPh_3/DIAD	60	THF	12 h	No reaction

Mesylation of alcohol **27** gave mesylate **S19** in a quantitative yield (Scheme 4.3). However, we were unable to effect elimination of this mesylate, with only decomposition observed under various conditions tested. Hypothesizing that the bridge OTBS group may block the approach of a base for E2 elimination, deprotection of the TBS group was carried out ,which gave **S20** in 83% yield over 2 steps. However, this also failed to generate alkene **S21** on treatment with base.

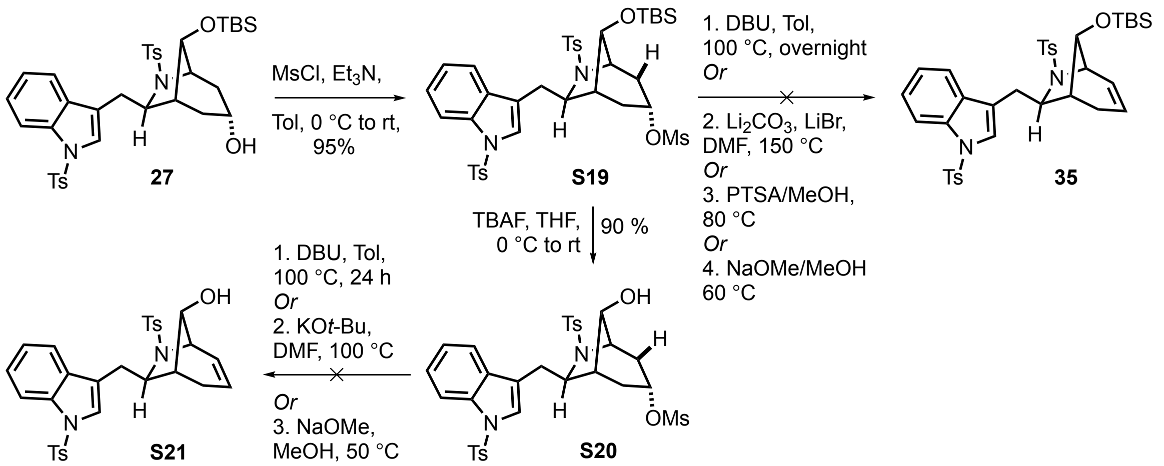

Scheme 4.3. Attempts to achieve elimination of **S19** and **S20**

5. Ring opening of THF ring 31. We questioned whether it might be possible to take advantage of this novel THF ring system **31** (6-azabicyclo[3.2.1]oct-3-ene framework), by attempting elimination under basic conditions. To our dismay, this THF proved too stable to open under a variety of conditions (Table 4.4).

Table 4.4. Attempted ring opening of THF **31**

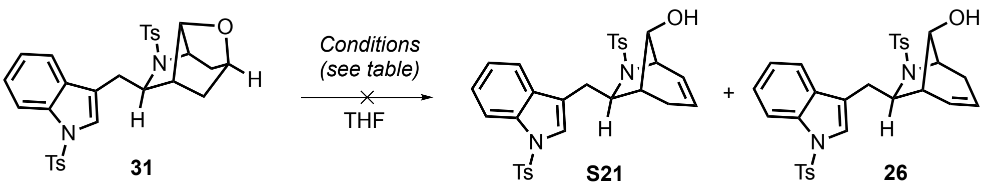

Entry	Reagent (2.2 equiv.)	Temp. (°C)	Time (h)	Results
1	*n*-BuLi	-78 to rt	3 h	No reaction
2	*sec*-BuLi	-50 to rt	3 h	No reaction
3	*sec*-BuLi/TMEDA	-50 to rt	3 h	No reaction
4	$BF_3 \cdot OEt_2$	-35	3 h	No reaction
5	Trisobutylaluminium[8]	0 to rt	12 h	No reaction
6	$ZrCl_4/Et_3N$[9]	rt	12 h	No reaction

6. Dehydration or elimination from diastereomer 28. We also tested elimination from the *β*-alcohol diastereomer **28** for dehydration (Scheme 4.4a) or elimination (Scheme 4.4b, via mesylation), but neither proved unsuccessful. Similarly, subjection of **28** to Tf_2O and pyridine in DCM overnight (Scheme 4.4c) again gave the "THF" ring product **31** and the undesired alkene **25**, supporting a likely S_N1 / E1 mechanism of this process.

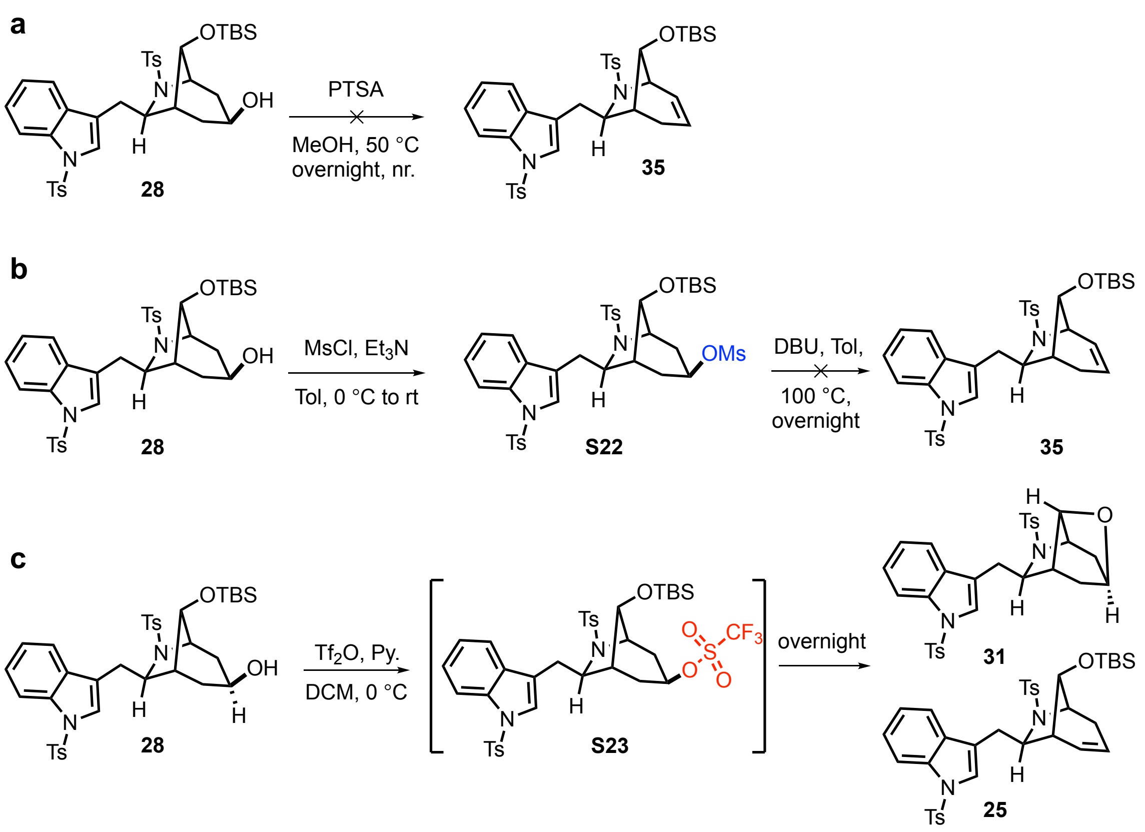

Scheme 4.4. Attempts on elimination from **28**

7. Other metal hydride-catalyzed double bond migrations. In addition to metal hydride-mediated isomerization under cycloisomerization conditions, we attempted isomerization using other metal hydrides.[10-12] Several different metal hydride complexes were tested (Rh, Ru, Pd), but to our dismay none of them were successful in achieving alkene isomerization (Table 4.5).

Table 4.5. Screening of reaction condition for metal hydride mediated isomerisation of alkene **35**

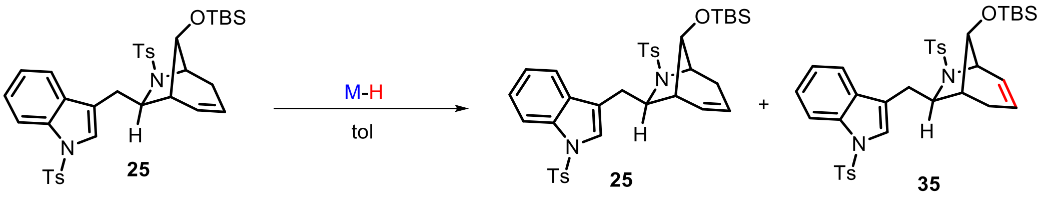

Entry	Catalyst (10 mol%)	Temp. (°C)	Time (h)	Results
1	$RhHCO(PPh)_3$	rt	3 h	No reaction
2	$RhHCO(PPh)_3$	40	12 h	No reaction
3	$RhHCO(PPh)_3$	60	12 h	No reaction
4	$In(OTf)_3/RuHCO(PPh)_3$	40	12 h	No reaction
5	Grubbs II catalyst/NaOH	110	12 h	No reaction

5. Additional strategies explored for asymmetric synthesis

In addition to the successful asymmetric synthesis strategy described in the main text, the following strategies were explored to develop an asymmetric approach towards peduncularine.

5.1 Direct Asymmetric aziridine of cyclohexa-1,3-diene

Conditions were screened for direct asymmetric aziridination of cyclohexadiene (Table 5.1). We first tested Evans' methodology – CuOTf-catalyzed asymmetric aziridination using a box ligand. However, no desired product was observed (entry 1). Switching to a Jacobsen SALEN ligand also led to no reaction (entry 2). However, using Cu(acac)$_2$ as precatalyst and box ligand **L1**, aziridine was obtained in 51% yield, albeit racemic (entry 3). Use of other box or pybox ligands (**L2**, **L3**), or decreasing the temperature to 0 °C failed, to improve the *ee* significantly; the best result obtained is 11% *ee* (Table 5.1, entry 4-6).

Table 5.1. Screening of reaction condition for direct asymmetric aziridination

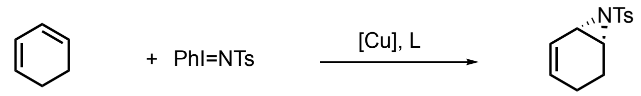

(+)-8

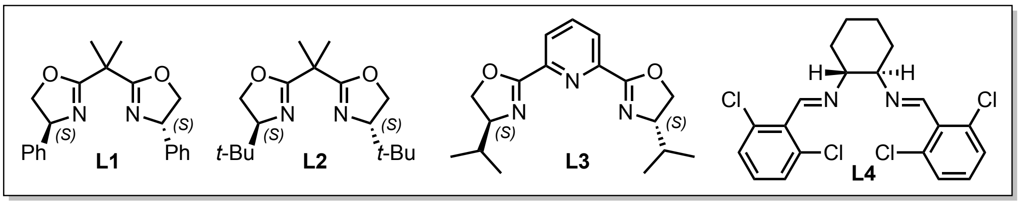

Entry	Catalyst	Ligand	Solvent	Temp. (°C)	Yield (%)	*ee* (%)
1	CuOTf	**L1**	MeCN	rt	0	-
2	CuOTf	**L4**	CH_2Cl_2	-78	0	-
3	Cu(acac)$_2$	**L1**	MeCN	rt	51	0
4	Cu(acac)$_2$	**L2**	MeCN	rt	55	5
5	Cu(acac)$_2$	**L2**	MeCN	0	16	5
6	Cu(acac)$_2$	**L3**	MeCN	rt	52	11

5.2 Saegusa oxidation of amino ketone

We questioned whether enantioenriched ketone **7** could be obtained through Saegusa oxidation[13] of cyclohexanone **S26** (Scheme 5.1a), the latter being obtained from the known sulfonamide-alcohol

S25, which could in turn be accessed from commercially available chiral pool amino-alcohol **S24**. This route was first tested in racemic form. Ring-opening of *meso* compound **S27** (Scheme 5.1b) to form (±)-**S28** proceeded in 84%, followed by DMP oxidation which provided (±)-**S29** in 60% yield. Treatment of this ketone with LiHMDS and TMSCl in THF resulted in successful TMS enol ether formation (±)-**S30**, as confirmed by ^1H NMR spectroscopy. However, the Saegusa oxidation failed, despite prolonged reaction times, increased $Pd(OAc)_2$ catalyst loading, elevation of the reaction temperature to 50 °C, *etc*.

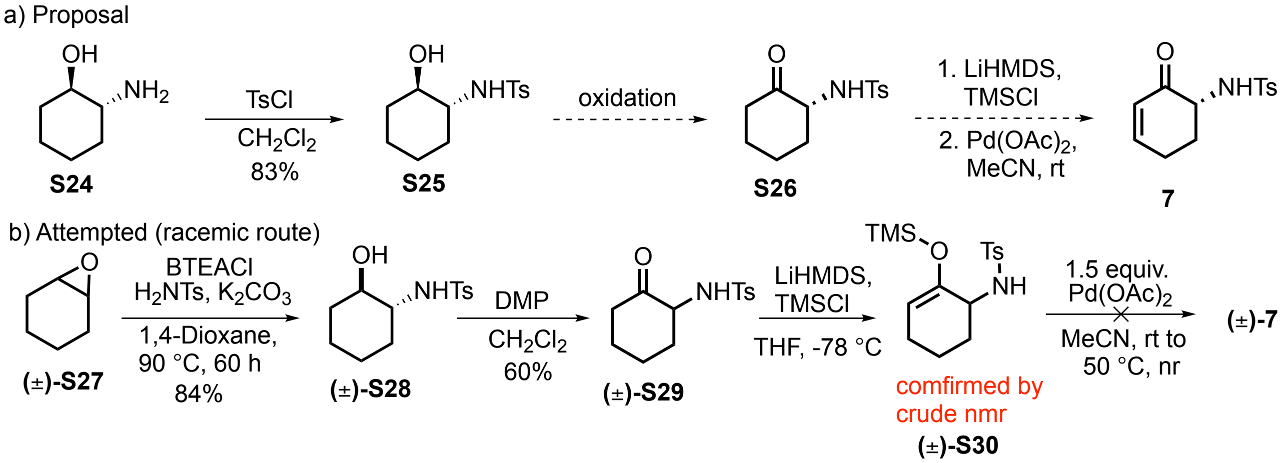

Scheme 5.1. Saegusa oxidation proposal and attempted racemic route

5.3 Attempts on Synthesis of enantiopure allylic sulfonamide by palladium-catalyzed deracemization

Finally, palladium-catalyzed deracemization of carbonate **S31** was tested, which gave the enantiopure allylic alcohol (–)-**S32**,[14] followed by Mitsunobu reaction to afford (+)-**S33** (Scheme 5.2). However, (–)-**S33** was found to be partially racemized (76% *ee*). We hypothesize that an SN2' Mitsunobu process may have occurred during this transformation, which would account for the enantiomerization.

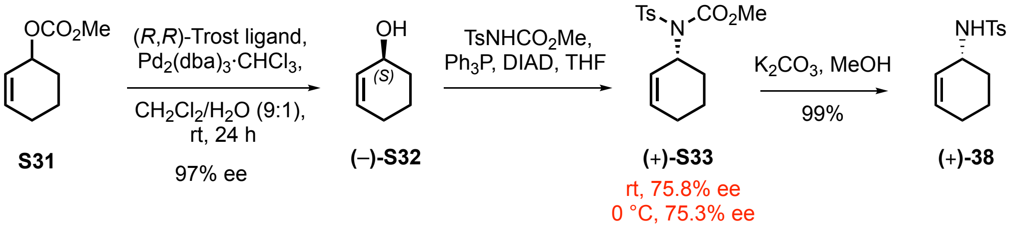

Scheme 5.2. Palladium-catalyzed deracemization strategy followed by Mitsunobu reaction

6. Reaction optimisation tables

Full details of reaction optimisation for: a) C7 reduction of compound **18**; b) oxidation of alcohol **22**; c) elimination of iodoaziridine **36**.

Table 6.1. Screening of reaction condition for enamine reduction of **18** to (undesired) C7 diastereomer **19**.

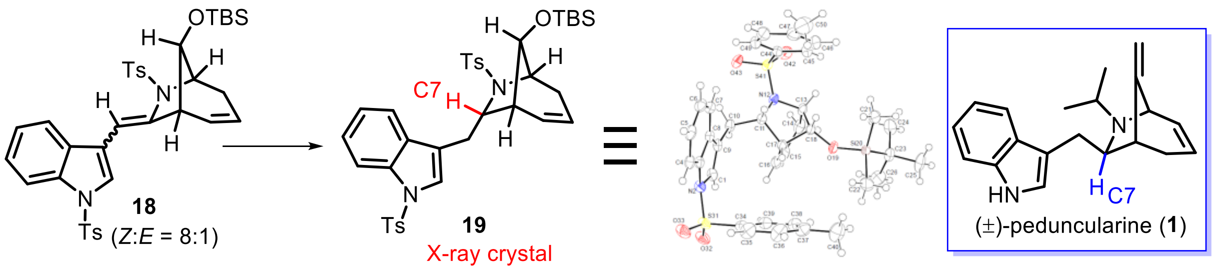

Entry	Acid	Hydride donor	Solvent	Temp. (°C)	Results
1	AcOH	$NaBH_3CN$	CH_2Cl_2	rt	No reaction
2	AcOH	$NaBH_3CN$	MeOH	90	*E*-isomer
3	PTSA	$NaBH_3CN$	Tol	60	Hydrolysis
4	PTSA	$NaBH_3CN$	MeOH	rt	*E*-isomer
5	PTSA	Hantzsch ester	Tol	60	Hydrolysis
6	AcOH	Hantzsch ester	Tol	60	*E*-isomer
7	CSA	Et_3SiH	CH_2Cl_2	rt	Hydrolysis
8	TFA	Et_3SiH	CH_2Cl_2	rt	Hydrolysis
9	TFA	$NaBH_3CN$	CH_2Cl_2	rt	**88% product**
10	TFA	$NaBH_3CN$	Tol	rt	18% product

Reactions marked "Hydrolysis" correspond to formation of the corresponding ketone by enamide hydrolysis.

Table 6.2 Screening of reaction conditions for oxidation of secondary alcohol **22**

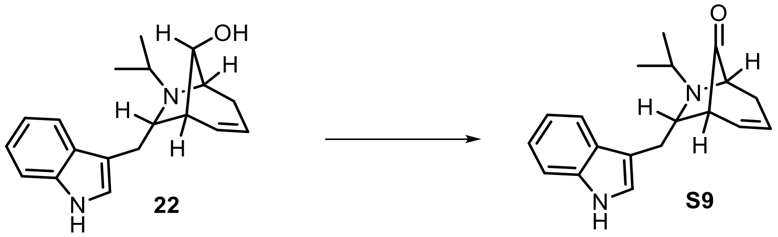

Entry	Reagent	Solvent	Temp. (°C)	Time	Results
1	Py•SO_3/Py	CH_2Cl_2	rt	2 h	No reaction
2	Py•SO_3/DIPEA	DMSO	rt	2 h	No reaction
3	Py•SO_3/DIPEA	DMSO	rt	12 h	No reaction
4	PCC	CH_2Cl_2	rt	12 h	No reaction
5	DMSO/$(COCl)_2$	THF	-78	12 h	Decomposed
6	IBX	DMSO	rt	12 h	No reaction
7	TPAP/NMO	CH_2Cl_2	rt	6 h	1:2 (sm:pdt)
8	IBX	EtOAc	85	12 h	**98%**
9	DMS/NCS	Tol/CH_2Cl_2	-78 to -50	15 min	**89%**

Table 6.3 Screening of reaction condition for elimination of **36**

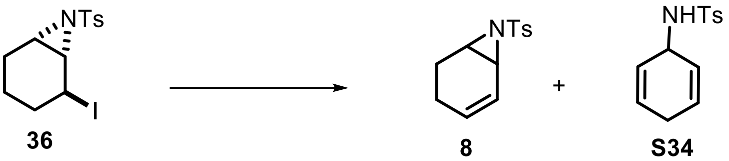

Entry	Reagent	Equiv.	Temp. (°C)	Solvent	Time (h)	Results
1	DBU	3.0	60	THF	1 h	Decomposed
2	KO*t*-Bu	3.0	60	THF	1 h	S34 formed
3	KO*t*-Bu	3.0	60	DMF	1 h	Decomposed
4	KO*t*-Bu	3.0	rt	THF	1 h	Decomposed
5	KO*t*-Bu	1.2	0 to rt	THF	1 h	(2.8:1) of (**8**:**S34**)
6	NaOMe	1.2	rt	MeOH	1 h	No reaction
7	NaOMe	1.2	60	MeOH	1 h	No reaction
8	KO*t*-Bu	1.2	0	THF	1 h	No reaction
9	KO*t*-Bu	1.2	rt	Tol	12 h	No reaction
10	KO*t*-Bu	3.0	rt	Tol	12 h	Decomposed

7. Characterization of compounds

4-Methyl-*N*-(phenyl-l3-iodaneylidene)benzenesulfonamide, PhI=NTs

PhI=NTs

p-Toluenesulfonamide (5.13 g, 29.8 mmol, 1.0 equiv.), potassium hydroxide (4.20 g, 74.9 mmol, 2.51 equiv.) and methanol (120 mL) were stirred in a conical flask in an ice bath, ensuring the reaction mixture maintained at 0 °C. Iodobenzene diacetate (9.60 g, 29.8 mmol, 1.0 equiv.) was added to the stirred mixture and the resulting yellow solution was stirred at room temperature for 3.5 h. The reaction mixture was poured into a large excess of ice water and stirred for 1 h. A yellow coloured solid precipitated on standing overnight. The light-yellow solid was isolated by filtration and dried with a flow of air through the Buchner funnel. Several portions of ether, in which the product is insoluble, were used to wash away any iodobenzene present. The yellow solid was then dissolved in a minimum of hot methanol, then allowed to cool to room temperature, and then placed in a freezer overnight whereupon an off-white solid **PhI=NTs** (7.95 g, 21.3 mmol, 71%) was isolated via filtration; **R$_f$** 0.22 (DCM/MeOH 10:1), the NMR of which is identical with that reported in literature;[15] **^{1}H NMR** (500 MHz, DMSO-d_6) δ_H 7.81-7.62 (m, 2H), 7.45 (dd, *J* = 7.6, 5.5 Hz, 2H), 7.29 (t, *J* = 7.7 Hz, 2H), 7.06 (d, *J* = 7.9 Hz, 2H), 2.27 (s, 3H); **^{13}C NMR** (125 MHz, DMSO-d_6) δ_C 142.6, 140.5, 133.6, 130.9, 130.6, 129.1, 126.6, 117.6, 21.2.

7-Tosyl-7-azabicyclo[4.1.0]hept-2-ene, 8

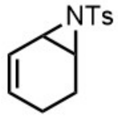

PhI=NTs (3.73 g, 10.0 mmol, 1.0 equiv.) was added to a solution of 1,3-cyclohexadiene (800 mg, 10.0 mmol, 1.0 equiv.) and Cu(acac)$_2$ (262 mg, 1.0 mmol, 0.1 equiv.) in acetonitrile (16 mL) under argon. The reaction was stirred for 30 min, then quenched with 1 M NaOH (3 mL) and extracted with diethyl ether (3 x 10 mL). The organic layer was dried (Na_2SO_4) and the solvent was evaporated. The residue was purified by flash column chromatography (gradient; pentane/EtOAc 20:1 to 10:1) to obtain the title compound **8** (1.48 g, 5.93 mmol, 59%) as a white solid. **R$_f$** 0.18 (pentane/EtOAc 3:1); the NMR of which is identical with that reported in literature;[16] **^{1}H NMR** (500 MHz, $CDCl_3$) δ_H 7.80 (d, *J* = 8.0 Hz, 2H), 7.31 (d, *J* = 8.0 Hz, 2H), 5.88 (ddd, *J* = 11.4, 4.2, 2.0 Hz, 2H), 3.30 (dd, *J* = 6.8, 3.1 Hz, 1H), 3.16 (ddd, *J* = 6.8, 4.4, 1.5 Hz, 1H), 2.42 (s, 3H), 2.09-2.04 (m, 1H), 2.03-1.96 (m, 2H), 1.56-1.47 (m, 1H); **^{13}C NMR** (125 MHz, $CDCl_3$) δ_C 144.3, 135.6, 133.6, 129.7, 127.8, 120.5, 41.7,

36.7, 21.7, 20.5, 18.7.

4-Methyl-*N*-(2-oxocyclohex-3-en-1-yl)benzenesulfonamide, 7

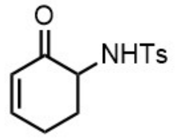

To a solution of **8** (25 mg, 0.10 mmol, 1.0 equiv.) in 1,2-dichloroethane (1.0 mL) was added DMSO (157 mg, 2.00 mmol, 20.0 equiv.). The mixture was stirred overnight at 60 °C, at which point the reaction had reached completion as judged by TLC. Then the reaction mixture was cooled to room temperature, diluted with water, the layers were separated and the aqueous layer extracted with ether (3 × 10 mL). The combined organic layers were dried over Na_2SO_4 and concentrated in vacuo. Silica gel flash chromatography (pentane/EtOAc 1:1) afforded the title compound **7** as a white solid (24.5 mg, 0.092 mmol, 92%); **Rf** 0.25 (pentane/EtOAc 2:1); NMR identical to that reported in literature;[17] **^{1}H NMR** (500 MHz, $CDCl_3$) δ_H 7.80-7.71 (m, 2H), 7.29 (d, J = 8.1 Hz, 2H), 7.03-6.94 (m, 1H), 6.00 (ddd, J = 10.0, 2.6, 1.3 Hz, 1H), 5.88-5.80 (m, 1H), 3.67 (ddd, J = 14.0, 5.1, 2.9 Hz, 1H), 2.64-2.55 (m, 1H), 2.51-2.45 (m, 2H), 2.40 (s, 3H), 1.98-1.86 (m, 1H); **^{13}C NMR** (125 MHz, $CDCl_3$) δ_C 194.5, 152.1, 143.8, 136.3, 129.9, 127.5, 127.3, 58.1, 31.4, 25.8, 21.7.

Alternatively, to a dry, N_2-flushed round bottomed flask was added **8** (3.95 g, 15.9 mmol, 1.0 equiv.) and added DMSO (25 mL). The mixture was stirred overnight at 60 °C, at which point the reaction had reached completion as judged by TLC. Then the reaction mixture was cooled to room temperature, diluted with water (5 mL), the layers were separated and the aqueous layer extracted with ethyl acetate (3 × 30 mL). The combined organic layers were dried over Na_2SO_4 and concentrated in vacuo to afford 3.65 g of compound **7**, which was of sufficient purity to progress directly to the next step without further purification.

(But-3-yn-1-yloxy)(*tert*-butyl)dimethylsilane, S35

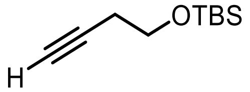

To a solution of 3-butyn-1-ol (1.42 g, 20 mmol, 1.0 equiv.) and imidazole (2.00 g, 29.35 mmol, 1.46 equiv.) in tetrahydrofuran (30 mL) was added *tert*-butyldimethylsilyl chloride (TBSCl) (3.32 g, 21.8

mmol, 1.09 equiv.), After stirring at ambient temperature for 3 h, the reaction mixture was filtered through a pad of silica and concentrated under reduced pressure. Gradient flash chromatography (pentane/EtOAc, 15:1) afforded the alkyne **S35** (3.20 g, 17.4 mmol, 87%) as a clear colorless oil; $\mathbf{R_f}$ 0.27 (pentane); the NMR of which is identical with that reported in literature;[18] **^{1}H NMR** (400 MHz, $CDCl_3$) δ_H 3.74 (t, J = 7.1 Hz, 2H), 2.40 (td, J = 7.1, 2.7 Hz, 2H), 1.95 (t, J = 2.7 Hz, 1H), 0.90 (s, 9H), 0.07 (s, 6H); **^{13}C NMR** (100 MHz, $CDCl_3$) δ_C 81.7, 69.4, 61.9, 26.0, 23.0, 18.5, -5.2.

[(4-Bromobut-3-yn-1-yl)oxy](*tert*-butyl)dimethylsilane, 10

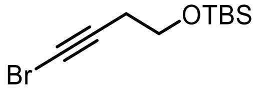

S35 (1.00 g, 5.43 mmol, 1.0 equiv.) was dissolved in acetone (10.0 mL) at room temperature. Then NBS (1.16 g, 6.52 mmol, 1.2 equiv.) and $AgNO_3$ (185 mg, 1.09 mmol, 0.2 equiv.) were added at the same temperature. The mixture was shielded from light and stirred for 3 h, then concentrated in vacuo. The residue was purified by flash column chromatography (pentane) to afford **10** (1.30 g, 4.96 mmol, 92%); $\mathbf{R_f}$ 0.39 (pentane); **IR** (thin film, ν_{max} / cm^{-1}) 2956, 2929, 2858, 2360, 1472, 1258, 1107, 912, 837, 808, 777, 664; **^{1}H NMR** (500 MHz, $CDCl_3$) δ_H 3.73 (t, J = 7.0 Hz, 2H), 2.41 (t, J = 7.0 Hz, 2H), 0.90 (s, 9H), 0.07 (s, 6H); **^{13}C NMR** (125 MHz, $CDCl_3$) δ_C 77.7, 61.6, 39.2, 26.0, 24.2, 18.5, -5.2; **HRMS** (ES$^+$) calc. for $C_{10}H_{20}BrOSi$ [M+H]$^+$ 263.0461, found 263.0461; Data identical to literature values.[18]

***N*-{2-[(*Tert*-butyldimethylsilyl)oxy]cyclohex-3-en-1-yl}-4-methylbenzenesulfonamide, 9**

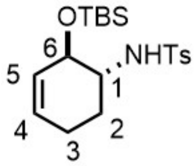

To a stirred solution of enone **7** (851 mg, 3.21 mmol, 1.0 equiv.) in MeOH (9.0 mL) at 0 °C was added $NaBH_4$ (146 mg, 3.85 mmol, 1.2 equiv.), then the reaction mixture was allowed to warm to room temperature and stirred for 1 h. The reaction was quenched by addition of NH_4Cl (2 mL), and the it was extracted with ether, filtered, and concentrated in vacuo to furnish the crude amino alcohol, which was used directly for next step without further purification.

To a stirred solution of crude amino alcohol in CH_2Cl_2 (5.0 mL) at 0 °C was added 2,6-lutidine (0.45

mL, 3.83 mmol, 1.4 equiv.), and then (dropwise) TBSOTf (0.76 mL, 3.289 mmol, 1.2 equiv.). The mixture was allowed to warm to room temperature and stirred for 1 h. The reaction mixture was quenched by the addition of *sat*. NH_4Cl *aq*., then the mixture was extracted with Et_2O, washed with brine, and concentrated. The residue was purified by flash chromatography on silica gel (pentane/EtOAc 15:1) to give the title compound **9** (590 mg, 1.55 mmol, 48%) as a colourless oil; $\mathbf{R_f}$ 0.27 (pentane/EtOAc 15:1); **IR** (thin film, ν_{max} / cm^{-1}) 3287, 2953, 2929, 2856, 2360, 2341, 1471, 1328, 1256, 1161, 1094, 1071, 864,777, 747, 667; ^{1}**H NMR** (400 MHz, $CDCl_3$) δ_H 7.76 (2H, d, J = 8.3 Hz, Ts*H*), 7.31-7.27 (2H, m, Ts*H*), 5.75 (1H, dtd, J = 10.1, 3.5, 1.1 Hz, H4), 5.52 (1H, ddt, J = 10.1, 3.7, 2.0 Hz, H5), 4.60 (1H, d, J = 6.8 Hz, NH), 3.87 (1H, ddt, J = 5.4, 4.0, 1.6 Hz, H6), 3.33-3.15 (1H, m, H1), 2.42 (3H, s, TsCH_3), 2.06 (1H, ddd, J = 8.0, 5.2, 2.8 Hz, H2), 2.03 (2H, dd, J = 2.8, 1.4 Hz, H3), 1.56-1.45 (1H, m, H2), 0.82 (9H, s, SiC(CH_3)$_3$), -0.00 (6H, d, J = 2.9 Hz, Si(CH_3)$_2$); ^{13}**C NMR** (100 MHz, $CDCl_3$) δ_C 143.5, 137.8, 130.0, 129.83, 127.7, 127.3, 69.0, 55.1, 25.9, 24.2, 22.4, 21.6, 18.1, -4.4, -4.6; **HRMS** (ES^+) calc. for $C_{19}H_{32}NO_3SSi$ $[M+H]^+$ 382.1872, found 382.1868.

Scaled up synthesis of 9

To a stirred solution of enone **7** (7.20 g, 27.2 mmol, 1.0 equiv.) in MeOH (55 mL) at 0 °C was added $NaBH_4$ (1.23 g, 32.6 mmol, 1.2 equiv.). The mixture was stirred for 30 min, then quenched by addition of *sat*. NH_4Cl (5 mL). The mixture was extracted with ether, then filtered and concentrated in vacuo to furnish the crude alcohol, which was used directly in the next step without further purification.

To a stirred solution of crude alcohol in DMF (48 mL) was added imidazole (2.78 g, 40.8 mmol, 1.5 equiv.) and TBSCl (4.91 g, 32.6 mmol, 1.2 equiv.) at room temperature, then the mixture was warmed to 40 °C and stirred for 1 h. The mixture was cooled to room temperature, then it was quenched by the addition of 30 mL H_2O. The mixture was then extracted with EtOAc (90 mL x 2), washed with brine, and the combined organic extracts were dried ($MgSO_4$) and concentrated. The residue was purified by flash chromatography on silica gel (pentane/EtOAc 15:1) to give the title compound **9** (10.36 g, 28.2 mmol, 100%) as a colourless oil; NMR data identical to above values.

(±)-*N*-[(1*R*,2*R*)-2-Hydroxycyclohex-3-en-1-yl]-4-methylbenzenesulfonamide, S36

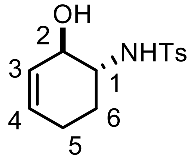

To a 0.067 M solution of *N*-tosylaziridine **8** (250 mg, 1.0 mmol, 1.0 equiv.) in acetone:H_2O (1:1; total volume 15.0 mL), was added $NaHSO_3$ (208 mg, 2.0 mmol, 2.0 equiv.). The solution was heated at 60 °C until consumption of starting material (about 30 min). Then, the reaction mixture was concentrated, and the remnant aqueous layer was extracted with CH_2Cl_2. The organic layers were combined and dried over Na_2SO_4, and then filtered and concentrated. Silica gel flash chromatography (pentane/EtOAc 3:1) gave the title compound **S36** as a white solid (171 mg, 0.64 mmol, 64%); **mp** 125-127 °C; **R_f** 0.18 (pentane/EtOAc 3:1); **IR** (thin film, ν_{max} / cm^{-1}) 3515, 3276, 2922, 1598, 1439, 1323, 1157, 1092, 1034, 938, 906, 851, 815, 738, 665; **^{1}H NMR** (400 MHz, $CDCl_3$) δ_H 7.79 (2H, d, *J* = 8.3 Hz, Ts*H*), 7.29 (2H, d, *J* = 8.0 Hz, Ts*H*), 5.71-5.63 (1H, m, H4), 5.59-5.54 (2H, m, H3, O*H*), 4.09-4.01 (1H, m, H2), 3.15-3.06 (2H, 2H, H1, N*H*), 2.40 (3H, s, TsC*H*3), 2.03-1.96 (2H, m, H5), 1.77-1.68 (1H, m, H6), 1.56-1.45 (1H, m, H6); **^{13}C NMR** (100 MHz, $CDCl_3$) δ_C 143.7, 137.3, 129.9, 129.8, 128.2, 127.2, 70.6, 56.7, 26.7, 24.3, 21.6; **HRMS** (ES^+) calc. for $C_{13}H_{18}NO_3S$ $[M+H]^+$ 268.0999, found 268.1002.

(±)-*N*-{4-[(*Tert*-butyldimethylsilyl)oxy]but-1-yn-1-yl}-*N*-{2-[(*tert*-butyldimethylsilyl) oxy]cyclo hex-3-en-1-yl}-4-methylbenzenesulfonamide, 6

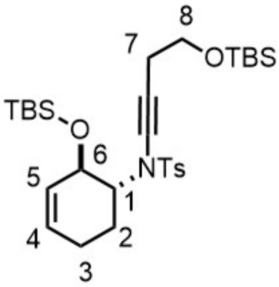

To a dry, nitrogen-flushed round bottomed flask equipped with a magnetic stirring bar and a septum was added **9** (307 mg, 0.81 mmol, 1.0 equiv.), toluene (4.7 mL, 0.17 M), coupling partner **10** (317 mg, 1.21 mmol, 1.5 equiv.) and Cs_2CO_3 (528 mg, 1.62 mmol, 2.0 equiv.). The reaction mixture was stirred at rt for 30 min, then CuI (46 mg, 0.24 mmol, 0.3 equiv.) and DMEDA (0.01 mL, 0.97 mmol, 1.2 equiv.) were added. The reaction was stirred at 70 °C until the starting material was consumed

(12 h, as monitored by TLC). Upon cooling to room temperature, the reaction mixture was filtered through celite and concentrated. The resulting residue was purified by flash column chromatography (gradient, pentane/EtOAc 100:1 to 50:1) to give **6** (200 mg, 44%, 66% brsm); $\mathbf{R_f}$ 0.30 (pentane/EtOAc 40:1); **IR** (thin film, ν_{max} / cm^{-1}) 3032, 2953, 2928, 2856, 2360, 2251, 1471, 1364, 1252, 1169, 1100, 1055, 835, 662; ^{1}**H NMR** (500 MHz, $CDCl_3$) δ_H 7.79 (2H, d, *J* = 8.4 Hz, Ts*H*), 7.29 (2H, d, *J* = 8.1 Hz, Ts*H*), 5.62 (1H, dq, *J* = 7.5, 2.8 Hz, H4), 5.52 (1H, dp, *J* = 10.0, 1.4 Hz, H5), 4.33 (1H, ddt, *J* = 7.4, 3.7, 1.9 Hz, H6), 3.87 (1H, ddd, *J* = 12.9, 8.5, 3.4 Hz, H1), 3.68 (2H, t, *J* = 7.3 Hz, H8), 2.51 (2H, t, *J* = 7.3 Hz, H7), 2.43 (3H, s, TsCH_3), 2.15-2.07 (1H, m, H3), 2.04-1.95 (1H, m, H3), 1.79-1.67 (1H, m, H2), 1.42-1.32 (1H, m, H2), 0.88 (18H, d, *J* = 2.8 Hz, 2x SiC(CH_3)$_3$), 0.12-0.01 (12H, m, 2 x Si(CH_3)$_2$); ^{13}**C NMR** (125 MHz, $CDCl_3$) δ_C 144.1, 136.7, 130.7, 129.7, 127.6, 127.4, 71.7, 69.3, 68.5, 62.7, 62.4, 26.0, 25.7, 25.1, 23.2, 21.7, 18.4, 18.2, -4.4, -4.6, -5.18, -5.19; **HRMS** (ES^+) calc. for $C_{29}H_{49}NNaO_4SSi_2$ $[M+Na]^+$ 586.2813, found 586.2811.

(±)-(1*R*,5*R*,*E*)-8-[(*Tert*-butyldimethylsilyl)oxy]-7-{3-[(*tert*butyldimethylsilyl)oxy]propylidenee}-6-tosyl-6-azabicyclo[3.2.1]oct-2-ene, 11

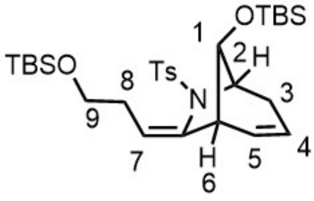

To a solution of ynamide **6** (345 mg, 0.613 mmol, 1.0 equiv.) in dry toluene (5.0 mL) in a dry, argon flushed flask, was added $Pd(OAc)_2$ (14 mg, 61.3 µmol, 0.1 equiv.) and bbeda (15 mg, 61.3 µmol, 0.1 equiv.). The mixture was stirred for 1 h at 60 °C, then cooled to room temperature and concentrated. Purification by column chromatography (pentane/EtOAc 40:1) gave **11** (267 mg, 0.474 mmol, 77%) as a light-yellow oil; $\mathbf{R_f}$ 0.34 (pentane/EtOAc 40:1); **IR** (thin film, ν_{max} / cm^{-1}) 3038, 2954, 2928, 2856, 2360, 1681, 1463, 1213, 1162, 1094, 914, 874, 835, 813,776 736, 706, 624; ^{1}**H NMR** (500 MHz, $CDCl_3$) δ_H 7.68 (2H, d, *J* = 8.3 Hz, Ts*H*), 7.31-7.26 (2H, m, Ts*H*), 5.65 (1H, ddq, *J* = 8.2, 6.4, 1.7 Hz, H5), 5.59-5.52 (1H, m, H4), 4.58 (1H, t, *J* = 7.1 Hz, H7), 4.25-4.19 (1H, m, H1), 3.97 (1H, ddd, *J* = 5.8, 4.4, 1.3 Hz, H2), 3.41 (2H, qt, *J* = 9.9, 6.4 Hz, H9), 2.72 (1H, ddd, *J* = 6.2, 4.5, 1.4 Hz, H6), 2.47 (2H, d, *J* = 2.6 Hz, H8), 2.41 (3H, s, TsCH_3), 2.38-2.30 (1H, m, H3), 2.30-2.21 (1H, m, H3), 0.86 (9H, s, SiC(CH_3)$_3$), 0.84 (9H, s, SiC(CH_3)$_3$), 0.00 (12H, d, *J* = 11.4 Hz, 2 x Si(CH_3)$_2$); ^{13}**C NMR** (125 MHz, $CDCl_3$) δ_C 143.5, 140.6, 138.4, 129.8, 126.9, 126.5, 124.9, 104.0, 67.9, 63.2, 62.1,

48.6, 31.7, 29.3, 26.1, 26.0, 25.8, 21.7, 18.5, 18.1, -4.8, -4.9, -5.10, -5.12; **HRMS** (ES^+) calc. for $C_{29}H_{50}NO_4SSi_2$ $[M+Na]^+$ 564.2994, found 564.2994.

4-Methyl-*N*-(2-methylenecyclohex-3-en-1-yl)benzenesulfonamide, 12

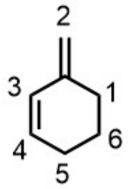

n-BuLi (1.97 M in hexanes, 6.27 mL, 12.35 mmol, 3.0 equiv.) was added dropwise to a stirred suspension of trimethylsulfonium iodide (2.52 g, 12.35 mmol, 3.0 equiv.) in THF (16 mL) at –10 ºC, and the mixture was stirred for 15 min. A solution of aziridine **8** (1.03 g, 4.12 mmol, 1.0 equiv.) in THF (5.0 mL) was added dropwise and the reaction was allowed to warm to 5 ºC, stirring for 1 h. After quenching with H_2O (5.0 mL), the layers were separated. The aqueous layer was extracted with Et_2O (3 × 20 mL), then the combined organic layers were dried over Na_2SO_4 and concentrated in vacuo. Silica gel flash chromatography (pentane/EtOAc 10:1) afforded the title compound **12** (1.03 g, 3.93 mmol, 95%) as white solid; **mp** 103-105 °C; $\mathbf{R_f}$ 0.20 (pentane/EtOAc 10 :1); **IR** (thin film, ν_{max} / cm^{-1}) 3277, 2921, 2360, 1434, 1327, 1159, 1093, 913, 800, 665; **^{1}H NMR** (400 MHz, $CDCl_3$) δ_H 7.77 (2H, d, *J* = 8.1 Hz, Ts*H*), 7.30 (2H, d, *J* = 8.0 Hz, Ts*H*), 6.04 (1H, dt, *J* = 10.0, 2.1 Hz, H4), 5.86-5.74 (1H, m, H3), 4.76 (2H, d, *J* = 12.4 Hz, H2), 4.47 (1H, d, *J* = 8.0 Hz, N*H*), 3.98 (1H, td, *J* = 8.0, 3.9 Hz, H1), 2.43 (3H, s, TsCH_3), 2.18 (2H, q, *J* = 5.5 Hz, H5), 1.85-1.69 (2H, m, H6); **^{13}C NMR** (100 MHz, $CDCl_3$) δ_C 143.5, 142.4, 138.2, 130.2, 129.8, 127.9, 127.2, 112.6, 53.2, 29.8, 23.3, 21.7; **HRMS** (ES+) calc. for $C_{14}H_{17}NNaO_2S$ $[M+Na]^+$ 286.0872, found 286.0872.

Phenyl(trimethylsilylethynyl)iodonium triflate, S1

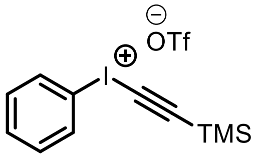

phenyliodonium diacetate (1.6 g, 5.0 mmol, 1.0 equiv.) was diluted with CH_2Cl_2 (5.0 mL) in a flask and the mixture was stirred 5 min. Tf_2O (0.42 mL, 2.5 mmol, 0.50 equiv.) was added dropwise at 0 °C and the resulting yellow mixture was stirred 30 min bis(trimethylsilyl)acetylene (852 mg, 5.00 mmol, 1.0 equiv.) was added. The mixture was then stirred 2 h at the same temperature, after concentrating the solvent, diethyl ether was added to precipitate the white product. Filtration afforded **S1** (1.71 g, 3.79 mmol, 76%) as colorless crystals. **^{1}H NMR** (400 MHz, $CDCl_3$) δ_H 8.11-8.01 (m, 2H), 7.69-7.60

(m, 1H), 7.52 (tt, J = 8.0, 1.4 Hz, 2H), 0.32 – -0.05 (m, 9H); **^{13}C NMR** (100 MHz, $CDCl_3$) δ_C 134.1, 134.1, 132.7, 132.6, 132.5, 124.7, 121.5, 119.6, 118.4, 116.7, 115.2, 44.0, -0.8; Data identical to literature values[19]

(*E*)-*N*-(1,2-Dichlorovinyl)-4-methyl-*N*-(2-methylenecyclohex-3-en-1-yl)benzenesulfonamide, 14

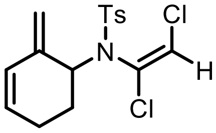

To a mixture of **12** (351 mg, 1.34 mmol, 1.0 equiv.), powdered Cs_2CO_3 (1.31 g, 4.02 mmol, 3.0 equiv.) in DMF (5.0 mL) in a dry, argon flushed flask, was added dropwise trichloroethylene (0.36 mL, 4 mmol, 3.0 equiv.). The resulting mixture was stirred at 50 ºC for 1 h under an Ar atmosphere. The mixture was then cooled to room temperature and ethyl acetate and water (2:1, 9.0 mL total) were added. The layers were separated and the aqueous layer extracted with EtOAc (3 × 10 mL). The combined organic layers were dried over Na_2SO_4 and concentrated in vacuo. Silica gel flash chromatography (pentane/EtOAc 6:1) afforded the title compound **14** (472 mg, 1.32 mmol, 99%) as a yellow oil; **Rf** 0.48 (pentane/EtOAc 6:1); **IR** (thin film, νmax / cm-1) 2360, 2342, 1598, 1356, 1165, 1089, 990, 906, 821, 668, 630; **^{1}H NMR** (500 MHz, $CDCl_3$) (1.7:0.3 mixture of rotamers) δ_H 7.92-7.80 (m, 2H), 7.31 (d, J = 7.9 Hz, 2H), 6.46 (s, 1H), 6.03 (s, 1H), 5.77 (s, 1H), 5.37 (s, 0.3H), 5.02-4.76 (m, 1.7H), 4.53 (s, 1H), 2.44 (s, 3H), 2.41-2.28 (m, 1H), 2.26 (s, 1H), 2.19-2.07 (m, 1H), 1.97-1.77 (m, 1H); **^{13}C NMR** (125 MHz, $CDCl_3$) δ_C 144.5, 138.9, 137.1, 136.5, 130.5, 129.8, 129.6, 128.6, 128.3, 122.4, 117.0, 116.6, 60.8, 27.7, 23.4, 21.7; HRMS (ES+) calc. for $C_{16}H_{17}Cl_2NNaO_2S$ $[M+Na]^+$ 380.0249, found 380.0250.

***N*-Ethynyl-4-methyl-*N*-(2-methylenecyclohex-3-en-1-yl)benzenesulfonamide, 13**

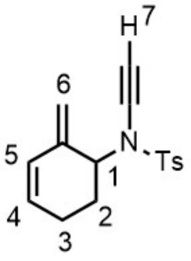

To a dry, argon flushed flask was added dichloroenamide **14** (132 mg, 0.37 mmol, 1.0 equiv.) and anhydrous THF (2.0 mL). The stirred mixture was cooled to -78 °C, then PhLi (1.9 M in dibutyl ether, 0.54 mL, 1.02 mmol, 2.75 equiv.) was added dropwise and the reaction stirred for 1 h. After total

conversion to the organolithium intermediate (as confirmed by consumption of starting material by TLC), the reaction was quenched by addition of saturated NH_4Cl solution (1.0 mL). The mixture was warmed to room temperature, diluted with water, the layers were separated and the aqueous layer extracted with diethyl ether (3 × 5 mL). The combined organic layers were dried over Na_2SO_4 and concentrated in vacuo. Silica gel flash chromatography (pentane/EtOAc 20:1) afforded the title compound **13** (43 mg, 0.15 mmol, 41%) as a yellow oil; **R_f** 0.29 (pentane/EtOAc 20:1); **IR** (thin film, ν_{max} / cm^{-1}) 3298, 2361, 2341, 2128, 1362, 1260, 1169,1091, 1019, 798, 750, 669; **^{1}H NMR** (500 MHz, $CDCl_3$) δ_H 7.84 (2H, d, *J* = 8.4 Hz, Ts*H*), 7.35 (2H, d, *J* = 8.0 Hz, Ts*H*), 6.12 (1H, dt, *J* = 9.8, 1.8 Hz, H4), 5.76 (1H, dt, *J* = 9.3, 4.1 Hz, H5), 4.99-4.89 (1H, m, H6), 4.82 (1H, s, H6), 4.61 (1H, ddt, *J* = 12.3, 4.5, 2.2 Hz, H1), 2.74 (1H, s, H7), 2.46 (3H, s, TsC*H*3), 2.30-2.18 (2H, m, H3), 1.95 (1H, tdd, *J* = 12.3, 10.4, 6.1 Hz, H2), 1.67 (1H, dq, *J* = 12.5, 4.2 Hz, H2); **^{13}C NMR** (125 MHz, $CDCl_3$) δ_C 144.9, 139.3, 135.8, 130.0, 129.2, 129.1, 127.7, 112.7, 73.7, 61.0, 59.4, 27.8, 25.1, 21.8; **HRMS** (ES^+) calc. for $C_{16}H_{17}NNaO_2S$ $[M+Na]^+$ 310.0872, found 310.0872.

4-Methyl-*N*-(2-methylenecyclohex-3-en-1-yl)-*N*-(prop-1-yn-1-yl)benzenesulfonamide, S5

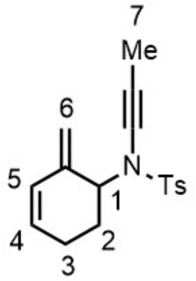

To a dry, argon flushed flask was added dichloroenamide **14** (128 mg, 0.36 mmol, 1.0 equiv.) and anhydrous THF (1.0 mL). The stirred mixture was cooled to -78 °C, then PhLi (1.9 M in dibutyl ether, 0.52 mL, 0.99 mmol, 2.75 equiv.) was added dropwise and the reaction was stirred for 1 h. After total conversion to the organolithium intermediate (as confirmed by TLC), MeI (67 µL, 1.08 mmol, 3 equiv.) was added, then the mixture was allowed to warm up to room temperature and stirred for 1 h. The reaction was then quenched by addition of saturated NH_4Cl solution (1.0 mL), diluted with water, the layers were separated and the aqueous layer extracted with diethyl ether (3 × 5 mL). The combined organic layers were dried over Na_2SO_4 and concentrated in vacuo. Silica gel flash chromatography (pentane/EtOAc 20:1) afforded the title compound **S5** (88 mg, 0.292 mmol, 82%) as a yellow oil; **R_f** 0.29 (pentane/EtOAc 20:1); **IR** (thin film, ν_{max} / cm^{-1}) 3042, 2361, 2342, 1361, 1168, 1092, 1061, 1031, 913, 815, 742, 660; **^{1}H NMR** (500 MHz, $CDCl_3$) δ_H 7.82 (2H, d, *J* = 8.3 Hz, Ts*H*), 7.37-7.30

(2H, m, Ts*H*), 6.12 (1H, dd, *J* = 10.1, 2.2 Hz, H4), 5.74 (1H, dt, *J* = 8.6, 4.0 Hz, H5), 4.97-4.90 (1H, m, H6), 4.85 (1H, s, H6), 4.60 (1H, ddt, *J* = 12.4, 4.4, 2.2 Hz, H1), 2.45 (3H, s, TsCH_3), 2.26-2.19 (2H, m, H3), 1.96-1.89 (1H, m, H2), 1.88 (3H, s, H7), 1.71-1.57 (1H, m, H2); **^{13}C NMR** (125 MHz, $CDCl_3$) δ_C 144.4, 139.8, 136.2, 129.8, 129.3, 129.0, 127.6, 112.6, 69.3, 67.4, 59.3, 27.9, 25.3, 21.8, 3.6, 1.2; **HRMS** (ES^+) calc. for $C_{17}H_{19}NNaO_2S$ $[M+Na]^+$ 324.1029, found 324.1028.

(±)-(1*S*,5*R*)-7,8-Dimethylene-6-tosyl-6-azabicyclo[3.2.1]oct-3-ene, 15

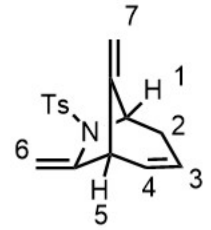

To a solution of ynamide **13** (30 mg, 104.5 µmol, 1.0 equiv.) in dry toluene (1.5 mL) in a dry, argon flushed flask, was added $Pd(OAc)_2$ (12 mg, 52.2 µmol, 0.5 equiv.) and bbeda (13 mg, 52.2 µmol, 0.5 equiv.). The mixture was stirred for 0.5 h at room temperature, then concentrated and purified by column chromatography (pentane/EtOAc 10:1) to afford **15** (6.0 mg, 20.9 µmol, 20%) as a colourless oil; **R_f** 0.18 (pentane/EtOAc 10:1); **IR** (thin film, ν_{max} / cm^{-1}) 3031, 2361, 2341, 1771, 1654, 1599, 1351, 1158, 1095, 985, 911, 816, 734, 669; **^{1}H NMR** (400 MHz, $CDCl_3$) δ_H 7.79 (2H, d, *J* = 8.4 Hz, Ts*H*), 7.36-7.28 (2H, m, Ts*H*), 6.11 (1H, dt, *J* = 9.8, 1.9 Hz, H4), 5.75 (1H, dt, *J* = 9.2, 4.2 Hz, H3), 5.14 (1H, s, H6), 5.08 (1H, d, *J* = 2.1 Hz, H7), 5.00 (1H, d, *J* = 2.2 Hz, H6), 4.94 (1H, d, *J* = 2.1 Hz, H7), 4.79-4.71 (1H, m, H1), 2.44 (3H, s, TsCH_3), 2.28-2.21 (1H, m, H5), 1.96-1.87 (1H, m, H2), 1.74 (1H, dq, *J* = 12.4, 4.1 Hz, H2); **^{13}C NMR** (100 MHz, $CDCl_3$) δ_C 143.9, 142.1, 141.5, 137.5, 129.7, 129.5, 129.0, 127.9, 113.2, 107.2, 59.8, 27.9, 25.7, 21.7; **HRMS** (ES^+) calc. for $C_{16}H_{18}NO_2S$ $[M+H]^+$ 288.1053, found 288.1054.

1-Tosyl-1*H*-indole-3-carbaldehyde, S37

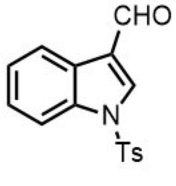

To a stirred solution of 1*H*-indole-3-carbaldehyde (9.2 g, 63.4 mmol, 1.00 equiv.) in CH_2Cl_2 (86 mL) at 0 °C was added Et_3N (26.5 mL, 190.1 mmol, 3.00 equiv.) and TsCl (12.32 g, 64.6 mmol, 1.02 equiv.). The mixture was allowed to warm up to room temperature, and stirred for 1 h. *Sat.* NH_4Cl

aq. (20 mL) was then added to quench the reaction. The layers were separated and the aqueous layer extracted with CH_2Cl_2 (3 × 30 mL). The combined organic layers were dried over Na_2SO_4 and concentrated in vacuo. Silica gel flash chromatography (pentane/EtOAc 3:1) afforded the title compound **S37** (18.8 g, 63.0 mmol, 98%) as a solid; **R_f** 0.39 pentane/EtOAc 3:1; **^{1}H NMR** (400 MHz, $CDCl_3$) δ_H 10.09 (s, 1H), 8.27-8.24 (m, 1H), 8.23 (s, 1H), 7.95 (dd, *J* = 8.3, 0.9 Hz, 1H), 7.85 (d, *J* = 8.4 Hz, 2H), 7.44-7.32 (m, 2H), 7.33-7.27 (m, 2H), 2.37 (s, 3H); **^{13}C NMR** (100 MHz, $CDCl_3$) δ_C 185.5, 146.3, 136.4, 135.3, 134.5, 130.4, 127.4, 126.43, 126.41, 125.2, 122.7, 122.5, 113.4, 21.8; Data identical to literature values.[20]

3-(2,2-Dibromovinyl)-1-tosyl-1*H*-indole, S38

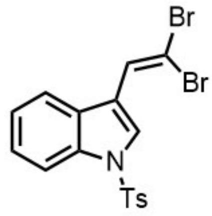

To a solution of PPh_3 (55.6 g, 200 mmol, 6.0 equiv.) in CH_2Cl_2 (167 mL) at –20 °C was added CBr_4 (533 g, 100 mmol, 3.0 equiv.) in a single portion. The mixture was stirred for 10 min, then cooled to –78 °C before dropwise addition of a solution of *N*-tosylindole-3-carboxaldehyde **S37** (10 g, 33.5 mmol, 1.0 equiv.) in CH_2Cl_2 (30 mL). Stirring was continued for 5 min, then the mixture was warmed to rt before addition of hexane (80 mL). The precipitate was filtered, concentrated *in vacuo*, redissolved in $CHCl_3$ (150 mL) and reconcentrated, then purified *via* rapid chromatography through a pad of silica (pentane/EtOAc 10:1) to afford the vinyl dibromide **S38** as a pale yellow solid (14.4 g, 31.8 mmol, 95%); **R_f** 0.48 (pentane/EtOAc 15:1); **^{1}H NMR** (400 MHz, $CDCl_3$) δ_H 8.32 (d, *J* = 0.9 Hz, 1H), 8.01 (dt, *J* = 8.3, 0.9 Hz, 1H), 7.81 (d, *J* = 8.4 Hz, 2H), 7.55 (d, *J* = 0.9 Hz, 1H), 7.53 (dt, *J* = 7.9, 1.0 Hz, 1H), 7.38 (ddd, *J* = 8.4, 7.3, 1.3 Hz, 1H), 7.32-7.28 (m, 1H), 7.25 (d, *J* = 8.1 Hz, 2H), 2.35 (s, 3H); **^{13}C NMR** (100 MHz, $CDCl_3$) δ_C 145.4, 134.9, 134.1, 130.1, 129.5, 126.98, 126.97, 125.5, 125.1, 123.7, 118.9, 117.4, 113.7, 90.4, 21.7; Data identical to literature values.[20]

3-(Bromoethynyl)-1-tosyl-1*H*-indole, 16

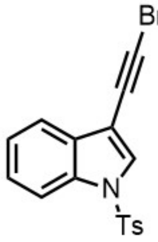

To a solution of vinyl dibromide **S38** (10.0 g, 22.1 mmol, 1.0 equiv.) in THF (110 mL) at –78 °C was added LiHMDS (1.0 M solution in THF, 26.5 mL, 26.5 mmol, 1.2 equiv.) dropwise and the mixture was stirred for 20 min, before being quenched by addition of *sat*. NH_4Cl (30 mL). The product was extracted with EtOAc (2 × 50 mL), then the organic fractions were combined, dried (Na_2SO_4), filtered and concentrated, giving the bromoalkyne **16** as a yellow solid (8.10 g, 21.7 mmol, 98%) which was used without further purification. **^{1}H NMR** (400 MHz, $CDCl_3$) δ_H 7.97 (dt, *J* = 8.3, 0.9 Hz, 1H), 7.78 (d, *J* = 8.5 Hz, 3H), 7.64 (ddd, *J* = 7.7, 1.3, 0.8 Hz, 1H), 7.37 (ddd, *J* = 8.4, 7.3, 1.4 Hz, 1H), 7.32-7.29 (m, 1H), 7.26-7.23 (m, 2H), 2.36 (s, 4H); **^{13}C NMR** (100 MHz, $CDCl_3$) δ_C 145.6, 134.9, 134.1, 130.8, 130.2, 130.0, 127.1, 125.7, 124.0, 120.6, 113.7, 104.9, 71.6, 53.6, 21.8; Data identical to literature values.[20]

(±)-*N*-{2-[(*Tert*-butyldimethylsilyl)oxy]cyclohex-3-en-1-yl}-4-methyl-*N*-[(1-tosyl-1*H*-indol-3-yl) ethynyl]benzenesulfonamide, 17

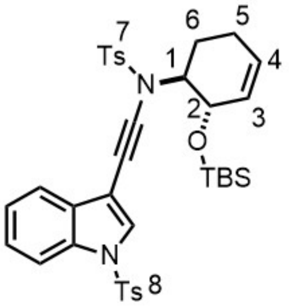

To a dry and nitrogen-flushed round bottomed flask, equipped with a magnetic stirring bar and a septum, were charged with **9** (4.80 g, 12.6 mmol, 1.0 equiv.), toluene (126 mL, 0.1 M), coupling partner **16** (13.1 g, 35.3 mmol, 2.8 equiv.) and Cs_2CO_3 (8.22 g, 25.2 mmol, 2.0 equiv.). The reaction mixture was stirred at rt for 5 min and then CuI (720 mg, 3.78 mmol, 0.3 equiv.) and DMEDA (1.64 mL, 15.1 mmol, 1.2 equiv.) were added. The reaction was stirred at 50 °C overnight. Upon cooling to room temperature, the reaction mixture was filtered through celite and concentrated. The residue was purified by flash column chromatography (pentane/EtOAc 15:1) to give **17** (6.98 g, 10.3 mmol, 82%); **R_f** 0.23 (pentane/EtOAc 15:1); **IR** (thin film, ν_{max} / cm^{-1}) 2928, 2525, 2360, 2341, 2238, 1597,

1447, 1373, 1249, 1171, 1130, 1092, 1000, 983, 908, 838, 743, 665; **^{1}H NMR** (400 MHz, $CDCl_3$) δ_H 7.96 (1H, dt, *J* = 8.3, 1.0 Hz, Ar*H*), 7.85 (2H, d, *J* = 8.3 Hz, Ts*H*), 7.80 (2H, d, *J* = 8.5 Hz, Ts*H*), 7.67 (1H, s, Ar*H*), 7.50 (1H, dt, *J* = 7.9, 1.0 Hz, Ar*H*), 7.37-7.31 (3H, m, 2 x Ts*H* + Ar*H*), 7.28-7.26 (2H, m, Ts*H*), 7.25 (1H, s, Ar*H*), 5.69-5.61 (1H, m, H3), 5.59-5.52 (1H, m, H4), 4.43 (1H, ddd, *J* = 7.8, 3.5, 1.8 Hz, H2), 4.02 (1H, ddd, *J* = 12.9, 8.5, 3.4 Hz, H1), 2.45 (3H, s, H7), 2.37 (3H, s, H8), 2.21-2.13 (1H, m, H5), 2.10-2.03 (1H, m, H5), 1.90-1.80 (1H, m, H6), 1.54-1.51 (1H, m, H6), 0.90 (9H, s, SiC(C*H*$_3$)$_3$), 0.11 (6H, d, *J* = 6.0 Hz, Si(C*H*$_3$)$_2$); **^{13}C NMR** (100 MHz, $CDCl_3$) δ_C 145.5, 144.6, 136.5, 135.1, 134.3, 131.4, 130.6, 130.3, 130.2, 129.9, 129.1, 127.8, 127.6, 127.1, 125.5, 123.8, 120.7, 113.7, 104.9, 83.9, 68.7, 63.7, 63.3, 26.0, 25.2, 21.8, 21.8, 18.2, -4.3, -4.5; **HRMS** (ES^+) calc. for $C_{36}H_{43}N_2O_5S_2Si$ $[M+H]^+$ 675.2377, found 675.2371.

(±)-3-{(*E*)-[(1*S*,5*R*,8*S*)-8-[(*Tert*-Butyldimethylsilyl)oxy]-6-tosyl-6-azabicyclo[3.2.1]oct-2-en-7-ylidene)methyl}-1-tosyl-1*H*-indole, 18

To a solution of ynamide **17** (6.30 g, 9.32 mmol, 1.0 equiv.) in a dry, argon flushed flask, was added $Pd(OAc)_2$ (313 mg, 1.40 mmol, 0.15 equiv.) and bbeda (330 mg, 1.40 mmol, 0.15 equiv.) and dry toluene (93 mL, c = 0.1 M). The mixture was stirred for 1 h at 60 °C, the cooled to room temperature, filtered through celite, and concentrated. The residue was purified by flash column chromatography (pentane/EtOAc 30:1 to 15:1) to afford **18** (4.60 g, 6.80 mmol, 73%) as an 8:1 mixture of (barely separable) ***Z*** and ***E*** isomers as a green foam.

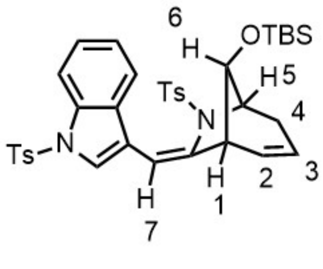

Z isomer: **R$_f$** 0.29 (pentane/EtOAc 6:1); **IR** (thin film, ν_{max} / cm^{-1}) 2954, 2929, 2897, 2857, 1739, 1651, 1597, 1447, 1371, 1249, 1172, 1092, 1048, 979, 875, 837, 813, 779, 745, 669; **^{1}H NMR** (400 MHz, $CDCl_3$) δ_H 7.88-7.80 (3H, m, Ts*H* + Ar*H*), 7.40 (1H, d, *J* = 1.5 Hz, Ar*H*), 7.22-7.19 (2H, m, Ts*H*), 7.18-7.15 (1H, m, Ar*H*), 7.09 (ddd, *J* = 7.9, 1.4, 0.8 Hz, 1H), 7.04-7.01 (m, 1H), 6.98 (2H, d, *J* = 8.3 Hz, Ts*H*), 6.80-6.77 (2H, m, Ts*H*), 5.83-5.78 (1H, m, H2), 5.76-5.70 (1H, m, H3), 5.37 (1H, d, *J* = 1.5 Hz, H7), 4.34-4.07 (1H, m, H5), 4.09 (1H, ddd, *J* = 5.9, 4.6, 1.3 Hz, H6), 2.97 (1H, ddd, *J* = 6.2, 4.6, 1.4 Hz, H1), 2.57 (2H, q, *J* = 2.5 Hz, H4), 2.29 (3H, s, TsC*H*$_3$), 2.18 (3H, s, TsC*H*$_3$), 0.87

(9H, s, SiC(CH_3)$_3$), 0.05 (6H, d, J = 8.2 Hz, Si(CH_3)$_2$); **^{13}C NMR** (100 MHz, $CDCl_3$) δ_C 144.9, 144.7, 143.3, 136.3, 135.8, 134.1, 131.2, 129.9, 129.3, 127.3, 126.8, 126.5, 125.8, 125.5, 124.0, 122.6, 120.2, 117.4, 113.2, 95.6, 68.1, 62.0, 48.3, 29.4, 25.8, 21.7, 21.5, 18.1, -4.7, -4.8; **HRMS** (ES$^+$) calc. for $C_{36}H_{43}N_2O_5S_2Si$ [M+H]$^+$ 675.2377, found 675.2370;

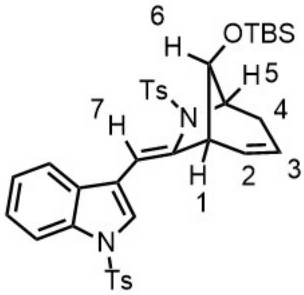

E **isomer**: **R$_f$** 0.285 (pentane/EtOAc 6:1); **IR** (thin film, ν_{max} / cm^{-1}) 2953, 2928, 2856, 2360, 2341, 1653, 1598, 1447, 1373, 1348, 1164, 1092, 982, 914, 873, 837, 744, 668, 624; **^{1}H NMR** (500 MHz, $CDCl_3$) δ_H 7.97 (1H, d, J = 8.2 Hz, ArH), 7.85-7.80 (2H, m, TsH), 7.72 (2H, d, J = 8.4 Hz, TsH), 7.41 (1H, d, J = 7.8 Hz, ArH), 7.34-7.29 (4H, dd, J = 8.3, 2.8 Hz, 2 x TsH + 2 x ArH), 7.24 (1H, d, J = 7.1 Hz, ArH), 7.19 (2H, d, J = 8.1 Hz, TsH), 6.39 (1H, br, H7), 5.78-5.73 (1H, m, H2), 5.71-5.67 (1H, m, H3), 4.28-4.17 (1H, m, H5), 3.87 (1H, ddd, J = 5.8, 4.4, 1.4 Hz, H6), 3.09 (1H, t, J = 5.3 Hz, H1), 2.56-2.48 (2H, m, H4), 2.43 (3H, s, TsCH_3), 2.33 (3H, s, TsCH_3), 0.85 (9H, s, SiC(CH_3)$_3$), -0.01 (6H, d, J = 13.6 Hz, Si(CH_3)$_2$); **^{13}C NMR** (125 MHz, $CDCl_3$) δ_C 145.1, 144.5, 144.3, 136.6, 135.3, 135.0, 131.1, 130.0, 129.8, 127.2, 127.1, 126.9, 125.2, 124.2, 123.5, 120.8, 119.9, 119.8, 113.9, 93.8, 68.3, 59.9, 43.3, 29.3, 25.8, 21.73, 21.71, 18.1, -4.7, -4.8; **HRMS** (ES$^+$) calc. for $C_{36}H_{43}N_2O_5S_2Si$ [M+H]$^+$ 675.2377, found 675.2366.

(±)-3-{[(1*S*,5*R*,7*R*,8*S*)-8-(*Tert*-butyldimethylsilyl)oxy)-6-tosyl-6-azabicyclo[3.2.1]oct-2-en-7-yl]methyl}-1-tosyl-1*H*-indole, 19

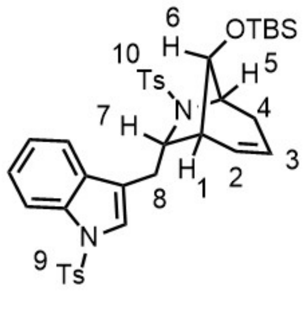

To a stirred solution of ***Z*** and ***E*** isomers **18** (2.20 g, 3.26 mmol, 1.0 equiv.) and $NaCNBH_3$ (1.02 g, 16.3 mmol, 5.0 equiv.) in dry CH_2Cl_2 (33 mL) at room temperature was added trifluoroacetic acid

(1.25 mL, 16.30 mmol, 5.0 equiv.). The reaction mixture was stirred for 10 min, then quenched by addition of saturated $NaHCO_3$ solution. The mixture was extracted with EtOAc (x 2), then the organic fractions were combined, dried (Na_2SO_4), filtered and concentrated. Silica gel flash chromatography (pentane/EtOAc 10:1) gave the title compound **19** as a yellow oil (1.83 g, 2.71 mmol, 83%). **R_f** 0.285 (pentane/EtOAc 6:1); **IR** (thin film, ν_{max} / cm^{-1}) 2954, 2928, 2897, 2855, 2360, 2341, 1598, 1447, 1373, 1347, 1174, 1162, 1089, 976, 912, 837, 814,779 670, 614; **^{1}H NMR** (400 MHz, $CDCl_3$) δ_H 8.02-7.98 (1H, m, Ar*H*), 7.79 (2H, d, *J* = 8.3 Hz, Ts*H*), 7.76-7.71 (3H, m, 2 x Ts*H* + Ar*H*), 7.33 (5H, td, *J* = 7.2, 1.4 Hz, 2 x Ts*H* + 3 x Ar*H*), 7.22-7.16 (2H, m, Ts*H*), 5.87-5.83 (1H, m, H2), 5.62-5.57 (1H, m, H3), 3.83 (1H, ddd, *J* = 11.5, 4.6, 3.5 Hz, H7), 3.74-3.68 (1H, m, H6), 3.60 (1H, ddd, *J* = 14.2, 3.6, 1.3 Hz, H8), 3.20 (1H, dd, *J* = 14.2, 11.4 Hz, H5), 3.10 (1H, ddd, *J* = 5.5, 4.1, 1.3 Hz, H8), 2.46 (1H, d, *J* = 4.1 Hz, H4), 2.43 (3H, s, H10), 2.32 (3H, s, H9), 2.24-2.16 (1H, m, H4), 2.13-2.09 (1H, m, H1), 0.73 (9H, s, $SiC(CH_3)_3$), -0.22 (6H, d, *J* = 15.3 Hz, $Si(CH_3)_2$); **^{13}C NMR** (100 MHz, $CDCl_3$) δ_C 145.0, 144.0, 135.4, 135.3, 134.9, 131.1, 130.0, 128.2, 127.7, 126.9, 125.0, 123.9, 123.54, 123.48, 120.1, 119.9, 114.0, 68.4, 66.1, 59.0, 41.5, 32.1, 28.0, 25.7, 21.7, 21.6, 18.0, -5.05, -5.07; **HRMS** (ES^+) calc. for $C_{36}H_{45}N_2O_5S_2Si$ $[M+H]^+$ 677.2534, found 677.2515.

One-pot procedure:

To a solution of ynamide **17** (150 mg, 0.222 mmol, 1.0 equiv.) in a dry, argon flushed flask, was added $Pd(OAc)_2$ (7.4 mg, 0.033 mmol, 0.15 equiv.) and bbeda (7.8 mg, 0.033 mmol, 0.15 equiv.) and dry toluene (2.2 mL, c = 0.1 M); the mixture was stirred for 1 h at 60 °C. The reaction was then cooled to room temperature, and to this mixture was added $NaCNBH_3$ (70 mg, 1.11 mmol, 5.0 equiv.) and then trifluoroacetic acid (0.09 mL, 1.11 mmol, 5.0 equiv.). The reaction was stirred for 10 min, then quenched with saturated $NaHCO_3$ solution, and extracted twice with EtOAc. The organic fractions were combined, dried (Na_2SO_4), filtered and concentrated. Silica gel flash chromatography (pentane/EtOAc 10:1) gave the title compound **19** as a yellow oil (78 mg, 0.115 mmol, 52%).

(±)-(1*S*,5*R*,7*S*,8*R*)-6-Tosyl-7-[(1-tosyl-1*H*-indol-3-yl)methyl]-6-azabicyclo[3.2.1]oct-2-en-8-ol, S12

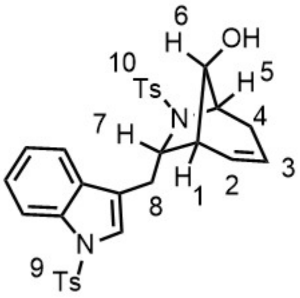

To a solution of **19** (2.03 g, 3.0 mmol, 1.0 equiv.) in MeOH (81 mL, c = 0.04 M), *p*-toluenesulfonic acid monohydrate (2.85 g, 15.0 mmol, 5.0 equiv.) was added at room temperature. The reaction was stirred at 50 °C overnight (12 h), then cooled to room temperature, and quenched with $NaHCO_3$ aq. The organic solvent was removed, and the remnant aqueous layer was extracted with EtOAc. The organic layers were combined and dried over Na_2SO_4, and then concentrated to give the title compound **S12** (1.65 g, 2.94 mmol, 98%) as a colourless oil. The crude product was directly used without further purification. **R_f** 0.34 (DCM/MeOH 30:1); **IR** (thin film, ν_{max} / cm^{-1}) 3052, 2920, 2848, 2360, 1598, 1447, 1371, 1212, 1174, 1122, 1091, 1020, 985, 814, 746, 705, 667; **^{1}H NMR** (400 MHz, $CDCl_3$) δ_H 7.95-7.88 (1H, m, Ar*H*), 7.71 (2H, d, *J* = 8.3 Hz, Ts*H*), 7.65 (2H, d, *J* = 8.4 Hz, Ts*H*), 7.62 (1H, dt, *J* = 7.4, 1.0 Hz, ArH), 7.31-7.21 (5H, m, 2 x Ts*H* + 3 x Ar*H*), 7.15-7.11 (2H, m, Ts*H*), 6.03-5.92 (1H, m, H2), 5.65-5.60 (1H, m, H3), 3.84-3.81 (1H, m, H6), 3.80-3.79 (1H, m, H7) 3.58 (1H, ddd, *J* = 14.3, 3.6, 1.3 Hz, H8), 3.18 (1H, dtd, *J* = 10.0, 4.2, 2.1 Hz, H5), 3.07 (1H, dd, *J* = 14.3, 11.4 Hz, H8), 2.45-2.39 (1H, m, H4), 2.36 (3H, s, H10), 2.32-2.30 (1H, m, H4), 2.26 (3H, s, H9), 2.23-2.22 (1H, m, H1); **^{13}C NMR** (100 MHz, $CDCl_3$) δ_C 145.1, 144.1, 135.3, 135.2, 134.7, 130.9, 130.2, 130.1, 130.0, 127.6, 126.8, 125.1, 124.4, 123.5, 123.4, 119.9, 119.6, 114.0, 68.1, 65.9, 58.1, 41.6, 31.8, 28.2, 21.7(2C); **HRMS** (ES^+) calc. for $C_{30}H_{31}N_2O_5S_2$ $[M+H]^+$ 563.1661, found 563.1669.

(±)-(1*S*,5*R*,7*S*,8*R*)-7-[(1*H*-Indol-3-yl)methyl]-6-azabicyclo[3.2.1]oct-2-en-8-ol, 21

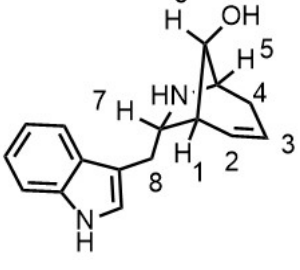

To prepare the sodium naphthalenide solution, freshly cut sodium pieces (825 mg, 75.0 mmol) were

added to a solution of naphthalene (2.31 g, 18.0 mmol) in anhydrous THF (90 mL). The mixture was sonicated at room temperature for 1 h with occasional swirling to afford a dark-green solution of sodium naphthalenide (0.2 M) in THF.

To a solution of **S12** (1.21 g, 2.15 mmol, 1.0 equiv.) in THF (12 mL) at -78 °C was added Na/nap solution (60 mL, 12.0 mmol, 5.58 equiv. 0.2 M) until the reaction solution became dark green. Stirring was continued at the same temperature until consumption of starting material (1 h). Then, the reaction mixture was quenched with NH_4Cl aq.; the aqueous layer was extracted with ether, and the organic layers were combined and dried over Na_2SO_4. Silica gel flash chromatography (DCM/MeOH/Et_3N (15:15:0.1)) gave the title compound **21** as a white foam (411 mg, 1.62 mmol, 75%). **R_f** 0.22 (DCM/MeOH/Et_3N (15:15:0.1); **IR** (thin film, ν_{max} / cm^{-1}) 3411, 3309, 2926, 2854, 2360, 2341, 1636, 1619, 1456, 1354, 1340, 1263, 1122, 1101, 1010, 812, 741, 689; **^{1}H NMR** (400 MHz, $CDCl_3$) δ_H 8.12 (1H, br, N*H*), 7.59 (1H, d, *J* = 7.9 Hz, Ar*H*), 7.34 (1H, d, *J* = 8.1 Hz, Ar*H*), 7.19 (1H, t, *J* = 7.5 Hz, Ar*H*), 7.11 (1H, t, *J* = 7.5 Hz, Ar*H*), 7.00 (1H, s, Ar*H*), 5.99 (1H, d, *J* = 10.0 Hz, H2), 5.80-5.68 (1H, m, H3), 4.31 (1H, t, *J* = 4.9 Hz, H6), 3.90-3.85 (1H, m, H7), 3.27 (1H, d, *J* = 5.4 Hz, H5), 2.92 (2H, d, *J* = 7.4 Hz, H8), 2.61-2.50 (1H, m, H4), 2.44 (1H, br, H1), 2.04-1.96 (1H, m, H4); **^{13}C NMR** (100 MHz, $CDCl_3$) δ_C 136.4, 128.9, 127.7, 125.7, 122.2, 121.8, 119.4, 119.0, 113.7, 111.3, 72.1, 64.6, 54.5, 43.1, 34.7, 28.6; **HRMS** (ES^+) calc. for $C_{16}H_{19}N_2O$ $[M+H]^+$ 255.1492, found 255.1492.

(±)-(1*S*,5*R*,7*S*,8*R*)-7-[(1*H*-Indol-3-yl)methyl]-6-isopropyl-6-azabicyclo[3.2.1]oct-2-en-8-ol, 22

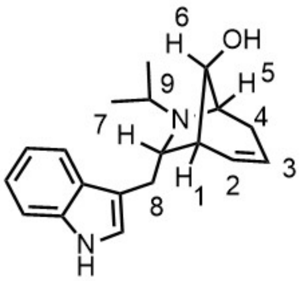

To a solution of amino alcohol **21** (52 mg, 0.205 mmol, 1.0 equiv.) in MeCN (5.0 mL) was added acetone (119 mg, 2.05 mmol, 10.0 equiv.) and $NaBH(OAc)_3$ (127 mg, 0.615 mmol, 3.0 equiv.). The reaction was stirred at 50 °C until consumption of the substrate (as confirmed by TLC). Then, the reaction mixture was quenched with *sat.* NH_4Cl *aq*. The residual aqueous layer was extracted with ether, then the organic layers were combined and dried over Na_2SO_4, and concentrated to afford **22** as a yellow oil which was used without further purification (59 mg, 0.20 mol, 97%); **R_f** 0.3

(DCM/MeOH/Et_3N (15:15:0.1); **IR** (thin film, ν_{max} / cm^{-1}) 3242, 2955, 2924, 2362, 2339, 1568, 1401, 1359, 1221, 1127, 911, 735, 649; **^{1}H NMR** (400 MHz, $CDCl_3$) δ_H 8.00 (1H, br, N*H*), 7.59 (1H, dt, *J* = 7.9, 1.0 Hz, Ar*H*), 7.36 (1H, dt, *J* = 8.2, 1.0 Hz, Ar*H*), 7.20 (1H, ddd, *J* = 8.2, 7.0, 1.3 Hz, Ar*H*), 7.12 (1H, ddd, *J* = 8.0, 7.0, 1.1 Hz, Ar*H*), 6.99 (1H, d, *J* = 2.3 Hz, Ar*H*), 5.99-5.95 (1H, m, H2), 5.80-5.70 (1H, m, H3), 3.97 (1H, ddd, *J* = 5.6, 4.0, 1.3 Hz, H6), 3.49 (1H, dt, *J* = 9.4, 4.7 Hz, H7), 3.17-3.11 (1H, m, H5), 3.11-3.04 (1H, m, H9), 2.98-2.87 (2H, m, H8), 2.47-2.44 (1H, m, H1), 2.44-2.41 (1H, m, H4), 2.00-1.91 (1H, m, H4), 1.16 (3H, d, *J* = 6.7 Hz, CH(C*H*$_3$)), 1.05 (3H, d, *J* = 6.3 Hz, CH(C*H*$_3$)); **^{13}C NMR** (100 MHz, $CDCl_3$) δ_C 136.3, 130.0, 127.8, 126.1, 122.1, 121.7, 119.4, 119.1, 114.4, 111.2, 70.5, 66.7, 53.5, 49.5, 43.0, 34.1, 27.5, 23.9, 15.9; **HRMS** (ES^+) calc. for $C_{19}H_{25}N_2O$ $[M+H]^+$ 297.1956, found 297.1961.

(±)-(1*S*,5*R*,7*S*)-7-[(1*H*-Indol-3-yl)methyl]-6-isopropyl-6-azabicyclo[3.2.1]oct-2-en-8-one, S9

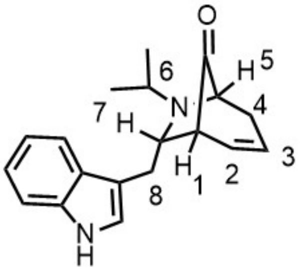

To a solution of **22** (30.0 mg, 0.10 mmol, 1.0 equiv.) in ethyl acetate (2.0 mL) was added IBX (140 mg, 0.50 mmol, 5.0 equiv.), and the reaction was stirred at 85 °C overnight. Then, the reaction mixture was cooled to room temperature, filtered through celite, and concentrated. Silica gel flash chromatography (DCM/MeOH 10:1 to 5:1) gave the title compound **S9** as a yellow oil (28.5 mg, 0.097 mmol, 97%); **R_f** 0.2 (DCM/MeOH 10:1); **IR** (thin film, ν_{max} / cm^{-1}) 3378, 2829, 2870, 2360, 2341, 1781, 1584, 1458, 1429, 1389, 1259, 1235, 1177, 1163, 1098, 1014, 911, 741, 625; **^{1}H NMR** (500 MHz, $CDCl_3$) δ_H 7.99 (1H, br, N*H*), 7.59 (1H, d, *J* = 7.9 Hz, Ar*H*), 7.37 (1H, d, *J* = 8.1 Hz, Ar*H*), 7.23-7.17 (1H, m, Ar*H*), 7.17-7.08 (1H, m, Ar*H*), 7.00 (1H, br, Ar*H*), 5.81-5.74 (2H, m, H2 + H3), 3.57 (1H, dt, *J* = 9.5, 4.6 Hz, H7), 3.26-3.03 (4H, m, H5 + H6 + H8), 2.85-2.75 (1H, m, H4), 2.64-2.58 (1H, m, H4), 2.56-2.51 (1H, m, H1), 1.15 (3H, d, *J* = 6.7 Hz, CH(C*H*$_3$), 0.97 (3H, d, *J* = 6.4 Hz, CH(C*H*$_3$); **^{13}C NMR** (125 MHz, $CDCl_3$) δ_C 212.2, 136.3, 128.9, 127.9, 127.6, 122.3, 121.7, 119.6, 119.0, 113.4, 111.3, 65.4, 58.9, 50.4, 48.7, 44.2, 27.4, 23.7, 15.9; **HRMS** (ES^+) calc. for $C_{19}H_{23}N_2O$ $[M+H]^+$ 295.1805, found 295.1804.

(±)-(1*S*,5*R*,7*S*,8*R*)-7-[(1*H*-Indol-3-yl)methyl]-6-isopropyl-8-methyl-6-azabicyclo[3.2.1]oct -2-en-8-ol, S39

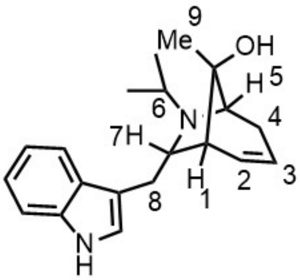

To a solution of **S9** (12 mg, 0.04 mmol, 1.0 equiv.) in dry THF (2.0 mL) at -78 °C was added MeLi (1.0 M in THF, 0.12 mL, 0.12 mmol, 3.0 equiv.), then the mixture was allowed to warm up to room temperature and stirred for 1 h. The mixture was quenched by the addition of *sat.* NH_4Cl *aq*. The contents were then extracted with Et_2O, washed with brine, dried over $MgSO_4$ and concentrated. Silica gel flash chromatography (DCM/MeOH 10:1) gave the title compound **S39** (12 mg, 0.039 mmol, 97%) as a yellow oil; **R_f** 0.28 (DCM/MeOH 10:1); **IR** (thin film, ν_{max} / cm^{-1}) 3412, 2967, 2921, 2359, 2342, 1671, 1457, 1389, 1381, 1198, 1173, 1093, 1011, 962, 911, 739, 646; **^{1}H NMR** (500 MHz, $CDCl_3$) δ_H 7.96 (1H, s, N*H*), 7.60 (1H, d, *J* = 7.9 Hz, Ar*H*), 7.37 (1H, d, *J* = 8.1 Hz, Ar*H*), 7.22-7.18 (1H, m, Ar*H*), 7.15-7.11 (1H, m, Ar*H*), 7.00 (1H, d, *J* = 2.3 Hz, Ar*H*), 6.07-5.97 (1H, m, H2), 5.81 (1H, ddt, *J* = 9.8, 6.4, 2.0 Hz, H3), 3.56 (1H, dt, *J* = 10.7, 4.0 Hz, H7), 3.19 (1H, q, *J* = 6.5 Hz, H6), 3.01 (1H, dd, *J* = 14.3, 3.7 Hz, H8), 2.93-2.90 (1H, m, H5), 2.85 (1H, dd, *J* = 14.4, 10.5 Hz, H8), 2.51-2.47 (1H, m, H4), 2.11-2.09 (1H, m, H1), 2.07-2.02 (1H, m, H4), 1.28 (3H, s, H9), 1.21 (3H, d, *J* = 6.7 Hz, CH(C*H*$_3$), 1.05 (3H, d, *J* = 6.3 Hz, CH(C*H*$_3$); **^{13}C NMR** (125 MHz, $CDCl_3$) δ_C 136.3, 130.5, 128.1, 127.9, 122.1, 121.6, 119.4, 119.2, 114.5, 111.2, 73.4, 66.3, 60.1, 49.2, 48.6, 37.1, 28.3, 24.3, 23.9, 19.5; **HRMS** (ES$^+$) calc. for $C_{20}H_{27}N_2O$ [M+H]$^+$ 311.2118, found 311.2111. The stereochemistry of the product was assigned by analogy to reduction of a bridge ketone, see: (M. Kitamura, Y. Ihara, K. Uera, K. Narasaka, *Bull. Chem. Soc. Jpn.* **2006**, *79*, 1552-1560).

(±)-3-{[(1*S*,5*R*,7*S*)-6-Isopropyl-8-methylene-6-azabicyclo[3.2.1]oct-2-en-7-yl]methyl}-1*H*-indole, 20

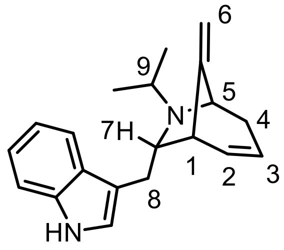

Dimethyl sulfide (0.11 mL, 1.5 mmol, 15.0 equiv.) was added to a cold (0 °C) solution of *N*-chlorosuccinimide (134 mg, 1.0 mmol, 10.0 equiv.) in toluene/dichloromethane mixture (2.0 mL total volume; 2:1) under an argon atmosphere. After 5 minutes, the resulting suspension was cooled to -78 °C and stirred for additional 10 minutes. A solution of alcohol **22** (30 mg, 0.1 mmol, 1.0 equiv.) in toluene/dichloromethane mixture (2:1, 3.0 mL total volume) was added dropwise and the reaction mixture was allowed to reach -50 °C, stirring for 30 minutes. Triethylamine (0.21 mL, 1.5 mmol, 15.0 equiv.) was added and the reaction mixture was stirred 15 minutes at -50 °C. Isopropanol (1.0 mL) was added to quench the reaction, and the mixture was partitioned between ethyl acetate and brine. The organic extract was dried over anhydrous Na_2SO_4, filtered and concentrated. The residue was taken directly to the next step directly.

Preparation of fresh Tebbe reagent[21]: to a nitrogen-flushed vial contained Cp_2TiCl_2 (500 mg, 2.0 mmol, 1.0 equiv.) was added $AlMe_3$ (2.0 M in toluene, 2.2 mL, 4.4 mmol, 2.2 equiv.) at room temperature; this mixture was stirred for 72 hours before use (note: a white gas evolves while adding $AlMe_3$ solution).

To a cooled (-45 °C) solution of above crude ketone **S9** in THF (1.5 mL) was added freshly prepared Tebbe reagent (3 days old, 1.0 M in toluene, 0.45 mL, 0.4 mmol, 4.0 equiv.). The reaction mixture was maintained at -45 °C for 30 min, and then slowly warmed to 0 °C and stirred for 2.5 h. The reaction mixture was then warmed to 23 °C and stirred for 3 h. The solution was diluted with THF (1.0 mL) and treated with 15% aq. NaOH (0.2 mL). After being stirred for 1 h, the heterogeneous mixture was filtered through celite, and the precipitate was washed with Et_2O. The filtrates were combined and concentrated in vacuo. Purification by flash chromatography (pentane/EtOAc 2:1) provided **20** (9 mg, 30.8 μmol, 31%, over 2 steps from **22**) as a white oil; $\mathbf{R_f}$ 0.21 (Pentane/EtOAc 1:1); **IR** (thin film, ν_{max} / cm^{-1}) 3420, 3027, 2962, 2927, 1707, 1682, 1619, 1493, 1382, 1360, 1290, 1219, 1092, 910, 884, 740; **^{1}H NMR** (400 MHz, $CDCl_3$) δ_H 7.98 (1H, br, N*H*), 7.61 (1H, dd, *J* = 7.9, 1.0 Hz, Ar*H*), 7.37 (1H, dt, *J* = 8.1, 0.9 Hz, Ar*H*), 7.22-7.17 (1H, m, Ar*H*), 7.14-7.10 (1H, m, Ar*H*), 7.03 (1H, d, *J* = 2.3 Hz, Ar*H*), 5.92-5.87 (1H, m, H2), 5.71-5.67 (1H, m, H3), 4.55 (2H, d, *J* = 10.1 Hz, H6), 3.50 (1H, s, H5), 3.35 (1H, td, *J* = 7.2, 4.3 Hz, H7), 3.14-3.06 (1H, m, H9), 3.01 (2H, d, *J* = 7.3 Hz, H8), 2.66-2.63 (1H, m, H1), 2.52-2.39 (1H, m, H4), 2.31-2.24 (1H, m, H4), 1.19 (3H, d, *J* = 6.7 Hz, $CHCH_3$), 1.00 (3H, d, *J* = 6.3 Hz, $CHCH_3$); **^{13}C NMR** (100 MHz, $CDCl_3$) δ_C 151.8, 136.3,

130.3, 127.9, 127.3, 122.1, 121.7, 119.4, 119.2, 114.6, 111.2, 98.0, 70.1, 58.5, 50.6, 45.0, 42.7, 27.5, 23.8, 16.1; **HRMS** (ES^+) calc. for $C_{20}H_{25}N_2$ $[M+H]^+$ 293.2012, found 293.2010.

(±)-*N*-[(1*R*,5*R*,6*S*)-6-[(*Tert*-butyldimethylsilyl)oxy]-5-[2-(1-tosyl-1*H*-indol-3-yl)acetyl] cyclohex -3-en-1-yl]-4-methylbenzenesulfonamide, 23

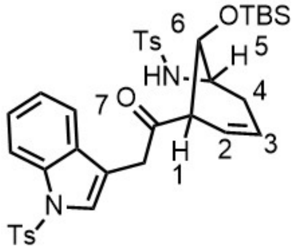

To a solution of an 8:1 *Z*/*E* mixture of **18** (34 mg, 0.05 mmol, 1.0 equiv.) in toluene (1.5 mL) at room temperature was added TFA (23 mg, 0.2 mmol, 4.0 equiv.) and H_2O (9 mg, 0.5 mmol, 10.0 equiv.). The reaction mixture was stirred for 30 min, then the reaction was quenched with $NaHCO_3$ solution, and extracted twice with ether. The combined organic fractions were dried (Na_2SO_4), filtered and concentrated. Silica gel flash chromatography (pentane/EtOAc 6:1) gave the title compound **23** as a colorless oil (33.4 mg, 0.048 mmol, 97%). $\mathbf{R_f}$ 0.28 (pentane/EtOAc 6:1); **IR** (thin film, ν_{max} / cm^{-1}) 2981, 2954, 2952, 2360, 2341, 1716, 1704, 1447, 1370, 1338, 1187, 1173, 1120, 1094, 913, 838, 743, 668; **^{1}H NMR** (500 MHz, $CDCl_3$) δ_H 7.99 (1H, dt, *J* = 8.4, 0.9 Hz, Ar*H*), 7.77 (2H, d, *J* = 8.4 Hz, Ts*H*), 7.72 (2H, d, *J* = 8.3 Hz, Ts*H*), 7.59 (1H, s, Ar*H*), 7.41-7.38 (1H, m, Ar*H*), 7.33 (1H, ddd, *J* = 8.4, 7.3, 1.2 Hz, Ar*H*), 7.25 (2H, br, Ts*H*), 7.23 (2H, d, *J* = 5.0 Hz, Ts*H*), 7.21 (1H, s, Ar*H*), 5.76-5.69 (1H, m, H2), 5.53 (1H, d, *J* = 6.2 Hz, H3), 5.50 (1H, br, N*H*), 3.99 (1H, dd, *J* = 5.6, 2.9 Hz, H6), 3.88 (2H, br, H7), 3.30 (1H, q, *J* = 2.7 Hz, H1), 3.26 (1H, t, *J* = 5.5 Hz, H5), 2.45-2.41 (1H, m, H4), 2.39 (3H, s, TsCH_3), 2.32 (3H, s, TsCH_3), 2.06-1.98 (1H, m, H4), 0.76 (9H, s, $SiC(CH_3)_3$), -0.07– -0.22 (6H, m, $Si(CH_3)_2$); **^{13}C NMR** (125 MHz, $CDCl_3$) δ_C 207.2, 145.2, 143.5, 137.7, 135.3, 135.2, 130.5, 130.1, 129.8, 127.3, 127.0, 126.7, 125.3, 125.2, 123.6, 121.2, 119.5, 114.3, 113.9, 67.9, 56.1, 51.7, 38.9, 27.8, 25.7, 21.7, 21.6, 17.9, -4.86, -4.95; **HRMS** (ES^+) calc. for $C_{36}H_{45}N_2O_6S_2Si$ $[M+H]^+$ 693.2483, found 693.2468.

Larger scale procedure:

To a solution of 8:1 *Z*/*E* mixture of **18** (698 mg, 1.03 mmol, 1.0 equiv.) in dry toluene (10.0 mL) was added TFA (470 mg, 4.12 mmol, 4.0 equiv.) and H_2O (371 mg, 20.6 mmol, 20.0 equiv.) at room

temperature; the mixture was stirred for 30 min. The reaction was then quenched by addition of $NaHCO_3$ solution, then was extracted twice with ether. The organic fractions were combined, dried (Na_2SO_4), filtered and concentrated. Silica gel flash chromatography (pentane/EtOAc 6:1 to 3:1) gave the title compound **23** as a colorless oil (557 mg, 0.823 mmol, 80%).

(±)-*N*-((1*R*,5*S*,6*R*)-6-((*Tert*-butyldimethylsilyl)oxy)-5-((*S*)-1-hydroxy-2-(1-tosyl-1*H*-indol-3-yl)ethyl)cyclohex-3-en-1-yl)-4-methylbenzenesulfonamide, 24a

(±)-*N*-((1*S*,5*R*,6*S*)-6-((*Tert*-butyldimethylsilyl)oxy)-5-((*S*)-1-hydroxy-2-(1-tosyl-1*H*-indol-3-yl)ethyl)cyclohex-3-en-1-yl)-4-methylbenzenesulfonamide, 24

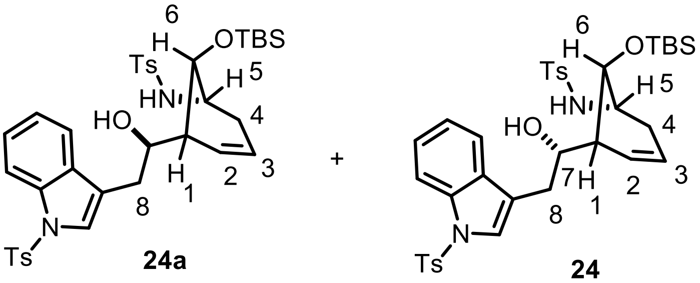

24a **24**

To a stirred solution of ketone **23** (1.27 g, 1.84 mmol, 1.0 equiv.) in dry methanol (9.2 mL) at 0 °C was added $NaBH_4$ (84 mg, 2.21 mmol, 1.2 equiv.). The reaction was stirred for 30 min, then quenched with saturated aqueous NH_4Cl (1.0 mL) and evaporated to dryness. The mixture was diluted with water (1.0 mL) and EtOAc (20 mL), the organic layer was separated and the aqueous layer was extracted with EtOAc (3×10 mL). The combined organic layers were washed with brine, dried over anhydrous Na_2SO_4 and concentrated in vacuo. The residue was purified by flash chromatography on silica gel (gradient, pentane/EtOAc = 6:1 to 3:1) to give the **24a** (355 mg, 0.512 mmol, 28%) and **24** (858 mg, 1.236 mmol, 70%).

Data for diastereomer **24a**, **^{1}H NMR** (400 MHz, $CDCl_3$) δ_H 8.02 (1H, dt, *J* = 8.2, 0.9 Hz, Ar*H*), 7.80-7.74 (4H, m, Ts*H*), 7.51-7.45 (2H, m, Ar*H*), 7.35 (1H, ddd, *J* = 8.3, 7.2, 1.3 Hz, Ar*H*), 7.30-7.26 (3H, m, 2 x Ts*H* + Ar*H*), 7.25-7.22 (2H, m, Ts*H*), 6.63 (1H, d, *J* = 8.4 Hz, N*H*), 5.72 (1H, ddd, *J* = 10.5, 5.3, 2.6 Hz, H2), 5.59-5.49 (1H, m, H3), 4.02 (1H, dt, *J* = 9.6, 3.7 Hz, H7), 3.98 (1H, d, *J* = 4.0 Hz, H6), 3.42-3.36 (1H, m, H5), 2.89-2.70 (2H, m, H8), 2.46 (1H, ddt, *J* = 15.4, 5.8, 3.1 Hz, H4), 2.41 (3H, s, TsC*H*$_3$), 2.37 (1H, dd, *J* = 3.5, 0.9 Hz, O*H*), 2.34 (3H, s, TsC*H*$_3$), 2.27 (1H, t, *J* = 2.7 Hz, H1), 1.96 (1H, ddd, *J* = 17.6, 6.4, 2.3 Hz, H4), 0.82 (9H, d, *J* = 4.8 Hz, SiC(C*H*$_3$)$_3$), -0.04 (6H, d, *J* = 12.7 Hz, Si(C*H*$_3$)$_2$); **^{13}C NMR** (100 MHz, $CDCl_3$) δ_C 145.2, 143.3, 138.6, 135.4, 135.3, 130.8, 130.1, 129.8,

127.2, 126.9, 126.2, 125.7, 125.2, 124.2, 123.4, 119.6, 119.1, 114.0, 72.1, 67.6, 50.2, 49.0, 30.8, 27.2, 25.8, 21.7, 21.6, 17.9, -4.5, -4.6; **HRMS** (ES^+) calc. for $C_{36}H_{47}N_2O_6S_2Si$ $[M+H]^+$ 695.2639, found 695.2627.

Data for **24**; **^{1}H NMR** (500 MHz, $CDCl_3$) δ_H 8.04-7.94 (m, 1H), 7.79-7.74 (m, 2H), 7.73-7.68 (m, 2H), 7.47-7.41 (m, 2H), 7.34 (ddd, *J* = 8.4, 7.2, 1.3 Hz, 1H), 7.26-7.20 (m, 5H), 6.69 (d, *J* = 8.4 Hz, 1H), 5.82-5.77 (m, 1H), 5.75-5.69 (m, 1H), 3.97-3.92 (m, 1H), 3.51 (dd, *J* = 5.0, 2.4 Hz, 1H), 3.29 (dq, *J* = 8.7, 4.6 Hz, 1H), 2.99 (dd, *J* = 14.3, 8.1 Hz, 1H), 2.90 (dd, *J* = 14.3, 5.8 Hz, 1H), 2.43 (dd, *J* = 5.3, 2.8 Hz, 1H), 2.33 (s, 3H), 2.13 (s, 1H), 1.96 (dt, *J* = 17.8, 4.3 Hz, 2H), 0.69 (s, 9H), -0.15 (s, 3H), -0.28 (s, 3H); **^{13}C NMR** (125 MHz, CDCl3) δ_C 145.2, 143.2, 138.6, 135.5, 135.3, 130.5, 130.1, 129.8, 127.4, 127.2, 126.9, 125.2, 124.5, 123.4, 121.7, 119.5, 118.7, 114.1, 73.0, 72.0, 51.2, 47.7, 31.9, 28.0, 25.7, 21.7, 21.6, 17.9, -4.6, -5.1; **HRMS** (ES^+) calc. for $C_{36}H_{47}N_2O_6S_2Si$ $[M+H]^+$ 695.2639, found 695.2627.

(±)-3-{[(1*R*,5*R*,7*R*,8*R*)-8-[(*Tert*-butyldimethylsilyl)oxy]-6-tosyl-6-azabicyclo[3.2.1]oct-2-en-7-yl]methyl}-1-tosyl-1*H*-indole, 25

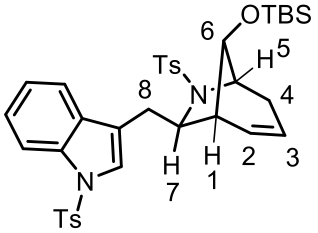

A heat gun-dried RBF was equipped with a magnetic stir bar and PPh_3 (240 mg, 0.914 mmol, 1.5 equiv.) was added a solution of substrate **24** (423 mg, 0.61 mmol, 1.0 equiv.) in THF (2.0 mL), then it was cooled down to 0 °C stirring for 5 min. then DIAD (0.18 mL, 0.914 mmol, 1.5 equiv.) was added, then the mixture was allowed to warm up to room temperature stirring for 1 h. The reaction mixture was concentrated under reduced pressure then silica gel column chromatography (gradient, pentane/EtOAc 15:1 to 10:1) afforded **25** (392 mg, 0.58 mmol, 95%) as a yellow oil; **R$_f$** 0.27 (pentane/EtOAc 6:1); **IR** (thin film, ν_{max} / cm^{-1}) 2953, 2933, 2857, 2362, 2341, 1599, 1448, 1373, 1355, 1175, 1159, 1121, 979, 890, 874, 779, 746, 670; **^{1}H NMR** (500 MHz, $CDCl_3$) δ_H 8.03 (1H, d, *J* = 8.3 Hz, Ar*H*), 7.80 (2H, d, *J* = 8.3 Hz, Ts*H*), 7.77 (2H, d, *J* = 8.4 Hz, Ts*H*), 7.70 (1H, dd, *J* = 7.7, 1.2 Hz, Ar*H*), 7.37 (1H, ddd, *J* = 8.4, 7.2, 1.3 Hz, Ar*H*), 7.34-7.29 (4H, m, 2 x Ts*H* + 2 x Ar*H*), 7.23 (2H, d, *J* = 8.4 Hz, Ts*H*), 5.30 (1H, ddd, *J* = 9.6, 6.2, 1.6 Hz, H2), 5.22 (1H, ddt, *J* = 8.4, 3.4, 1.6 Hz, H3), 4.38 (1H, ddd, *J* = 5.6, 4.0, 1.4 Hz, H6), 4.00 (1H, d, *J* = 3.1 Hz, H7), 3.98 (1H, d, *J* = 3.4 Hz,

H5), 3.47 (1H, ddd, *J* = 14.3, 3.2, 1.2 Hz, H8), 2.78 (1H, dd, *J* = 14.3, 10.8 Hz, H8), 2.44 (3H, s, TsC*H*3), 2.36 (3H, s, TsC*H*3), 2.23 (3H, td, *J* = 5.6, 3.1 Hz, 2 x H4 + H1), 0.86 (9H, s, SiC(C*H*3)3), 0.02 (6H, d, *J* = 4.6 Hz, Si(C*H*3)2); **^{13}C NMR** (125 MHz, $CDCl_3$) δ_C 145.1, 143.3, 138.8, 135.4, 135.3, 130.9, 130.0, 129.6, 127.4, 126.9, 126.3, 126.3, 125.1, 124.0, 123.6, 120.0, 119.9, 114.0, 68.2, 68.0, 58.7, 42.7, 31.4, 28.2, 25.7, 21.69, 21.67, 18.1, -4.8, -4.9; **HRMS** (ES^+) calc. for $C_{36}H_{45}N_2O_5S_2Si$ $[M+H]^+$ 677.2534, found 677.2521.

(±)-3-(((1*S*,5*R*,7*S*,8*R*)-8-((*Tert*-butyldimethylsilyl)oxy)-6-tosyl-6-azabicyclo[3.2.1]oct-2-en-7-yl)methyl)-1-tosyl-1*H*-indole, 19

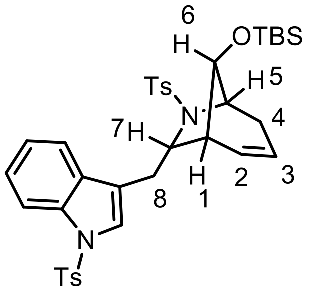

To a flamed-dried RBF was equipped with a magnetic stir bar and PPh_3 (8 mg, 0.03 mmol, 1.5 equiv.) was added a solution of substrate **24a** (14 mg, 0.02 mmol, 1.0 equiv.) in THF (2.0 mL), then it was cooled down to 0 °C stirring for 5 min. then DIAD (6 mg, 0.03 mmol, 1.5 equiv.) was added, then the mixture was allowed to warm up to room temperature stirring for 1 h. The reaction mixture was concentrated under reduced pressure then silica gel column chromatography (gradient, pentane/EtOAc = 15:1 to 10:1) afforded **19** (8 mg, 0.012 mmol, 59%) as a yellow oil; **^{1}H NMR** (400 MHz, $CDCl_3$) δ_H 8.03-7.96 (m, 1H), 7.79 (d, *J* = 8.3 Hz, 2H), 7.77-7.69 (m, 3H), 7.33 (td, *J* = 7.2, 1.5 Hz, 5H), 7.21-7.17 (m, 2H), 6.01-5.77 (m, 1H), 5.71-5.52 (m, 1H), 3.93-3.79 (m, 1H), 3.76-3.68 (m, 1H), 3.60 (ddd, *J* = 14.2, 3.6, 1.3 Hz, 1H), 3.20 (dd, *J* = 14.2, 11.4 Hz, 1H), 3.10 (ddd, *J* = 5.6, 4.1, 1.2 Hz, 1H), 2.46 (q, *J* = 3.0 Hz, 1H), 2.43 (s, 3H), 2.32 (s, 3H), 2.20 (ddt, *J* = 18.0, 3.9, 2.1 Hz, 1H), 2.11 (ddt, *J* = 5.8, 4.4, 2.5 Hz, 1H), 0.74 (s, 9H), -0.22 (d, *J* = 15.3 Hz, 6H); **^{13}C NMR** (100 MHz, $CDCl_3$) δ_C 145.0, 144.0, 135.4, 135.3, 134.9, 131.1, 130.0, 128.2, 127.7, 126.9, 125.0, 123.9, 123.54, 123.48, 120.1, 119.9, 114.0, 68.4, 66.1, 59.0, 41.5, 32.1, 28.0, 25.7, 21.7, 21.6, 18.0, -5.0, -5.1; **HRMS** (ES^+) calc. for $C_{36}H_{45}N_2O_5S_2Si$ $[M+H]^+$ 677.2534, found 677.2521. Data identical to previously made through enamine reduction**.**

(±)-(1*S*,5*R*,7*R*,8*R*)-6-Tosyl-7-((1-tosyl-1*H*-indol-3-yl)methyl)-6-azabicyclo[3.2.1]oct-2-en-8-ol, 26

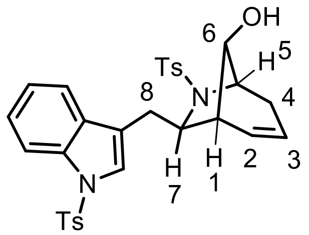

To a stirred solution of **25** (284 mg, 0.42 mmol, 1.0 equiv.) in THF (2.1 mL) at 0 °C was added TBAF (1.0 M in THF, 0.84 mL, 0.84 mmol, 2.0 equiv.). The reaction mixture was allowed to warm to room temperature and stirred for 1 hours, monitored by TLC until disappearance of the starting material. It was then quenched by with water and the aqueous phase extracted with EtOAc. The combined organic extracts were washed with brine, dried over Na_2SO_4 and concentrated. The residue was purified by flash column chromatography (pentane/EtOAc 2:1) to afford **26** (234 mg, 0.416 mmol, 99%) as a white oil; **R$_f$** 0.26 (pentane/EtOAc 2:1); **IR** (thin film, ν_{max} / cm^{-1}) 3527, 2955, 2927, 1598, 1448, 1371, 1333, 1233, 1174, 1158, 1120, 1107, 1050, 1017, 814, 745, 672, 642; **^{1}H NMR** (400 MHz, $CDCl_3$) δ_H 7.99 (1H, d, *J* = 8.1 Hz, Ar*H*), 7.76 (4H, dd, *J* = 14.1, 8.4 Hz, Ts*H*), 7.70 (1H, d, *J* = 7.9 Hz, Ar*H*), 7.37-7.25 (5H, m, 4 x Ts*H* + 1 x Ar*H*), 7.21 (2H, d, *J* = 8.2 Hz, Ar*H*), 5.49 (1H, ddd, *J* = 7.6, 3.7, 2.0 Hz, H2), 5.45-5.39 (1H, m, H3), 4.47 (1H, br, H6), 4.07 (1H, d, *J* = 3.0 Hz, H7), 4.04-4.01 (1H, m, H5), 3.44 (1H, ddd, *J* = 14.1, 3.1, 1.2 Hz, H8), 2.67 (1H, 1H, dd, *J* = 14.2, 11.3 Hz, H8), 2.53-2.45 (1H, m, H4), 2.45-2.43 (1H, m, H1), 2.42 (3H, s, TsC*H*$_3$), 2.33 (3H, s, TsC*H*$_3$), 2.28-2.19 (1H, m, H4); **^{13}C NMR** (100 MHz, $CDCl_3$) δ_C 145.1, 143.5, 138.8, 135.4, 135.3, 130.8, 130.0, 129.8, 128.2, 127.2, 126.9, 126.4, 125.1, 124.0, 123.7, 119.9, 119.6, 113.9, 67.9, 67.4, 57.7, 42.4, 31.1, 28.1, 21.69, 21.66; **HRMS** (ES$^+$) calc. for $C_{30}H_{30}N_2O_5S_2Na$ [M+Na]$^+$ 585.1488, found 585.1494.

(±)-{(1*S*,5*R*,7*S*,8*R*)-8-[(*Tert*-butyldimethylsilyl)oxy]-6-tosyl-6-azabicyclo[3.2.1]oct-2-en-7-yl}(1-tosyl-1*H*-indol-3-yl)methanol, S17

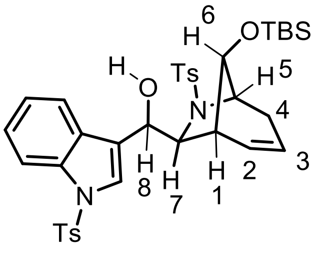

To a heatgun-dried vial equipped with a magnetic stir bar and SeO_2 (3.3 mg, 0.03 mmol, 5.0 equiv.)

was added a solution of substrate **25** (4.0 mg, 5.9 μmol, 1.0 equiv.) in 1,4-dioxane (2.0 mL), then it was heated up to 110 °C stirring overnight. Then it was cooled to rt, to this mixture was added SeO_2 (3.3 mg, 0.03 mmol, 5.0 equiv.) then it was heated up to 110 °C stirring overnight again. Then it was cooled to rt, quenched with 1 mL $NaHCO_3$. The layers were separated, and the aqueous layer was extracted with EtOAc. The combined organic layer was washed with brine, dried over anhydrous Na_2SO_4, and concentrated under reduced pressure. Silica gel column chromatography (pentane/EtOAc 6:1) afforded **S17** (3.0 mg, 4.3 μmol, 71%) as a yellow oil; $\mathbf{R_f}$ 0.16 (pentane/EtOAc 10:1); **IR** (thin film, ν_{max} / cm^{-1}) 3548, 2955, 2929, 2857, 1679, 1598, 1448, 1375, 1176, 1158, 1018, 876, 839, 814, 779, 749, 677; **^{1}H NMR** (400 MHz, $CDCl_3$) δ_H 8.01 (1H, dt, *J* = 8.4, 0.9 Hz, Ar*H*), 7.80 (2H, d, *J* = 8.3 Hz, Ts*H*), 7.76 (2H, d, *J* = 8.4 Hz, Ts*H*), 7.57 (2H, td, *J* = 4.3, 1.1 Hz, Ar*H*), 7.35 (1H, dt, *J* = 8.4, 1.5 Hz, Ar*H*), 7.32-7.29 (2H, m, Ts*H*), 7.25-7.19 (3H, m, 2 x Ts*H* + Ar*H*), 5.53 (1H, ddd, *J* = 4.0, 2.5, 1.2 Hz, H8), 5.24-5.12 (2H, m, H2 + H3), 4.80 (1H, t, *J* = 4.8 Hz, H6), 4.01 (1H, d, *J* = 5.7 Hz, H5), 3.94 (1H, d, *J* = 2.4 Hz, H7), 2.47 (1H, d, *J* = 4.5 Hz, O*H*), 2.44 (3H, s, TsCH_3), 2.33 (3H, s, TsCH_3), 2.31 (1H, d, *J* = 5.6 Hz, H1), 2.29-2.25 (2H, m, H4), 0.83 (9H, s, SiC(CH_3)$_3$), 0.01 (6H, d, *J* = 5.8 Hz, Si(CH_3)$_2$); **^{13}C NMR** (100 MHz, $CDCl_3$) δ_C 145.2, 143.7, 138.0, 135.5, 135.3, 130.5, 130.0, 129.9, 128.7, 127.4, 126.9, 126.4, 125.2, 123.69, 123.67, 123.3, 120.2, 114.1, 72.3, 69.2, 68.3, 58.8, 40.3, 28.5, 25.8, 21.7, 18.1, -4.7, -4.9; **HRMS** (ES$^+$) calc. for $C_{36}H_{44}N_2O_6S_2SiNa$ $[M+Na]^+$ 715.2302, found 715.2293.

(±)-(1*R*,3*R*,5*R*,7*R*,8*R*)-8-[(*Tert*-butyldimethylsilyl)oxy]-6-tosyl-7-[(1-tosyl-1*H*-indol-3-yl) methyl]-6-azabicyclo[3.2.1]octan-3-ol, 27

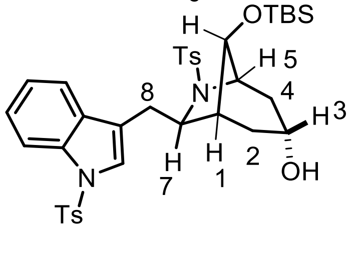

To a stirred solution of **25** (121 mg, 0.179 mmol, 1.0 equiv.) in THF (2.5 mL) at 0 °C was added a solution of $BH_3{\bullet}SMe_2$ (2.0 M in THF, 0.27 mL, 0.564 mmol, 3.0 equiv.). The reaction mixture was allowed to warm to heat at 50 °C and stirred for 2 hours, monitored by TLC until disappearance of the starting material. It was then recooled to 0 °C, and methanol (0.5 mL) was added slowly followed by addition of 3.0 M NaOH (1.79 mL, 5.37 mmol, 30.0 equiv.) and 30% H_2O_2 (0.37 mL, 3.58 mmol,

30.0 equiv.). The resulting mixture was stirred at room temperature for 0.5 h, then diluted with water and EtOAc. The organic phase was separated, and the aqueous phase extracted with EtOAc. The combined organic extracts were washed with brine, dried over Na_2SO_4 and concentrated. The residue was purified by flash column chromatography (gradient, pentane/EtOAc 6:1 to 3:1) to afford **27** (50 mg, 0.072 mmol, 40%) as a yellow oil; **R_f** 0.23 (pentane/EtOAc 2:1); **IR** (thin film, ν_{max} / cm^{-1}) 3534, 2954, 2931, 2857, 2360, 2342, 1599, 1494, 1448, 1175, 1161, 1121, 911, 874, 838, 814, 780, 737, 707, 674; **^{1}H NMR** (500 MHz, $CDCl_3$) δ_H 8.04-7.98 (1H, m, Ar*H*), 7.88-7.83 (2H, m, Ts*H*), 7.74 (2H, d, *J* = 8.4 Hz, Ts*H*), 7.69-7.64 (1H, m, Ar*H*), 7.36 (3H, dd, *J* = 8.1, 6.4 Hz, 2 x Ts*H* + Ar*H*), 7.31 (1H, dd, *J* = 7.7, 1.1 Hz, Ar*H*), 7.28 (1H, br, Ar*H*), 7.22 (2H, d, *J* = 8.1 Hz, Ts*H*), 3.99 (1H, t, *J* = 4.9 Hz, H6), 3.86 (1H, t, *J* = 4.9 Hz, H5), 3.80 (1H, dd, *J* = 10.1, 3.0 Hz, H7), 3.46-3.35 (2H, m, H8 and H3), 2.70 (1H, dd, *J* = 14.4, 10.0 Hz, H8), 2.47 (3H, s, TsC*H*3), 2.35 (3H, s, TsC*H*3), 2.23 (1H, dt, *J* = 10.9, 5.0 Hz, H4), 2.05 (1H, dd, *J* = 5.3, 2.6 Hz, H1), 1.77-1.72 (1H, m, H4), 1.72 (1H, d, *J* = 2.5 Hz, H2), 1.45 (1H, ddd, *J* = 11.3, 5.6, 3.1 Hz, H2), 0.89 (9H, s, SiC(C*H*3)3), -0.03 (6H, d, *J* = 4.4 Hz, Si(C*H*3)2); **^{13}C NMR** (125 MHz, $CDCl_3$) δ_C 145.1, 143.8, 138.3, 135.4, 135.3, 130.9, 130.03, 129.98, 127.1, 126.9, 125.2, 124.2, 123.6, 119.8, 119.4, 114.0, 68.4, 64.3, 62.1, 60.1, 43.0, 33.0, 32.7, 31.0, 25.8, 21.74, 21.70, 18.1, -4.9, -5.0; **HRMS** (ES$^+$) calc. for $C_{36}H_{46}N_2O_6S_2SiNa$ [M+Na]$^+$ 717.2459, found 717.2453.

(±)-(1*R*,3*S*,5*R*,7*R*,8*R*)-8-[(*Tert*-butyldimethylsilyl)oxy]-6-tosyl-7-[(1-tosyl-1*H*-indol-3-yl)methyl]-6-azabicyclo[3.2.1]octan-3-ol, 28.

(±)-(1*S*,2*S*,5*R*,7*R*,8*R*)-8-[(*Tert*-butyldimethylsilyl)oxy]-6-tosyl-7-[(1-tosyl-1*H*-indol-3-yl)methyl]-6-azabicyclo[3.2.1]octan-2-ol, 29.

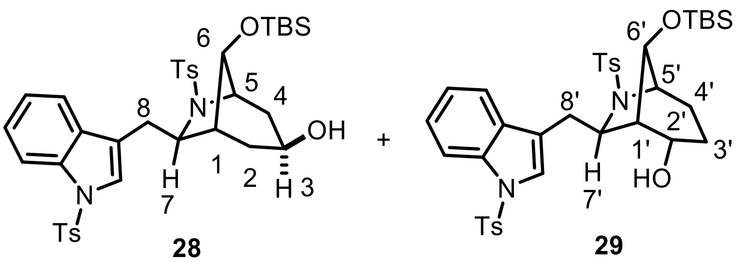

28 **29**

And inseparable **28** and **29** (42 mg, 0.061 mmol, 34%) as a yellow oil; **R_f** 0.26 (Pentane/EtOAc 6:1); **IR** (thin film, ν_{max} / cm^{-1})3558, 2952, 2857, 2362, 2257, 1719, 1598, 1494, 1448, 1212, 1159, 1121, 1094, 1007, 975, 911, 871, 838, 814, 780, 736, 705, 672; **^{1}H NMR** (400 MHz, $CDCl_3$) (**11a/11b** = 1:1.4) δ_H 8.00-7.95 (2.4H, m, 1 x Ar*H* + 1.4 x Ar*H'*), 7.83 (4.8H, t, *J* = 8.1 Hz, 2 x Ts*H* + 2.8 x Ts*H'*),

7.76-7.72 (1.4H, m, Ar*H'*), 7.69 (2.8H, d, *J* = 8.5 Hz, Ts*H'*), 7.68-7.67 (2H, m, Ts*H*), 7.61-7.59 (1H, m, Ar*H*), 7.46 (1H, br, Ar*H*), 7.36-7.32 (4.8H, m, 2 x Ts*H* + 2.8 x Ts*H'*), 7.31 (1.4H, dd, *J* = 3.4, 1.2 Hz, Ar*H'*), 7.30 (1H, dd, *J* = 3.4, 1.3 Hz, Ar*H*), 7.27 (1.4H, d, *J* = 1.1 Hz, Ar*H'*), 7.25 (1.4H, dd, *J* = 7.5, 1.1 Hz, Ar*H'*), 7.22 (1H, br, Ar*H'*), 7.19 (4.8H, dd, *J* = 8.1, 4.6 Hz, 2 x Ts*H* +2.8 x Ts*H'*), 4.64 (1.4H, dd, *J* = 10.8, 3.1 Hz, H7'), 4.11 (1H, dd, *J* = 9.8, 3.0 Hz, H7), 4.06-4.04 (2.4H, m, H6 + H6'), 4.03-4.02 (1.4H, m, H2'), 4.02-4.00 (1.4H, m, H5'), 3.80 (1H, t, *J* = 4.6 Hz, H5), 3.52-3.45 (2.4H, m, H3 + 1.4 x H8'), 3.43 (1H, ddd, *J* = 15.0, 3.1, 1.2 Hz, H8), 2.83 (1H, dd, *J* = 15.0, 9.7 Hz, H8), 2.57 (1.4H, dd, *J* = 14.0, 10.7 Hz, H8'), 2.45 (7.2H, s, TsCH_3 + 4.2 x TsCH_3'), 2.33 (7.2H, s, TsCH_3 + 4.2 x TsCH_3'), 2.15-2.13 (1H, m, H4), 2.12-2.10 (1H, m, H4), 2.09-2.07 (1H, m, H1), 1.99 (1.4H, d, *J* = 3.1 Hz, H3'), 1.95-1.92 (1H, d, *J* = 4.3 Hz, H1'), 1.82-1.77 (1H, m, H2), 1.75-1.68 (1H, m, H2), 1.56-1.48 (1.4H, m, H4'), 1.38 (1.4H, d, *J* = 14.4 Hz, H3'), 0.85 (12.6H, s, SiC(CH_3)$_3$), 0.81 (9H, s, SiC(CH_3)$_3$), 0.75-0.63 (1.4H, m, H4'), -0.04 (8.4H, d, *J* = 9.6 Hz, Si(CH_3)$_2$), -0.13 (6H, d, *J* = 6.5 Hz, Si(CH_3)$_2$); **^{13}C NMR** (100 MHz, $CDCl_3$) δ_C 145.0, 144.9, 143.61, 143.58, 138.7, 138.4, 135.4, 135.3, 131.2, 131.0, 129.9, 127.2, 127.1, 127.0, 126.8, 125.1, 125.0, 124.4, 124.0, 123.7, 123.5, 120.01, 120.13, 119.8, 119.6, 114.0, 113.9, 70.2, 68.9, 65.5, 64.5, 62.1, 59.6, 57.9, 56.8, 50.1, 40.9, 31.6, 31.0, 30.9, 30.7, 29.8, 26.5, 25.79, 25.75, 22.4, 21.74, 21.72, 21.69, 18.1, 18.0, -4.9, -5.0, -5.13, -5.15; **HRMS** (ES$^+$) calc. for $C_{36}H_{46}N_2O_6S_2SiNa$ [M+Na]$^+$ 717.2459, found 717.2453.

(±)-(1*S*,3*R*,5*R*,7*R*,8*R*)-8-[(*Tert*-butyldimethylsilyl)oxy]-6-tosyl-7-[(1-tosyl-1*H*-indol-3-yl)methyl]-6-azabicyclo[3.2.1]octan-3-yl methanesulfonate, S19

(±)-(1*S*,3*R*,5*R*,7*R*,8*R*)-8-Hydroxy-6-tosyl-7-[(1-tosyl-1*H*-indol-3-yl)methyl]-6-azabicy clo[3.2.1]octan-3-yl methanesulfonate, S20

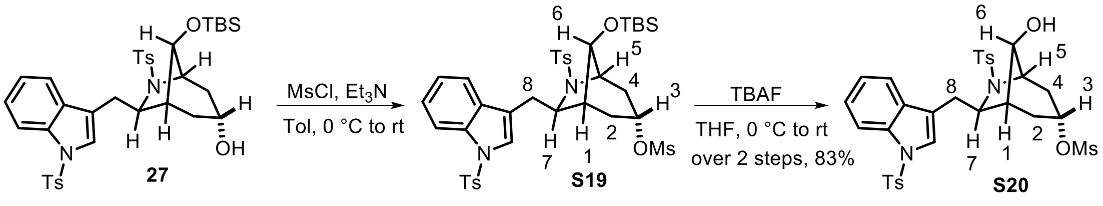

To a stirred solution of **27** (14 mg, 0.02 mmol, 1.0 equiv.) in toluene (1.5 mL) at 0 °C was added Et_3N (10 mg, 0.1 mmol, 5.0 equiv.) and MsCl (5 mg, 0.044 mmol, 2.2 equiv.). The reaction mixture was allowed to warm to room temperature and stirred for 1 hours, monitored by TLC until disappearance of the starting material. It was then quenched by with water a and the aqueous phase extracted with EtOAc. The combined organic extracts were washed with brine, dried over Na_2SO_4 and concentrated.

A sample of **S19** could be obtained for characterisation through purification by flash chromatography (pentane/EtOAc 6:1) as a white oil; $\mathbf{R_f}$ 0.14 (pentane/EtOAc 6:1); **IR** (thin film, ν_{max} / cm^{-1}) 2957, 2930, 2361, 2342, 1736, 1598, 1472, 1362, 1261, 1177, 1162, 972, 955, 912, 873, 738, 670; **^{1}H NMR** (400 MHz, $CDCl_3$) δ_H 8.00 (1H, dt, *J* = 8.2, 0.9 Hz, Ar*H*), 7.85 (2H, d, *J* = 8.4 Hz, Ts*H*), 7.72 (2H, d, *J* = 8.4 Hz, Ts*H*), 7.62 (1H, dt, *J* = 7.7, 1.0 Hz, Ar*H*), 7.38 (2H, d, *J* = 8.1 Hz, Ts*H*), 7.36-7.33 (1H, m, Ar*H*), 7.31-7.27 (2H, m, Ar*H*), 7.23-7.19 (2H, m, Ts*H*), 4.35-4.22 (1H, m, H2), 3.97 (1H, t, *J* = 4.9 Hz, H6), 3.85-3.77 (2H, m, H5 + H7), 3.44-3.35 (1H, m, H8), 2.84 (3H, s, -SO_2CH_3), 2.72 (1H, dd, *J* = 14.3, 9.8 Hz, *H*8), 2.45 (3H, s, TsCH_3), 2.34 (3H, s, TsCH_3), 2.30 (1H, q, *J* = 6.1 Hz, H4), 2.10-2.04 (1H, m, H1), 2.00-1.97 (1H, m, H4), 1.97-1.94 (1H, m, H2), 1.63 (1H, dt, *J* = 11.7, 5.3 Hz, H2), 0.86 (9H, s, SiC(CH_3)$_3$), -0.06 (6H, d, *J* = 6.2 Hz, Si(CH_3)$_2$); **^{13}C NMR** (100 MHz, $CDCl_3$) δ_C 145.2, 144.4, 137.5, 135.4, 135.2, 130.8, 130.4, 130.0, 127.1, 126.9, 125.3, 124.4, 123.8, 119.7, 119.0, 114.1, 75.4, 67.8, 62.0, 59.5, 43.1, 38.3, 38.2, 31.4, 31.0, 30.7, 29.9, 29.6, 25.8, 21.8, 21.7, 18.1, -4.96, -5.03; **HRMS** (ES^+) calc. for $C_{37}H_{49}N_2O_8S_3Si$ $[M+H]^+$ 773.2415, found 773.2396.

To a stirred solution of **S19** (16 mg, 0.02 mmol, 1.0 equiv.) in THF (2.0 mL) at 0 °C was added TBAF (1.0 M in THF, 0.04 mL, 0.04 mmol, 2.0 equiv.). The reaction mixture was allowed to warm to room temperature and stirred for 1 hours, monitored by TLC until disappearance of the starting material. It was then quenched by water and the aqueous phase extracted with EtOAc. The combined organic extracts were washed with brine, dried over Na_2SO_4 and concentrated. The residue was purified by flash column chromatography (pentane/EtOAc 1:1) to afford **S20** (11 mg, 16.7 µmol, 83%) as a white oil; $\mathbf{R_f}$ 0.23 (pentane/EtOAc 1:1); **IR** (thin film, ν_{max} / cm^{-1}) 3510, 2938, 2365, 2332, 1598, 1448, 1358, 1176, 1121, 953, 736, 676; **^{1}H NMR** (400 MHz, $CDCl_3$) δ_H 7.99 (1H, dt, *J* = 8.1, 1.0 Hz, Ar*H*), 7.82 (2H, d, *J* = 8.4 Hz, Ts*H*), 7.71 (2H, d, *J* = 8.4 Hz, Ts*H*), 7.69-7.65 (1H, m, Ar*H*), 7.39-7.27 (5H, m, 2 x Ts*H* + 3 x Ar*H*), 7.22-7.17 (2H, m, Ts*H*), 4.51-4.34 (1H, m, H3), 4.23 (1H, t, *J* = 4.9 Hz, H6), 3.90 (1H, t, *J* = 4.8 Hz, H5), 3.86 (1H, dd, *J* = 10.7, 3.0 Hz, H7), 3.47 (1H, ddd, *J* = 14.2, 3.0, 1.2 Hz, H8), 2.87 (3H, s, -SO_2CH_3), 2.60 (1H, dd, *J* = 14.2, 10.7 Hz, H8), 2.43 (3H, s, TsCH_3), 2.39 (1H, dd, *J* = 12.5, 6.1 Hz, H4), 2.32 (3H, s, TsCH_3), 2.22 (1H, q, *J* = 3.7 Hz, H1), 2.09-2.04 (1H, m, H4), 2.01 (1H, dd, *J* = 10.6, 2.7 Hz, H2), 1.70-1.61 (1H, m, H2); **^{13}C NMR** (100 MHz, $CDCl_3$) δ_C 145.3, 144.4, 137.6, 135.4, 135.1, 130.8, 130.3, 130.0, 127.1, 126.8, 125.3, 124.3, 123.9, 119.8, 119.3, 114.0, 75.4, 67.1, 62.1, 59.1, 42.2, 38.3, 31.0, 30.4, 29.6, 21.72, 21.69; **HRMS** (ES^+) calc. for $C_{31}H_{34}N_2O_8S_3Na$ $[M+Na]^+$ 681.1370, found 681.1362.

(±)-(2*S*)-4-Tosyl-5-[(1-tosyl-1*H*-indol-3-yl)methyl]hexahydro-2*H*-2,6-methanofuro[3,2-b]pyrrole, 30

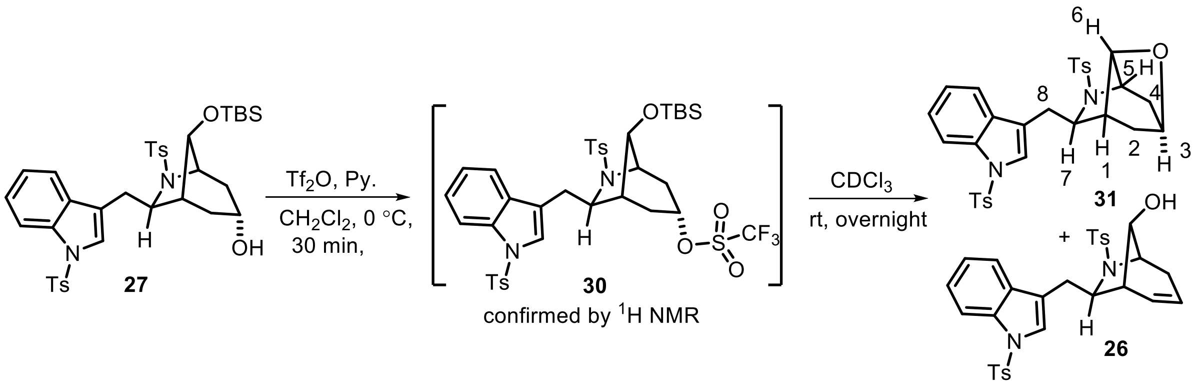

To a stirred solution of **27** (10 mg, 14.4 μmol, 1.0 equiv.) in CH_2Cl_2 (2.0 mL) at 0 °C was added pyridine (3.48 μL, 43.2 μmol, 3.0 equiv.) and Tf_2O (5.6 μL, 31.7 μmol, 2.2 equiv.), stirring for 30 min, then was then quenched with water and the aqueous phase extracted with EtOAc. The organic extract was dried over anhydrous Na_2SO_4, filtered and concentrated. The residue **30** was confirmed by crude ^{1}**H NMR** (400 MHz, $CDCl_3$) δ_H 8.01 (dt, *J* = 8.3, 0.9 Hz, 1H), 7.83 (d, *J* = 8.3 Hz, 2H), 7.74 (d, *J* = 8.4 Hz, 2H), 7.65-7.58 (m, 1H), 7.39-7.33 (m, 3H), 7.32-7.27 (m, 2H), 7.23-7.19 (m, 2H), 4.63-4.56 (m, 1H), 4.45 (tt, *J* = 10.5, 6.7 Hz, 1H), 3.95 (t, *J* = 4.9 Hz, 1H), 3.87 (t, *J* = 4.8 Hz, 1H), 3.74 (dd, *J* = 9.5, 3.1 Hz, 1H), 3.41 (ddd, *J* = 14.4, 3.1, 1.1 Hz, 1H), 2.79 (dd, *J* = 14.4, 9.5 Hz, 1H), 2.45 (s, 3H), 2.40-2.34 (m, 1H), 2.33 (s, 3H), 2.12 (q, *J* = 3.7 Hz, 1H), 2.07-2.04 (m, 1H), 2.04-2.01 (m, 1H), 1.66 (dt, *J* = 11.5, 4.8 Hz, 1H), 0.85 (s, 9H), -0.08 (d, *J* = 4.6 Hz, 6H); Then it stayed overnight in $CDCl_3$ to decompose to **26** and **31**, further flash column chromatography (pentane/EtOAc 4:1 to 2:1) to afford **26** (3.2 mg, 5.69 μmol, 40%) and **31** (3.2 mg, 5.69 μmol, 40%) as a white solid; **R$_f$** 0.15 (pentane/EtOAc 4:1); **IR** (thin film, ν_{max} / cm^{-1}) 2927, 2855, 2360, 2342, 1598, 1449, 1371, 1338, 1175, 1151, 1122, 1096, 1045, 976, 912, 815, 736, 670; ^{1}**H NMR** (400 MHz, $CDCl_3$) δ_H 8.01-7.99 (1H, m, N*H*), 7.82 (2H, d, *J* = 8.3 Hz, Ts*H*), 7.72 (2H, d, *J* = 8.4 Hz, Ts*H*), 7.69-7.64 (1H, m, Ar*H*), 7.37-7.31 (3H, m, 2 x Ts*H* + Ar*H*), 7.29 (1H, dd, *J* = 7.7, 1.2 Hz, Ar*H*), 7.22-7.21 (1H, m, Ar*H*), 7.19 (2H, d, *J* = 2.9 Hz, Ts*H*), 4.51 (1H, t, *J* = 4.9 Hz, H6), 4.36 (1H, t, *J* = 4.6 Hz, H3), 4.04 (1H, dd, *J* = 9.5, 3.0 Hz, H7), 3.99-3.89 (1H, m, H5), 3.33 (1H, ddd, *J* = 14.2, 3.0, 1.1 Hz, H8), 2.62 (1H, dd, *J* = 14.3, 9.5 Hz, H81), 2.45 (3H, s, TsC*H*$_3$), 2.33 (3H, s, TsC*H*$_3$), 2.31-2.23 (1H, m, H1), 1.94-1.78 (1H, m, H2), 1.64-1.56 (2H, m, H4), 1.04 (1H, dd, *J* = 12.5, 2.5 Hz, H2); ^{13}**C NMR** (100 MHz, $CDCl_3$) δ_C 145.2, 143.9, 138.6, 135.4, 135.3, 131.0, 130.1, 130.0, 127.3, 126.9, 125.1, 124.3,

123.7, 119.9, 119.1, 114.0, 81.0, 75.9, 64.4, 59.3, 43.7, 38.3, 37.6, 31.7, 21.7; **HRMS** (ES^+) calc. for $C_{30}H_{31}N_2O_5S_2$ $[M+Na]^+$ 563.1669, found 563.1662.

Alternatively:

To a stirred solution of **27** (10 mg, 14.4 μmol, 1.0 equiv.) in CH_2Cl_2 (2.0 mL) at 0 °C was added pyridine (3.48 μL, 43.2 μmol, 3.0 equiv.) and Tf_2O (5.6 μL, 31.68 μmol, 2.2 equiv.), stirring overnight. It was then quenched with water and the aqueous phase extracted with EtOAc. The organic extract was dried over anhydrous Na_2SO_4, filtered and concentrated. The residue was purified by flash column chromatography (pentane/EtOAc 4:1) to afford **25** (3.9 mg, 5.77 μmol, 40%) and **31** (3.1 mg, 5.52 μmol, 38%) as a white solid.

(±)-(1*S*,5*R*,7*R*,8*R*)-8-[(*Tert*-butyldimethylsilyl)oxy]-6-tosyl-7-[(1-tosyl-1*H*-indol-3-yl)methyl]-6-azabicyclo[3.2.1]octan-3-one, 32

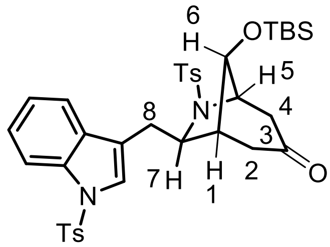

To a stirred solution of **27** (57 mg, 0.082 mmol, 1.0 equiv.) in CH_2Cl_2 (2.0 mL) at 0 °C was added $NaHCO_3$ (69 mg, 0.82 mmol, 10.0 equiv.), DMP (52 mg, 0.123 mmol, 1.5 equiv.). The reaction mixture was allowed to warm to room temperature and stirred for 1 hour, monitored by TLC until disappearance of the starting material. It was then quenched with *sat.* 0.5 mL $NaHCO_3$ (aq.) and 0.5 mL *sat.* $Na_2S_2O_3$ (aq.) then diluted with water and CH_2Cl_2. The organic phase was separated, and the aqueous phase extracted with CH_2Cl_2. The combined organic extracts were washed with brine, dried over Na_2SO_4 and concentrated. The residue was purified by flash column chromatography (pentane/EtOAc 4:1) to afford **32** (52 mg, 0.075 mmol, 92%) as a white oil; $\mathbf{R_f}$ 0.26 (pentane/EtOAc 4:1); **IR** (thin film, ν_{max} / cm^{-1}) 2952, 2930, 2858, 2361, 2341, 1718, 1598, 1448, 1374, 1345, 1175, 1161, 1123, 1090, 911, 868, 839, 815, 782, 702, 672; **^{1}H NMR** (400 MHz, $CDCl_3$) δ_H 8.00 (1H, d, *J* = 8.3 Hz, Ar*H*), 7.75 (4H, dd, *J* = 9.9, 8.3 Hz, Ts*H*), 7.60 (1H, d, *J* = 7.8 Hz, Ar*H*), 7.39-7.26 (5H, m, 4 x Ts*H* + Ar*H*), 7.21 (2H, d, *J* = 8.1 Hz, Ar*H*), 4.23 (1H, t, *J* = 5.2 Hz, H6), 4.12 (1H, q, *J* = 3.2 Hz, H5), 3.77 (1H, dd, *J* = 9.6, 3.1 Hz, H7), 3.50-3.38 (1H, m, H8), 2.84 (1H, dd, *J* = 14.5, 9.6 Hz,

H8), 2.67-2.55 (2H, m, H4), 2.55-2.50 (1H, m, H2), 2.46 (3H, s, TsCH_3), 2.34 (3H, s, TsCH_3), 2.29 (1H, br, H1), 1.87 (1H, d, J = 17.3 Hz, H2), 0.82 (9H, s, SiC(CH_3)$_3$), -0.05 (6H, d, J = 3.5 Hz, Si(CH_3)$_2$); **^{13}C NMR** (100 MHz, $CDCl_3$) δ_C 207.3, 145.2, 144.2, 137.6, 135.4, 135.3, 130.8, 130.2, 130.0, 127.4, 126.9, 125.3, 124.4, 123.7, 119.7, 118.8, 114.1, 68.4, 63.8, 59.5, 43.0, 42.5, 41.1, 31.1, 25.7, 21.8, 21.7, 18.0, -5.0, -5.1; **HRMS** (ES$^+$) calc. for $C_{36}H_{45}N_2O_6S_2Si$ [M+H]$^+$ 693.2483, found 693.2464.

(±)-(1*S*,5*R*,7*R*,8*R*)-8-[(*Tert*-butyldimethylsilyl)oxy]-6-tosyl-7-[(1-tosyl-1*H*-indol-3-yl)methyl]-6-azabicyclo[3.2.1]octan-2-one, S40

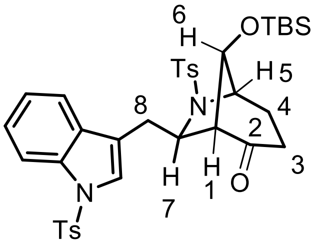

To a stirred solution of **28** and **29** (23 mg, 33.1 μmol, 1.0 equiv.) in CH_2Cl_2 (2.0 mL) at 0 °C was added $NaHCO_3$ (28 mg, 331 μmol, 10 equiv.), DMP (21 mg, 49. 7 μmol, 1.5 equiv.). The reaction mixture was allowed to warm to room temperature and stirred for 1 hour, monitored by TLC until disappearance of the starting material. It was then quenched with *sat.* $NaHCO_3$ (*aq.*) and *sat.* $Na_2S_2O_3$ (*aq.*) then diluted with water and EtOAc. The organic phase was separated, and the aqueous phase extracted with EtOAc. The combined organic extracts were washed with brine, dried over Na_2SO_4 and concentrated. The residue was purified by flash column chromatography (pentane/EtOAc 8:1) to afford **32** (10 mg, 14.4 μmol, 44%) as a white oil and **S40** (10 mg, 14.4 μmol, 44%) as a white oil; **R_f** 0.16 (pentane/EtOAc 10:1); **IR** (thin film, ν_{max} / cm^{-1}) 2955, 2930, 2894, 2858, 2364, 2341, 1721, 1598, 1448, 1373, 1350, 1341, 1175, 1123, 865, 746, 735, 672; **^{1}H NMR** (400 MHz, $CDCl_3$) δ_H 7.98 (1H, dt, J = 8.2, 0.9 Hz, Ar*H*), 7.88 (2H, d, J = 8.3 Hz, Ts*H*), 7.75 (2H, d, J = 8.4 Hz, Ts*H*), 7.65-7.59 (1H, m, Ar*H*), 7.39-7.35 (2H, m, Ts*H*), 7.34-7.28 (2H, m, Ar*H*), 7.26-7.22 (3H, m, 2x Ts*H* + Ar*H*), 4.10 (1H, t, J = 5.2 Hz, H6), 4.02 (1H, d, J = 3.9 Hz, H5), 3.95 (1H, dd, J = 8.3, 2.9 Hz, H7), 3.32 (1H, ddd, J = 14.7, 3.0, 1.0 Hz, H8), 3.01 (1H, dd, J = 14.7, 8.3 Hz, H8), 2.66 (1H, d, J = 5.1 Hz, H1), 2.46 (3H, s, TsCH_3), 2.33 (3H, s, TsCH_3), 2.33-2.17 (1H, m, H4), 2.01-1.96 (1H, m, H3), 1.96-1.93 (1H, m, H4), 1.64-1.58 (1H, m, H3), 0.73 (9H, s, SiC(CH_3)$_3$), -0.18 – -0.32 (6H, m, Si(CH_3)$_2$); **^{13}C NMR** (100 MHz, $CDCl_3$) δ_C 207.8, 145.1, 144.3, 137.4, 135.3, 130.8, 130.2, 130.1,

127.3, 127.0, 125.2, 125.1, 123.7, 119.6, 117.9, 114.1, 71.8, 60.3, 60.0, 59.8, 34.0, 30.8, 25.6, 24.0, 21.8, 21.7, 17.9, -5.2, -5.5; **HRMS** (ES^+) calc. for $C_{36}H_{44}N_2O_6S_2SiNa$ $[M+Na]^+$ 715.2302, found 715.2283.

(±)-*N'*-{(1*R*,5*R*,7*R*,8*R*,*Z*)-8-[(*Tert*-butyldimethylsilyl)oxy]-6-tosyl-7-[(1-tosyl-1*H*-indol-3-yl)methyl]-6-azabicyclo[3.2.1]octan-3-ylidene}-4-methylbenzenesulfonohydrazide, 33

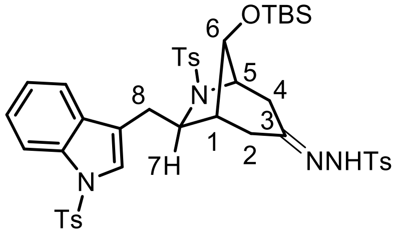

To a stirred solution of **32** (133 mg, 0.192 mmol, 1.0 equiv.) in MeOH (2.0 mL) at room temperature was added $TsNHNH_2$ (79 mg, 0.422 mmol, 2.2 equiv.) and $TsOH•H_2O$ (7.3 mg, 38.4 µmol, 0.2 equiv.). The reaction mixture was allowed to warm to warm up to 80 °C and stirred for 1.5 hour, monitored by TLC until disappearance of the starting material. It was concentrated then diluted with water and EtOAc. The organic phase was separated, and the aqueous phase extracted with EtOAc. The combined organic extracts were washed with brine, dried over Na_2SO_4 and concentrated. The residue was purified by flash column chromatography (pentane/EtOAc 4:1) to afford **33** (162 mg, 0.188 mmol, 98%) as a yellow oil; $\mathbf{R_f}$ 0.16 (pentane/EtOAc 4:1); **IR** (thin film, ν_{max} / cm^{-1}) 2941, 2359, 2343, 1746, 1599, 1448, 1374, 1339, 1172, 1121, 1097, 1048, 1018, 872, 840, 814, 779, 669, 649; **^{1}H NMR** (400 MHz, $CDCl_3$) (*Z*:*E*=1.66:1) δ_H 8.02-7.94 (m, 2.66H), 7.86-7.80 (m, 3.32H), 7.77-7.72 (m, 10.64H), 7.70-7.65 (m, 3.32H), 7.47 (ddt, *J* = 15.4, 7.9, 1.1 Hz, 2.66H), 7.39-7.35 (m, 3.66H), 7.31 (td, *J* = 7.6, 1.9 Hz, 10.64H), 7.23 (dd, *J* = 7.7, 3.1 Hz, 3.32H), 7.20 -7.17 (m, 4.0H), 6.95 (br, 1H), 4.15-4.02 (m, 2.66H), 3.96 (dt, *J* = 5.5, 2.6 Hz, 1.0H), 3.74 (dd, *J* = 10.3, 3.2 Hz, 1.66H), 3.63-3.57 (m, 1.66H), 3.54 (dd, *J* = 9.4, 3.2 Hz, 1.0H), 3.40-3.31 (m, 2.66H), 2.99 (ddd, *J* = 15.6, 4.1, 1.8 Hz, 1.66H), 2.78 (dd, *J* = 14.5, 9.5 Hz, 1.0H), 2.69-2.59 (m, 1.66H), 2.64-2.54 (m, 2.66H), 2.47 (s, 3.0H), 2.45 (s, 9.96H), 2.43 (s, 3H), 2.42-2.41 (m, 1.0H), 2.34 (s, 3.0H), 2.33 (s, 4.98H), 2.22 (d, *J* = 15.2 Hz, 1.66H), 2.16-2.11 (m, 1.66H), 2.08-2.05 (m, 1.66H), 2.00 (br, 1.0H), 1.90-1.82 (m, 1.66H), 0.80 (s, 19.94H), 0.74 (s, 9.0H), -0.08 (d, *J* = 7.2 Hz, 9.96H), -0.12 (d, *J* = 2.5 Hz, 6.0H); **^{13}C NMR** (100 MHz, $CDCl_3$) δ_C 155.9, 155.5, 145.19, 145.16, 144.6, 144.2, 144.1, 143.8, 137.3, 137.2, 135.6, 135.5, 135.4, 135.29, 135.27, 135.2, 130.7, 130.6, 130.3, 130.2, 130.03, 129.99, 129.7, 129.5, 128.3, 128.1, 127.4, 127.2, 126.9, 126.8, 125.2, 124.5, 124.2, 123.6, 123.5, 119.7, 119.6, 118.9, 118.6, 114.1,

114.0, 68.9, 68.5, 63.5, 62.5, 58.7, 58.5, 42.4, 41.4, 34.5, 33.3, 30.8, 28.3, 27.2, 25.69, 25.66, 21.80, 21.78, 21.75, 21.69, 18.0, 17.97, -4.9, -5.06, -5.09, -5.14; **HRMS** (ES^+) calc. for $C_{43}H_{53}N_4O_7S_3Si$ $[M+H]^+$ 861.2840 found 861.2825.

(±)-3-{[(1*R*,5*R*,7*R*,8*R*)-8-[(*Tert*-butyldimethylsilyl)oxy]-6-tosyl-6-azabicyclo[3.2.1]oct-2-en-7-yl]methyl}-1*H*-indole, 34

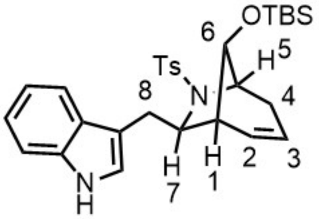

Sodium hydride (10 mg, 24.4 μmol, 10.0 equiv.) was added to dry 1,4-dioxane (0.5 mL). The suspension was stirred and heated to reflux, and then a solution of a tosylhydrazone **33** (21 mg, 24.4 μmol, 1.0 equiv.) in dry 1,4-dioxane (1.0 mL) was added dropwise. the mixture was cooled and the insoluble material filtered off. The solvent was removed under reduced pressure, residue was purified by column chromatography (pentane/EtOAc 10:1) to afford **25** (5 mg, 7.40 μmol, 30%) and **34** (7.0 mg, 13.4 μmol, 55%) as a white oil; $\mathbf{R_f}$ 0.33 (pentane/EtOAc 6:1); **IR** (thin film, ν_{max} / cm^{-1}) 3411, 2955, 2927, 2858, 2360, 2341, 2330, 1459, 1426, 1334, 1252, 1231, 1158, 889, 841, 778, 741, 671; **^{1}H NMR** (400 MHz, $CDCl_3$) δ_H 8.03 (1H, s, N*H*), 7.87-7.79 (2H, m, Ts*H*), 7.76-7.72 (1H, m, Ar*H*), 7.38 (1H, dt, *J* = 8.0, 1.0 Hz, Ar*H*), 7.28-7.26 (2H, m, Ts*H*), 7.24-7.19 (1H, m, Ar*H*), 7.16 (1H, ddd, *J* = 8.0, 7.0, 1.1 Hz, Ar*H*), 7.01 (1H, d, *J* = 2.3 Hz, Ar*H*), 5.42-5.27 (1H, m, H2), 5.26-5.15 (1H, m, H3), 4.41 (1H, ddd, *J* = 5.5, 4.0, 1.4 Hz, H6), 4.02 (1H, dd, *J* = 10.7, 3.2 Hz, H7), 4.00-3.97 (1H, m, H5), 3.50 (1H, ddd, *J* = 14.3, 3.3, 1.0 Hz, H8), 2.83 (1H, dd, *J* = 14.3, 10.7 Hz, H8), 2.41 (3H, s, TsCH_3), 2.34 (1H, dd, *J* = 6.2, 4.0 Hz, H1), 2.25-2.22 (2H, m, H4), 0.82 (9H, s, $SiC(CH_3)_3$), -0.01 (6H, d, *J* = 16.3 Hz, $Si(CH_3)_2$); **^{13}C NMR** (100 MHz, $CDCl_3$) δ_C 143.1, 139.0, 136.3, 129.5, 127.7, 127.5, 126.7, 126.0, 122.5, 122.4, 119.9, 119.3, 113.3, 111.3, 69.2, 68.3, 58.8, 42.7, 31.5, 28.3, 25.8, 21.7, 18.1, -4.8, -4.9; **HRMS** (ES^+) calc. for $C_{29}H_{39}N_2O_3SSi[M+H]^+$ 523.2445 found 523.2437.

(±)-3-{[(1*R*,5*R*,7*R*,8*R*)-8-[(*Tert*-butyldimethylsilyl)oxy]-6-tosyl-6-azabicyclo[3.2.1]oct-3-en-7-yl]methyl}-1-tosyl-1*H*-indole, 35

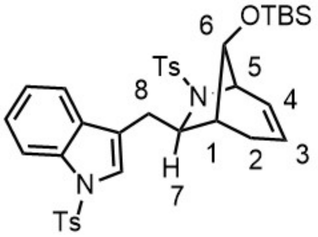

To a solution of **33** (17 mg, 0.02 mmol, 1.0 equiv.) in THF (2.0 mL) was added *n*-BuLi (2.1 M, 0.06 mL, 0.132 mmol, 6.6 equiv.) at -78 °C, stirring for 30 min then was allowed to warm up to 0 °C, stirring for further 30 min. Then it was quenched with *sat.* NH_4Cl (1.0 mL) and extracted with EtOAc (3 x 10 mL). The combined organic phases were washed with brine, dried over Na_2SO_4, and concentrated. Silica gel flash chromatography (pentane/EtOAc 5:1) afforded compound afford **25** (1.1 mg, 1.6 μmol, <5%) and the title **35** (1.2 mg, 1.8 μmol, <5%) as a white oil; **R$_f$** 0.27 (pentane/EtOAc 6:1); **IR** (thin film, ν_{max} / cm^{-1}) 3413, 2971, 2930, 2858, 2360, 2341, 1598, 1460, 1449, 1378, 1329, 1252, 1174, 1160, 1133, 1098, 952, 838, 776, 756, 704, 674; **^{1}H NMR** (400 MHz, $CDCl_3$) δ_H 7.93 (1H, dt, *J* = 8.2, 0.9 Hz, Ar*H*), 7.84-7.78 (1H, m, Ar*H*), 7.72 (2H, d, *J* = 8.5 Hz, Ts*H*), 7.39 (2H, d, *J* = 8.3 Hz, Ts*H*), 7.33-7.27 (2H, m, Ar*H*), 7.23 (1H, br, Ar*H*), 7.21-7.16 (2H, m, Ts*H*), 7.10-7.05 (2H, m, Ts*H*), 5.60 (1H, dd, *J* = 9.7, 2.8 Hz, H3), 5.53-5.45 (1H, m, H4), 4.39 (1H, d, *J* = 7.8 Hz, H6), 4.02 (1H, dt, *J* = 11.4, 3.9 Hz, H5), 3.58 (1H, dd, *J* = 10.3, 7.7 Hz, H7), 2.92-2.81 (1H, m, H8), 2.60 (1H, dd, *J* = 15.3, 10.4 Hz, H8), 2.39 (3H, s, TsCH_3), 2.31 (3H, s, TsCH_3), 2.16-2.13 (1H, m, H2), 2.15-2.08 (1H, m, H1), 1.91-1.84 (1H, m, H2), 0.96 (9H, s, $SiC(CH_3)_3$), 0.19-0.06 (6H, m, $Si(CH_3)_2$); **^{13}C NMR** (100 MHz, $CDCl_3$) δ_C 144.9, 143.5, 137.1, 135.4, 135.1, 131.2, 130.0, 129.6, 129.4, 127.4, 127.0, 126.8, 124.8, 124.7, 124.3, 123.3, 119.0, 118.3, 113.9, 75.4, 53.9, 45.4, 44.8, 26.5, 25.1, 24.9, 21.71, 21.69, 18.9, -2.4, -2.7; **HRMS** (ES^+) calc. for $C_{36}H_{44}N_2O_5S_2SiNa$ $[M+Na]^+$ 699.2353, found 699.2343.

(1*E*,1'*E*)-*N*,*N*'-((1*R*,2*R*)-Cyclohexane-1,2-diyl)bis(1-(2,6-dichlorophenyl)methanimine), L4

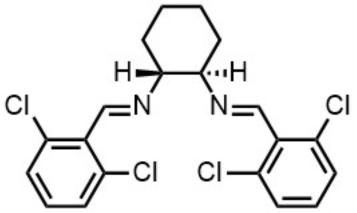

Synthesized according to literature procedure.[22] A 10 mL flask was charged with 2,6-

dichlorobenzaldehyde (232 mg, 1.33 mmol, 2.0 equiv.), (*R*, *R*)-diaminocyclohexane (75.7 mg, 0.663 mmol, 1.0 equiv.) and absolute ethanol (5.0 mL). The resulting solution was heated to reflux for one hour and then allowed to cool to room temperature. Compound **L4** crystallized from the cooled solution, and it was collected by filtration and dried in high vacuum to obtained **L4** (212 mg, 0.498 mmol, 75%); **^{1}H NMR** (400 MHz, $CDCl_3$) δ_H 8.47 (s, 2H), 7.30-7.23 (m, 4H), 7.16 (dd, *J* = 8.7, 7.3 Hz, 2H), 3.65-3.53 (m, 2H), 1.95-1.80 (m, 6H), 1.56-1.46 (m, 2H); **^{13}C NMR** (100 MHz, $CDCl_3$) δ_C 156.7, 135.0, 133.1, 130.1, 128.8, 75.1, 33.0, 24.4; Data identical to literature values.[22]

(±)-*N*-((1*R*,2*R*)-2-Hydroxycyclohexyl)-4-methylbenzenesulfonamide, S28

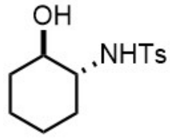

To a stirred solution of cyclohexene oxide **S27** (981.4 mg, 10.0 mmol, 1.0 equiv.) in dioxane (5.0 mL) were added anhydrous potassium carbonate (138.2 mg, 1.0 mmol, 0.1 equiv.), benzyltriethylammonium chloride (227.8 mg, 1.0 mmol, 0.1 equiv.) and $TsNH_2$ (2.05 g, 12.0 mmol, 1.2 equiv.) The mixture was allowed to stir for 60 hours at 90°C under argon atmosphere. After completion of the reaction, dichloromethane (25 mL) was added and the solids were removed by filtration through Celite. The filtrate was dried and evaporated under reduced pressure to give the crude product. Silica gel column chromatography (pentane/EtOAc 2:1) afforded **S28** (2.25 g, 8.37 mmol, 84%) as a white solid; **^{1}H NMR** (400 MHz, $CDCl_3$) δ_H 7.79 (d, *J* = 8.3 Hz, 2H), 7.29 (d, *J* = 8.1 Hz, 2H), 5.40 (d, *J* = 7.2 Hz, 1H), 3.30 (td, *J* = 9.2, 3.3 Hz, 1H), 2.98 (d, *J* = 3.3 Hz, 1H), 2.89-2.81 (m, 1H), 2.41 (s, 3H), 2.05-1.92 (m, 1H), 1.74-1.48 (m, 3H), 1.25-1.02 (m, 4H); **^{13}C NMR** (100 MHz, $CDCl_3$) δ_C 143.6, 137.6, 129.9, 127.2, 73.3, 59.8, 33.5, 31.8, 24.7, 23.9, 21.6; Data identical to literature values.[23]

4-Methyl-*N*-(2-oxocyclohexyl)benzenesulfonamide, S29

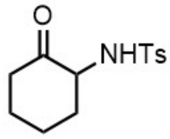

To a stirred solution of **S28** (321 mg, 1.19 mmol, 1.0 equiv.) in CH_2Cl_2 (5.5 mL) were added $NaHCO_3$ (500 mg, 5.95 mmol, 5.0 equiv.), DMP (760 mg, 1.790 mmol, 1.5 equiv.) at 0 °C; The reaction mixture was allowed to warm to room temperature and stirred for 1 hour, monitored by TLC until

disappearance of the starting material. It was then quenched with *sat.* 0.5 mL $NaHCO_3$ (*aq.*) and 0.5 mL *sat.* $Na_2S_2O_3$ (*aq.*) then diluted with water and CH_2Cl_2. The organic phase was separated, and the aqueous phase extracted with CH_2Cl_2. The combined organic extracts were washed with brine, dried over Na_2SO_4 and concentrated. The residue was purified by flash column chromatography (pentane/EtOAc 3:1) to afford **S29** (191 mg, 0.715 mmol, 60%) as a white oil; **^{1}H NMR** (400 MHz, $CDCl_3$) δ_H 7.76-7.65 (m, 2H), 7.27 (dd, *J* = 9.2, 1.4 Hz, 2H), 5.77 (d, *J* = 4.6 Hz, 1H), 3.78-3.72 (m, 1H), 2.56-2.51 (m, 1H), 2.50-2.45 (m, 1H), 2.40 (s, 3H), 2.22-2.18 (m, 1H), 2.10-2.03 (m, 1H), 1.88-1.83 (m, 1H), 1.73-1.62 (m, 1H), 1.62-1.55 (m, 1H), 1.55-1.47 (m, 1H); **^{13}C NMR** (100 MHz, $CDCl_3$) δ_C 205.9, 143.7, 137.1, 129.9, 127.1, 60.8, 40.9, 37.0, 27.5, 24.1, 21.7; Data identical to literature values.[24]

_N_-(Cyclohex-2-en-1-yl)-4-methylbenzenesulfonamide, (±)-38

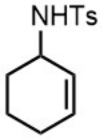

To a mixture of $Bi(OTf)_3$(20 mg, 0.03 mmol, 0.05 equiv.), KPF_6 (5.5 mg, 0.03 mmol, 0.05 equiv.), and $CaSO_4$ (110 mg) in a RBF was added 1,4-dioxane (2.0 mL). After being stirred for 10 min at room temperature, $TsNH_2$ (147 mg, 0.86 mmol, 1.5 equiv.) and then cyclohex-2-en-1-ol (115 mg, 0.57 mmol, 1.0 equiv.) were successively added. The reaction mixture was stirred at 40 °C for 2 hours. The mixture was diluted with diethyl ether (5 mL) and silica gel (150 mg) was added to the mixture. After filtration and washing with diethyl ether, solvent was removed under reduced pressure. The residue was purified by silica gel column chromatography (pentane/EtOAc 15 :1) to give **(±)-38** (141 mg, 0.56 mmol, 99%) as a white solid; **^{1}H NMR** (400 MHz, $CDCl_3$) δ_H 7.77 (d, *J* = 8.3 Hz, 2H), 7.32-7.25 (m, 2H), 5.73 (dtd, *J* = 9.7, 3.7, 1.7 Hz, 1H), 5.33 (ddt, *J* = 10.0, 3.9, 2.2 Hz, 1H), 4.85 (d, *J* = 7.9 Hz, 1H), 3.79 (tdd, *J* = 9.3, 4.6, 2.4 Hz, 1H), 2.41 (s, 3H), 1.97-1.86 (m, 2H), 1.74 (qd, *J* = 7.8, 4.1 Hz, 1H), 1.61-1.44 (m, 3H); **^{13}C NMR** (100 MHz, $CDCl_3$) δ_C 143.3, 138.4, 131.5, 129.8, 127.14, 127.07, 49.1, 30.3, 24.5, 21.6, 19.4; Data identical to literature values.[25]

(±)-(2*S*)-2-Iodo-7-tosyl-7-azabicyclo[4.1.0]heptane, (±)-36

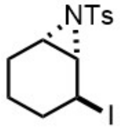

To a solution of **(±)-38** (128 mg, 0.51 mmol, 1.0 equiv.) in toluene (2.0 mL) was added KO*t*-Bu (114 mg, 1.02 mmol, 2.0 equiv.) at room temperature, stirring for 30 min. Then to this mixture was added I_2 (388 mg, 1.53 mmol, 3.0 equiv.), stirring for 30 min. Quenching it with Na_2SO_3 (sat. aq.), diluted with water and EtOAc. The organic phase was separated, and the aqueous phase extracted with EtOAc. The combined organic extracts were washed with brine, dried over Na_2SO_4 and concentrated. The residue was purified by flash column chromatography (pentane/EtOAc 10:1) to afford **(±)-36** (98 mg, 0.26 mmol, 51%) as a yellow oil; **^{1}H NMR** (400 MHz, $CDCl_3$) δ_H 7.72 (d, *J* = 8.4 Hz, 2H), 7.33-7.23 (m, 2H), 4.38 (td, *J* = 4.5, 1.5 Hz, 1H), 3.39 (dt, *J* = 6.7, 1.1 Hz, 1H), 3.02 (ddd, *J* = 6.9, 5.7, 1.1 Hz, 1H), 2.39 (s, 2H), 1.94-1.83 (m, 1H), 1.80-1.69 (m, 2H), 1.69-1.59 (m, 1H), 1.56-1.49 (m, 1H), 1.37-1.28 (m, 1H); **^{13}C NMR** (100 MHz, $CDCl_3$) δ_C 144.7, 135.2, 129.9, 127.9, 46.1, 40.2, 29.9, 24.9, 21.8, 21.5, 17.7; Data identical to literature values.[26]

_N_-(Cyclohexa-2,5-dien-1-yl)-4-methylbenzenesulfonamide, S34

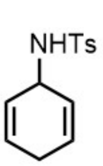

To a solution of **(±)-36** (18 mg, 0.05 mmol, 1.0 equiv.) in THF (1.5 mL) was added KO*t*-Bu (17 mg, 0.15 mmol, 3.0 equiv.) at room temperature, then it was allowed to heat at 60 °C stirring for 1 h. Cooling down to rt and quenching it with NH_4Cl (*sat. aq.*), diluted with water and EtOAc. The organic phase was separated, and the aqueous phase extracted with EtOAc. The combined organic extracts were washed with brine, dried over Na_2SO_4 and concentrated. The residue was purified by flash column chromatography (pentane/EtOAc 6:1) to afford **S34** (6.3 mg, 25.3 μmol, 51%) as a yellow oil; **R_f** 0.20 (pentane/EtOAc 6:1); **IR** (thin film, ν_{max} / cm^{-1}) 3266, 3043, 2923, 2869, 2363, 1598, 1495, 1427, 1408, 1328, 1159, 1094, 1036, 964, 947, 937, 816, 737, 688, 665; **^{1}H NMR** (400 MHz, $CDCl_3$) δ_H 7.75 (2H, d, *J* = 8.3 Hz, Ts*H*), 7.30 (2H, d, *J* = 7.8 Hz, Ts*H*), 5.99-5.93 (2H, m, 2 x CHC*H*=), 5.82-5.69 (1H, m, CH=C*H*CH$_2$), 5.61-5.50 (1H, m, CH=C*H*CH$_2$), 4.63 (1H, d, *J* = 9.2 Hz, N*H*), 3.98-3.82 (1H, m, NHC*H*), 2.43 (3H, s, TsC*H*$_3$), 2.38 (2H, ddd, *J* = 6.1, 4.2, 1.6 Hz, CH=CHC*H*$_2$); **^{13}C NMR** (100 MHz, $CDCl_3$) δ_C 143.5, 138.5, 129.9, 127.1, 126.6, 125.3, 125.1, 123.9, 46.4, 30.5, 21.7; **HRMS** (ES+) calc. for $C_{13}H_{16}NO_2S$ $[M+H]^+$ 250.0896, found 250.0892.

Cyclohex-2-en-1-yl benzoate, 37

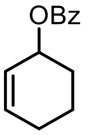

To a solution of the cyclohex-2-en-1-ol (2.64 g, 26.94 mmol, 1.0 equiv.) in CH_2Cl_2 (45 mL) was added Et_3N (11.2 mL, 80.82 mmol, 3.0 equiv.) and benzoyl chloride (3.44 mL, 29.63 mmol, 1.1 equiv.) at 0 °C. It was then allowed to warm up to room temperature, stirring for 3 hours. Quenching it with 5 mL H_2O. The organic phase was separated, and the aqueous phase extracted with CH_2Cl_2. The combined organic extracts were washed with brine, dried over Na_2SO_4 and concentrated. The residue was purified by flash column chromatography (pentane/EtOAc 50:1) to afford **37** (4.90 g, 24.24 mmol, 90%) as a white oil; **^{1}H NMR** (400 MHz, $CDCl_3$) δ_H 8.14-7.98 (m, 2H), 7.56-7.51 (m, 1H), 7.45-7.39 (m, 2H), 6.00 (dtd, *J* = 10.1, 3.8, 1.2 Hz, 1H), 5.84 (ddt, *J* = 10.0, 4.0, 2.2 Hz, 1H), 5.52 (dtq, *J* = 5.3, 3.6, 1.7 Hz, 1H), 2.20-2.03 (m, 2H), 2.02-1.88 (m, 2H), 1.87-1.80 (m, 1H), 1.75-1.64 (m, 1H); **^{13}C NMR** (100 MHz, $CDCl_3$) δ_C 166.3, 132.9, 132.8, 130.9, 129.7, 128.3, 128.3, 125.8, 68.7, 28.5, 25.0, 19.0; Data identical to literature values.[27]

Cyclohex-2-en-1-yl methyl carbonate, S31

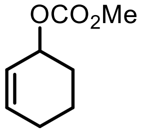

To a solution of cyclohex-2-en-1-ol (281 mg, 2.87 mmol, 1.0 equiv.) and pyridine (0.69 mL, 8.61 mmol, 3.0 equiv.) in CH_2Cl_2 (4.0 mL), cooled in ice water bath under N_2, was added dropwise a solution of methyl chloroformate (0.45 mL, 5.74 mmol, 2.0 equiv.). The mixture was stirred overnight, during which time the ice bath warmed up to ambient temperature. The reaction was quenched with water and diluted with CH_2Cl_2. The organic layer was separated and washed with 1N HCl, saturated $NaHCO_3$, saturated brine, dried over Na_2SO_4 and the crude product was then purified by flash column chromatography (pentane/EtOAc 500:1) to afford **S31** (412 mg, 2.64 mmol, 92%) as a yellow oil; **^{1}H NMR** (400 MHz, $CDCl_3$) δ_H 6.02-5.92 (m, 1H), 5.78-5.74 (m, 1H), 5.13-5.09 (m, 1H), 3.77 (s, 3H), 2.14-1.96 (m, 2H), 1.90-1.71 (m, 2H), 1.67-1.61 (m, 1H); **^{13}C NMR** (100 MHz, $CDCl_3$) δ_C 155.6, 133.5, 125.1, 72.0, 54.6, 28.3, 25.0, 18.7; Data identical to literature values.[28]

(*S*)-cyclohex-2-en-1-ol, (–)-S32

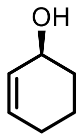

A heatgun-dried RBF was charged with $Pd_2(dba_3)$•$CHCl_3$ (20.7 mg, 0.02 mmol, 0.02 equiv.), (*R*,*R*)-Trost ligand (55 mg, 0.08 mmol, 0.08 equiv.) and degassed CH_2Cl_2 (3.5 mL), and the resulting orange solution was stirred at room temperature for 15 min. Then water (0.8 mL) and a solution of (±)-**31** (156 mg, 1.0 mmol, 1.0 equiv.) in degassed CH_2Cl_2 (3.5 mL) were added. After stirring the mixture at room temperature for 24 h, it filtered through a layer of silica gel with pentane/ether (1:1), then the filtrate was dried with Na_2SO_4 and concentrated in vacuo. Purification of the residue by chromatography on silica gel (gradient, pentane/Et_2O, 30:1 to 1:1) afforded enantiopure alcohol **(–)-S32** (90 mg, 0.92 mmol, 92%); **^{1}H NMR** (400 MHz, $CDCl_3$) δ_H 5.84-5.77 (m, 1H), 5.75-5.68 (m, 1H), 4.17 (br, 1H), 2.07-1.92 (m, 3H), 1.92-1.82 (m, 2H), 1.76-1.66 (m, 1H), 1.64-1.51 (m, 2H); **^{13}C NMR** (100 MHz, $CDCl_3$) δ_C 130.6, 130.0, 65.6, 32.1, 25.1, 19.0; Data identical to literature values.[29]

Methyl (*R*)-cyclohex-2-en-1-yl(tosyl)carbamate, (+)-S33

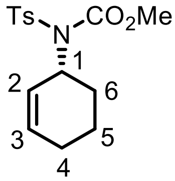

A solution of PPh_3 (724 mg, 2.76 mmol, 3.0 equiv.), DIAD (0.54 mL, 2.76 mmol, 3.0 equiv.), TsNH(CO_2Me) (315 mg, 1.38 mmol, 1.5 equiv.) in THF (12 mL) at 0 °C was stirred for 30 min, then a solution of enantioenriched **(–)-S32** (90 mg, 0.92 mmol, 1.0 equiv.) in THF (4.6 mL) was added. The reaction was allowed to warm to room temperature and stirred for an additional 1 h. The solvent was removed under reduced pressure. The crude product was then purified by flash column chromatography (pentane/EtOAc 20:1) to afford **(+)-S33** (116 mg, 0.375 mmol, 41%, 76% ee) as a yellow oil; $[\alpha]_D^{25}$ = +119.8 (c = 1.7, $CHCl_3$); **R_f** 0.26 (pentane/EtOAc 10:1); **IR** (thin film, ν_{max} / cm^{-1}) 3030, 2955, 2941, 2873, 1738, 1598, 1440, 1360, 1268, 1243, 1171, 1121, 1006, 917, 816, 768, 736, 667; **^{1}H NMR** (400 MHz, $CDCl_3$) δ_H 7.76 (2H, d, *J* = 8.1 Hz, 2 x Ts*H*), 7.24 (2H, d, *J* = 8.1 Hz, 2 x Ts*H*), 5.74-5.65 (1H, m H2), 5.45-5.37 (m, H3), 5.12-5.02 (1H, m, H1), 3.58 (3H, s, CO_2CH_3), 2.37 (s, TsCH_3), 2.15-2.08 (1H, m, H6), 2.03-1.97 (1H, m, H6), 1.97-1.87 (2H, m, H4), 1.86-1.79 (1H, m, H5), 1.70-1.59 (1H, m, H5); **^{13}C NMR** (100 MHz, $CDCl_3$) δ_C 152.7, 144.5, 137.4, 129.5,

128.9, 128.2, 128.2, 56.8, 53.4, 28.4, 24.1, 22.6, 21.7; **HRMS** (ES^{+}) calc. for $C_{15}H_{19}NO_4SNa$ $[M+Na]^+$ 332.0927, found 332.0921. The ee (76% ee) of (+)-**S33** was determined by HPLC analysis using CHIRALPAK® IC Column [250 x 4.6 mm ID; 10% *i*-PrOH in hexane; flow rate 2.0 mL/min; (+)-**S33** (major), t_R = 13.62 min, (–)-**S33** (minor); t_R = 15.17 min];

HPLC racemic sample for compound (±)-**S33**:

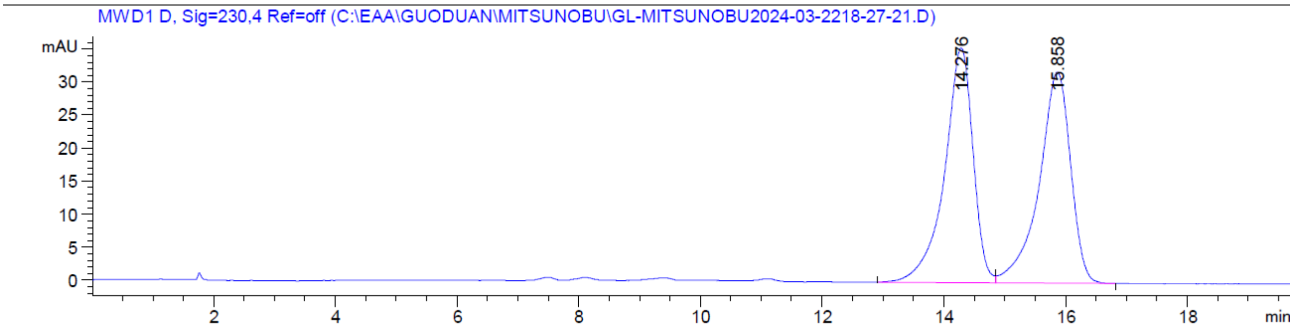

Peak #	RetTime [min]	Type	Width [min]	Area [mAU*s]	Height [mAU]	Area %
1	14.276	BV	0.4837	1161.29517	35.49670	50.2290
2	15.858	VB	0.5265	1150.70825	31.92862	49.7710

HPLC asymmetric sample for compound **(+)-S33**:

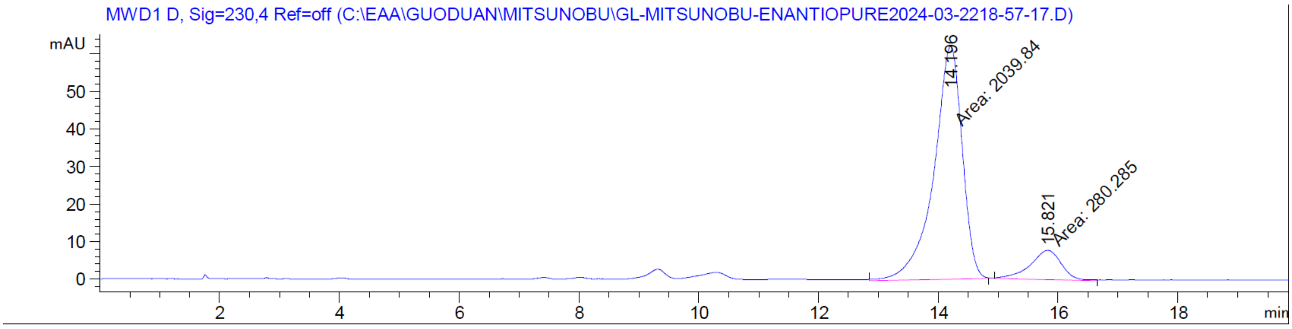

Peak #	RetTime [min]	Type	Width [min]	Area [mAU*s]	Height [mAU]	Area %
1	14.196	MM	0.5475	2039.83813	62.09267	87.9194
2	15.821	MM	0.6027	280.28452	7.75021	12.0806

_N_-(cyclohex-2-en-1-yl)-4-methylbenzenesulfonamide, (+)-38 with 76% ee

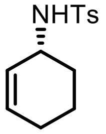

To a mixture of K_2CO_3 (14.3 mg, 0.10 mmol, 2.0 equiv.) in methanol (0.5 mL) was added a solution of **(+)-S33** (16 mg, 0.052 mmol, 1.0 equiv.) in methanol (0.5 mL) at 0 °C, the reaction mixture was allowed to warm up to room temperature, stirred for 3 h. The reaction was quenched with *sat*. NH_4Cl, mixture then was extracted with EtOAc (3 × 10 mL). The combined organic layers were dried over Na_2SO_4 and concentrated in vacuo to afford the title compound **(+)-38** (14 mg, 0.22 mmol, 100%, 75% ee) as yellow oil; **^{1}H NMR** (400 MHz, $CDCl_3$) δ_H 7.77 (d, *J* = 8.2 Hz, 1H), 7.30 (d, *J* = 8.0 Hz, 1H), 5.76 (dtd, *J* = 9.6, 3.7, 1.8 Hz, 1H), 5.34 (ddt, *J* = 10.0, 4.0, 2.2 Hz, 1H), 4.52 (d, *J* = 8.6 Hz, 1H), 3.81 (br, 1H), 2.43 (s, 3H), 1.98-1.84 (m, 2H), 1.80-1.70 (m, 1H), 1.65-1.49 (m, 3H); **^{13}C NMR** (100 MHz, $CDCl_3$) δ_C 143.4, 138.5, 131.7, 129.8, 127.2, 127.1, 49.1, 30.4, 24.6, 21.7, 19.4 ; Data identical to previous racemic sample.

(_R_)-_N_-(cyclohex-2-en-1-yl)-4-methylbenzenesulfonamide, (+)-38 with 95% ee

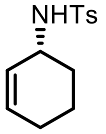

NaH (60% dispersion in mineral oil, 132 mg, 3.3 mmol, 2.2 equiv.) was added to the solution of $TsNH_2$ (514 mg, 3.0 mmol, 2.0 equiv.) in degassed THF (8.0 mL) at 0 °C. The mixture was then allowed to warm up to room temperature, stirring for 1 h. A solution of 3-cyclohexenyl benzoate **37** (303 mg, 1.5 mmol, 1.0 equiv.) in degassed THF (1.0 mL) was added to the above deprotonated sulfonamide reaction mixture, followed by adding a pre-stirring 15 min solution of (*S*, *S*)-Trost ligand (89.1 mg, 0.43 mmol, 0.086 equiv.) and $Pd_2(dba_3)$•$CHCl_3$ (37.3 mg, 0.036 mmol, 0.024 equiv.) in degassed THF (2.0 mL) and then the yellow mixture was stirred overnight at room temperature. The reaction was quenched with water (3.0 mL) and diluted with EtOAc (10.0 mL). The combined organic extracts were washed with brine, dried over Na_2SO_4 and concentrated. The residue was purified by flash column chromatography (pentane/EtOAc 10:1) to afford **(+)-38** (262 mg, 1.04 mmol, 70%, 95% ee) as yellow oil; and recovered benzoate (50 mg); $[\alpha]_D^{25}$ = +62.7 (c = 1.0, $CHCl_3$); **^{1}H NMR** (400 MHz, $CDCl_3$) δ_H 7.80 (d, *J* = 8.3 Hz, 1H), 7.32 (d, *J* = 7.9 Hz, 1H), 5.78 (dtd, *J* = 9.5, 3.7, 1.8 Hz, 1H), 5.36 (ddt, *J* = 10.0, 4.0, 2.2 Hz, 1H), 4.65 (d, *J* = 8.6 Hz, 1H), 3.83 (ttd, *J* = 8.5, 3.5, 1.7 Hz, 1H),

2.45 (s, 3H), 2.03-1.86 (m, 2H), 1.82-1.72 (m, 1H), 1.67-1.49 (m, 3H); **^{13}C NMR** (100 MHz, $CDCl_3$) δ_C 143.4, 138.5, 131.7, 129.8, 127.2, 127.1, 49.1, 30.4, 24.6, 21.7, 19.4; Data identical to previous racemic sample.

(1*R*,2*R*,6*R*)-2-iodo-7-tosyl-7-azabicyclo[4.1.0]heptane, (–)-36

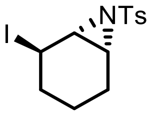

To a solution of **(+)-38** (502 mg, 2.0 mmol, 1.0 equiv.) in toluene (10.0 mL) was added KO*t*-Bu (449 mg, 4.0 mmol, 2.0 equiv.) at room temperature, stirring for 30 min. Then to this mixture was added I_2 (1.52 g, 6.0 mmol, 3.0 equiv.), stirring for 30 min. Quenching it with *sat*. $Na_2S_2O_3$ (5 mL), diluted with water and EtOAc. The organic phase was separated, and the aqueous phase extracted with EtOAc (20 x 3 mL). The combined organic extracts were washed with brine, dried over Na_2SO_4 and concentrated. The residue was purified by flash column chromatography (pentane/EtOAc 10:1) to afford **(–)-36** (385 mg, 0.51 mmol, 51%) as a yellow solid and recovered **(+)-38** (116 mg). The yield based on recovered starting material is 66%; $[\alpha]_D^{25}$ = –55.1 (c = 1.0, $CHCl_3$); **mp** 125-127 °C; **^{1}H NMR** (400 MHz, $CDCl_3$) δ_H 7.78 (d, *J* = 8.3 Hz, 2H), 7.34 (d, *J* = 8.0 Hz, 2H), 4.44 (ddd, *J* = 5.3, 4.3, 1.5 Hz, 1H), 3.45 (ddd, *J* = 6.8, 1.6, 0.8 Hz, 1H), 3.08 (ddd, *J* = 6.9, 5.8, 1.1 Hz, 1H), 2.44 (s, 3H), 1.99-1.90 (m, 1H), 1.86-1.76 (m, 2H), 1.75-1.66 (m, 1H), 1.63-1.50 (m, 1H), 1.43-1.35 (m, 1H); **^{13}C NMR** (100 MHz, $CDCl_3$) δ_C 144.7, 135.2, 129.9, 127.9, 46.1, 40.2, 29.9, 24.9, 21.8, 21.5, 17.7; Data identical to previous racemic sample.

(1*S*,6*R*)-7-tosyl-7-azabicyclo[4.1.0]hept-2-ene, (+)-8

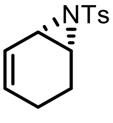

To a solution of **(–)-36** (180 mg, 0.534 mmol, 1.0 equiv.) in THF (5.3 mL) was added KO*t*-Bu (72 mg, 0.64 mmol, 1.2 equiv.) at room temperature, stirring for 30 min, then it was quenched with NH_4Cl (sat. aq.), diluted with water and EtOAc. The organic phase was separated, and the aqueous phase extracted with EtOAc (10 x 3 mL). The combined organic extracts were washed with brine, dried over Na_2SO_4 and concentrated. The residue was purified by flash column chromatography (pentane/EtOAc 15:1) to afford **(+)-8** (71 mg, 0.285 mmol, 53%, 95% ee) as a white solid, and recovered iodide (11 mg); The yield based on recovered starting material is 57%; **mp** 113-115 °C;

$[\alpha]_D^{25}$ = +83.2 (c = 1.0, $CHCl_3$); The ee (95% ee) of (+)-**8** was determined by HPLC analysis using Chiral column Lux 5 µm i-Amylose-1-00G-4762-E0 [250 x 4.6 mm ID; 3% *i*-PrOH in hexane; flow rate 1.5 mL/min; (–)-**8** (minor), t_R =11.97 min, (+)-**8** (major); t_R = 15.02 min]; **^{1}H NMR** (400 MHz, $CDCl_3$) δ_H 7.80 (d, *J* = 8.3 Hz, 1H), 7.31 (d, *J* = 8.0 Hz, 1H), 5.94-5.81 (m, 2H), 3.34-3.24 (m, 1H), 3.16 (ddd, *J* = 7.2, 4.3, 1.6 Hz, 1H), 2.42 (s, 3H), 2.10-2.03 (m, 1H), 2.02-1.97 (m, 2H), 1.56-1.45 (m, 1H); **^{13}C NMR** (100 MHz, $CDCl_3$) δ_C 144.3, 135.7, 133.6, 129.7, 127.8, 120.5, 41.7, 36.7, 21.7, 20.5, 18.7; Data identical to previous racemic sample.

HPLC racemic sample for compound **(±)-8**:

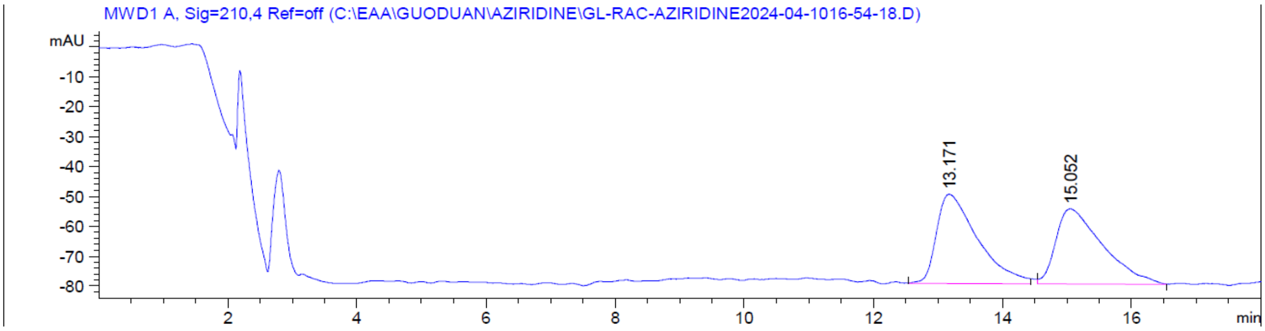

Signal 1: MWD1 A, Sig=210,4 Ref=off

Peak #	RetTime [min]	Type	Width [min]	Area [mAU*s]	Height [mAU]	Area %
1	13.171	BV	0.5345	1316.00378	29.84333	50.9894
2	15.052	VV	0.6042	1264.93396	25.13151	49.0106

HPLC asymmetric sample for compound **(–)-8**:

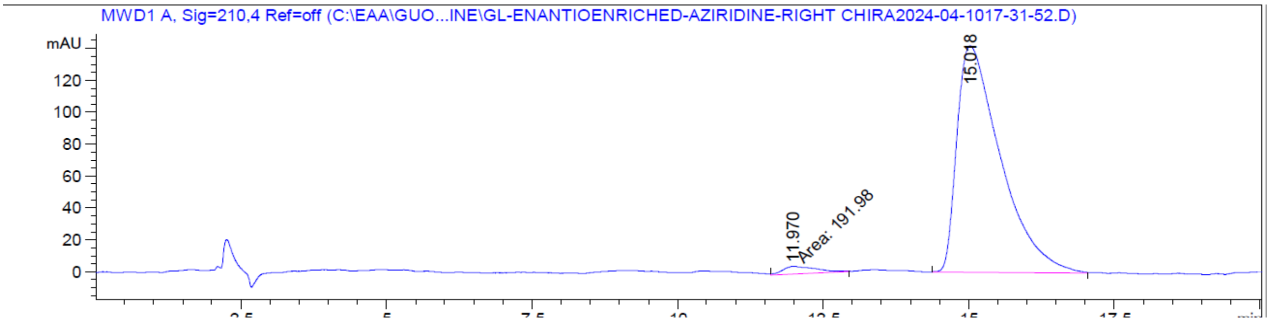

Peak #	RetTime [min]	Type	Width [min]	Area [mAU*s]	Height [mAU]	Area %
1	11.970	MM	0.6596	191.97997	4.85071	2.5248
2	15.018	VV	0.7025	7411.67383	141.87279	97.4752

(1*R*,6*S*)-7-tosyl-7-azabicyclo[4.1.0]hept-2-ene, (–)-8

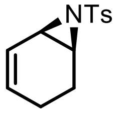

To a solution of **(+)-36** (54 mg, 0.16 mmol, 1.0 equiv.) in THF (1.6 mL) was added KO*t*-Bu (22 mg, 0.19 mmol, 1.2 equiv.) at room temperature, stirring for 30 min, then it was quenched with NH_4Cl (*sat. aq*.), diluted with water and EtOAc. The organic phase was separated, and the aqueous phase extracted with EtOAc. The combined organic extracts were washed with brine, dried over Na_2SO_4 and concentrated. The residue was purified by flash column chromatography (pentane/EtOAc 15:1) to afford **(–)-8** (16 mg, 64.3 µmol, 40%, 96.5% ee) as a white solid; and recovered iodide (6 mg). The ee (96.5% ee) of (–)-**8** was determined by HPLC analysis using Chiral column Lux 5 µm i-Amylose-1-00G-4762-E0 [250 x 4.6 mm ID; 5% *i*-PrOH in hexane; flow rate 1.0 mL/min; (–)-**8** (major), t_R =15.22 min, (+)-**8** (minor); t_R = 17.52 min]; $[\alpha]_D^{25}$ = –73.3 (c = 1.3, $CHCl_3$); **mp** 108-110 °C; NMR Data identical to previous racemic sample.

HPLC racemic sample for compound **(±)-8**:

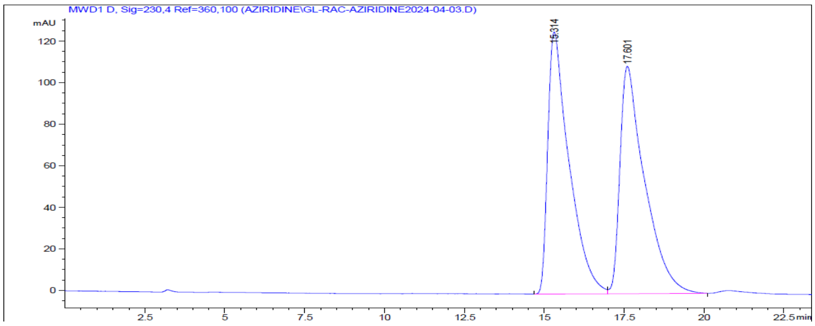

HPLC asymmetric sample for compound **(–)-8**:

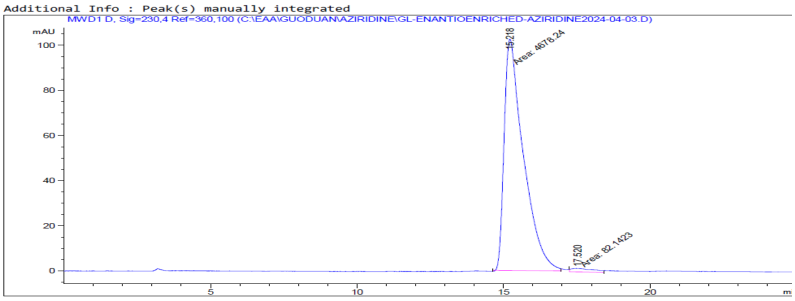

Peak #	RetTime [min]	Type	Width [min]	Area [mAU*s]	Height [mAU]	Area %
1	15.218	MM	0.7614	4678.24219	102.40559	98.2745
2	17.520	MM	0.8936	82.14230	1.53204	1.7255

***N*-((1*R*,2*R*)-2-((*tert*-butyldimethylsilyl)oxy)cyclohex-3-en-1-yl)-4-methylbenzenesulfonamide, (–)-9**

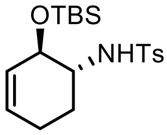

To a dry, N_2 flushed RBF was charged substrate (432 mg, 1.73 mmol, 1.0 equiv.) was added DMSO (4.0 mL) at 60 °C, stirring for overnight, confirmed by TLC, then reaction mixture was cooled to room temperature, diluting with water (5.0 mL), the layers were separated and the aqueous layer extracted with ethyl acetate (3 × 30 mL). The combined organic layers were dried over Na_2SO_4 and concentrated in vacuo to afford 535 mg enantioenriched compound **7**. It can go to next step without further purification.

To a solution of enone **7** (535 mg, 2.0 mmol, 1.0 equiv.) in MeOH (3 mL) was added $NaBH_4$ (92 mg, 2.4 mmol, 1.2 equiv.) at 0 °C, stirring for 30 min, quenching reaction with *sat.* NH_4Cl (2 mL), and the it was extracted with ether, then filtration was concentrated in vacuo to furnish the crude alcohol, the residue was used directly for next step without further purification.

To a solution of crude alcohol in DMF (4.0 mL) was added imidazole (204 mg, 3.0 mmol, 1.5 equiv.) and TBSCl (363 mg, 2.4 mmol, 1.2 equiv.) at room temperature, then the mixture was allowed to warm up to 40 °C stirring for 1 h. The reaction mixture was cooled down to room temperature, then it was quenched by the addition of 3 mL H_2O. The contents were then extracted with EtOAc (10 mL x 2), washed with brine, the residue upon workup was chromatographed on silica gel (pentane/EtOAc 15:1) with as eluent to give title **(–)-9** (424 mg, 1.10 mmol, 63%, 94.6% ee) as a white oil; $[\alpha]_D^{25}$ = –75.1 (c = 1.0, $CHCl_3$); The ee (94.6% ee) of (–)-**9** was determined by HPLC analysis using CHIRALPAK® IB Column [250 x 4.6 mm ID; 5% *i*-PrOH in hexane; flow rate 1.0 mL/min; (+)-**9** (minor), t_R = 5.27 min, (–)-**9** (major); t_R = 6.58 min]; ^{1}H NMR data matches the racemic sample.

HPLC racemic sample for compound **(±)-9**:

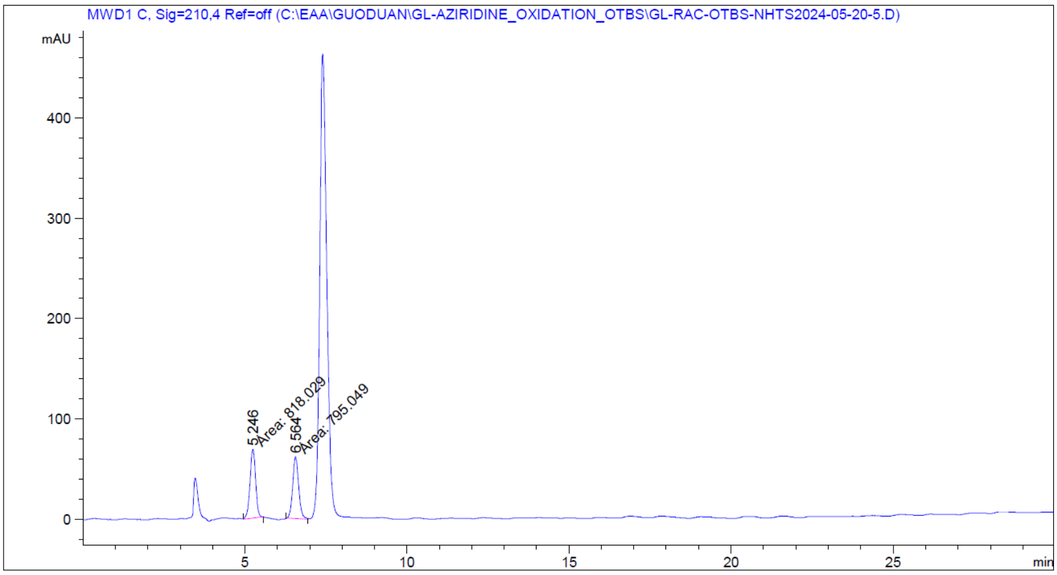

Signal 1: MWD1 C, Sig=210,4 Ref=off

Peak #	RetTime [min]	Type	Width [min]	Area [mAU*s]	Height [mAU]	Area %
1	5.246	MM	0.1988	818.02850	68.57004	50.7123
2	6.564	MM	0.2156	795.04858	61.47319	49.2877

HPLC asymmetric sample for **(–)-9**:

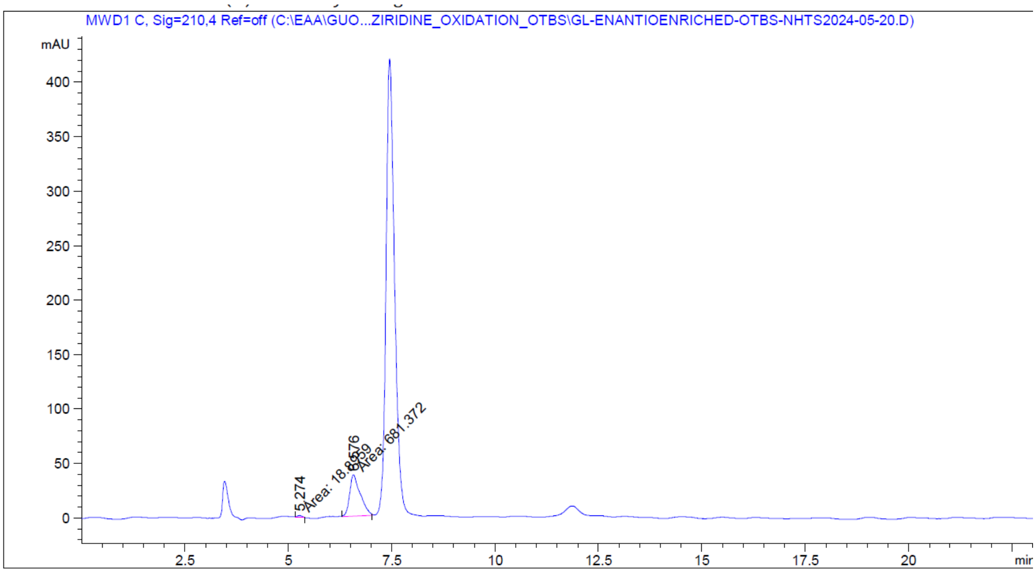

Peak #	RetTime [min]	Type	Width [min]	Area [mAU*s]	Height [mAU]	Area %
1	5.274	MM	0.1541	18.89590	2.04309	2.6984
2	6.576	MM	0.2972	681.37177	38.21394	97.3016

N-((1R,2R)-2-((tert-butyldimethylsilyl)oxy)cyclohex-3-en-1-yl)-4-methyl-N-((1-tosyl-1H-indol-3-yl)ethynyl)benzenesulfonamide, (−)-17

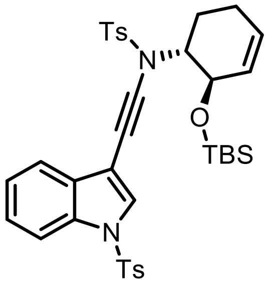

The above procedure (see Page S28) was repeated using enantioenriched **(−)-9** (364 mg, 0.94 mmol) and bromoalkyne **16** (982 mg, 2.6 mmol, 2.8 equiv.) as substrates, which afforded **(−)-17** (547 mg, 0.81 mmol) in 86% yield. ^{1}H NMR data matches the racemic sample; $[\alpha]_D^{25}$ = −121.6 (c = 1.0, $CHCl_3$).

(±)-3-{(E)-[(1S,5R,8S)-8-[(Tert-Butyldimethylsilyl)oxy]-6-tosyl-6-azabicyclo[3.2.1]oct-2-en-7-ylidene)methyl}-1-tosyl-1H-indole, (+)-18

The above procedure (see Page S29) was repeated using enantioenriched **(−)-17** (617 mg, 0.91 mmol) as substrate, which afforded **(+)-18** (469 mg, 0.69 mmol) in 76% yield as ***Z*** and ***E*** hardly separable mixture isomers as a green foam;

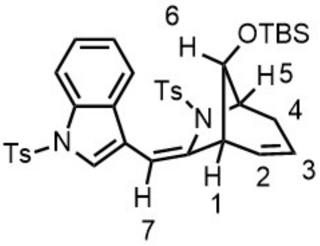

Z isomer: $[\alpha]_D^{25}$ = +102.7 (c = 1.0, $CHCl_3$); ^{1}H NMR data matches the racemic sample.

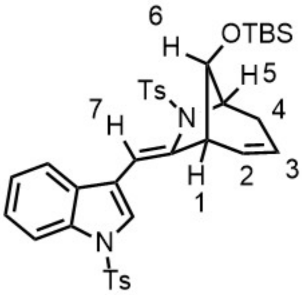

E isomer: $[\alpha]_D^{25}$ = +12.1 (c = 0.55, $CHCl_3$); ^{1}H NMR data matches the racemic sample.

N-((1R,5R,6R)-6-((tert-butyldimethylsilyl)oxy)-5-(2-(1-tosyl-1H-indol-3-yl)acetyl)cyclohex-3-en-1-yl)-4-methylbenzenesulfonamide, (+)-23

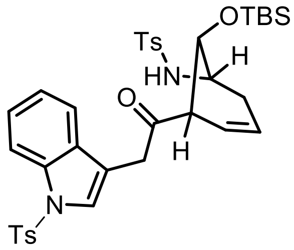

The above procedure (see Page S37) was repeated using enantioenriched (*Z*, *E*)-mixture of **(+)-18** (451 mg, 0.667 mmol) as substrate, which afforded **(+)-23** (365 mg, 0.527 mmol,) in 81% yield. ^{1}H NMR data matches the racemic sample; $[\alpha]_D^{25}$ = +52.2 (c = 1.0, $CHCl_3$).

N-((1R,5S,6R)-6-((Tert-butyldimethylsilyl)oxy)-5-((S)-1-hydroxy-2-(1-tosyl-1H-indol-3-yl)ethyl)cyclohex-3-en-1-yl)-4-methylbenzenesulfonamide, (+)-24a

N-((1S,5R,6S)-6-((Tert-butyldimethylsilyl)oxy)-5-((S)-1-hydroxy-2-(1-tosyl-1H-indol-3-yl)ethyl)cyclohex-3-en-1-yl)-4-methylbenzenesulfonamide, (+)-24

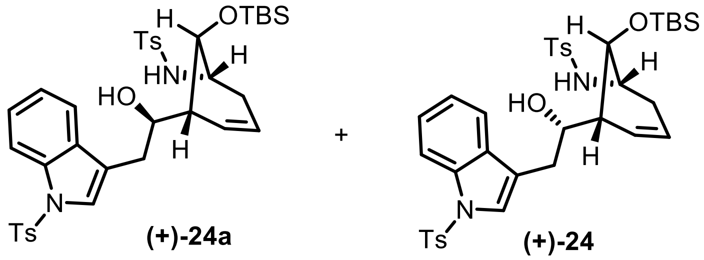

The above procedure (see Page S38) was repeated using enantioenriched **(+)-23** (355 mg, 0.513 mmol, 1.0 equiv.) as substrate, which afforded **(+)-24a** (100 mg, 0.144 mmol) in 28% yield; ^{1}H NMR data matches the racemic sample; $[\alpha]_D^{25}$ = +59.5 (c = 1.0, $CHCl_3$); and afforded **(+)-24** (249 mg, 0.359 mmol) in 70% yield and 95.1% ee; $[\alpha]_D^{25}$ = +47.2 (c = 1.0, $CHCl_3$); The *ee* (95.1% ee) of **(+)-24** was determined by HPLC analysis using CHIRALPAK® IC Column [250 x 4.6 mm ID; 20% EtOH in hexane; flow rate 1.0 mL/min; (–)-**24** (minor), t_R = 9.02 min, (+)-**24** (major); t_R = 9.75 min];^{1}H NMR data matches the racemic sample;

HPLC racemic sample for compound **(±)-24**:

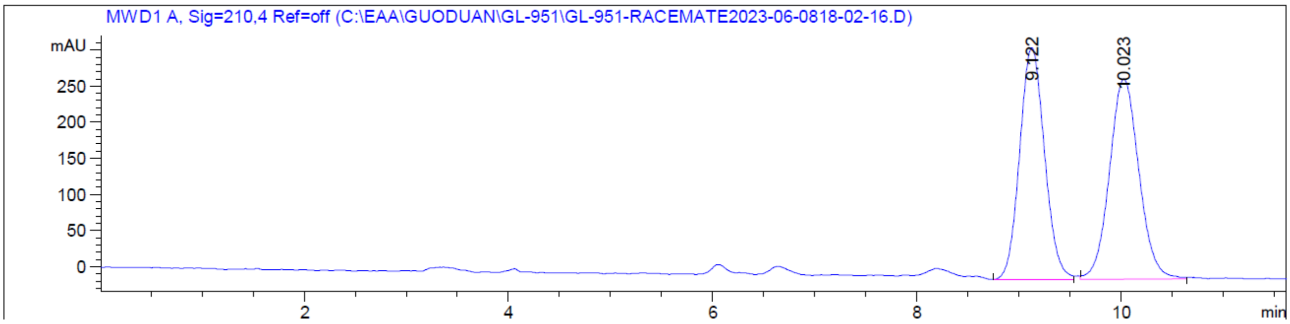

Peak #	RetTime [min]	Type	Width [min]	Area [mAU*s]	Height [mAU]	Area %
1	9.122	BV	0.2675	5442.08936	321.49277	49.3757
2	10.023	VV	0.3103	5579.71289	277.84039	50.6243

HPLC asymmetric sample compound **(+)-24**:

Additional Info : Peak(s) manually integrated

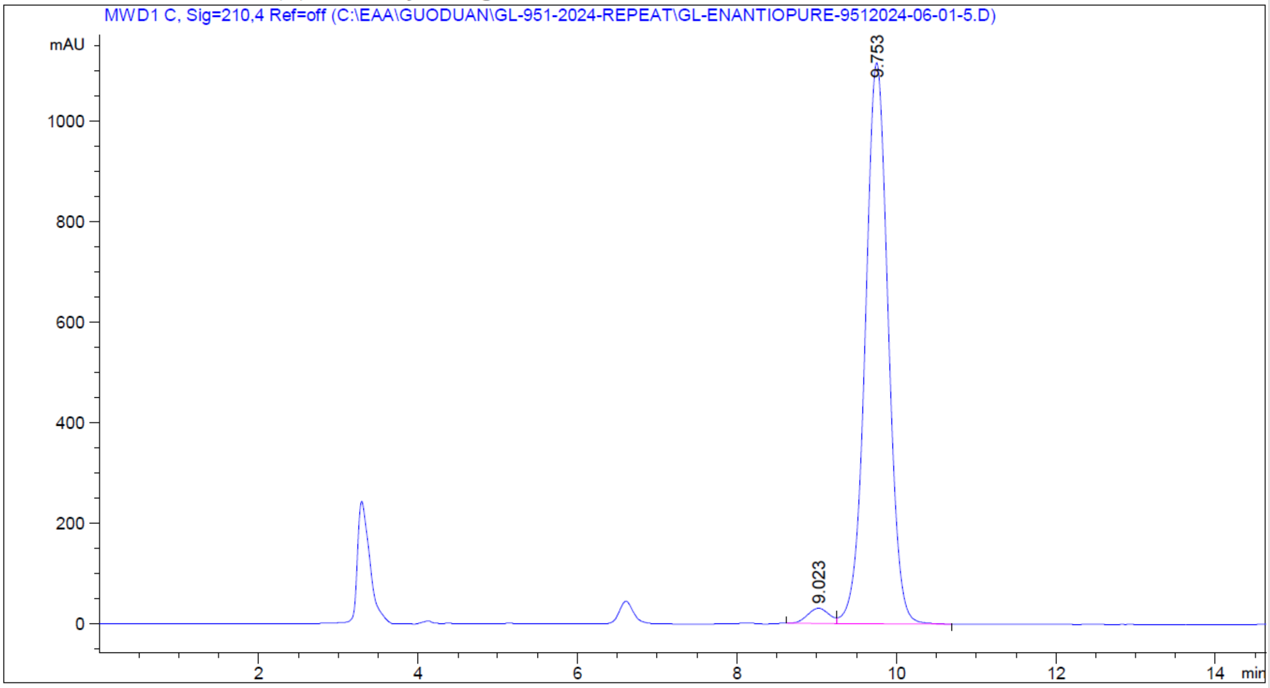

Peak #	RetTime [min]	Type	Width [min]	Area [mAU*s]	Height [mAU]	Area %
1	9.023	BV	0.2866	566.52618	30.23441	2.4910
2	9.753	VV	0.3058	2.21767e4	1116.16040	97.5090

3-(((1*R*,5*R*,7*R*,8*R*)-8-((*tert*-butyldimethylsilyl)oxy)-6-tosyl-6-azabicyclo[3.2.1]oct-2-en-7-yl)methyl)-1-tosyl-1*H*-indole, (–)-25

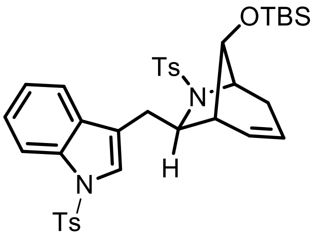

The above procedure (see Page S39) was repeated using enantioenriched **(+)-24** (196 mg, 0.282 mmol) as substrate, which afforded **(–)-25** (181 mg, 0.268 mmol) in 95% yield and 97% ee; ^{1}H NMR data matches the racemic sample; $[\alpha]_D^{25}$ = –31.2 (c = 1.0, $CHCl_3$); The *ee* (97% ee) of (–)-**25** was determined by HPLC analysis using CHIRALPAK® IC Column [250 x 4.6 mm ID; 10% EtOH in hexane; flow rate 1.0 mL/min; (–)-**25** (major), t_R = 14.26 min, (+)-**25** (minor); t_R = 17.41 min].

HPLC racemic sample for **(±)-25**:

Additional Info : Peak(s) manually integrated

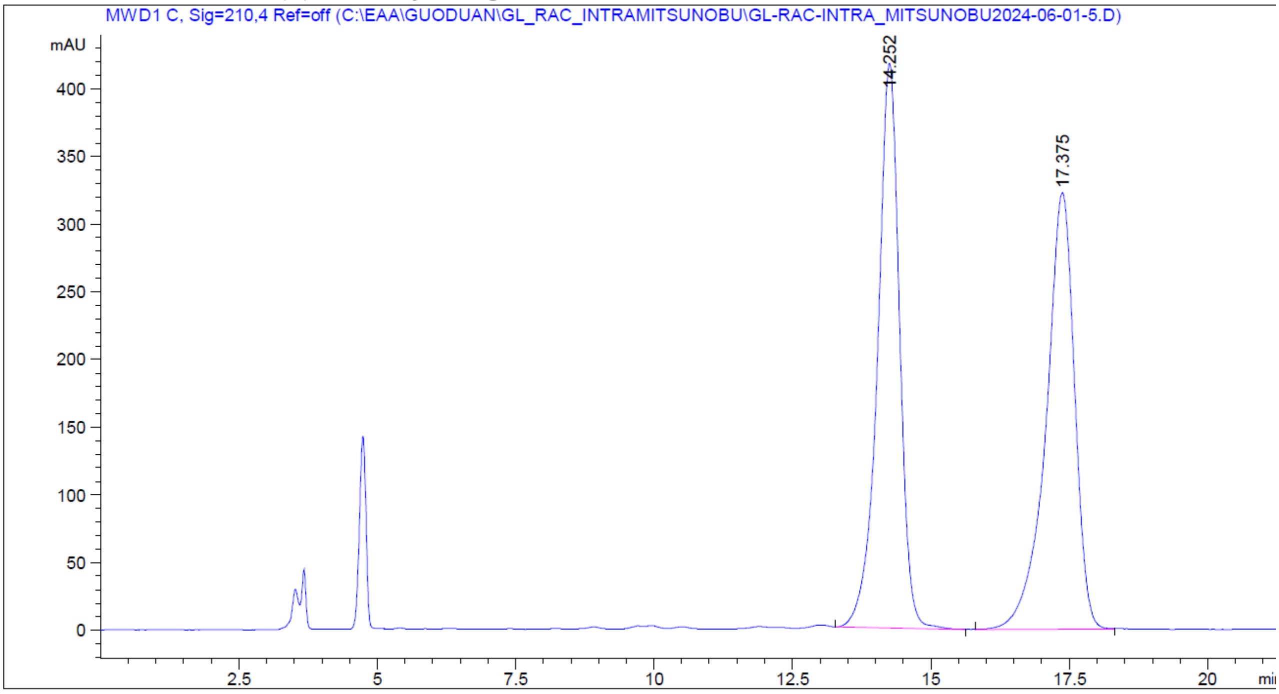

Peak #	RetTime [min]	Type	Width [min]	Area [mAU*s]	Height [mAU]	Area %
1	14.252	BB	0.4157	1.14981e4	417.02997	49.8442
2	17.375	VV	0.5347	1.15700e4	322.40228	50.1558

HPLC asymmetric sample **(–)-25**:

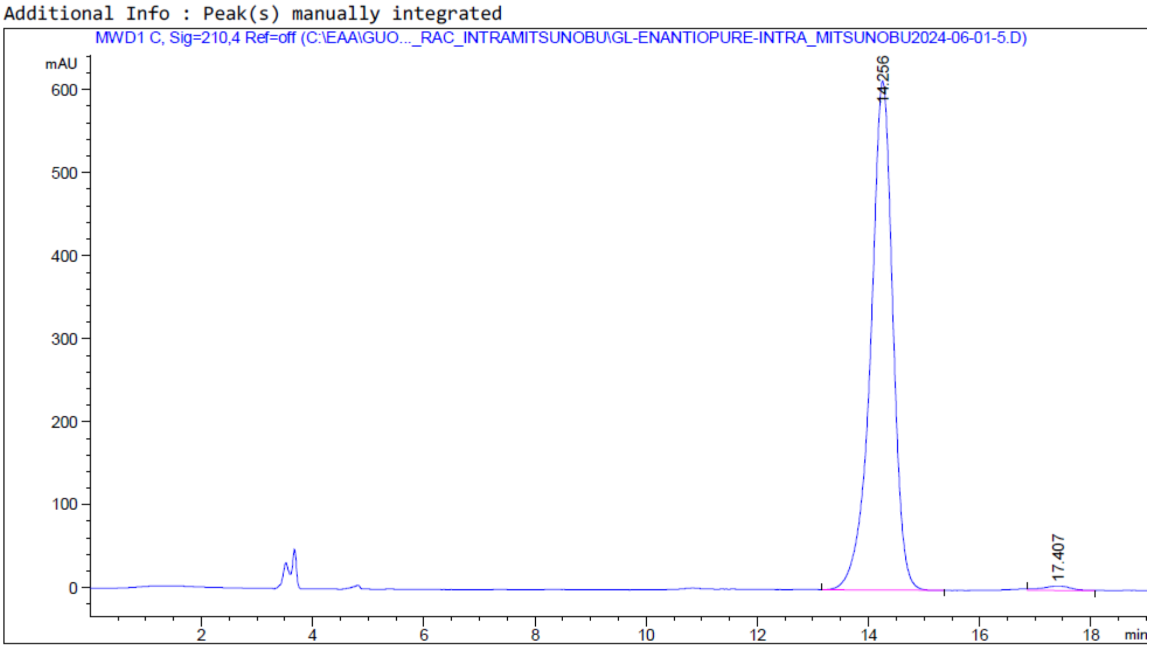

Peak #	RetTime [min]	Type	Width [min]	Area [mAU*s]	Height [mAU]	Area %
1	14.256	VB	0.4167	1.69617e4	613.21332	98.8490
2	17.407	VB	0.4301	197.50504	5.64638	1.1510

(1*R*,5*R*,7*R*,8*R*)-6-tosyl-7-((1-tosyl-1*H*-indol-3-yl)methyl)-6-azabicyclo[3.2.1]oct-2-en-8-ol, (–)-26

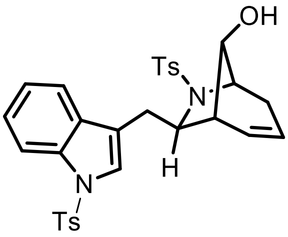

The above procedure (see Page S41) was repeated using enantioenriched **(–)-25** (174 mg, 0.257 mmol) as substrate, which afforded **(–)-26** (144 mg, 0.256 mmol) in 99% yield; $[\alpha]_D^{25}$ = –38.5 (c = 1.0, $CHCl_3$); ^{1}H NMR data matches the racemic sample.

(1*R*,5*R*,7*R*,8*R*)-7-((1*H*-indol-3-yl)methyl)-6-azabicyclo[3.2.1]oct-2-en-8-ol, (+)-39

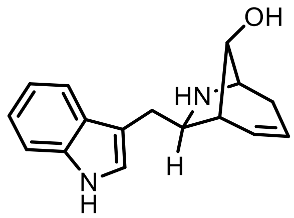

To a solution of **(–)-26** (149 mg, 0.265 mmol, 1.0 equiv.) in THF (3.0 mL), Na/nap solution (0.2 M, 7.0 mL, 1.4 mmol, 5.28 equiv.) was added at -78 °C until reaction solution become dark green. And stirred at the same temperature until consumption of starting material (roughly 1 h). Then, the reaction mixture was quenched with *Sat*. NH_4Cl *aq*. The remnant aqueous layer was extracted with EtOAc, and organic layers were combined and dried over Na_2SO_4. Silica gel flash chromatography (DCM/MeOH 15:1) gave the title compound **(+)-39** as a white foam (62 mg, 0.244 mmol, 93%); **R_f** 0.11 (DCM/MeOH 10:1); $[\alpha]_D^{25}$ = +57.9 (c = 1.0, MeOH); **IR** (thin film, ν_{max} / cm^{-1}) 3415, 3327, 3063, 3053, 2918, 2358, 2342, 1618, 1491, 1457, 1340, 1271, 1230, 1217, 1009, 744, 690; **^{1}H NMR** (400 MHz, $CDCl_3$) δ_H 8.23 (1H, s, N*H*), 7.64-7.57 (1H, m, Ar*H*), 7.34 (1H, dt, *J* = 8.1, 0.9 Hz, Ar*H*), 7.19 (1H, ddd, *J* = 8.1, 7.0, 1.2 Hz, Ar*H*), 7.11 (1H, ddd, *J* = 8.0, 7.0, 1.1 Hz, Ar*H*), 7.03-6.95 (1H, m, Ar*H*), 5.76 (2H, d, *J* = 4.1 Hz, H2 and H3), 4.53 (1H, t, *J* = 4.8 Hz, H6), 3.69 (1H, t, *J* = 7.6 Hz, H7), 3.40-3.31 (1H, m, H5), 2.92-2.81 (2H, m, H8), 2.54-2.47 (1H, m, H4), 2.45 (1H, dd, *J* = 4.5, 2.5 Hz, H1), 2.04-1.93 (1H, m, 3H); **^{13}C NMR** (100 MHz, $CDCl_3$) δ_C 136.4, 129.2, 127.6, 127.5, 122.3, 122.2, 119.5, 118.9, 113.6, 111.3, 70.0, 66.6, 54.8, 43.2, 34.1, 33.6; **HRMS** (ES^+) calc. for $C_{16}H_{19}N_2O$ $[M+H]^+$ 255.1492, found 255.1492.

(1*R*,5*R*,7*R*,8*R*)-7-((1*H*-indol-3-yl)methyl)-6-isopropyl-6-azabicyclo[3.2.1]oct-2-en-8-ol, (+)-40

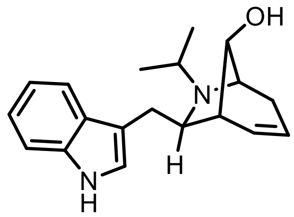

To a solution of aminol alcohol **(+)-39** (35 mg, 0.138 mmol, 1.0 equiv.) in MeCN (1.5 mL) was added acetone (80 mg, 1.38 mmol, 10.0 equiv.) and $NaBH(OAc)_3$ (88 mg, 0.414 mmol, 3.0 equiv.), stirring at 50 °C until completion of substrate (confirmed by TLC). Then, the reaction mixture was quenched with *sat.* NH_4Cl (1.0 mL). The remnant aqueous layer was extracted with ether (10 mL x 2), and organic layers were combined and washed with Brine (5 mL x 2) dried over Na_2SO_4, concentrating

afford **(+)-40** (39 mg, 0.132 mmol, 96%) as a yellow oil without further purification; **R$_f$** 0.17 (DCM/MeOH 15:1); $[\alpha]_D^{25}$ = +18.1 (c = 0.63, MeOH); **IR** (thin film, ν_{max} / cm^{-1}) 3413, 2972, 2926, 2360, 2249, 1456, 1233, 1177, 1073, 910, 740, 648; **^{1}H NMR** (400 MHz, $CDCl_3$) δ_H 8.13 (1H, br, N*H*), 7.61 (1H, dt, *J* = 7.8, 0.9 Hz, Ar*H*), 7.35 (1H, dt, *J* = 8.1, 1.0 Hz, Ar*H*), 7.20 (1H, ddd, *J* = 8.2, 7.0, 1.2 Hz, Ar*H*), 7.13 (1H, ddd, *J* = 8.0, 7.0, 1.1 Hz, Ar*H*), 6.97 (1H, d, *J* = 2.2 Hz, Ar*H*), 5.73 (1H, dq, *J* = 7.9, 1.9 Hz, H2), 5.67-5.57 (1H, m, H3), 4.70-4.56 (1H, m, H6), 3.57 (1H, td, *J* = 3.5, 1.7 Hz, H5), 3.24 (1H, dd, *J* = 11.2, 3.2 Hz, H7), 3.06 (1H, p, *J* = 6.2 Hz, H9), 2.97-2.89 (1H, m, H8), 2.82 (1H, dd, *J* = 14.9, 11.2 Hz, H8), 2.52-2.46 (1H, m, H4), 2.46-2.42 (1H, m, H1), 2.23-2.15 (1H, m, H4), 1.33 (3H, d, *J* = 6.3 Hz, CHC*H*$_3$), 1.18 (1H, d, *J* = 6.1 Hz, CHC*H*$_3$); **^{13}C NMR** (100 MHz, $CDCl_3$) δ_C 136.3, 129.7, 127.8, 127.7, 122.1, 121.9, 119.4, 119.2, 114.7, 111.2, 70.1, 69.7, 58.3, 50.2, 43.2, 34.8, 24.7, 23.7, 23.3; **HRMS** (ES$^+$) calc. for $C_{19}H_{24}N_2ONa$ [M+Na]$^+$ 319.1781, found 319.1775.

3-{[(1*S*,5*R*,7*R*)-6-isopropyl-8-methylene-6-azabicyclo[3.2.1]oct-2-en-7-yl]methyl}-1*H*-indole, (–)-41

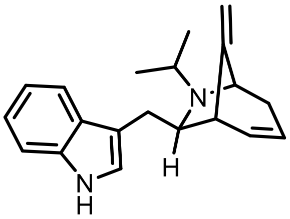

Dimethyl sulfide (0.04 mL, 555 μmol, 15.0 equiv.) was added to a cold (0 °C) solution of *N*-chlorosuccinimide (50 mg, 372 μmol, 10.0 equiv.) in toluene/dichloromethane mixture (total 1.5 mL; 2:1) under an argon atmosphere. After 5 minutes, the resulting suspension was cooled down to -78 °C and stirred for additional 10 minutes. A solution of alcohol **(+)-39** (11 mg, 37.2 μmol, 1.0 equiv.) in toluene/dichloromethane mixture (total 2.0 mL; 2:1) was added dropwise and the reaction mixture was allowed to reach -50 °C during 30 minutes. Triethylamine (0.08 mL, 555 μmol, 15.0 equiv.) was added and the reaction mixture was stirred 15 minutes at -50 °C. MeOH (0.2 mL) was added to quench the reaction and the mixture was partitioned between ethyl acetate and brine. The organic extract was dried over anhydrous Na_2SO_4, filtered and concentrated to furnish the crude ketone ; the residue was used directly for next step without further purification.

To a cooled (-45 °C) solution of above obtained crude ketone (10 mg, 34.0 μmol, 1.0 equiv.) in THF (1.2 mL) was added freshly prepared Tebbe reagent[21] (3 days old, 1.0 M in toluene, 0.15 mL, 0.136

mmol, 4.0 equiv.). The reaction mixture was maintained at -45 °C for 30 min and then slowly warmed to 0 °C stirring for 2.5 h. The reaction mixture was warmed to 23 °C and stirred for 3 h. The solution was diluted with 1 mL of THF and treated with 15% aq. NaOH (0.2 mL) After being stirred for 1 h, the heterogeneous mixture was filtered through celite, and the precipitate was washed with Et_2O. The filtrates were combined and concentrated in vacuo. Purification by flash chromatography (pentane/EtOAc 3:1) provided **(–)-41** (3.5 mg, 12.0 μmol, 32.4%) as a white oil; **R_f** 0.21 (pentane/EtOAc 3:1); $[\alpha]_D^{25}$ = –4.6 (c = 0.13, MeOH); **IR** (thin film, ν_{max} / cm^{-1}) 3420, 2976, 2926, 2247, 1686, 1456, 1354, 1309, 1227, 1176, 1154, 1095, 910, 740, 643; **^{1}H NMR** (400 MHz, $CDCl_3$) δ_H 7.98 (1H, br, N*H*), 7.61 (1H, dd, *J* = 7.8, 1.2 Hz, Ar*H*), 7.37 (1H, dt, *J* = 8.1, 0.9 Hz, Ar*H*), 7.20 (1H, ddd, *J* = 8.1, 7.0, 1.3 Hz, Ar*H*), 7.12 (1H, ddd, *J* = 8.0, 7.0, 1.1 Hz, Ar*H*), 6.98 (1H, br, Ar*H*), 5.81-5.77 (1H, m, H2), 5.49-5.43 (1H, m, H3), 5.02 (1H, s, H6), 4.84 (1H, s, H6), 3.91 (1H, s, H5), 3.29 (1H, dd, *J* = 11.2, 3.1 Hz, H7), 3.11-2.99 (1H, m, H9), 2.86-2.78 (1H, m, H8), 2.74-2.68 (1H, m, H4), 2.68-2.64 (1H, m, H8), 2.60 (1H, d, *J* = 6.6 Hz, H1), 2.28-2.20 (1H, m, H4), 1.34 (3H, d, *J* = 6.2 Hz, CHC*H*3), 1.17 (3H, d, *J* = 6.0 Hz, CHC*H*3); **^{13}C NMR** (100 MHz, $CDCl_3$) δ_C 151.0, 136.3, 134.2, 128.0, 125.1, 122.1, 121.6, 119.5, 119.3, 115.1, 111.2, 101.6, 71.2, 61.3, 50.2, 45.4, 34.6, 32.5, 24.1, 23.9; **HRMS** (ES^+) calc. for $C_{20}H_{25}N_2$ $[M+H]^+$ 293.2012, found 293.2008.

8. X-ray crystallographic data

Crystals were grown by diffusion. An uncapped small vial which contained a solution of the compound (~50 mg) in CH_2Cl_2 (2 mL) was placed in a larger jar containing pentane (10 mL). The jar was sealed to allow diffusion of the solvents over 1-2 weeks at ~22 °C until crystals appeared.

Low temperature single crystal X-ray diffraction data were collected using a (Rigaku) Oxford Diffraction SuperNova diffractometer for **19** and a Rigaku XtaLAB Synergy-DW for **8**, **26**, **32** and **S40**. Raw frame data were reduced using CrysAlisPro and the structures were solved using 'Superflip' [L. Palatinus, G. Chapuis, *J. Appl. Cryst.* **2007**, *40*, 786-790] before refinement with CRYSTALS [a) P. Parois, R.I. Cooper, A.L. Thompson, *Chem. Cent. J.*, **2015**, 9:30; b) R.I. Cooper, A.L. Thompson, D.J. Watkin, *J. Appl. Cryst.* **2010**, *43*, 1100-1107] as per the SI (CIF). X-ray crystallographic data (.cif). CCDC 2427507–2427511 contains the supplementary crystallographic data for this paper. In the images below and manuscript, all ellipsoids are shown at 50% probability. These data can be obtained free of charge via www.ccdc.cam.ac.uk/data_request/cif, or by emailing data_request@ccdc.cam.ac.uk, or by contacting The Cambridge Crystallographic Data Centre, 12 Union Road, Cambridge CB2 1EZ, U.K..

X-ray data for compound **(+)-8**

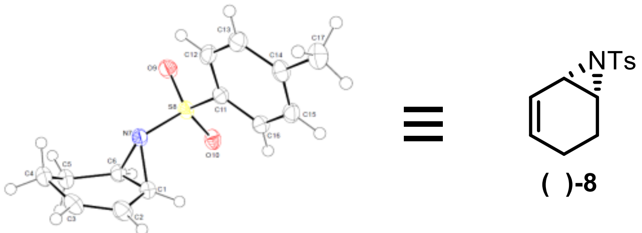

( )-8

Table 8.1. Crystal data and structure refinement for compound (+)-8

CCDC	2427507	
Identification code	7848	
Empirical formula	C13 H15 N O2 S	
Formula weight	249.33	
Temperature	100 K	
Wavelength	1.54184 Å	
Crystal system	Monoclinic	
Space group	P 2_1	
Unit cell dimensions	a = 8.02470(10) Å	α= 90°.
	b = 5.79580(10) Å	β= 103.3918(11)°.
	c = 13.54080(10) Å	γ= 90°.
Volume	612.652(14) $Å^3$	
Z	2	
Density (calculated)	1.352 Mg/m3	
Absorption coefficient	2.262 mm^{-1}	
F(000)	264	
Crystal size	0.14 x 0.11 x 0.10 mm3	
Theta range for data collection	3.355 to 75.854°	
Index ranges	-9<=h<=10, -7<=k<=7, -16<=l<=16	
Reflections collected	22214	
Independent reflections	2522 [R(int) = 0.033]	
Completeness to theta = 74.337°	99.9 %	
Absorption correction	Semi-empirical from equivalents	
Max. and min. transmission	0.80 and 0.73	
Refinement method	Full-matrix least-squares on F^2	
Data / restraints / parameters	2521 / 1 / 155	
Goodness-of-fit on F^2	1.0065	
Final R indices [I>2sigma(I)]	R1 = 0.0311, wR2 = 0.0811	
R indices (all data)	R1 = 0.0312, wR2 = 0.0812	
Absolute structure parameter	0.014(5)	
Largest diff. peak and hole	0.06 and -0.09 e.$Å^{-3}$	

X-ray data for compound **(±)-19**

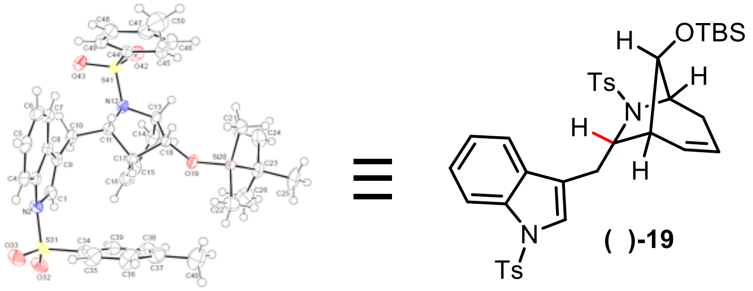

Table 8.2. Crystal data and structure refinement for compound (±)-19

CCDC	2427508
Empirical formula	C36 H44 N2 O5 S2 Si
Formula weight	676.97
Temperature	150 K
Wavelength	1.54184 Å
Crystal system	Triclinic
Space group	P -1
Unit cell dimensions	a = 10.1569(2) Å α= 93.6775(17)°
	b = 11.6700(3) Å β= 98.8933(17)°
	c = 15.1901(3) Å γ = 94.6050(18)°
Volume	1767.74(7) $Å^3$
Z	2
Density (calculated)	1.272 Mg/m^3
Absorption coefficient	2.041 mm^{-1}
F(000)	720
Crystal size	0.20 x 0.12 x 0.10 mm^3
Theta range for data collection	3.812 to 76.392°
Index ranges	-11<=h<=12, -14<=k<=14, -18<=l<=19
Reflections collected	42482
Independent reflections	7345 [R(int) = 0.030]
Completeness to theta = 74.100°	99.7 %
Absorption correction	Semi-empirical from equivalents
Max. and min. transmission	0.82 and 0.69
Refinement method	Full-matrix least-squares on F^2
Data / restraints / parameters	7345 / 0 / 415
Goodness-of-fit on F^2	0.9924
Final R indices [I>2sigma(I)]	R1 = 0.0308, wR2 = 0.0837
R indices (all data)	R1 = 0.0336, wR2 = 0.0865
Largest diff. peak and hole	0.36 and -0.40 e.$Å^{-3}$

X-ray data for compound for **(±)-26**

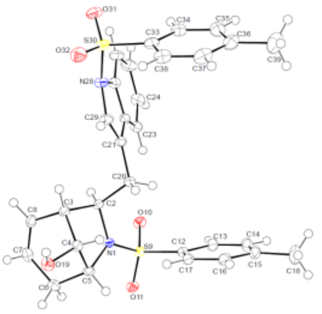

≡

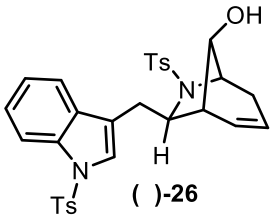

Table 8.3. Crystal data and structure refinement for compound (±)-26

CCDC	2427509	
Empirical formula	C30 H30 N2 O5 S2	
Formula weight	562.71	
Temperature	100 K	
Wavelength	1.54184 Å	
Crystal system	Monoclinic	
Space group	P 2_1/c	
Unit cell dimensions	a = 18.44320(10) Å	α= 90°
	b =13.53090(10) Å	β= 98.6638(3)°
	c = 10.93140(10) Å	γ = 90°
Volume	2696.84(3) $Å^3$	
Z	4	
Density (calculated)	1.386 Mg/m^3	
Absorption coefficient	2.154 mm^{-1}	
F(000)	1184	
Crystal size	0.15 x 0.07 x 0.05 mm^3	
Theta range for data collection	2.423 to 75.939°	
Index ranges	-23<=h<=23, -16<=k<=16, -13<=l<=13	
Reflections collected	121101	
Independent reflections	5569 [R(int) = 0.023]	
Completeness to theta = 74.420°	99.6 %	
Absorption correction	Semi-empirical from equivalents	
Max. and min. transmission	0.90 and 0.76	
Refinement method	Full-matrix least-squares on F^2	
Data / restraints / parameters	5568 / 0 / 353	
Goodness-of-fit on F^2	1.0015	
Final R indices [I>2sigma(I)]	R1 = 0.0284, wR2 = 0.0749	
R indices (all data)	R1 = 0.0288, wR2 = 0.0751	
Largest diff. peak and hole	0.35 and -0.36 e.$Å^{-3}$	

X-ray data for compound **(±)-32**

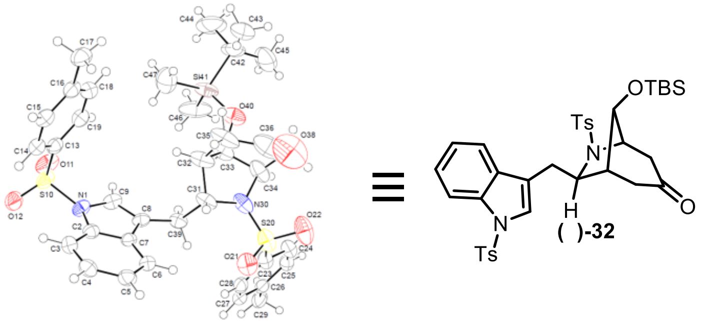

Table 8.4. Crystal data and structure refinement for compound (±)-32

CCDC	2427510	
Empirical formula	C36 H44 N2 O6 S2 Si	
Formula weight	692.97	
Temperature	100 K	
Wavelength	1.54184 Å	
Crystal system	Monoclinic	
Space group	P 2_1	
Unit cell dimensions	a = 8.13710(10) Å	α = 90°
	b = 11.48300(10) Å	β = 101.4337(9)°
	c =19.4059(2) Å	γ = 90°
Volume	1777.27(3) $Å^3$	
Z	2	
Density (calculated)	1.295 Mg/m^3	
Absorption coefficient	2.064 mm^{-1}	
F(000)	735.997	
Crystal size	0.15 x 0.03 x 0.02 mm^3	
Theta range for data collection	2.323 to 75.920°	
Index ranges	-10<=h<=10, -14<=k<=14, -24<=l<=22	
Reflections collected	73491	
Independent reflections	7290 [R(int) = 0.036]	
Completeness to theta = 74.065°	99.9 %	
Absorption correction	Semi-empirical from equivalents	
Max. and min. transmission	0.96 and 0.81	
Refinement method	Full-matrix least-squares on F^2	
Data / restraints / parameters	7290 / 400 / 501	
Goodness-of-fit on F^2	0.9967	
Final R indices [I>2sigma(I)]	R1 = 0.0539, wR2 = 0.1485	
R indices (all data)	R1 = 0.0560, wR2 = 0.1508	
Absolute structure parameter	0.010(3)	
Extinction coefficient	16(4)	
Largest diff. peak and hole	0.66 and -0.48 e.$Å^{-3}$	

X-ray data for compound **(±)-S40**

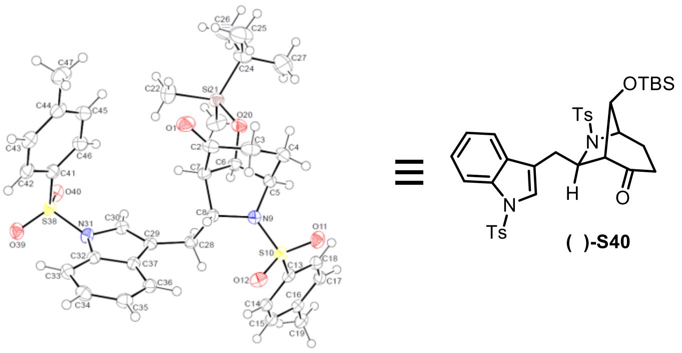

Table 8.5. Crystal data and structure refinement for compound (±)-S40

CCDC	2427511	
Empirical formula	C36 H44 N2 O6 S2 Si	
Formula weight	692.97	
Temperature	100 K	
Wavelength	1.54184 Å	
Crystal system	Monoclinic	
Space group	P 2_1	
Unit cell dimensions	a = 8.15460(10) Å	α = 90°
	b = 11.37120(10) Å	β = 100.3795(8)°
	c =19.61720(10) Å	γ = 90°
Volume	1789.29(3) $Å^3$	
Z	2	
Density (calculated)	1.286 Mg/m^3	
Absorption coefficient	2.051 mm-1	
F(000)	736	
Crystal size	0.18 x 0.16 x 0.01 mm^3	
Theta range for data collection	2.290 to 75.576°	
Index ranges	-10<=h<=10, -14<=k<=14, -24<=l<=22	
Reflections collected	44681	
Independent reflections 7307	[R(int) = 0.041]	
Completeness to theta = 74.065°	99.7 %	
Absorption correction	Semi-empirical from equivalents	
Max. and min. transmission	0.98 and 0.66	
Refinement method	Full-matrix least-squares on F2	
Data / restraints / parameters	7307 / 1 / 426	
Goodness-of-fit on F2	1.0042	
Final R indices [I>2sigma(I)]	R1 = 0.0348, wR2 = 0.0919	
R indices (all data)	R1 = 0.0356, wR2 = 0.0924	
Absolute structure parameter	0.011(5)	
Extinction coefficient	16(4)	
Largest diff. peak and hole	0.30 and -0.28 e.$Å^{-3}$	

9. References

1. S. D. Burke, M. E. Kort, S. M. S. Strickland, H. M. Organ, L. A. Silks III, *Tetrahedron Lett.* **1994**, *35*, 1503−1506.
2. M. Shimano, H. Nagaoka, Y. Yamada, *Chem. Parm. Bull* **1990**, *38*, 276−278.
3. Y. Hu, C. Gu, X. Wang, L. Min, C. Li, *J. Am. Chem. Soc.* **2021**, *143*, 17862–17870.
4. M. L. Shrestha, W. Qi, M. C. McIntosh, *J. Org. Chem.* **2017**, *82*, 8359−8370.
5. A. G. Myers, B. Zheng, *Tetrahedron Lett*. **1996**, *37*, 4841−4844.
6. P. Wang, J. Chen, W. He, J. Song, H. Song, H. Wei, W. Xie, *Org. Lett*. **2021**, *23*, 5476−5479.
7. C. W. Roberson, K. A. Woerpel, *J. Am. Chem. Soc*. **2002**, *124*, 11342–11348.
8. A. Barbero, A. Diez-Varga, M. Herrero, F. J. Pulido, *J. Org Chem*. **2016**, *81*, 2704−2712.
9. G. Vidari, S. Beszant, J. E. Merabet, M. Bovolenta, G. Zanoni, *Tetrahedron Lett*. **2002**, *43*, 2687–2690.
10. D. S. Tsang, S. Yang, F. Alphonse, A. K. Yudin, *Chem. Eur. J.* **2008**, *14*, 886–894.
11. F. Alphonse, A. K. Yudin, *J. Am. Chem. Soc.* **2006**, *128*, 11754–11755.
12. H. Wang, S. Liu, T. Sun, Z. Lv, Z. Zhan, G. Yin, Z. Chen, *Molecular Catalysis* **2019**, *469*, 10–17.
13. Y. Ito, T. Hirao, T. Saegusa, *J. Org. Chem*. **1978**, *43*, 1011−1013.
14. B. J. Lüssem, Hans-Joachim Gais, *J. Am. Chem. Soc*. **2003**, *125*, 6066–6067.
15. 10.1039/SP123
16. O. Salvado, R. Gava, E. Fernández, *Org. Lett*. **2019**, *21*, 9247–9250.
17. B. Biletskyi, L. Kong, A. Tenaglia, H. Clavier, *Adv. Synth. Catal*. **2021**, *363*, 2578–2585.
18. D. Fujino, H. Yorimitsu, A. Osuka, *J. Am. Chem. Soc*. **2014**, *136*, 6255–6258.
19. T. Kitamura, M. H. Morshed, S. Tsukada, Y. Miyazaki, N. Iguchi, D. Inoue, *J. Org. Chem*. **2011**, *76*, 8117–8120.
20. C. D. Campbell, R. L. Greenaway, O. T. Holton, P. R. Walker, H. A. Chapman, C. A. Russell, G. Carr, A. L. Thomson, E. A. Anderson, *Chem. Eur. J.* **2015**, *21*, 12627–12639.
21. L. F. Cannizzo, R. H. Grubbs, *J. Org. Chem*. **1985**, *50*, 2386–2381.
22. Z. Li, K. R. Conser, E. N. Jacobsen, *J. Am. Chem. Soc*. **1993***, 115,* 5326–5327.
23. L. Copey, L. Jean-Gerard, E. Framery, G. Pilet, V. Robert, B. Andrioletti, *Chem. Eur. J.* **2015**, *21*, 9057–9061.
24. I. Schiffers, T. Rantanen, F. Schmidt, W. Bergmans, L. Zani, C. Bolm, *J. Org. Chem.* **2006**, *71*, 2320–2331.

25. H. Qin, N. Yamagiwa, S, Matsunaga, M. Shibasaki, *Angew. Chem. Int. Ed*. **2007**, *46,* 409–413.

26. O. Kitagawa, T. Suzuki, T. Taguchi, *J. Org. Chem*. **1998**, *63*, 4842 –4845.

27. E. Shi, Y. Shao, S. Chen, H. Hu, Z. Liu, J. Zhang, X. Wan, *Org Lett*. **2012**, *14*, 3384–3387.

28. T. R. Ramadhar, J. Kawakami, A. J. Lough, R. A, Batey, *Org. Lett*. **2010**, *12*, 4446–4449.

29. B. J. Lüssem, Hans-Joachim Gais, *J. Am. Chem. Soc*. **2003**, *125*, 6066–6067.

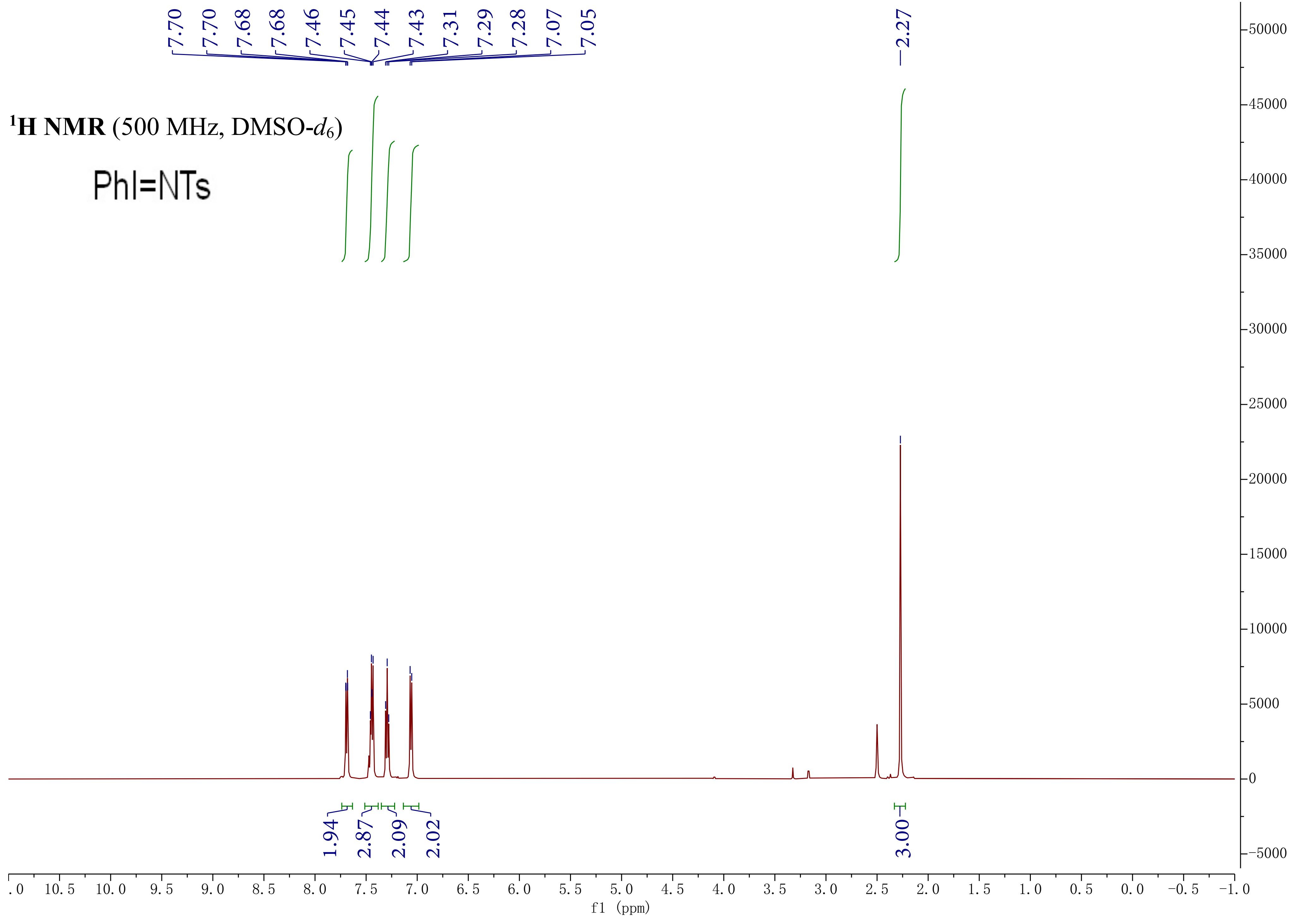

7.70
7.70
7.68
7.68
7.46
7.45
7.44
7.43
7.31
7.29
7.28
7.07
7.05
2.27
1H NMR (500 MHz, DMSO-d6)
PhI=NTs
1.94
2.87
2.09
2.02
3.00
f1 (ppm)

^{13}C NMR (125 MHz, DMSO-d_6)

PhI=NTs

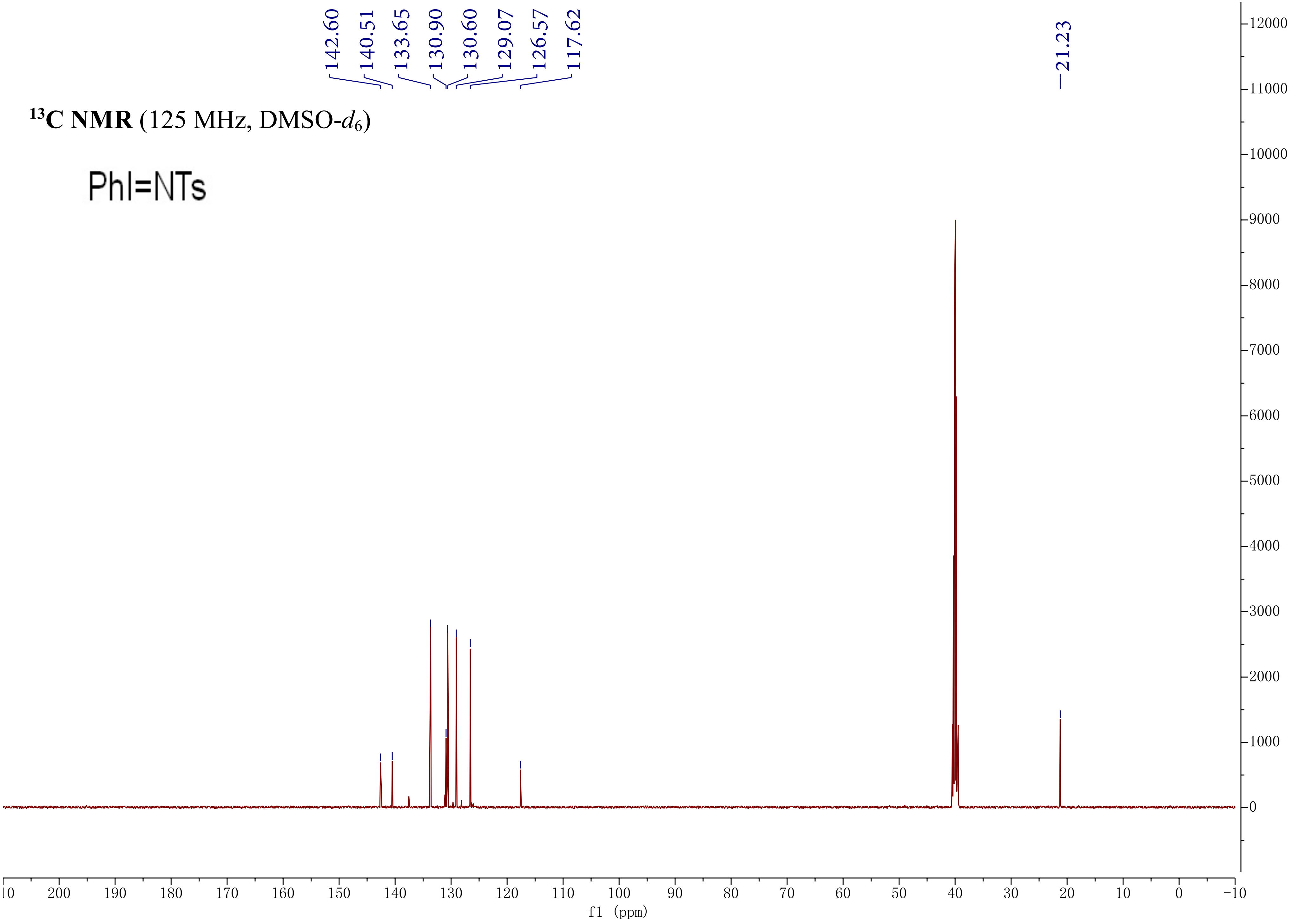

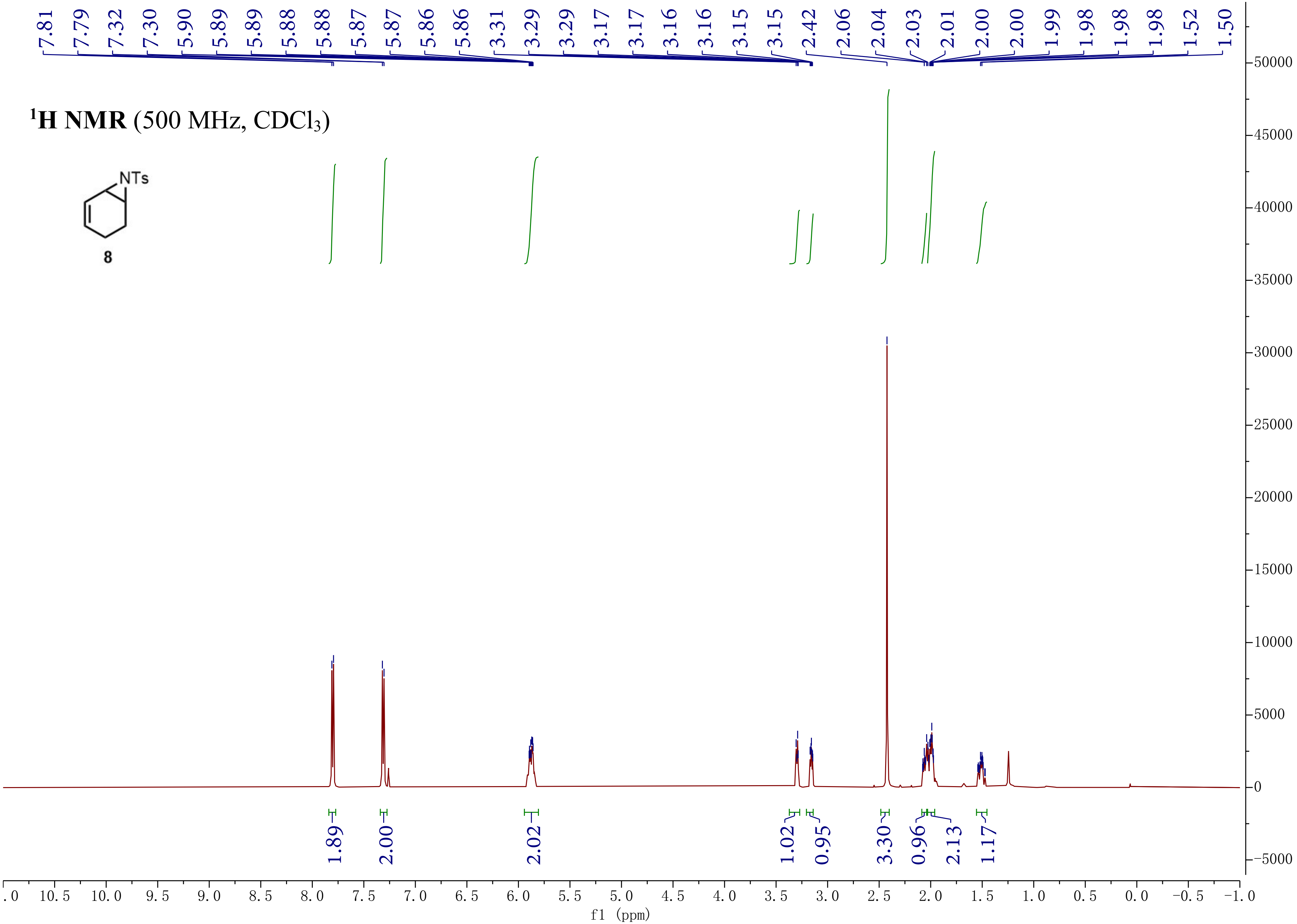

7.81 7.79 7.32 7.30 5.90 5.89 5.89 5.88 5.88 5.87 5.87 5.86 5.86 3.31 3.29 3.29 3.17 3.17 3.16 3.16 3.15 3.15 2.42 2.06 2.04 2.03 2.01 2.00 2.00 1.99 1.98 1.98 1.98 1.52 1.50
¹H NMR (500 MHz, CDCl3)
NTs
8
1.89 2.00 2.02 1.02 0.95 3.30 0.96 2.13 1.17
f1 (ppm)

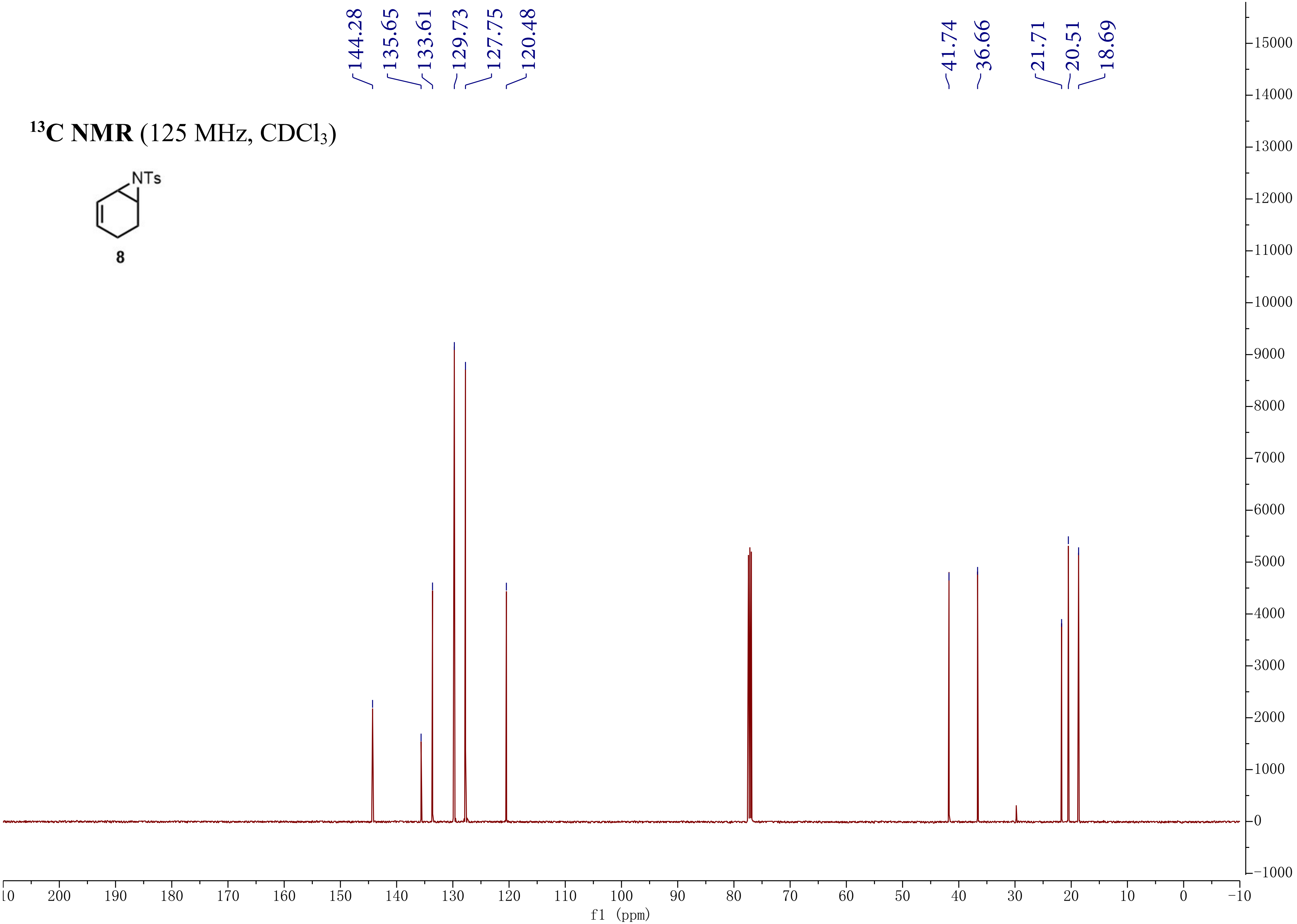

144.28
135.65
133.61
129.73
127.75
120.48
41.74
36.66
21.71
20.51
18.69
13C NMR (125 MHz, CDCl3)
NTs
8
f1 (ppm)

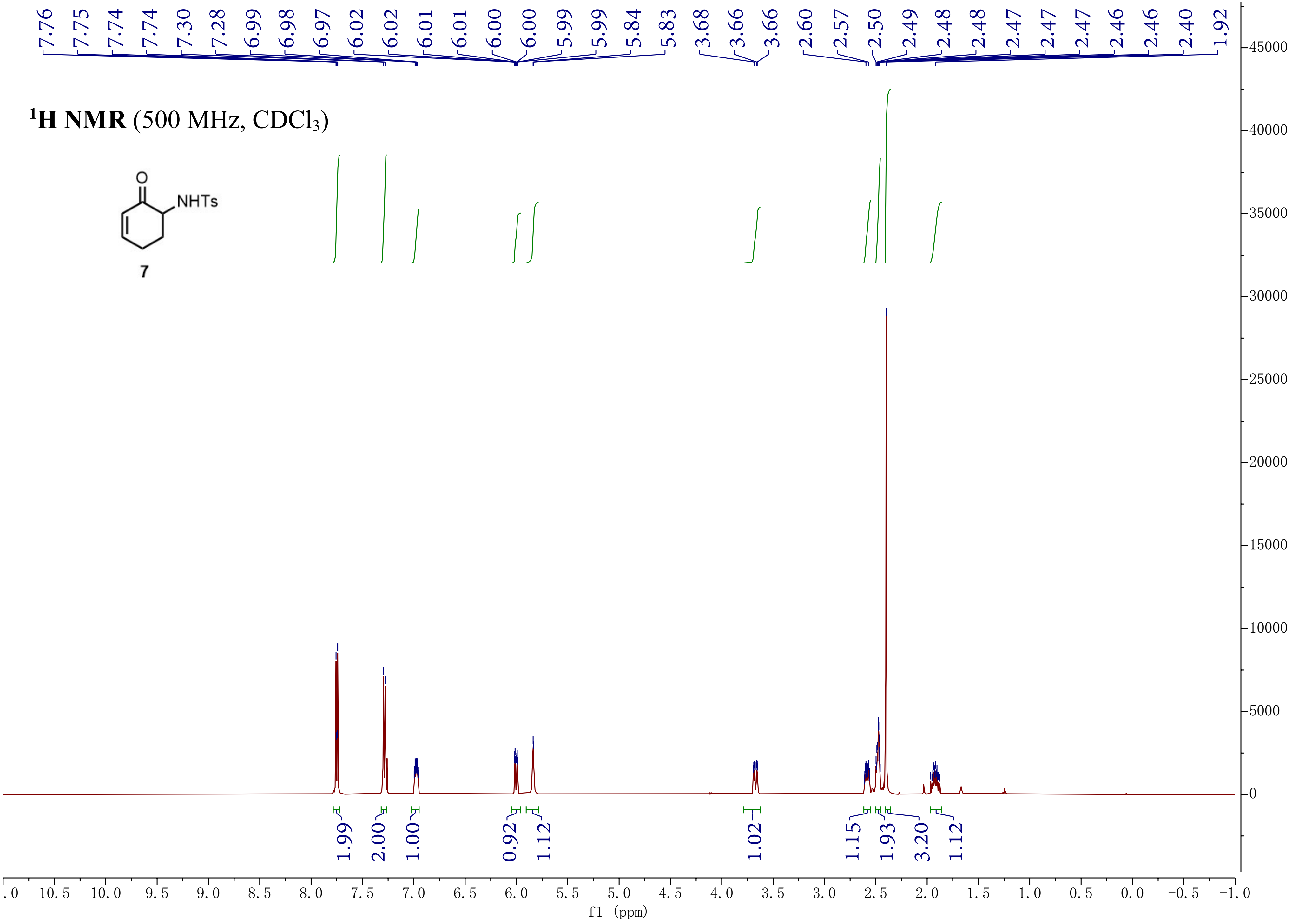

1H NMR (500 MHz, CDCl3)
O
NHTs
7
7.76
7.75
7.74
7.74
7.30
7.28
6.99
6.98
6.97
6.02
6.02
6.01
6.01
6.00
6.00
5.99
5.99
5.84
5.83
3.68
3.66
3.66
2.60
2.57
2.50
2.49
2.48
2.48
2.47
2.47
2.47
2.46
2.46
2.40
1.92
1.99
2.00
1.00
0.92
1.12
1.02
1.15
1.93
3.20
1.12
45000
40000
35000
30000
25000
20000
15000
10000
5000
0
f1 (ppm)

^{13}C NMR (125 MHz, $CDCl_3$)

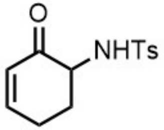

7

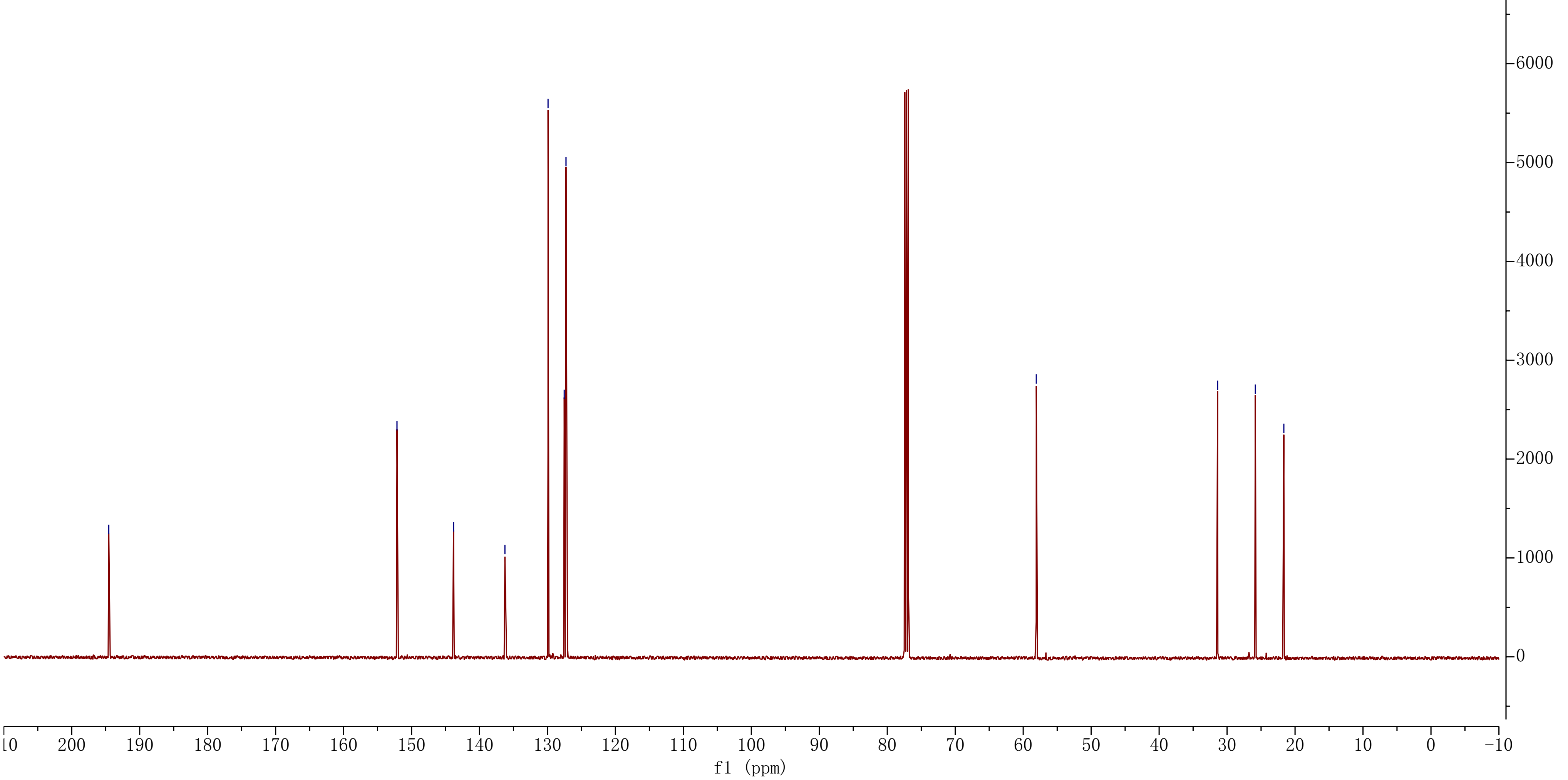

^{1}H NMR (400 MHz, $CDCl_3$)

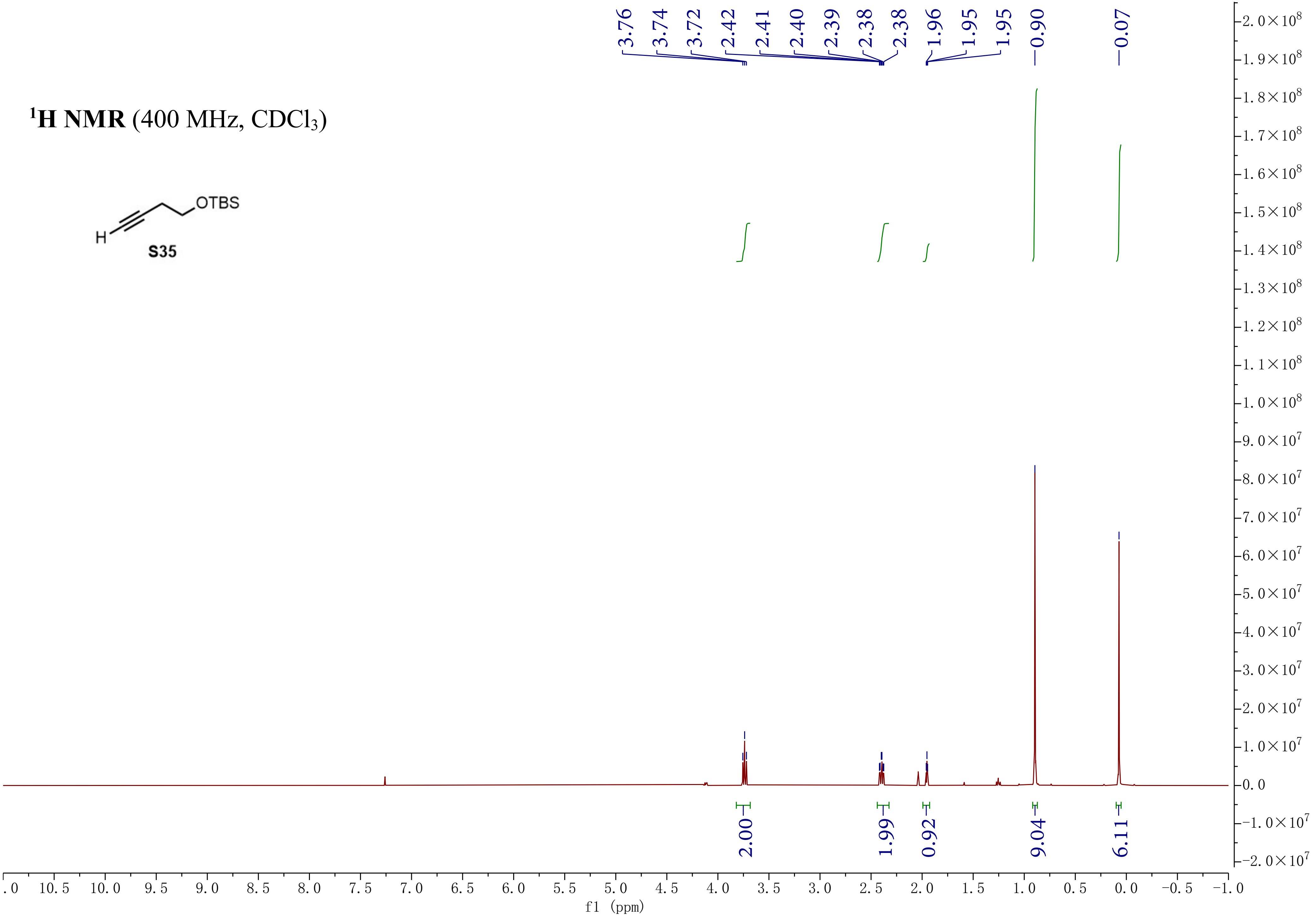

^{13}C NMR (100 MHz, $CDCl_3$)

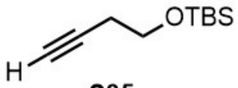

S35

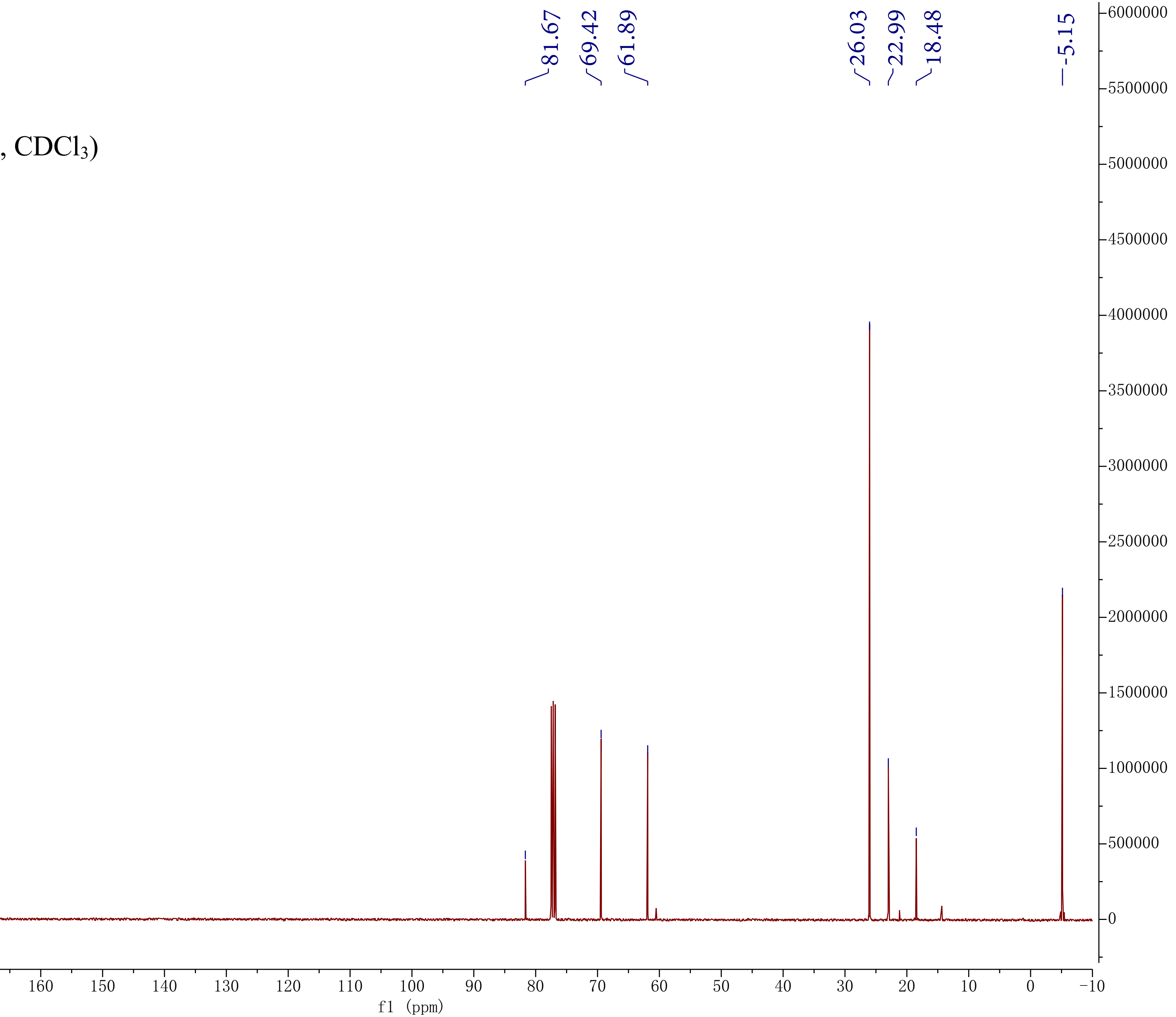

^{1}H NMR (500 MHz, $CDCl_3$)

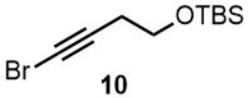

10

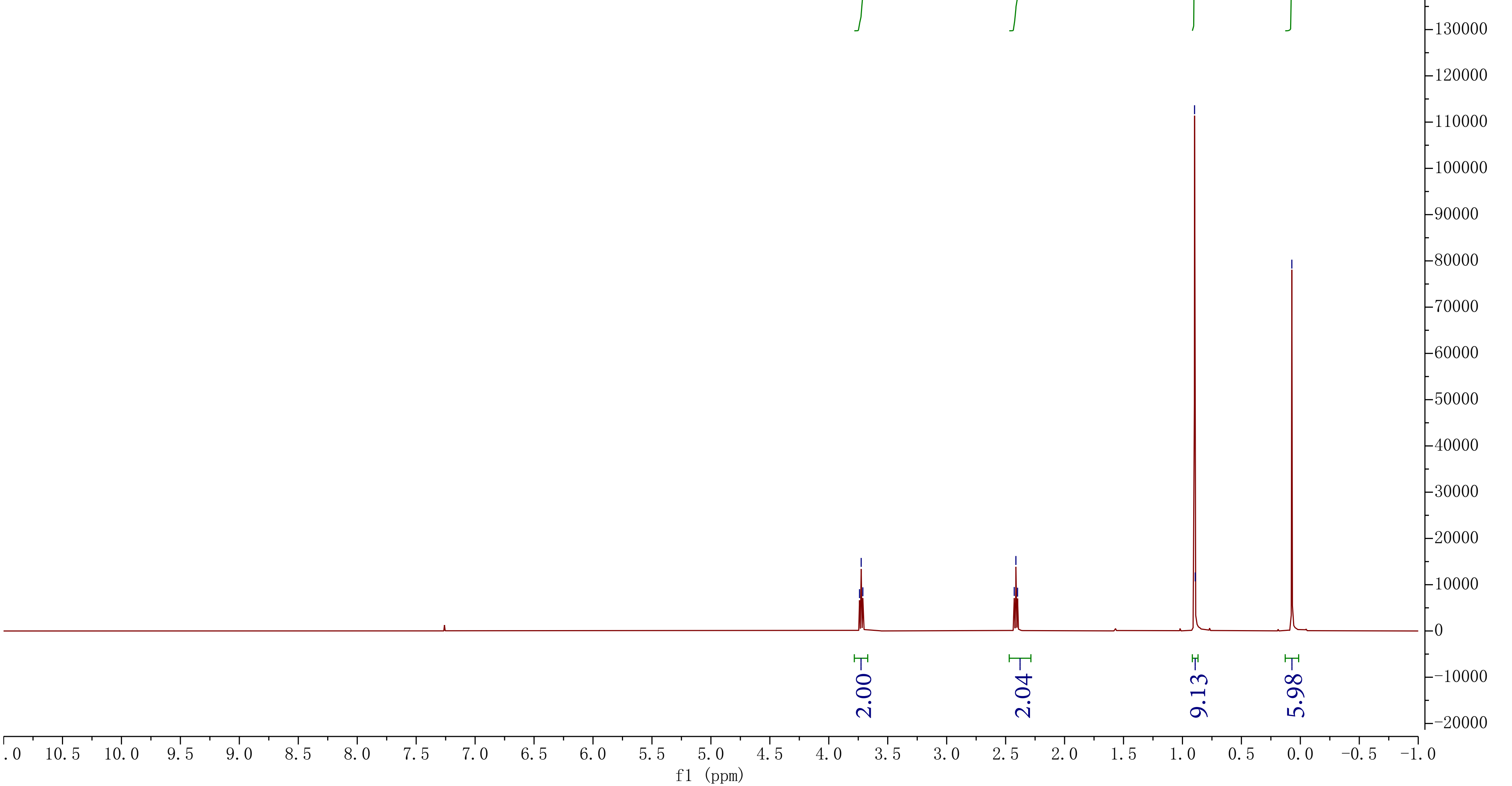

^{13}**C NMR** (125 MHz, $CDCl_3$)

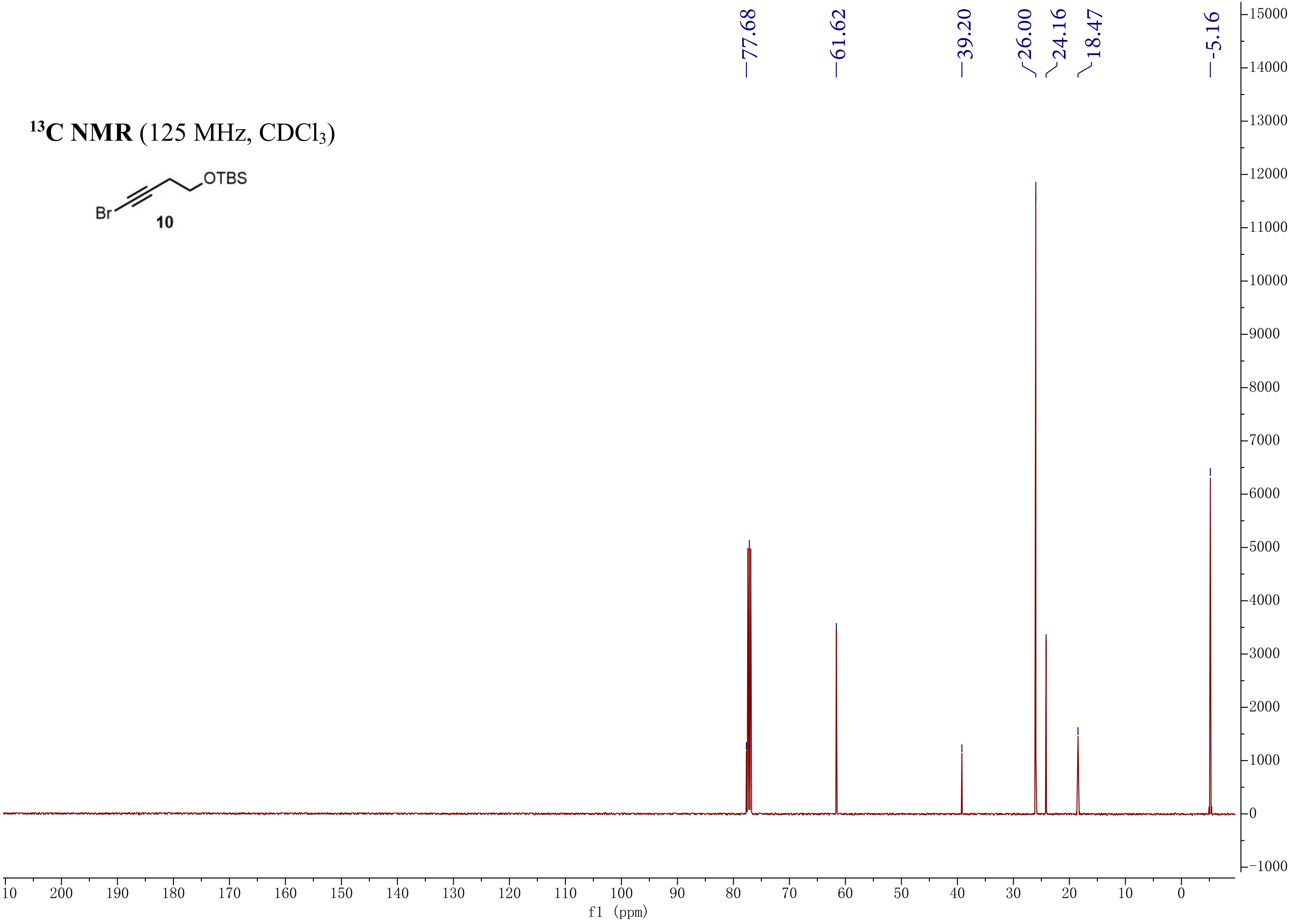

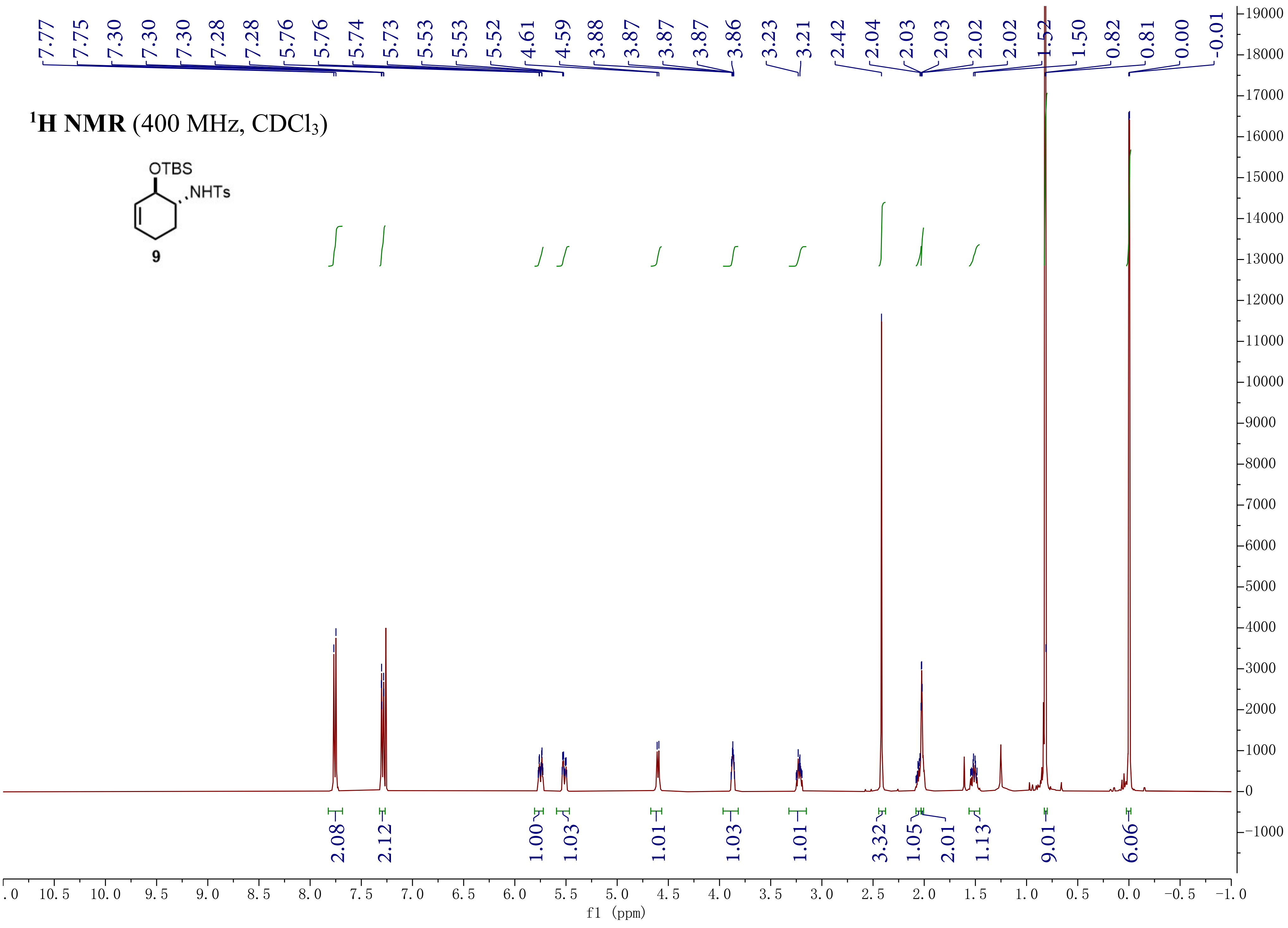
1H NMR (400 MHz, CDCl3)
OTBS
NHTs
9
7.77 7.75 7.30 7.30 7.30 7.28 7.28 5.76 5.76 5.74 5.73 5.53 5.53 5.52 4.61 4.59 3.88 3.87 3.87 3.87 3.86 3.23 3.21 2.42 2.04 2.03 2.03 2.02 2.02 1.52 1.50 0.82 0.81 0.00 -0.01
2.08 2.12 1.00 1.03 1.01 1.03 1.01 3.32 1.05 2.01 1.13 9.01 6.06
f1 (ppm)

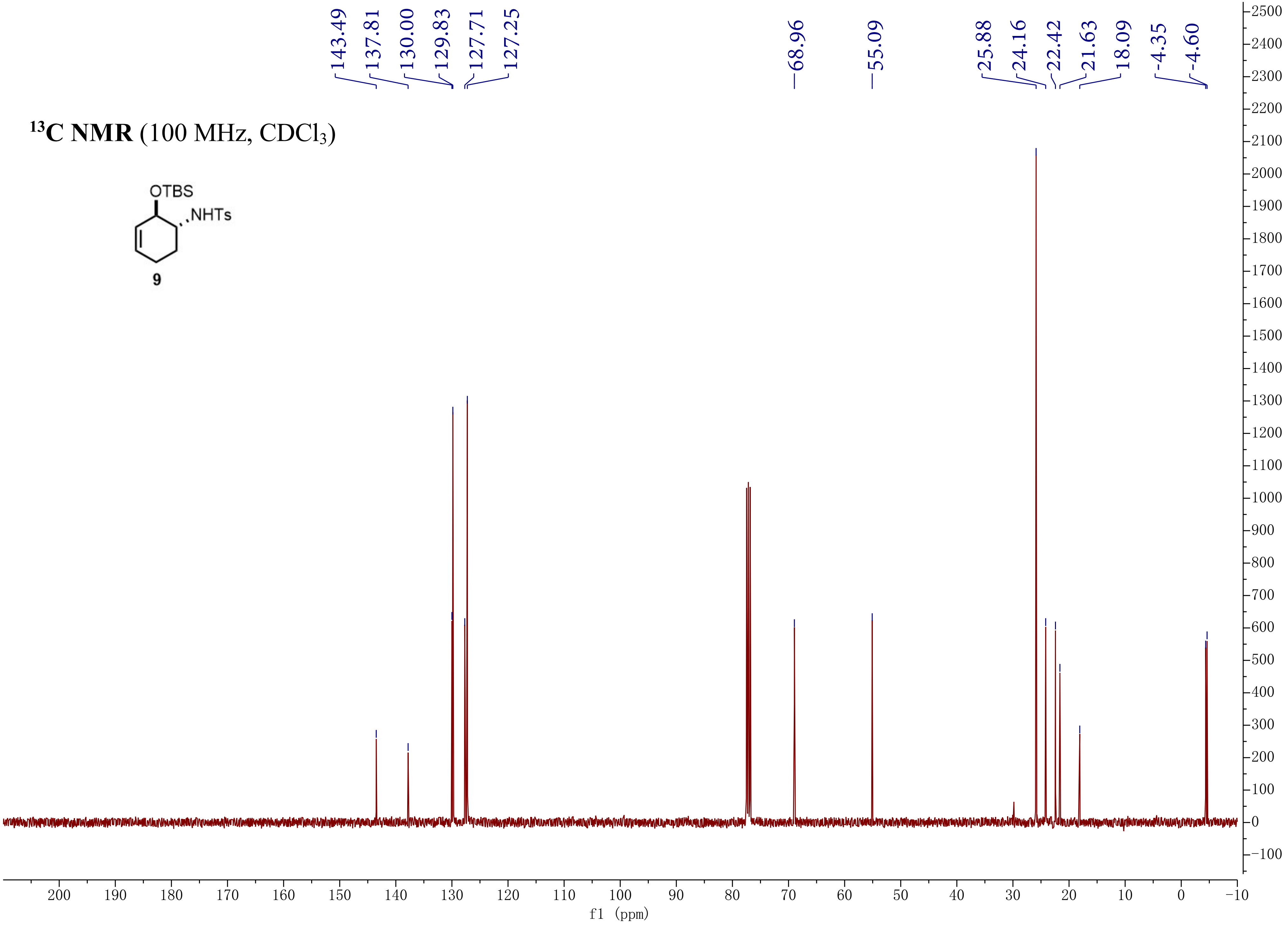

13C NMR (100 MHz, CDCl3)
OTBS
NHTs
9
143.49
137.81
130.00
129.83
127.71
127.25
68.96
55.09
25.88
24.16
22.42
21.63
18.09
-4.35
-4.60
f1 (ppm)

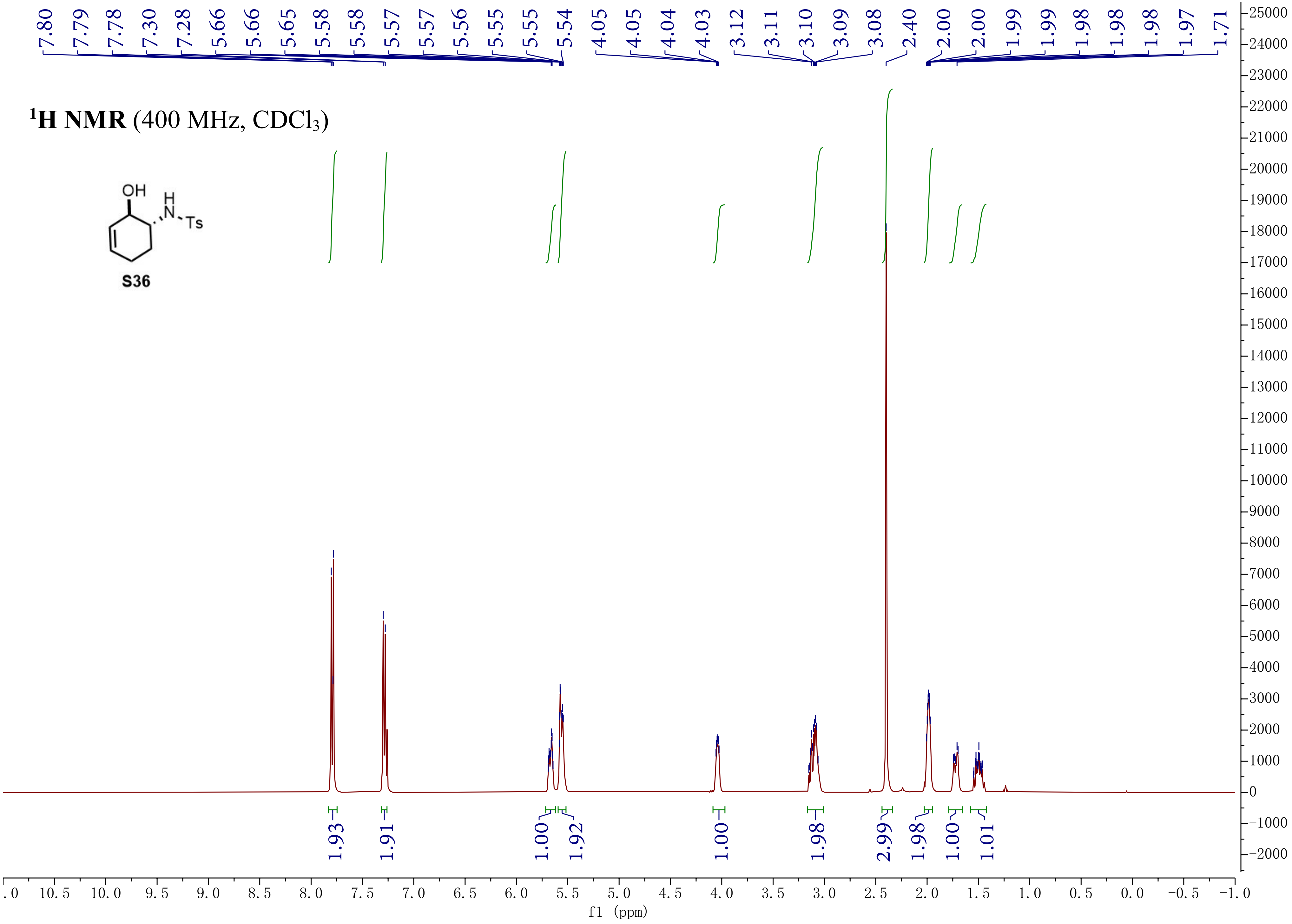

7.80
7.79
7.78
7.30
7.28
5.66
5.66
5.65
5.58
5.58
5.57
5.57
5.56
5.55
5.55
5.54
4.05
4.05
4.04
4.03
3.12
3.11
3.10
3.09
3.08
2.40
2.00
2.00
1.99
1.99
1.98
1.98
1.98
1.97
1.71
1H NMR (400 MHz, CDCl3)
OH
H
N
Ts
S36
1.93
1.91
1.00
1.92
1.00
1.98
2.99
1.98
1.00
1.01
f1 (ppm)

^{13}C NMR (100 MHz, $CDCl_3$)

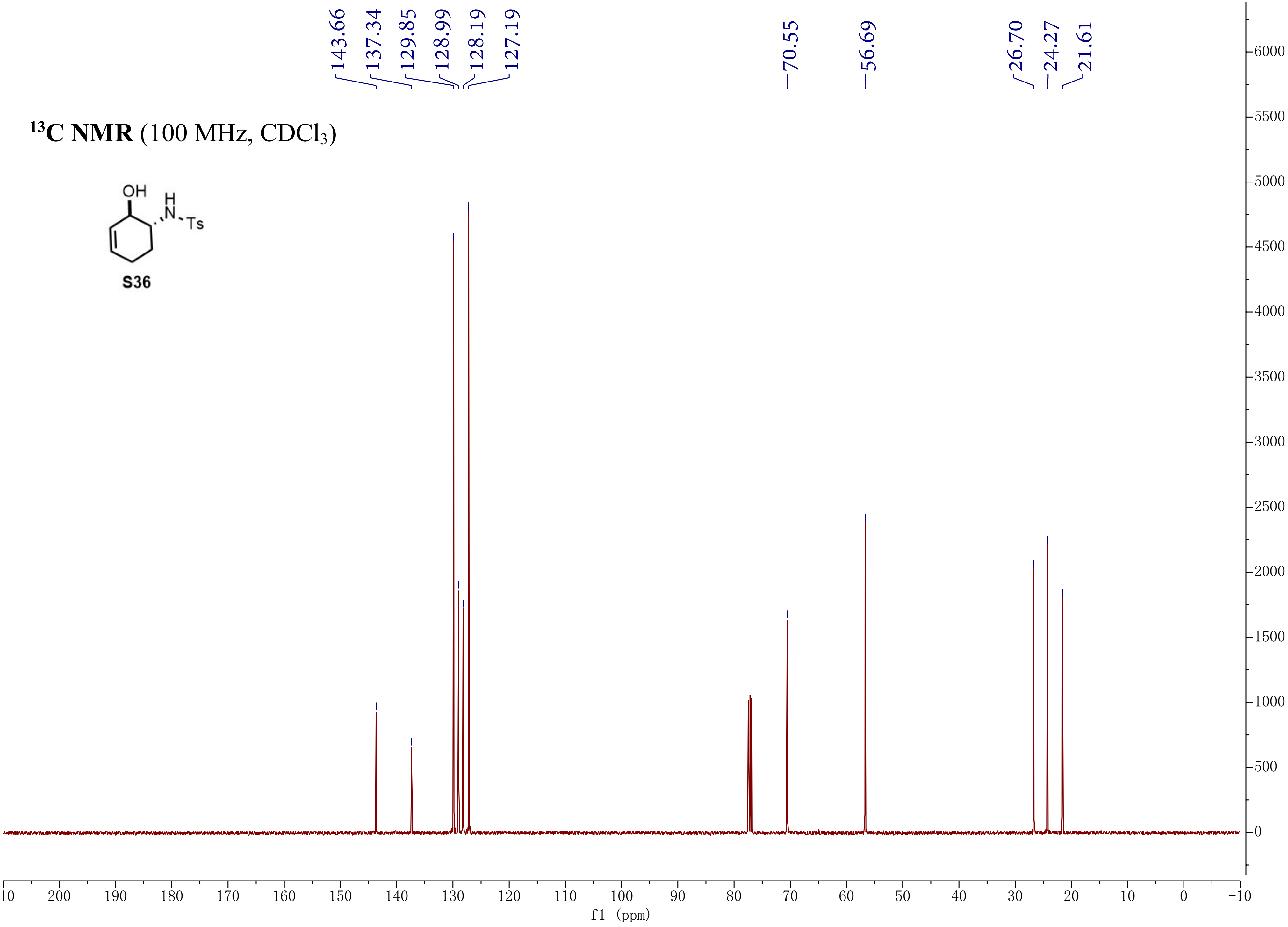

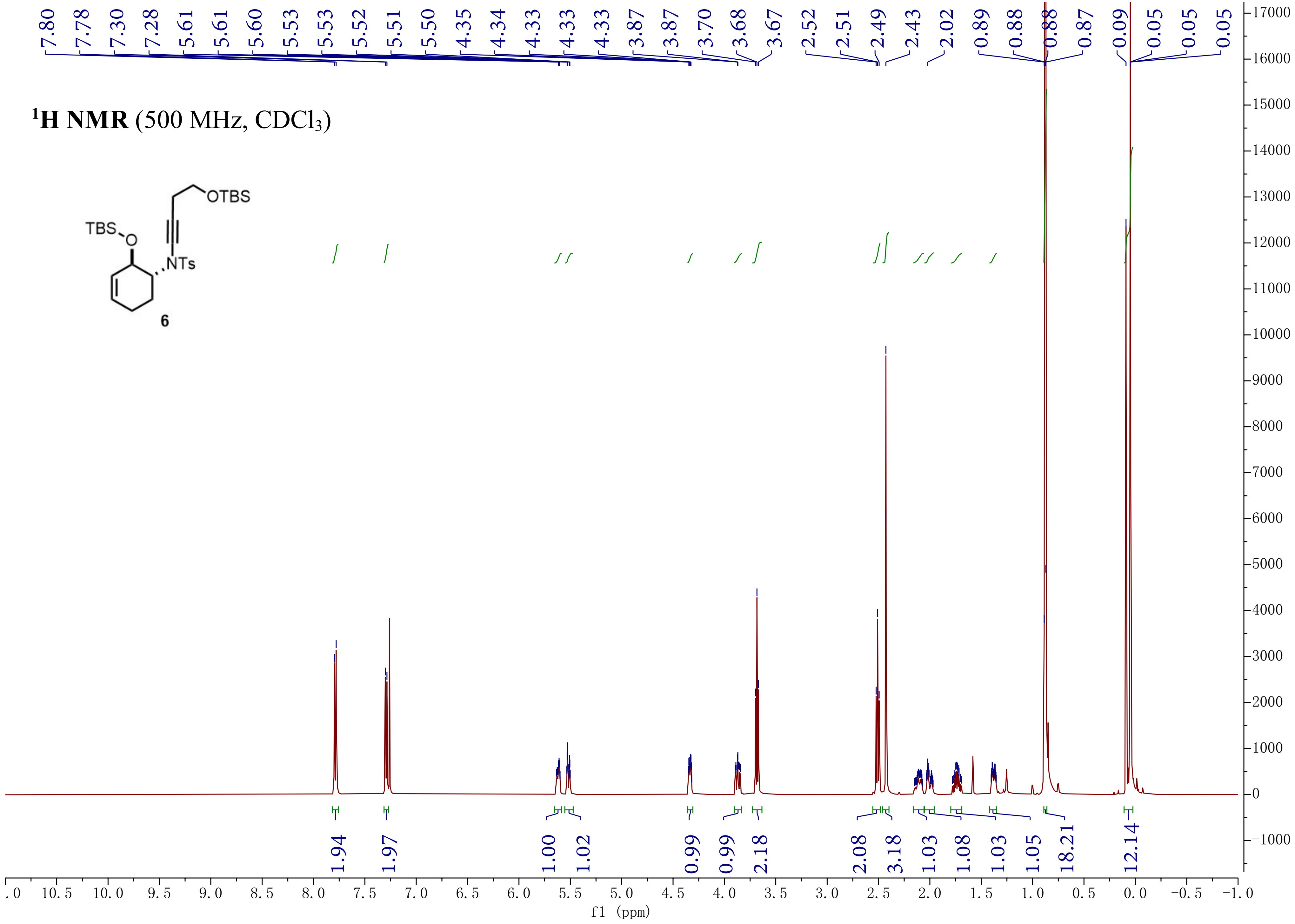

1H NMR (500 MHz, CDCl3)
7.80 7.78 7.30 7.28 5.61 5.61 5.60 5.53 5.53 5.52 5.51 5.50 4.35 4.34 4.33 4.33 4.33 3.87 3.87 3.70 3.68 3.67 2.52 2.51 2.49 2.43 2.02 0.89 0.88 0.88 0.87 0.09 0.05 0.05 0.05
OTBS
TBS
O
NTs
6
1.94 1.97 1.00 1.02 0.99 0.99 2.18 2.08 3.18 1.03 1.08 1.03 1.05 18.21 12.14
f1 (ppm)

^{13}C NMR (125 MHz, $CDCl_3$)

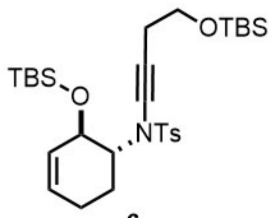

6

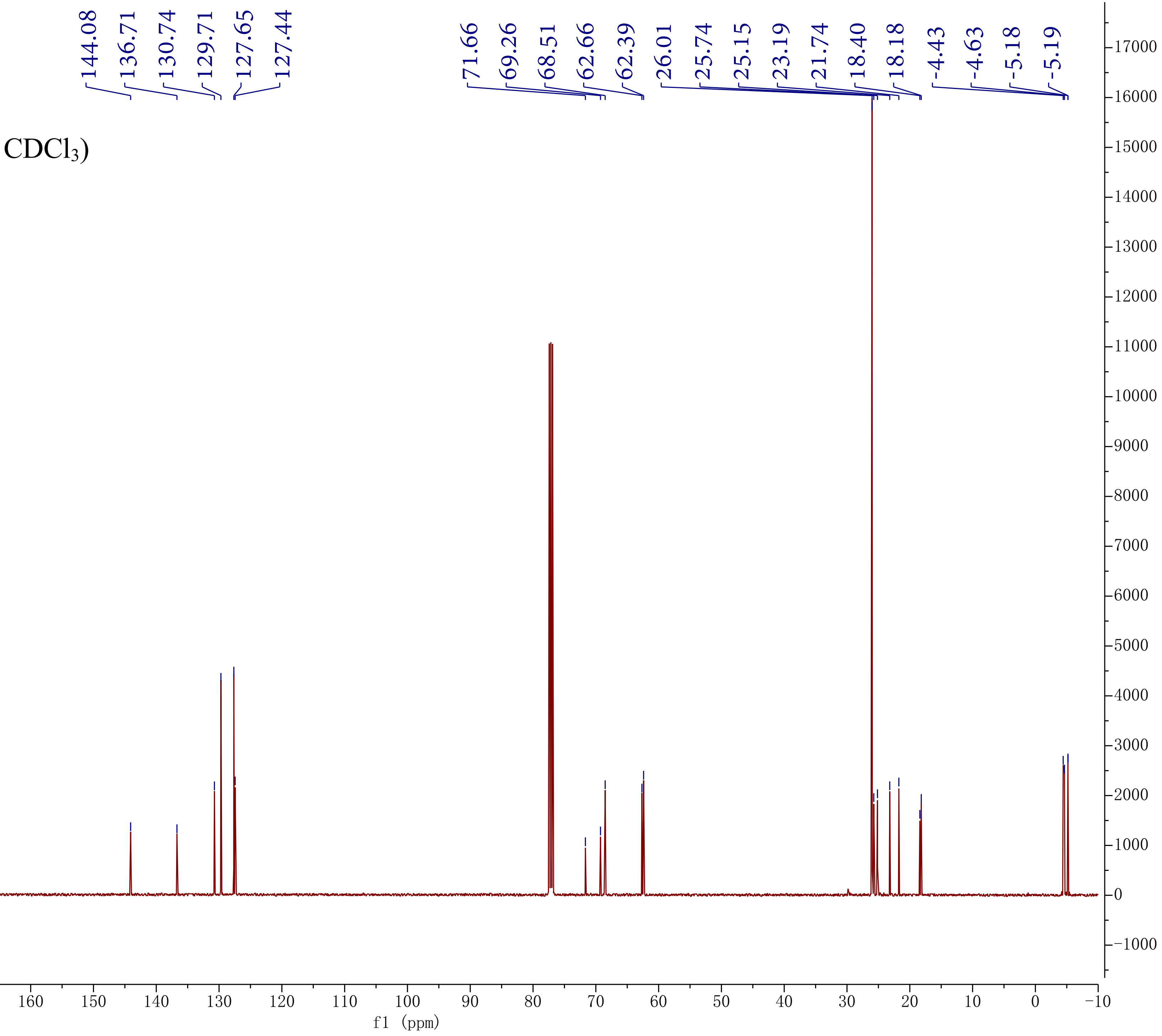

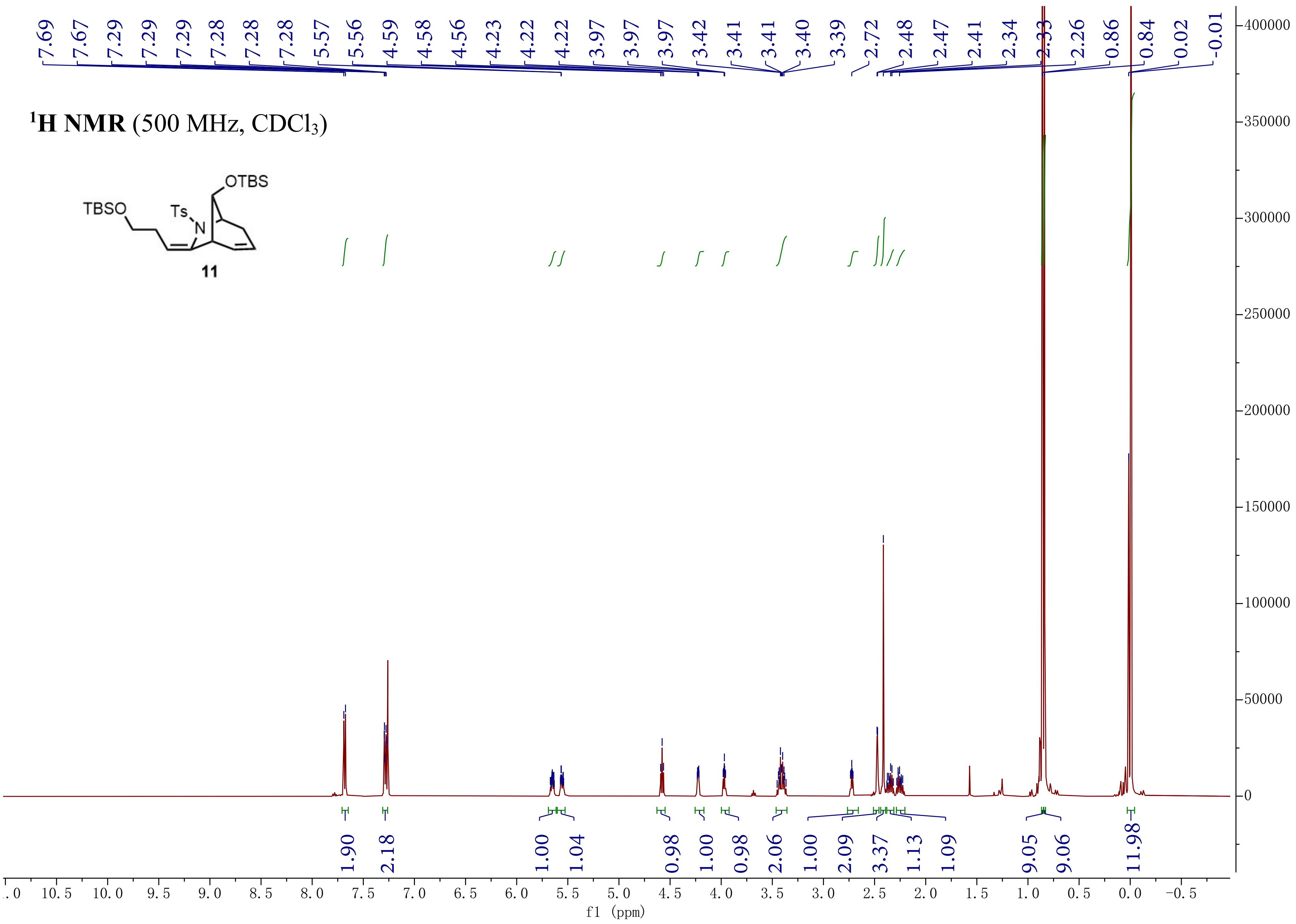
1H NMR (500 MHz, CDCl3)
TBSO
Ts
N
OTBS
11
7.69 7.67 7.29 7.29 7.29 7.28 7.28 7.28 5.57 5.56 4.59 4.58 4.56 4.23 4.22 4.22 3.97 3.97 3.97 3.42 3.41 3.41 3.40 3.39 2.72 2.48 2.47 2.41 2.34 2.33 2.26 0.86 0.84 0.02 -0.01
1.90 2.18 1.00 1.04 0.98 1.00 0.98 2.06 1.00 2.09 3.37 1.13 1.09 9.05 9.06 11.98
f1 (ppm)

^{13}**C NMR** (125 MHz, $CDCl_3$)

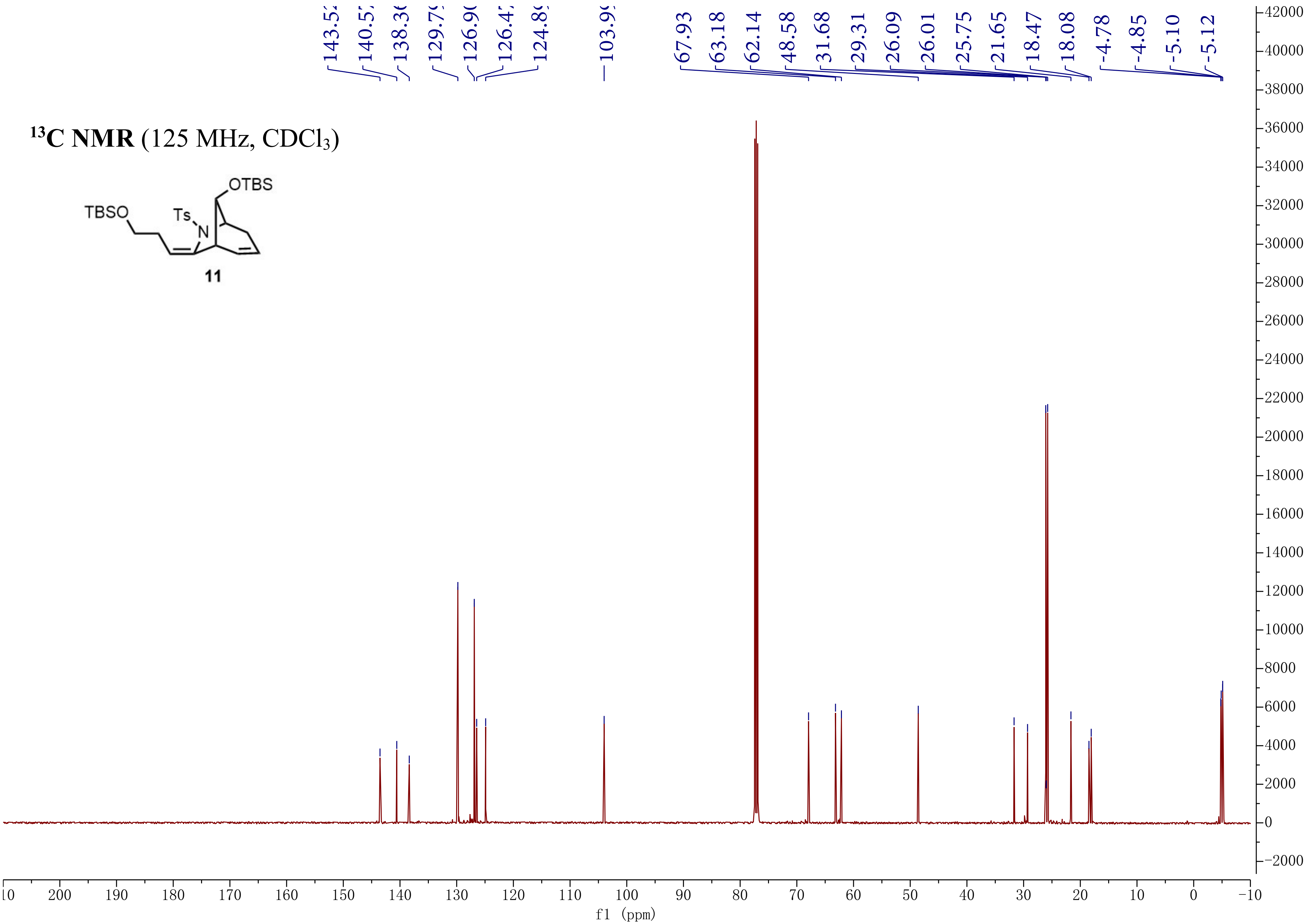

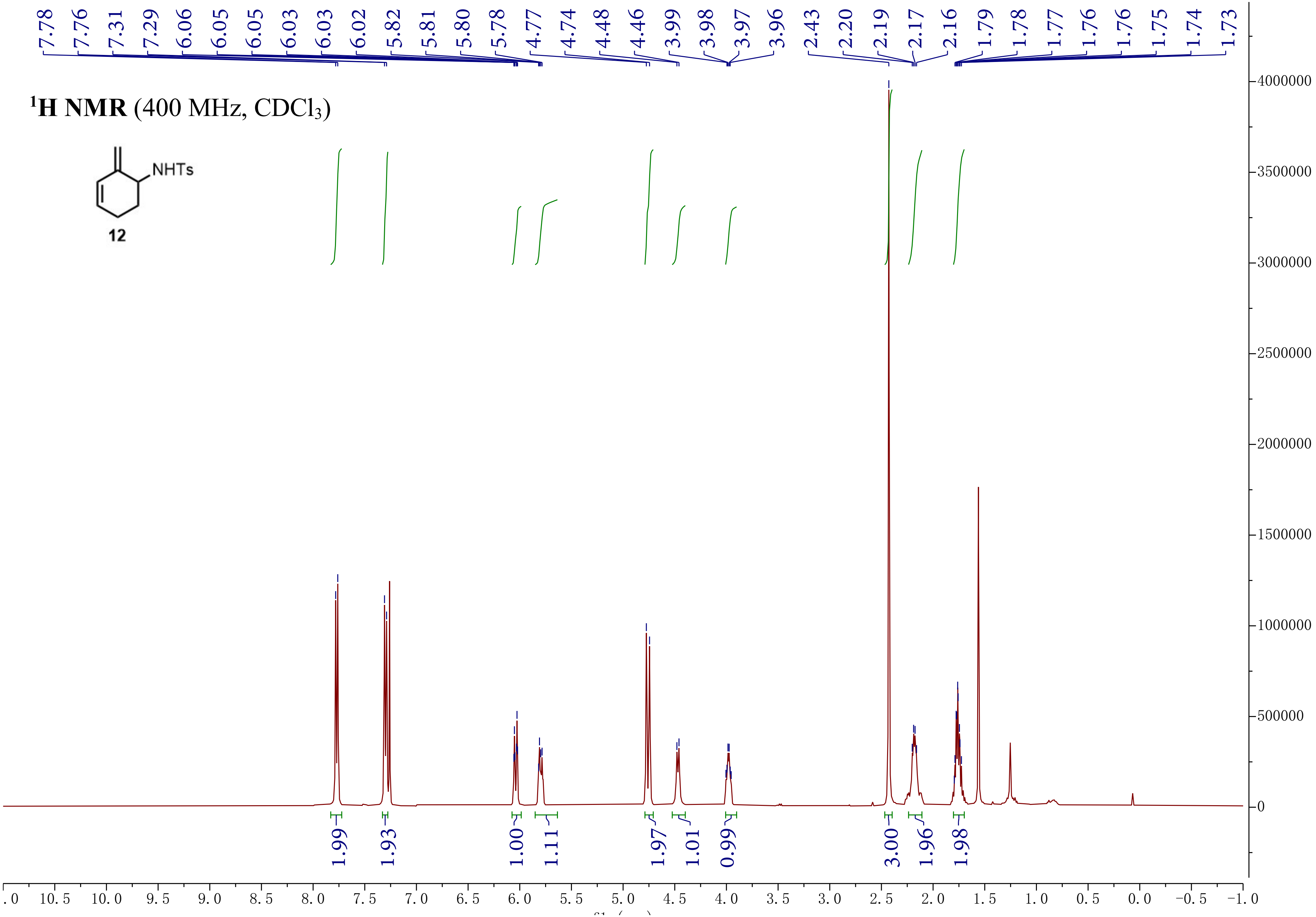

7.78
7.76
7.31
7.29
6.06
6.05
6.05
6.03
6.03
6.02
5.82
5.81
5.80
5.78
4.77
4.74
4.48
4.46
3.99
3.98
3.97
3.96
2.43
2.20
2.19
2.17
2.16
1.79
1.78
1.77
1.76
1.76
1.75
1.74
1.73
1H NMR (400 MHz, CDCl3)
NHTs
12
1.99
1.93
1.00
1.11
1.97
1.01
0.99
3.00
1.96
1.98
4000000
3500000
3000000
2500000
2000000
1500000
1000000
500000
0
10.5
10.0
9.5
9.0
8.5
8.0
7.5
7.0
6.5
6.0
5.5
5.0
4.5
4.0
3.5
3.0
2.5
2.0
1.5
1.0
0.5
0.0
-0.5
-1.0

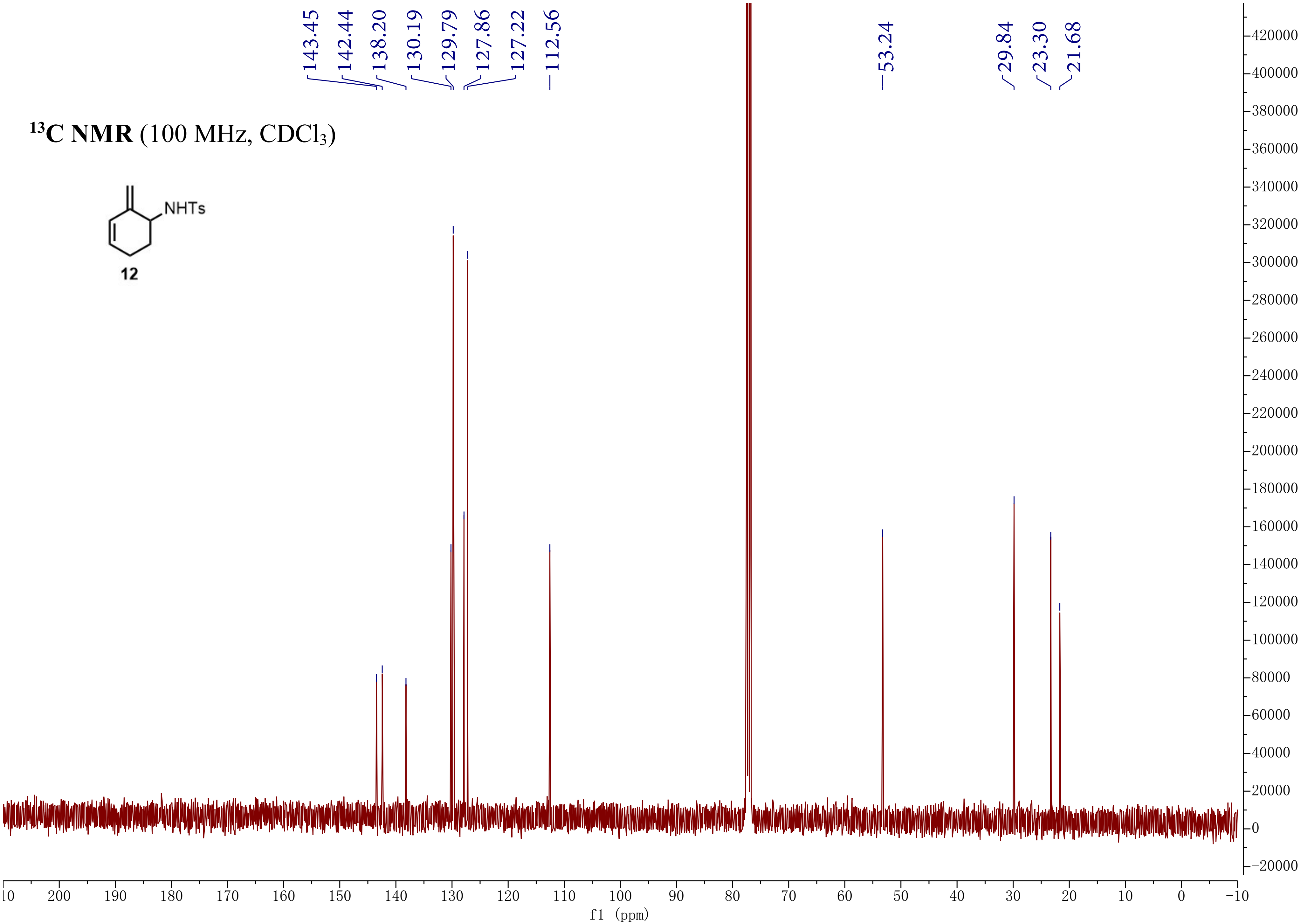

13C NMR (100 MHz, CDCl3)
NHTs
12
143.45
142.44
138.20
130.19
129.79
127.86
127.22
112.56
53.24
29.84
23.30
21.68
f1 (ppm)

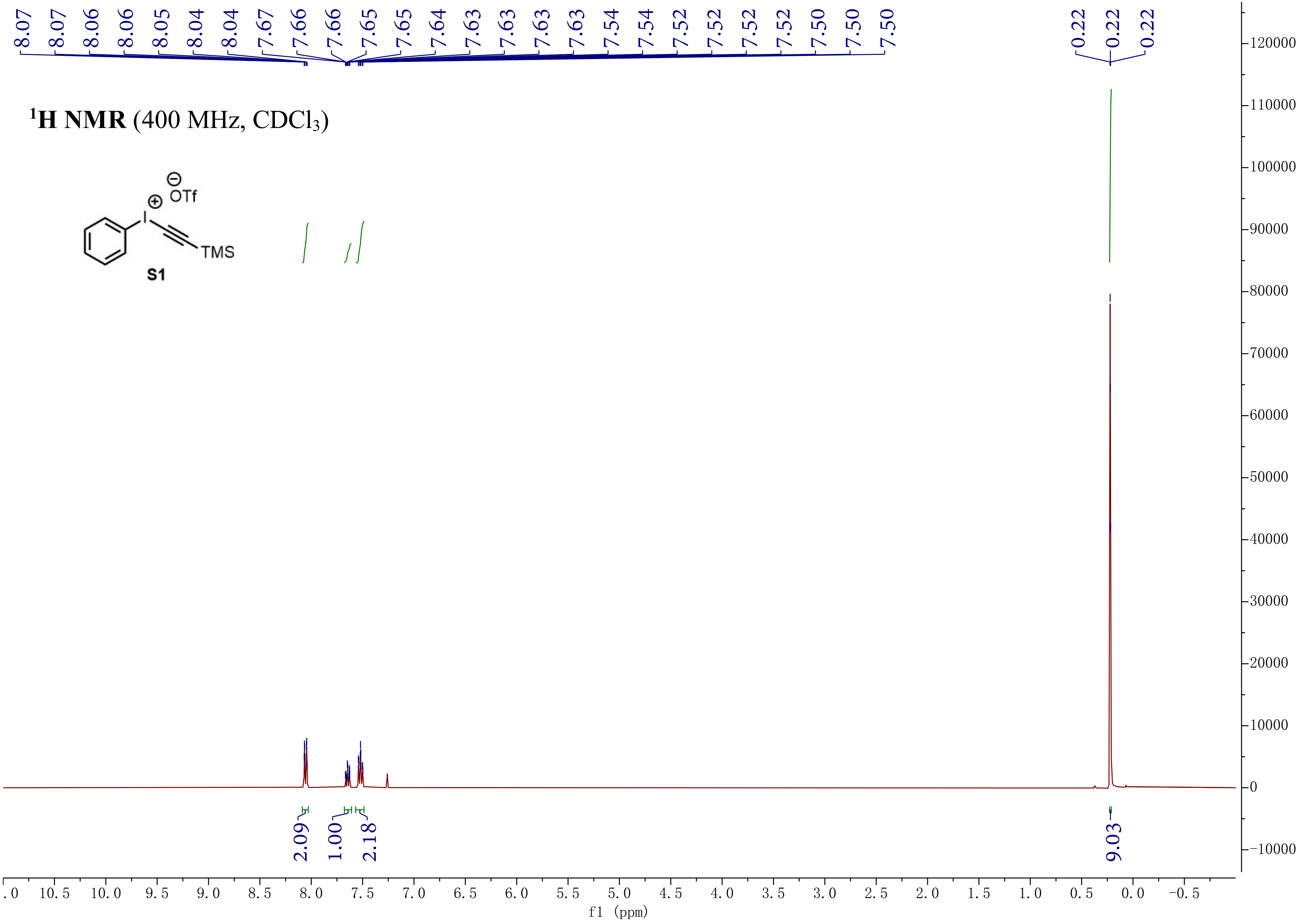
1H NMR (400 MHz, CDCl3)
OTf
TMS
S1
8.07 8.07 8.06 8.06 8.05 8.04 8.04 7.67 7.66 7.66 7.65 7.65 7.64 7.63 7.63 7.63 7.63 7.54 7.54 7.52 7.52 7.52 7.52 7.50 7.50 7.50
0.22 0.22 0.22
2.09 1.00 2.18
9.03
f1 (ppm)

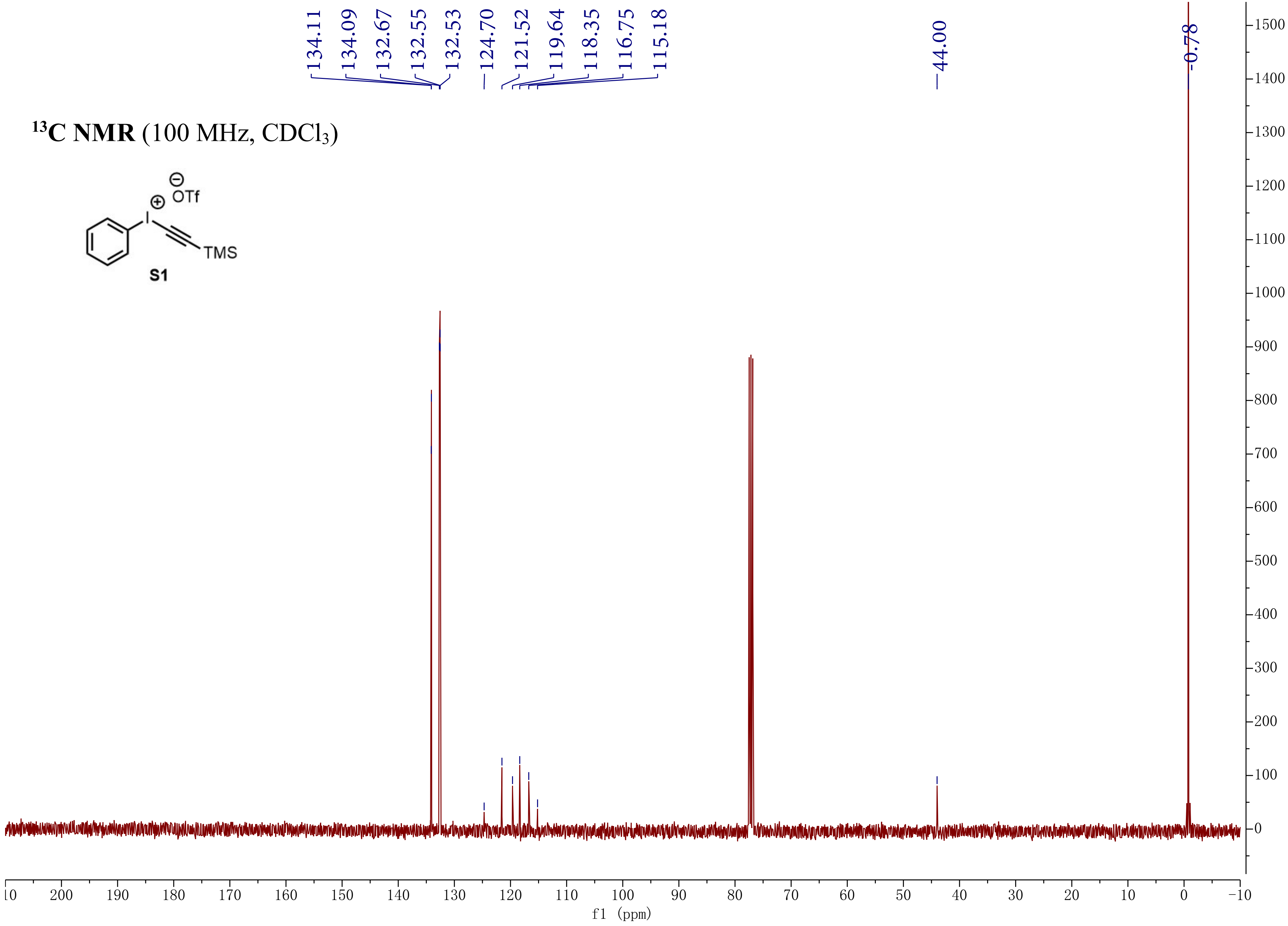
134.11
134.09
132.67
132.55
132.53
124.70
121.52
119.64
118.35
116.75
115.18
44.00
-0.78
13C NMR (100 MHz, CDCl3)
OTf
I
TMS
S1
f1 (ppm)

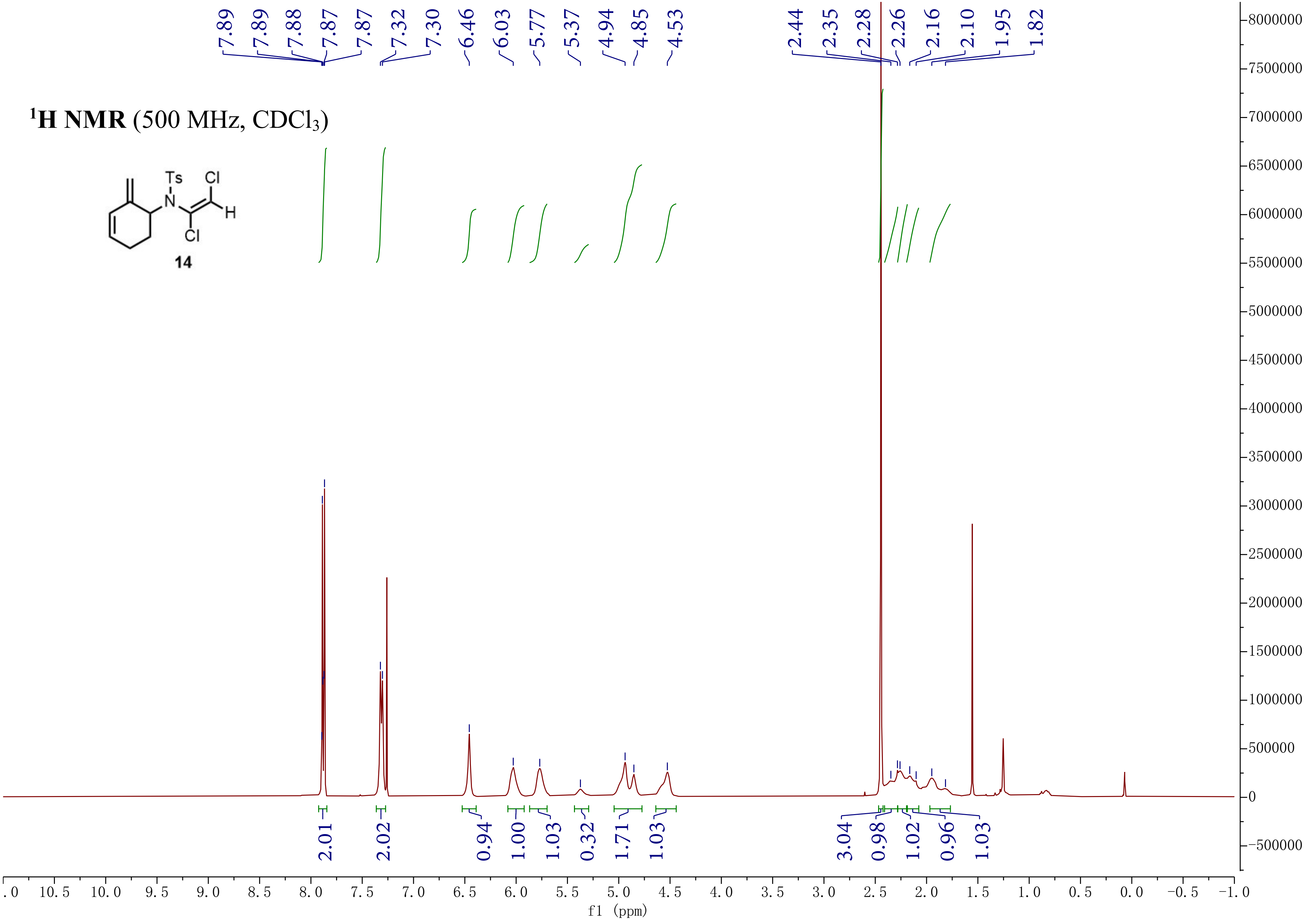

^{1}H NMR (500 MHz, CDCl$_3$)
Ts
N
Cl
H
Cl
14
7.89
7.89
7.88
7.87
7.87
7.32
7.30
6.46
6.03
5.77
5.37
4.94
4.85
4.53
2.44
2.35
2.28
2.26
2.16
2.10
1.95
1.82
2.01
2.02
0.94
1.00
1.03
0.32
1.71
1.03
3.04
0.98
1.02
0.96
1.03
f1 (ppm)

^{13}C NMR (125 MHz, $CDCl_3$)

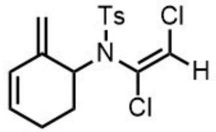

14

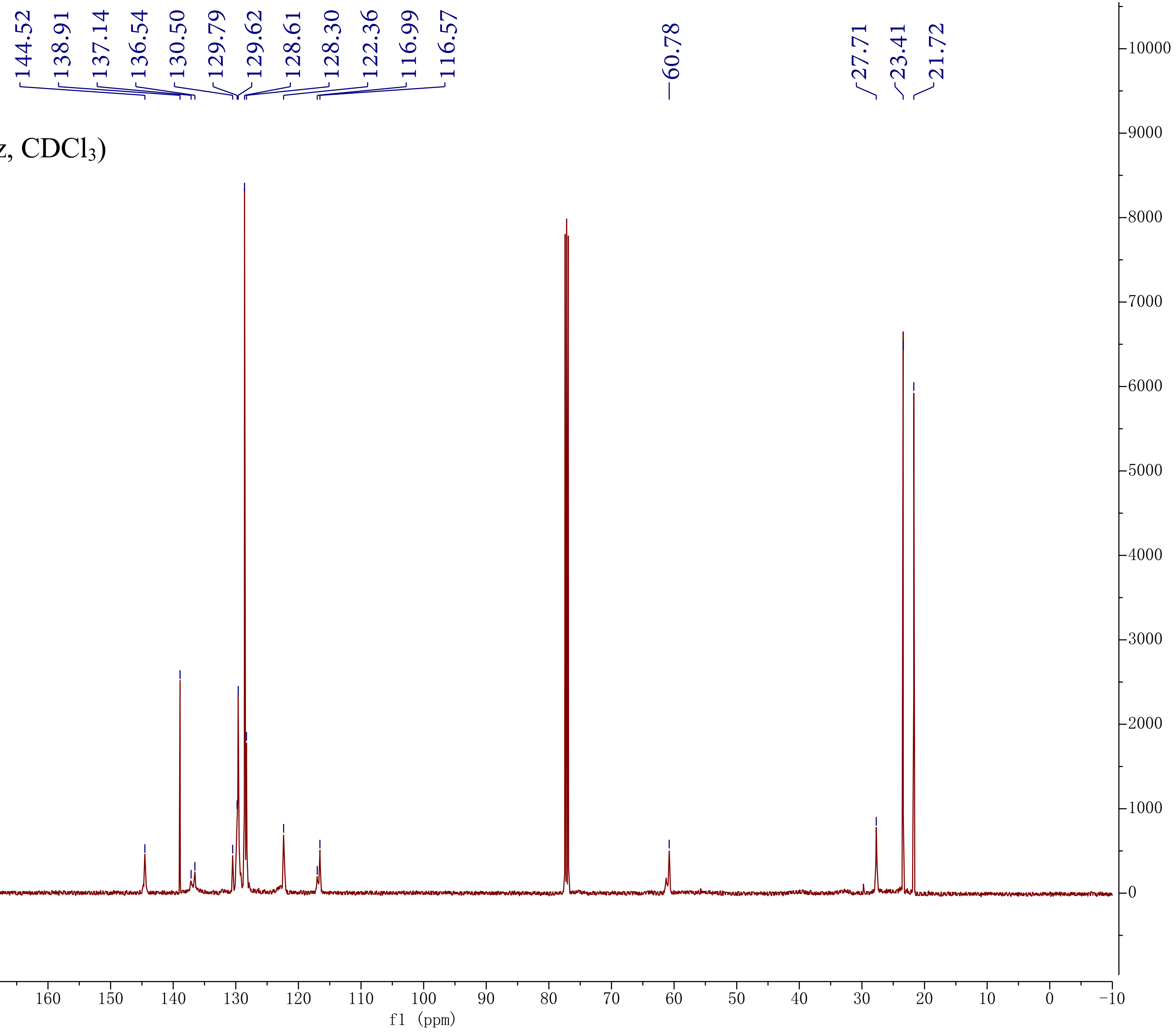

^{1}H NMR (500 MHz, $CDCl_3$)

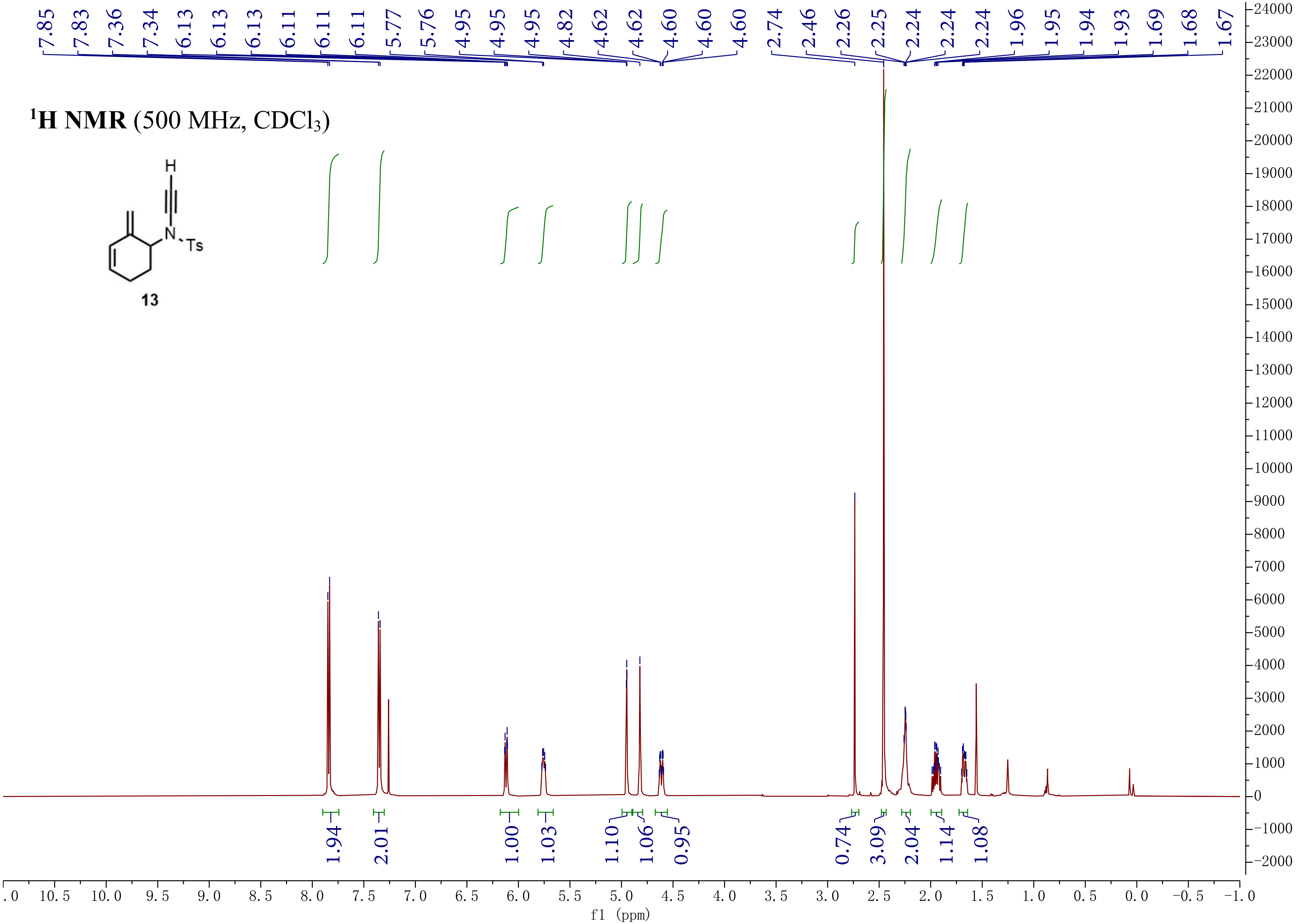

^{13}C NMR (125 MHz, $CDCl_3$)

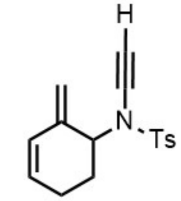

13

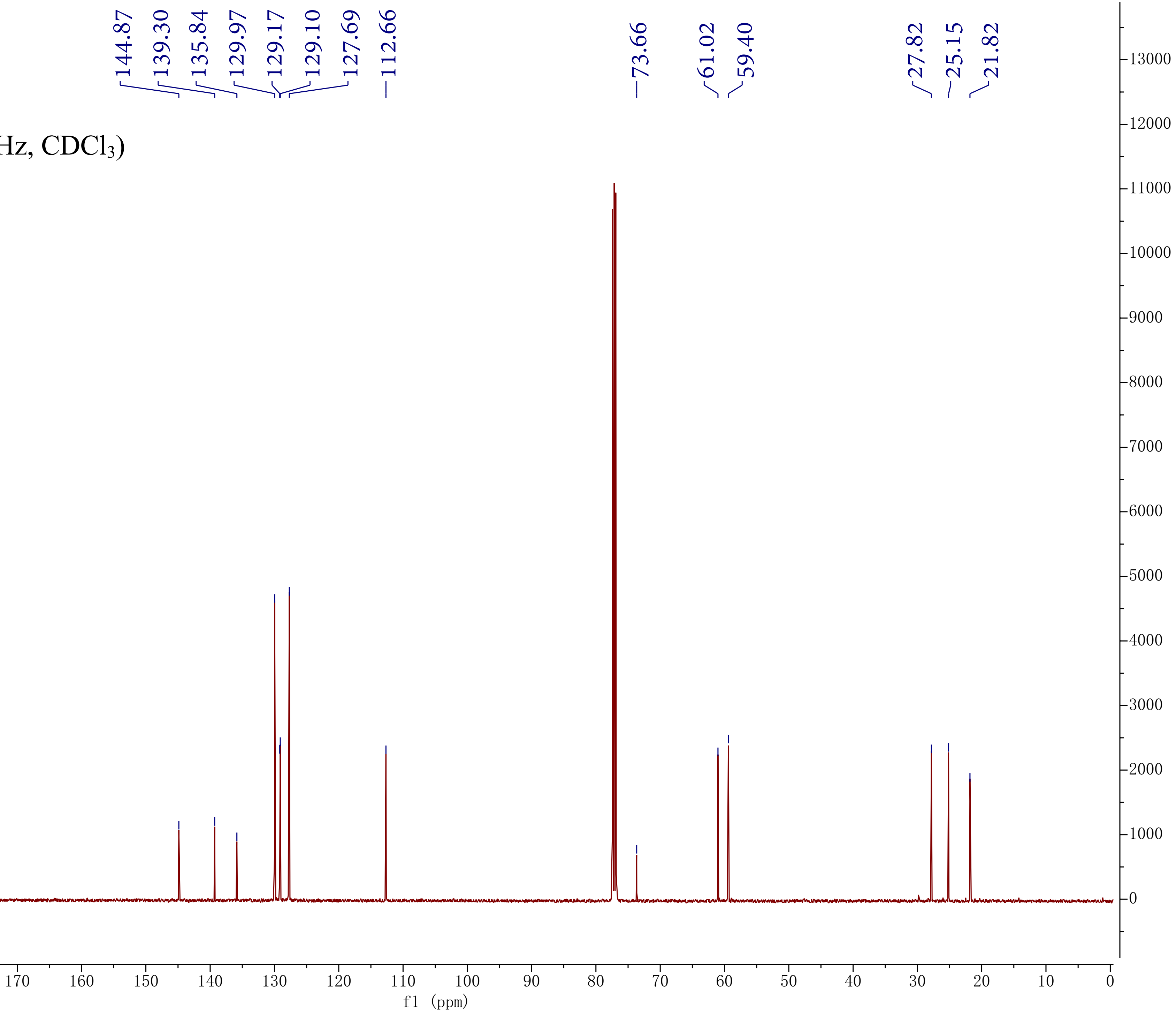

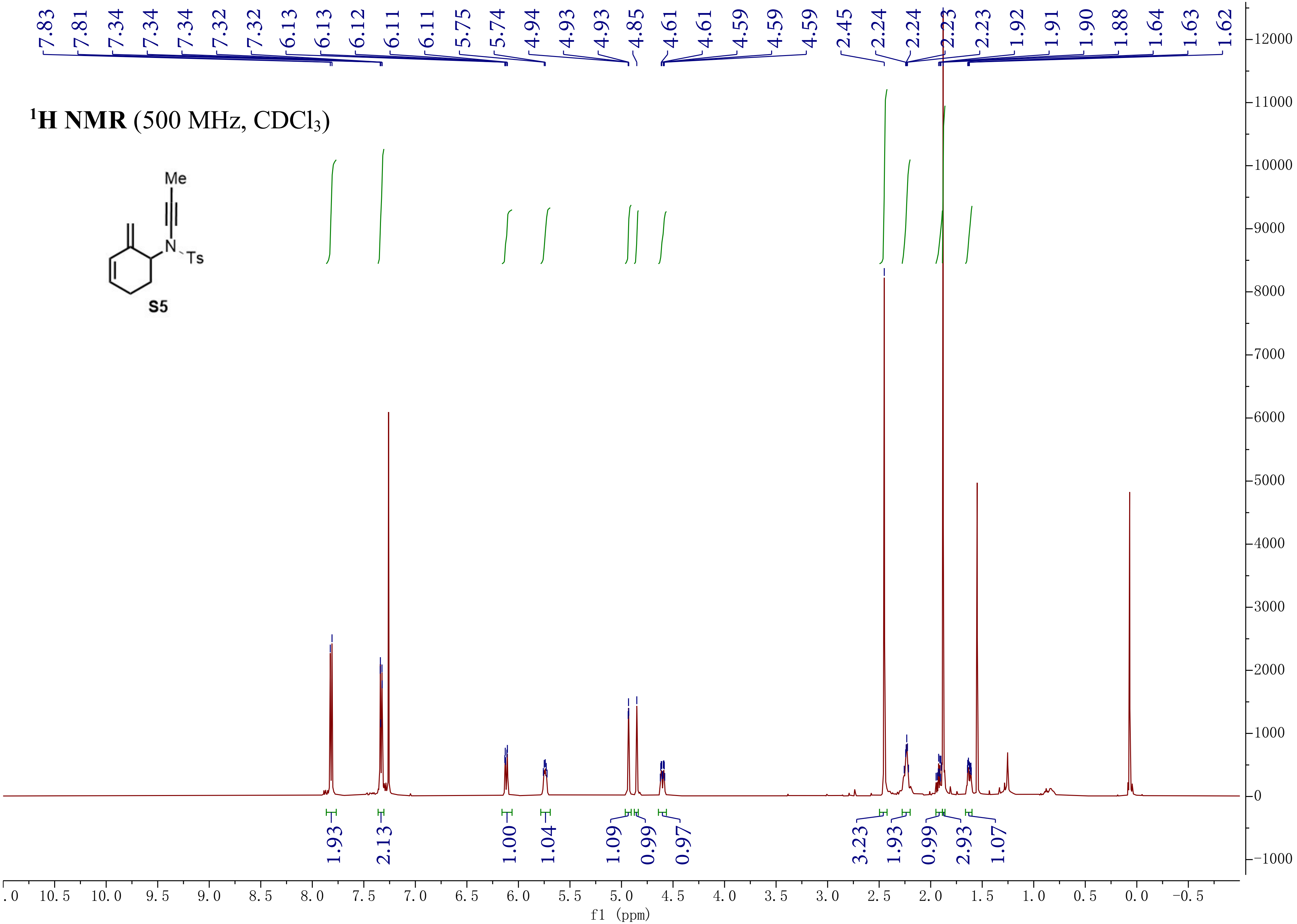

7.83
7.81
7.34
7.34
7.34
7.32
7.32
6.13
6.13
6.12
6.11
6.11
5.75
5.74
4.94
4.93
4.93
4.85
4.61
4.61
4.59
4.59
4.59
2.45
2.24
2.24
2.23
2.23
1.92
1.91
1.90
1.88
1.64
1.63
1.62
^{1}H NMR (500 MHz, $CDCl_3$)
Me
N
Ts
S5
1.93
2.13
1.00
1.04
1.09
0.99
0.97
3.23
1.93
0.99
2.93
1.07
f1 (ppm)

^{13}C NMR (125 MHz, $CDCl_3$)

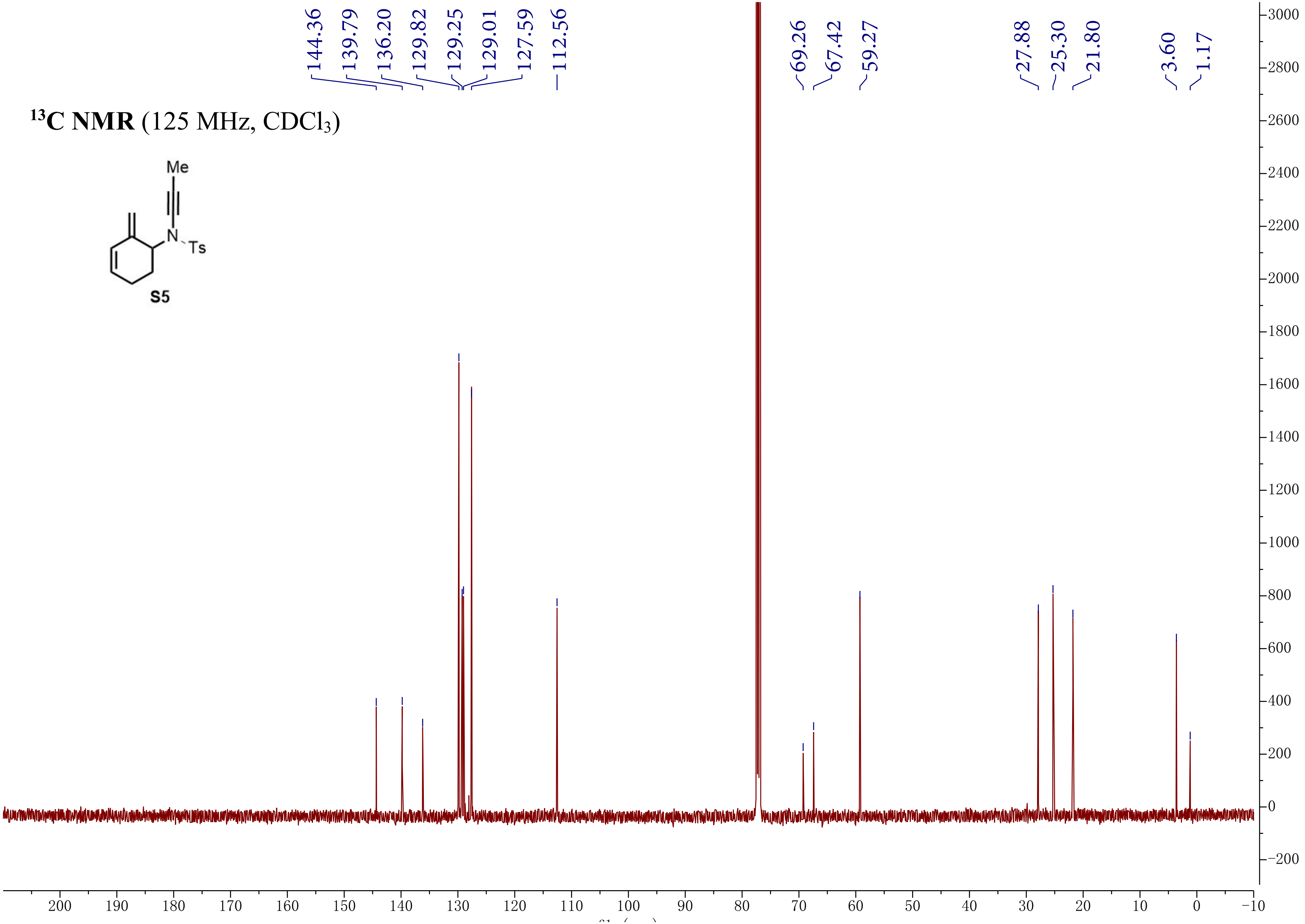

^{1}H NMR (400 MHz, $CDCl_3$)

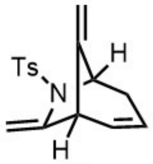

15

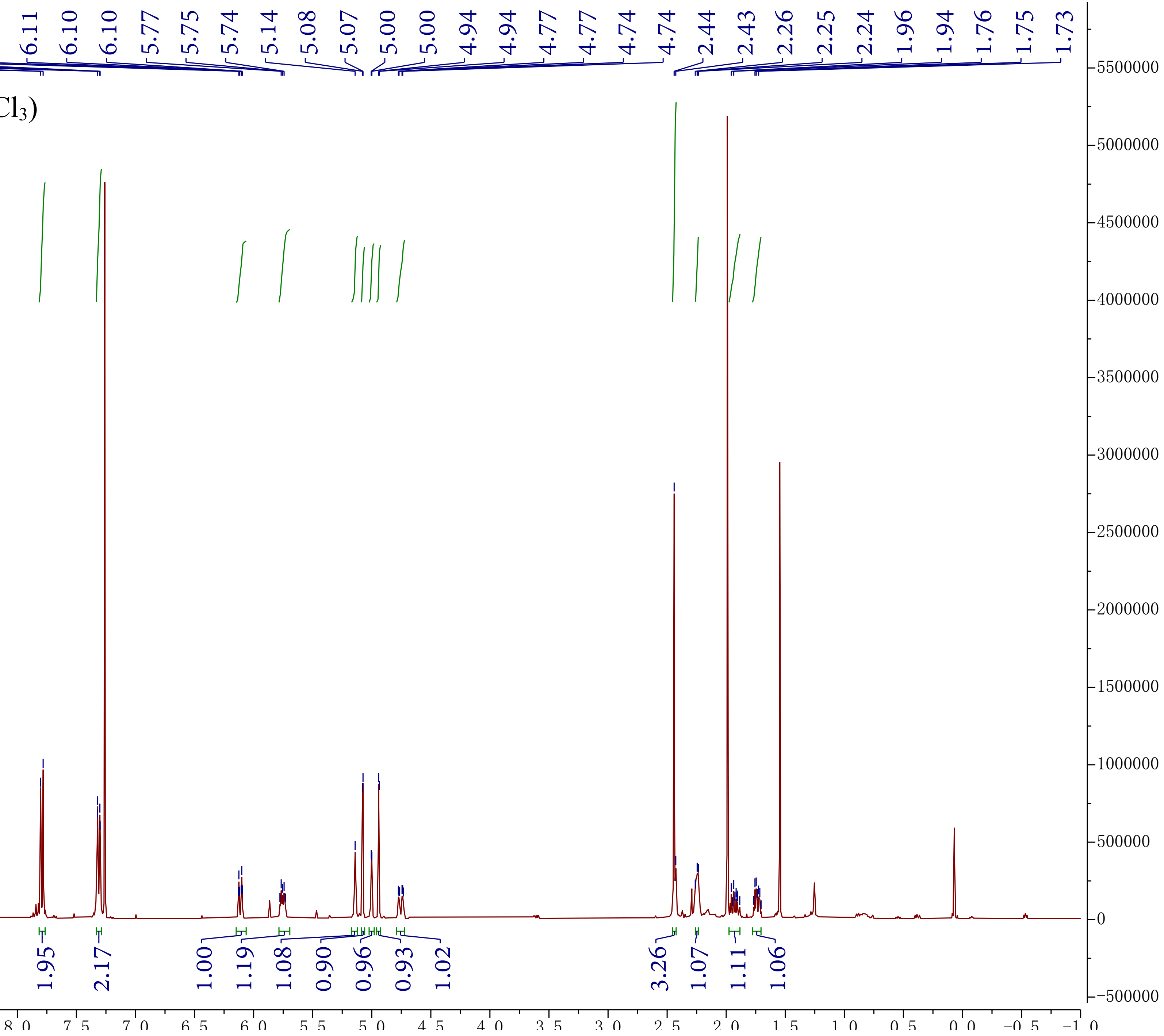

^{13}C NMR (100 MHz, $CDCl_3$)

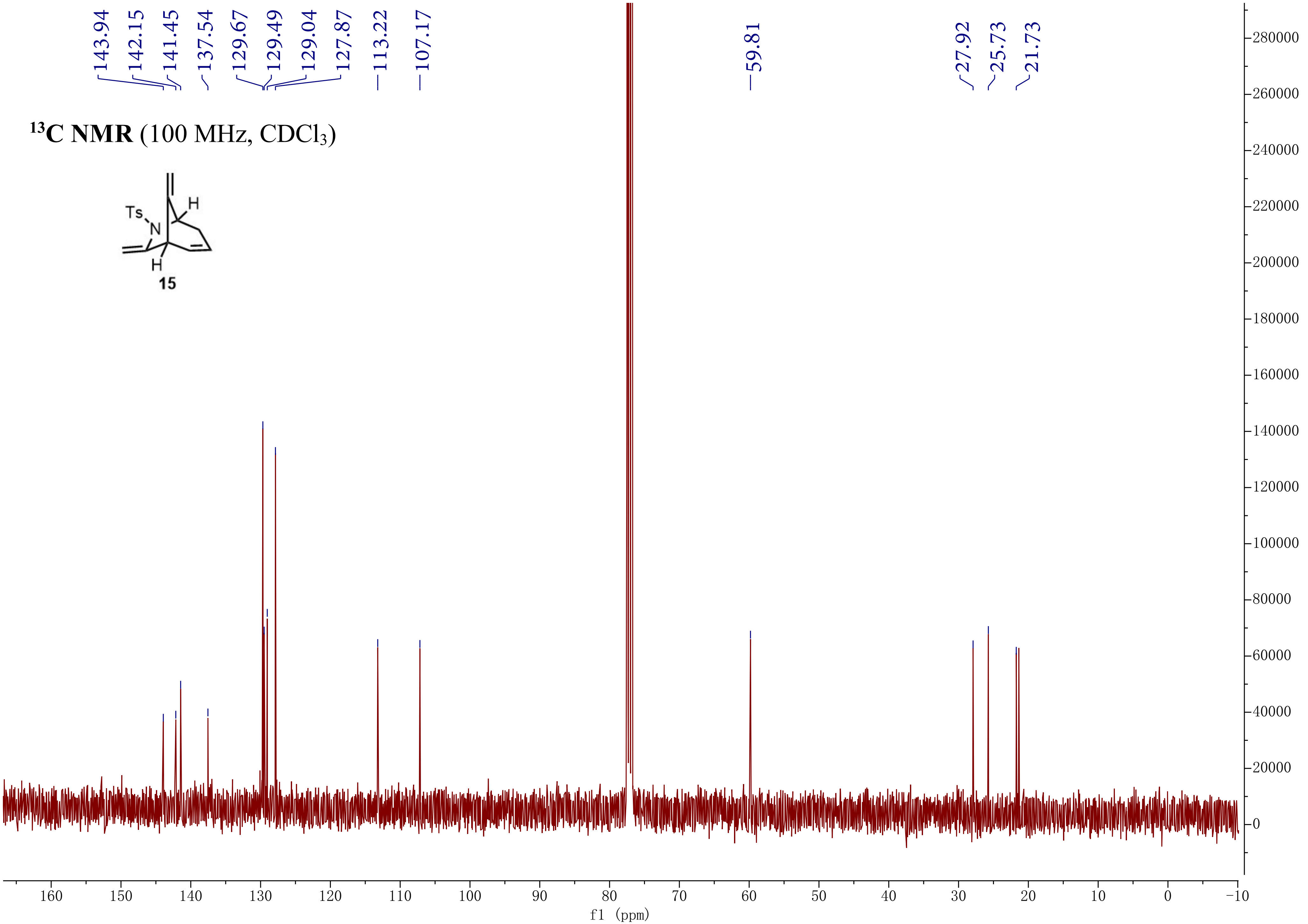

15

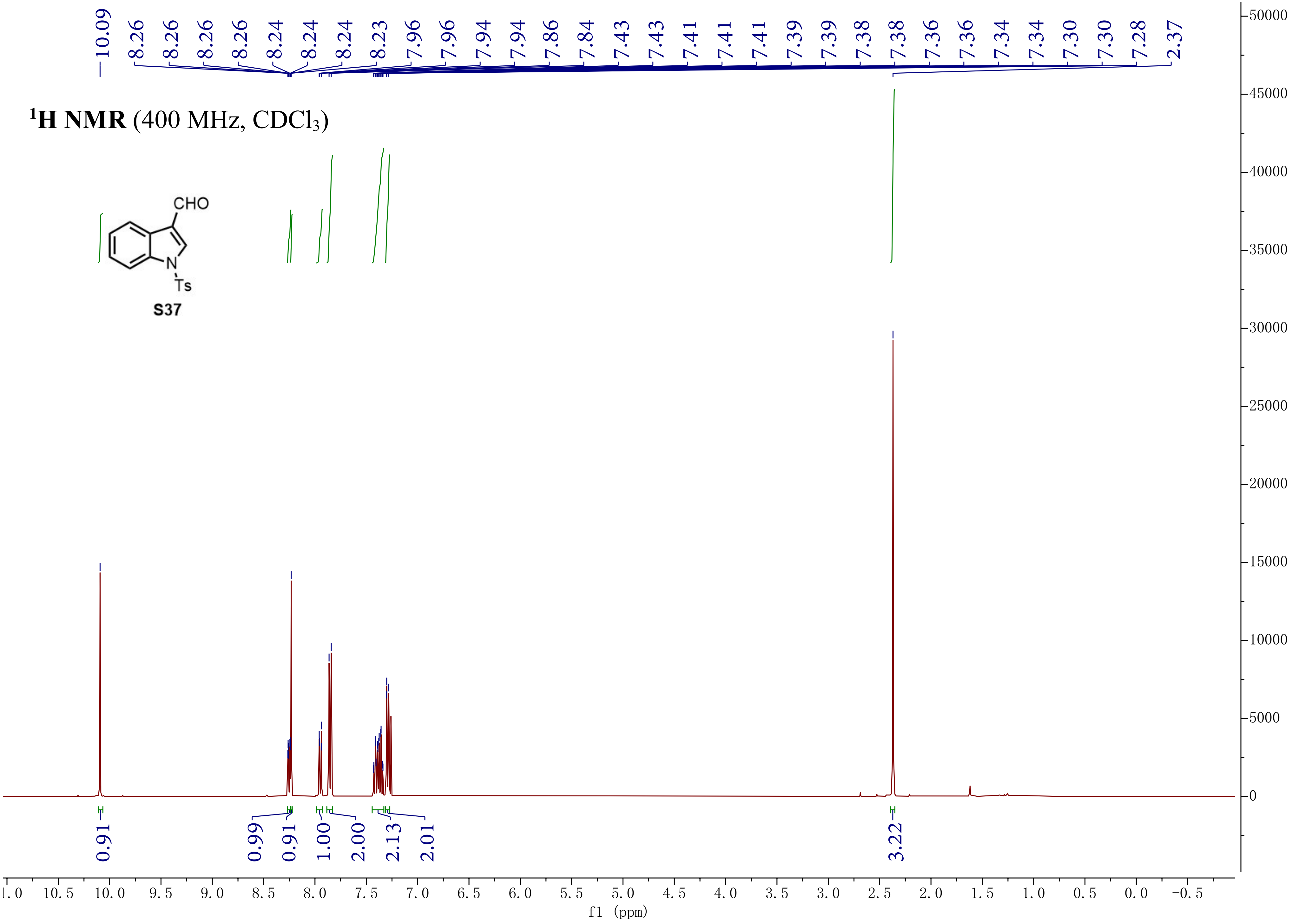

^{1}H NMR (400 MHz, $CDCl_3$)
CHO
N
Ts
S37
10.09
8.26 8.26 8.26 8.26 8.24 8.24 8.24 8.23 7.96 7.96 7.94 7.94 7.86 7.84 7.43 7.43 7.41 7.41 7.41 7.39 7.39 7.38 7.38 7.36 7.36 7.34 7.34 7.30 7.30 7.28 2.37
0.91
0.99
0.91
1.00
2.00
2.13
2.01
3.22
50000
45000
40000
35000
30000
25000
20000
15000
10000
5000
0
1.0 10.5 10.0 9.5 9.0 8.5 8.0 7.5 7.0 6.5 6.0 5.5 5.0 4.5 4.0 3.5 3.0 2.5 2.0 1.5 1.0 0.5 0.0 -0.5
f1 (ppm)

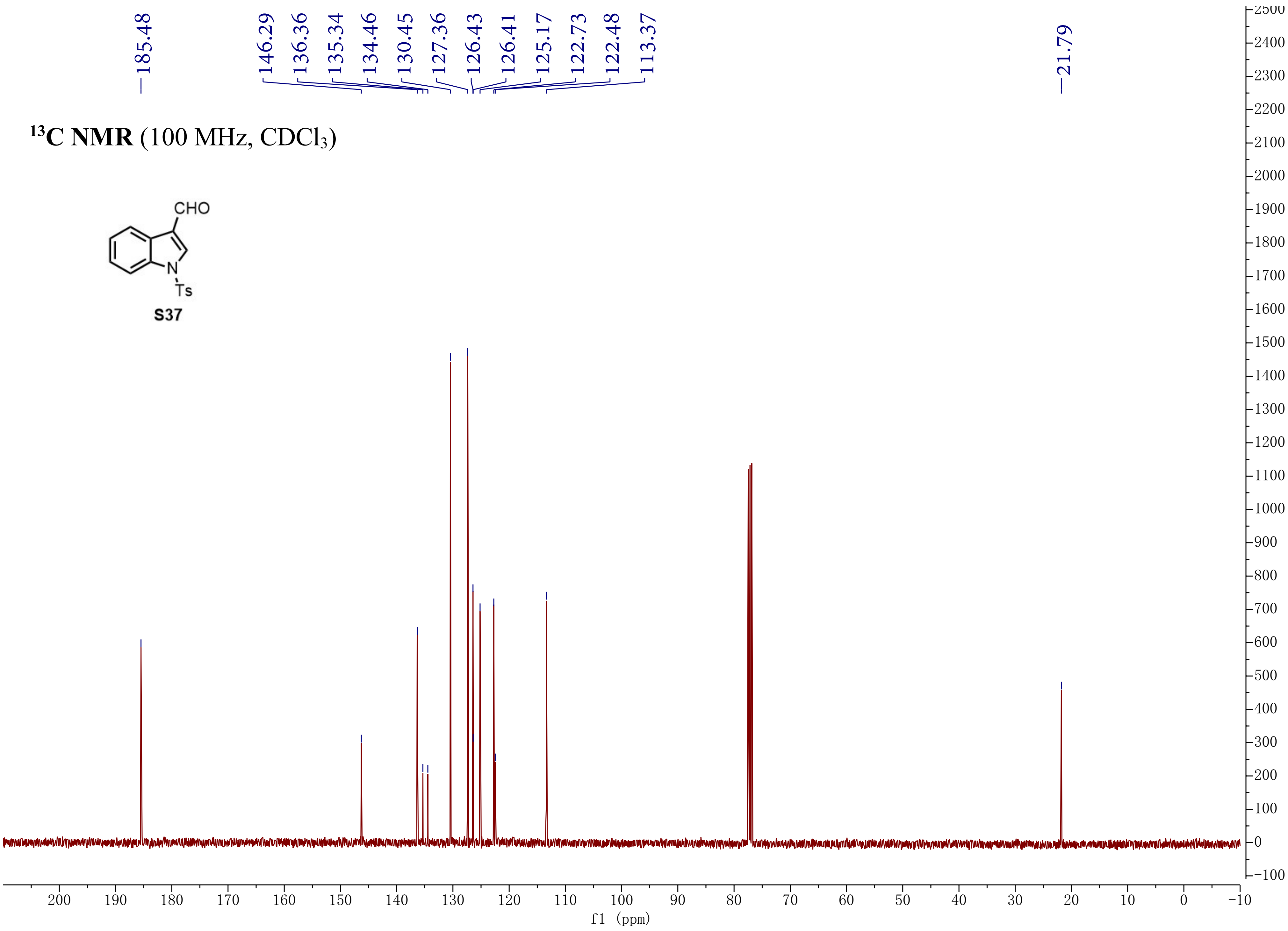
185.48
146.29
136.36
135.34
134.46
130.45
127.36
126.43
126.41
125.17
122.73
122.48
113.37
21.79
^{13}C NMR (100 MHz, $CDCl_3$)
CHO
N
Ts
S37
f1 (ppm)

^{1}H NMR (400 MHz, $CDCl_3$)

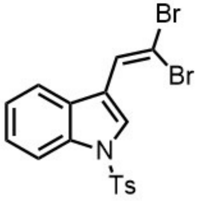

S38

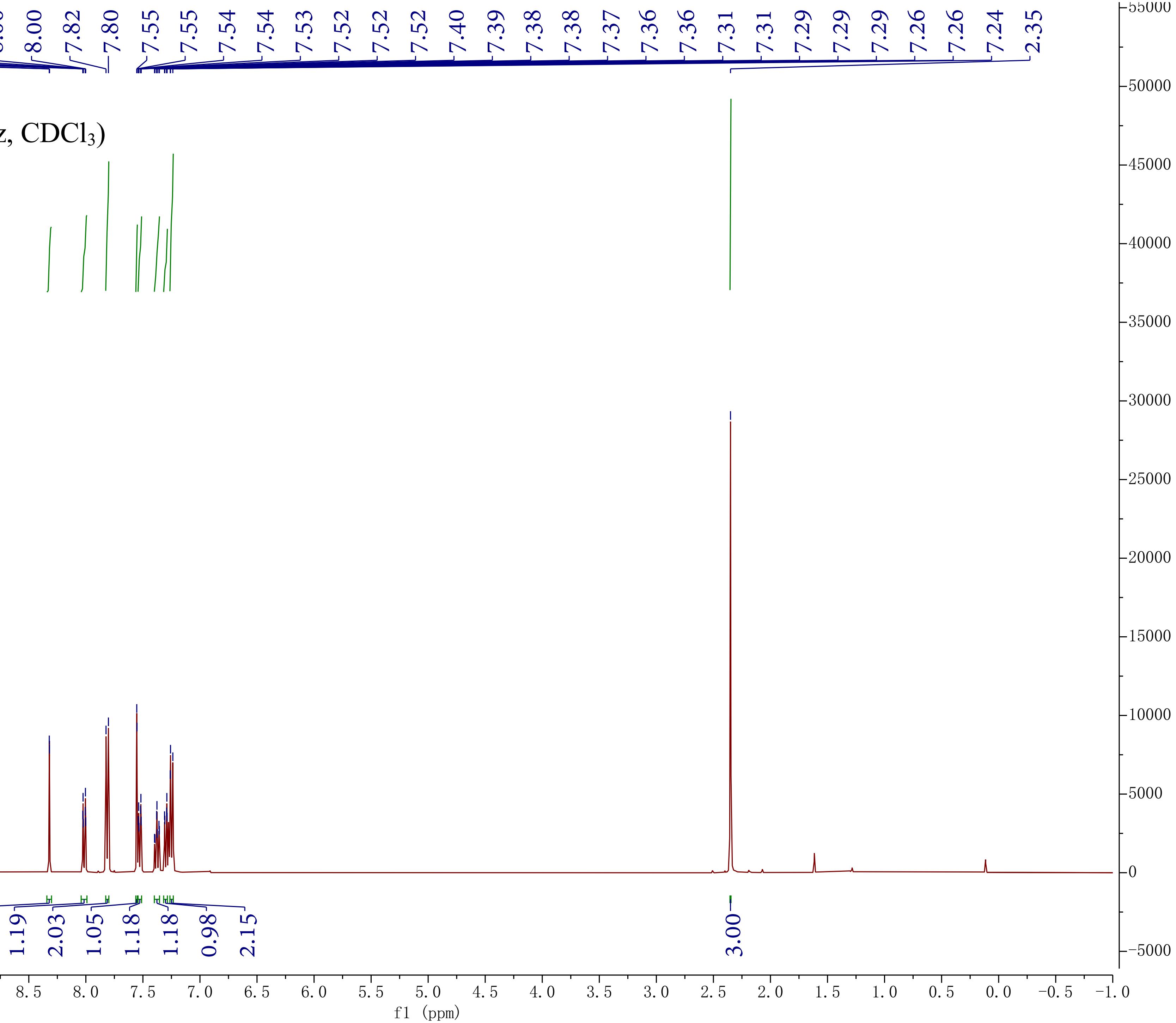

^{13}C NMR (100 MHz, $CDCl_3$)

S38

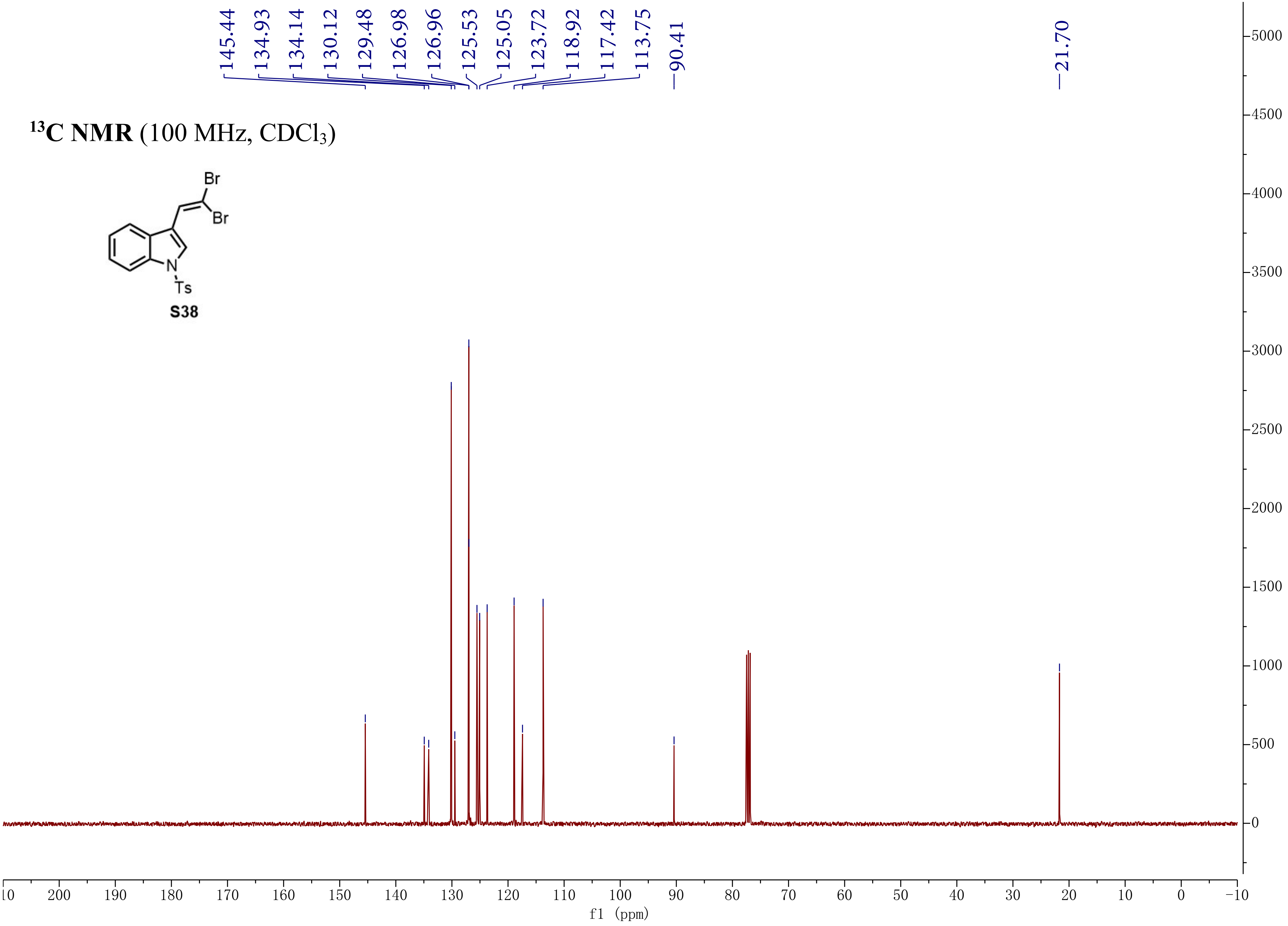

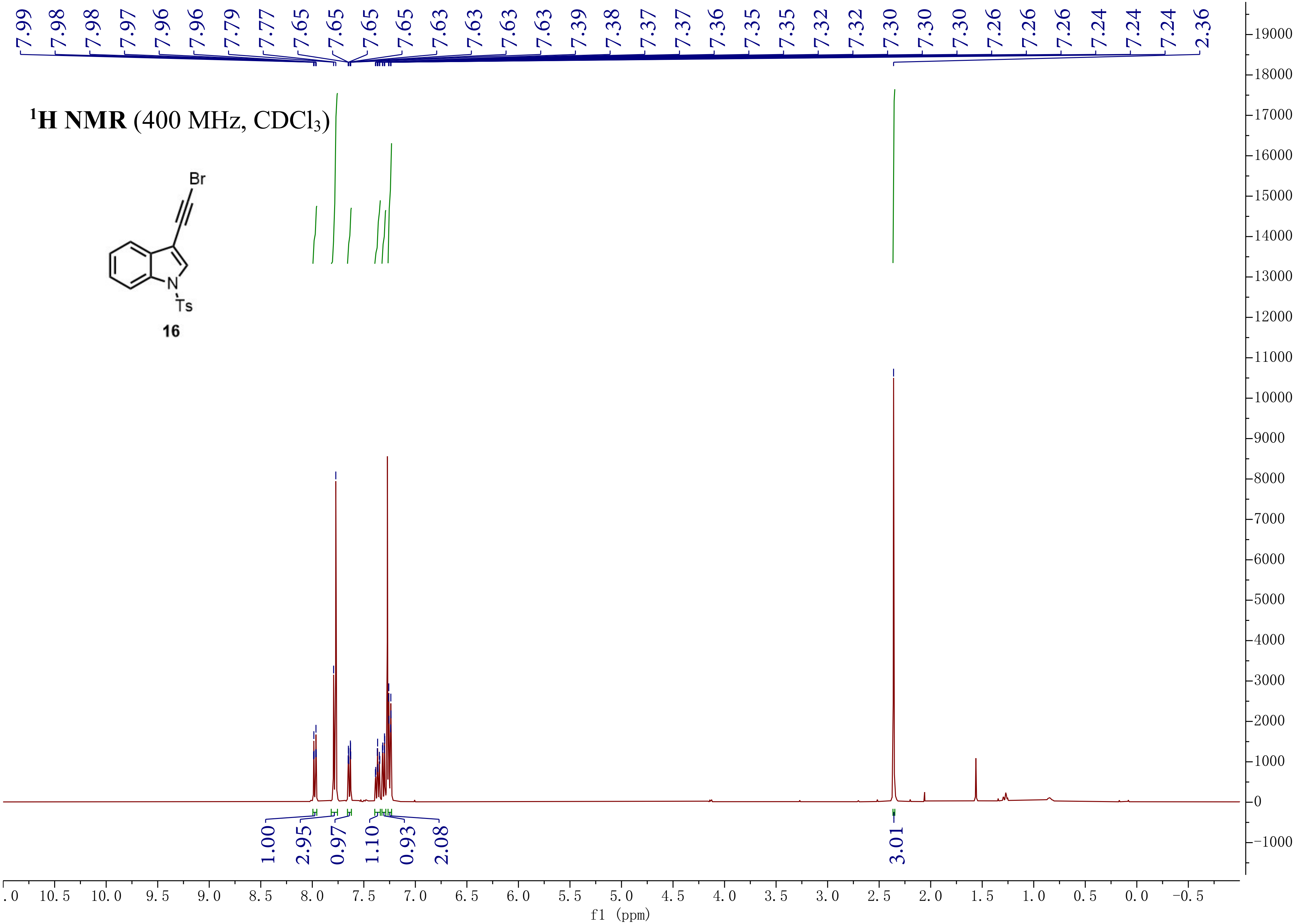

7.99 7.98 7.98 7.97 7.96 7.96 7.79 7.77 7.65 7.65 7.65 7.65 7.63 7.63 7.63 7.63 7.39 7.38 7.37 7.37 7.36 7.35 7.35 7.32 7.32 7.30 7.30 7.30 7.26 7.26 7.26 7.24 7.24 7.24 2.36
1H NMR (400 MHz, CDCl3)
Br
N
Ts
16
1.00 2.95 0.97 1.10 0.93 2.08 3.01
f1 (ppm)

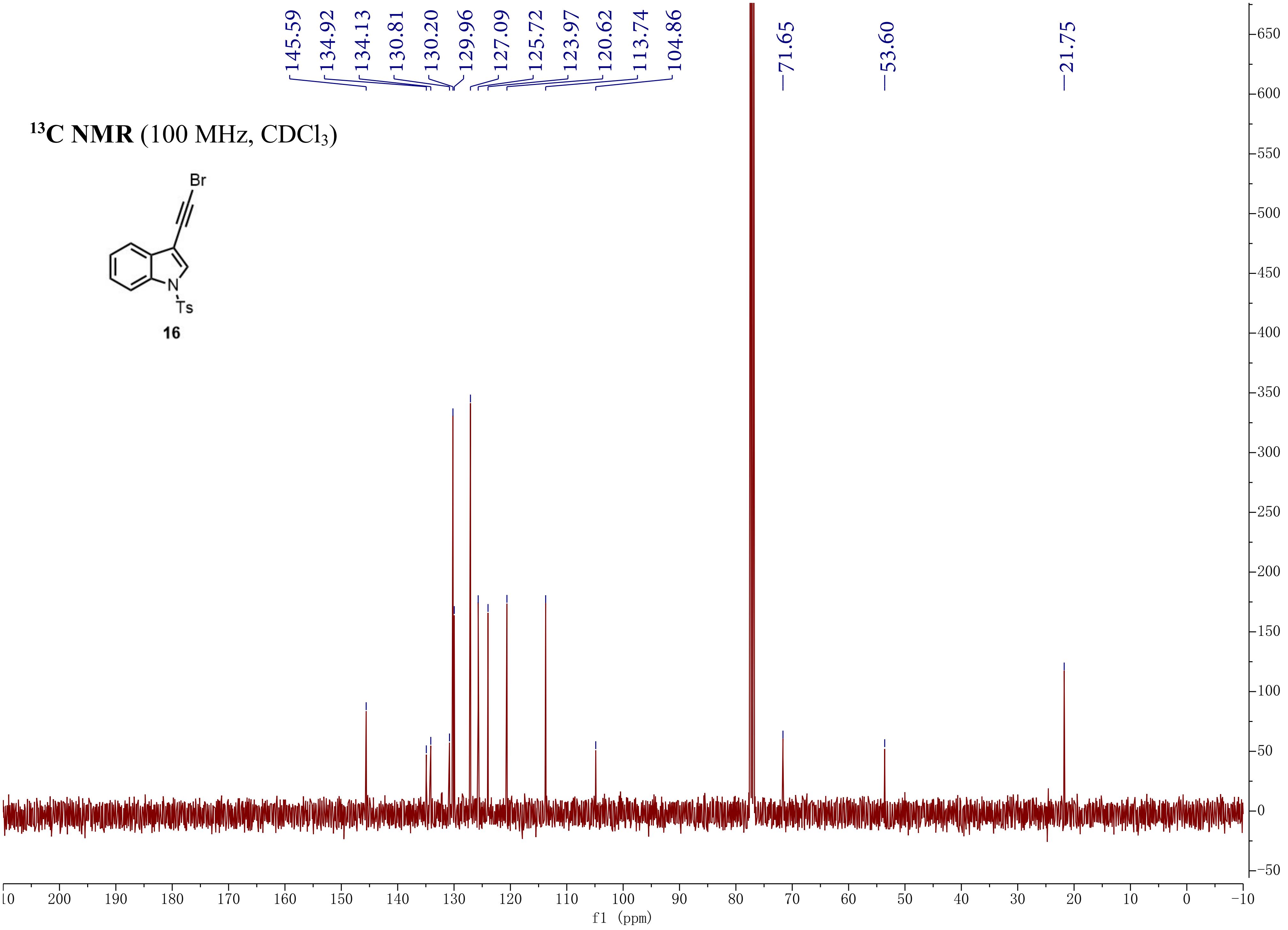
13C NMR (100 MHz, CDCl3)
Br
N
Ts
16
145.59
134.92
134.13
130.81
130.20
129.96
127.09
125.72
123.97
120.62
113.74
104.86
71.65
53.60
21.75
650
600
550
500
450
400
350
300
250
200
150
100
50
0
−50
10
200
190
180
170
160
150
140
130
120
110
100
90
80
70
60
50
40
30
20
10
0
−10
f1 (ppm)

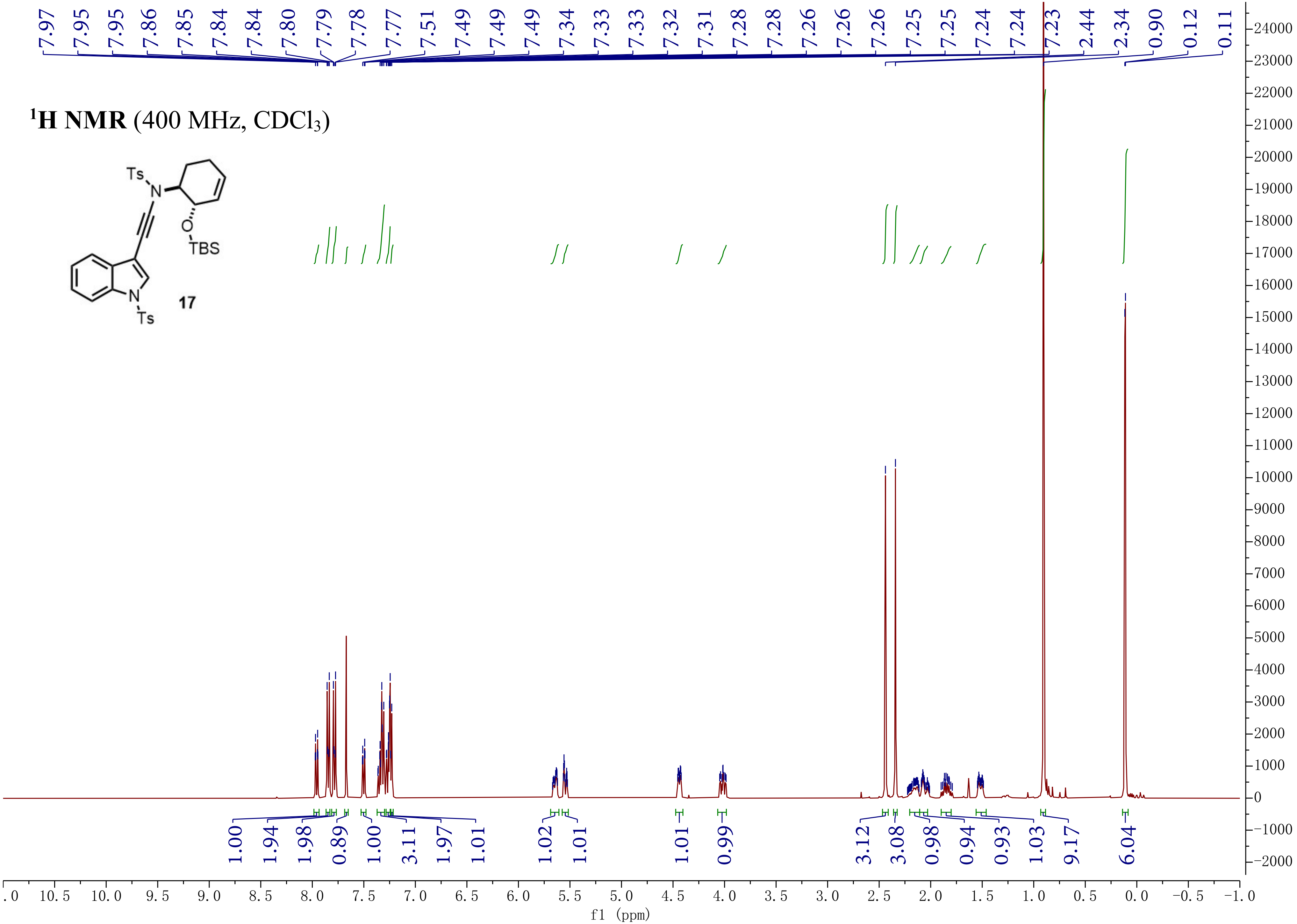

1H NMR (400 MHz, CDCl3)
Ts
N
O
TBS
N
Ts
17
7.97 7.95 7.95 7.86 7.85 7.84 7.84 7.80 7.79 7.78 7.77 7.51 7.49 7.49 7.49 7.34 7.33 7.33 7.32 7.31 7.28 7.28 7.26 7.26 7.26 7.25 7.25 7.24 7.24 7.23 2.44 2.34 0.90 0.12 0.11
1.00 1.94 1.98 0.89 1.00 3.11 1.97 1.01 1.02 1.01 1.01 0.99 3.12 3.08 0.98 0.94 0.93 1.03 9.17 6.04
f1 (ppm)

^{13}C NMR (100 MHz, $CDCl_3$)

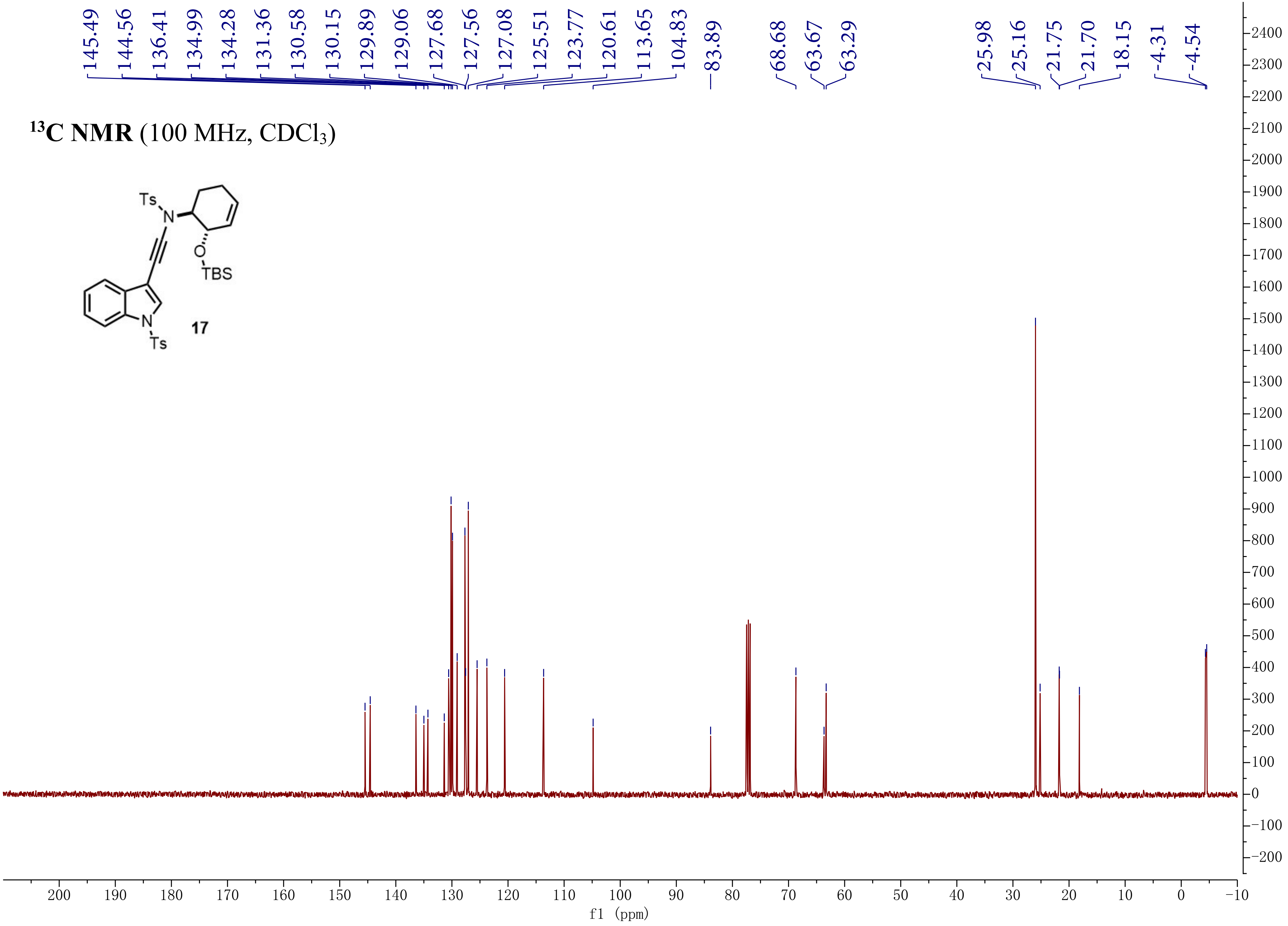

17

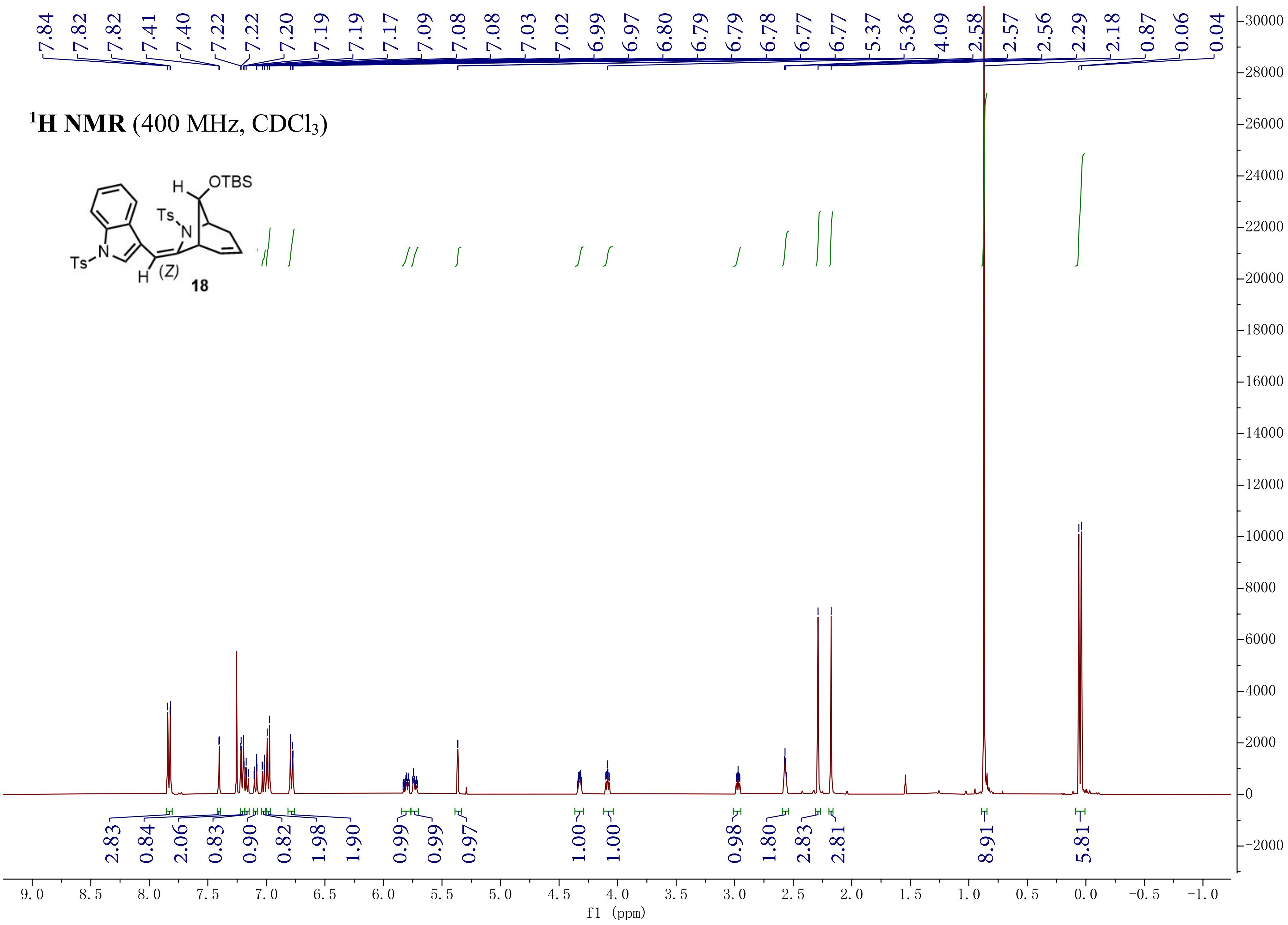
7.84 7.82 7.82 7.41 7.40 7.22 7.22 7.20 7.19 7.19 7.17 7.09 7.08 7.08 7.03 7.02 6.99 6.97 6.80 6.79 6.79 6.78 6.77 6.77 5.37 5.36 4.09 2.58 2.57 2.56 2.29 2.18 0.87 0.06 0.04
¹H NMR (400 MHz, CDCl₃)
OTBS
H
Ts
N
Ts
N
H
(Z)
18
2.83 0.84 2.06 0.83 0.90 0.82 1.98 1.90 0.99 0.99 0.97 1.00 1.00 0.98 1.80 2.83 2.81 8.91 5.81
9.0 8.5 8.0 7.5 7.0 6.5 6.0 5.5 5.0 4.5 4.0 3.5 3.0 2.5 2.0 1.5 1.0 0.5 0.0 -0.5 -1.0
f1 (ppm)
30000 28000 26000 24000 22000 20000 18000 16000 14000 12000 10000 8000 6000 4000 2000 0 -2000

144.86, 144.73, 143.31, 136.35, 135.84, 134.14, 129.88, 129.34, 127.25, 126.84, 126.46, 125.77, 125.51, 124.00, 122.60, 120.24, 117.40, 113.24, 95.63, 68.09, 61.97, 48.33, 29.37, 25.77, 21.67, 21.48, 18.10, -4.75, -4.80

^{13}C NMR (100 MHz, $CDCl_3$)

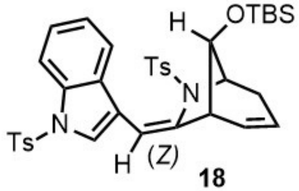

18

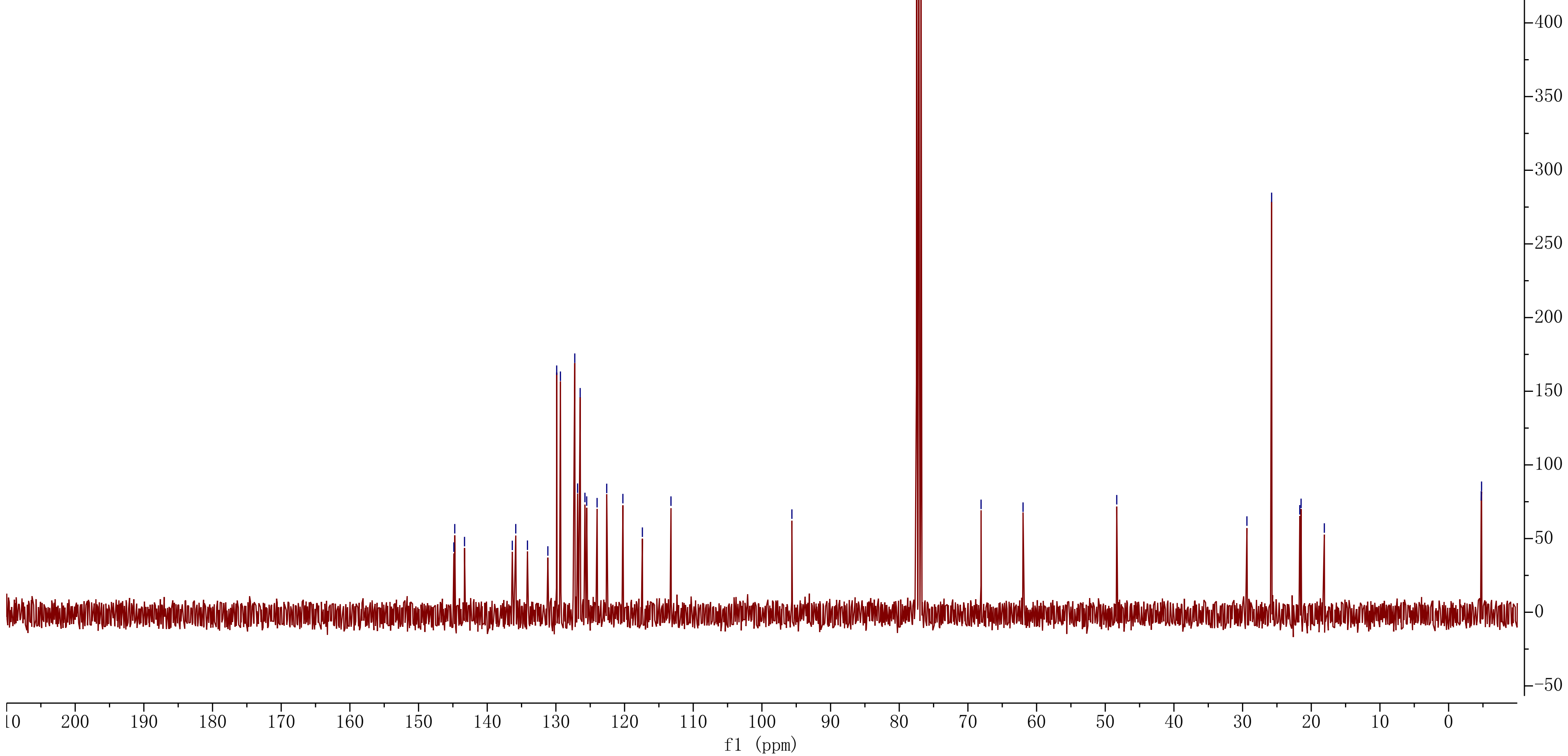

^{1}H NMR (400 MHz, $CDCl_3$)

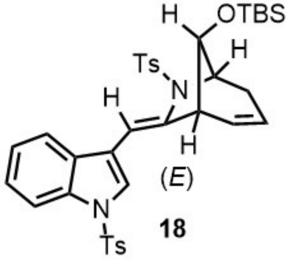

18

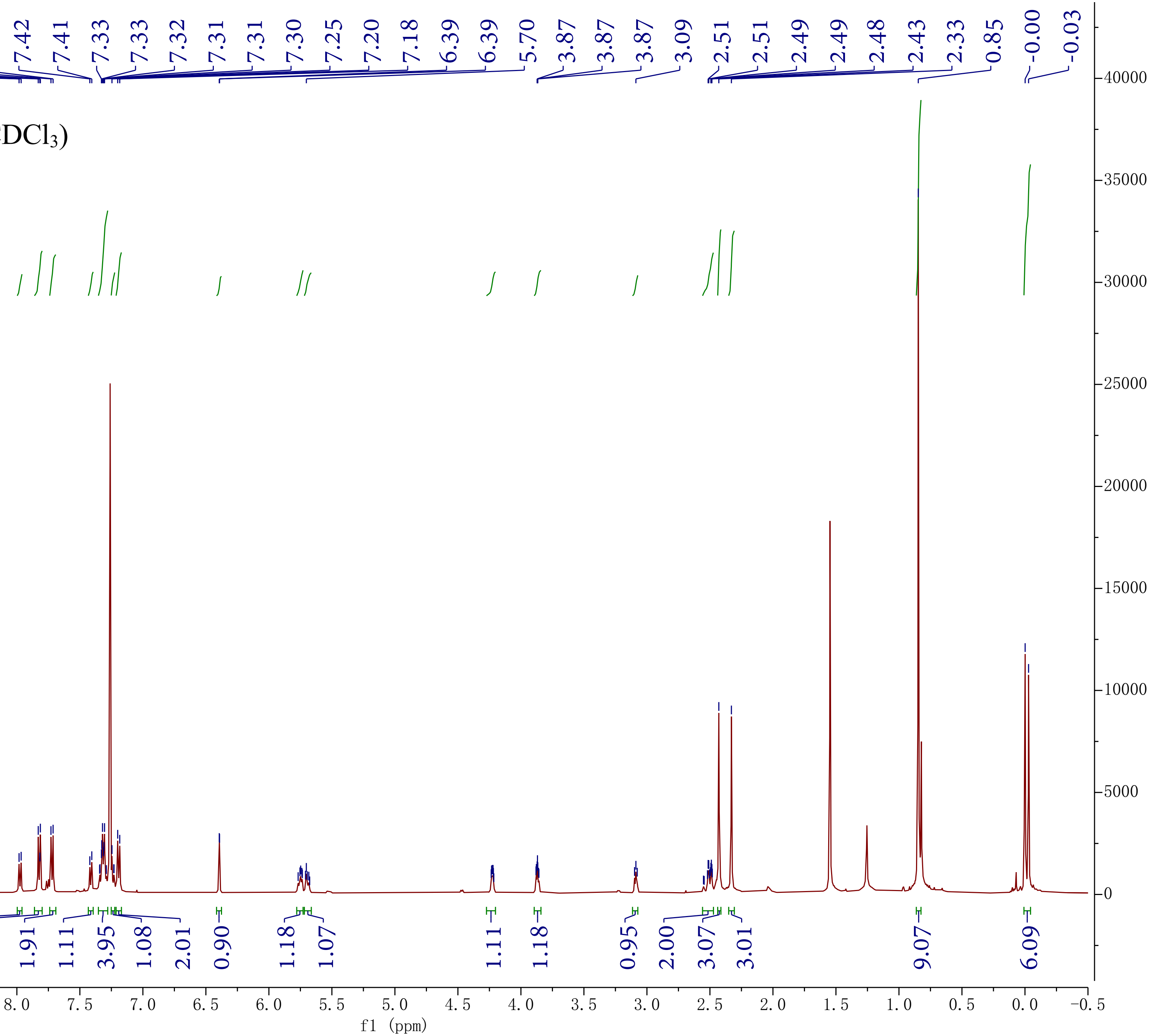

^{13}C NMR (100 MHz, $CDCl_3$)

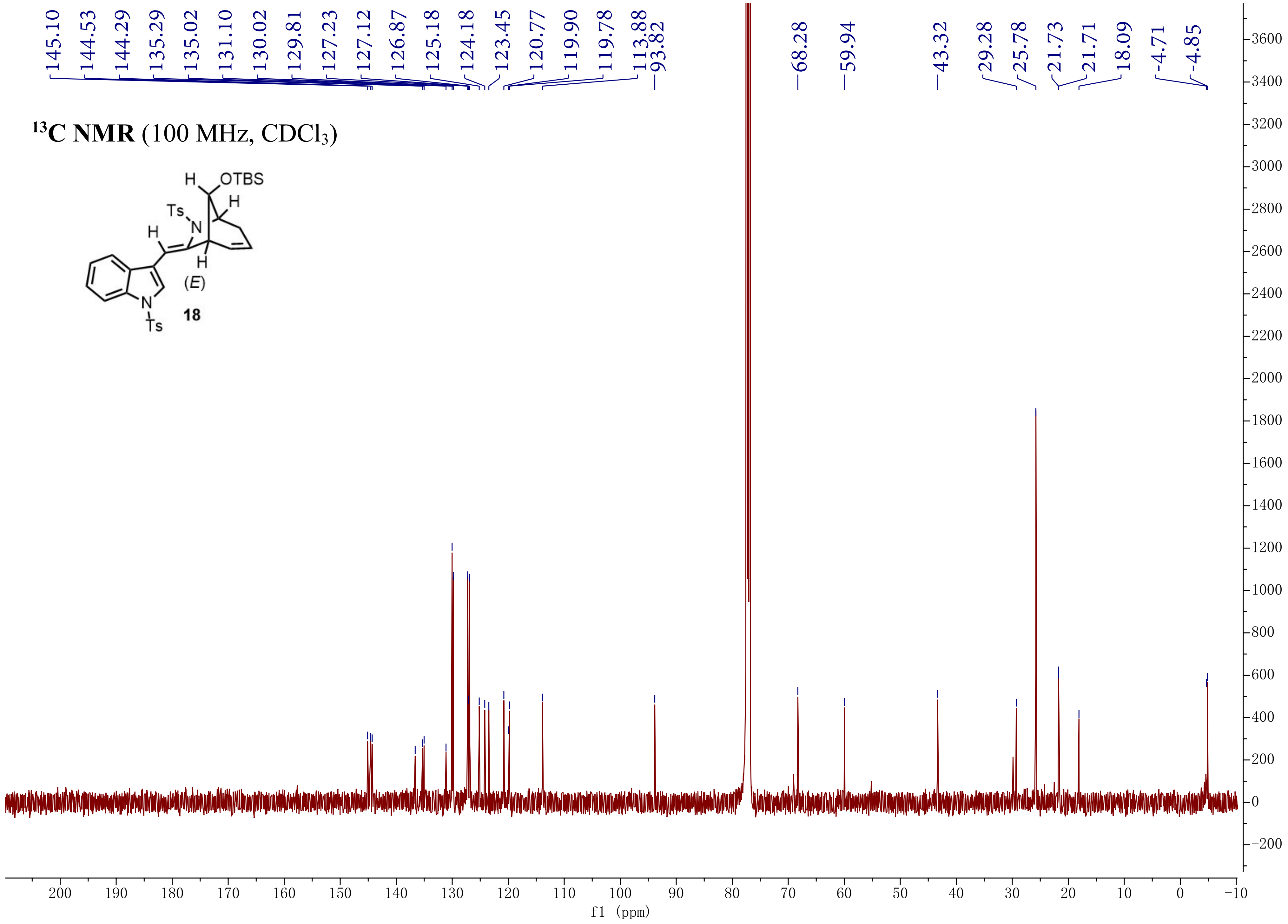

^{1}H NMR (400 MHz, $CDCl_3$)

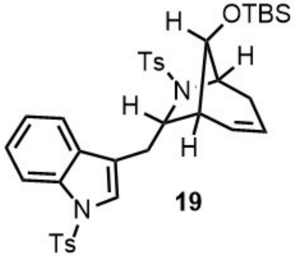

19

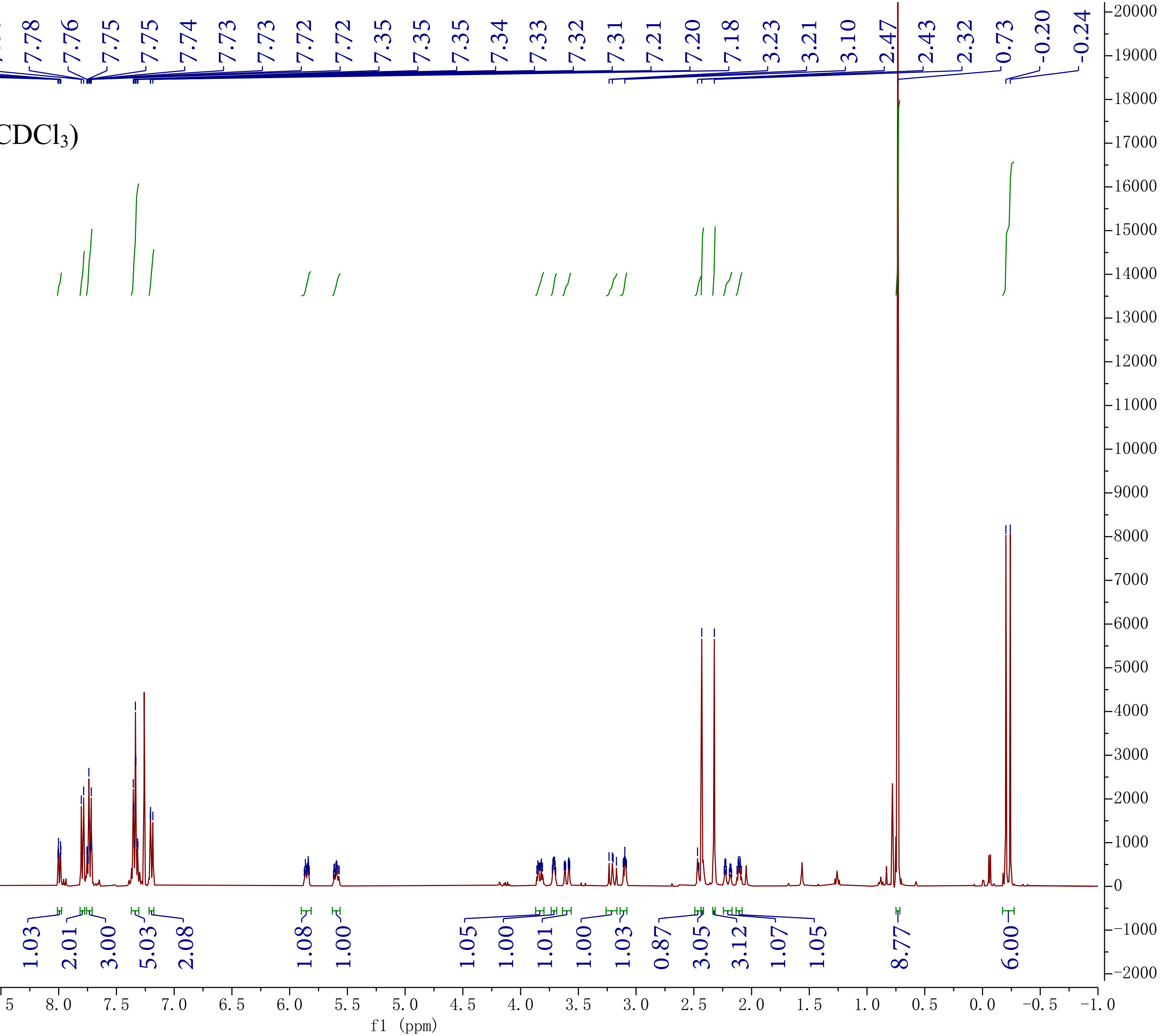

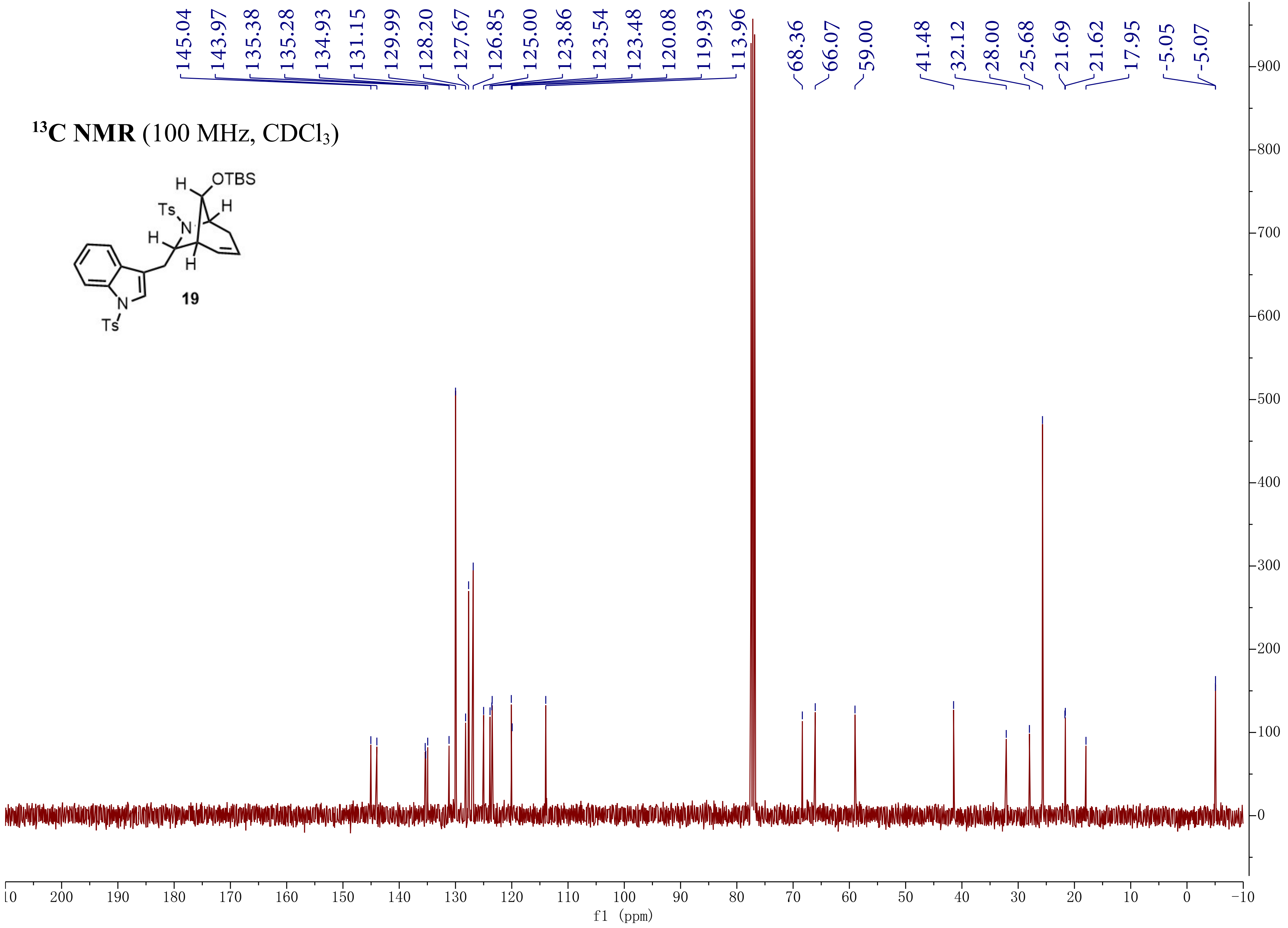

^{13}C NMR (100 MHz, $CDCl_3$)
OTBS
Ts
N
H
19
145.04
143.97
135.38
135.28
134.93
131.15
129.99
128.20
127.67
126.85
125.00
123.86
123.54
123.48
120.08
119.93
113.96
68.36
66.07
59.00
41.48
32.12
28.00
25.68
21.69
21.62
17.95
-5.05
-5.07
f1 (ppm)

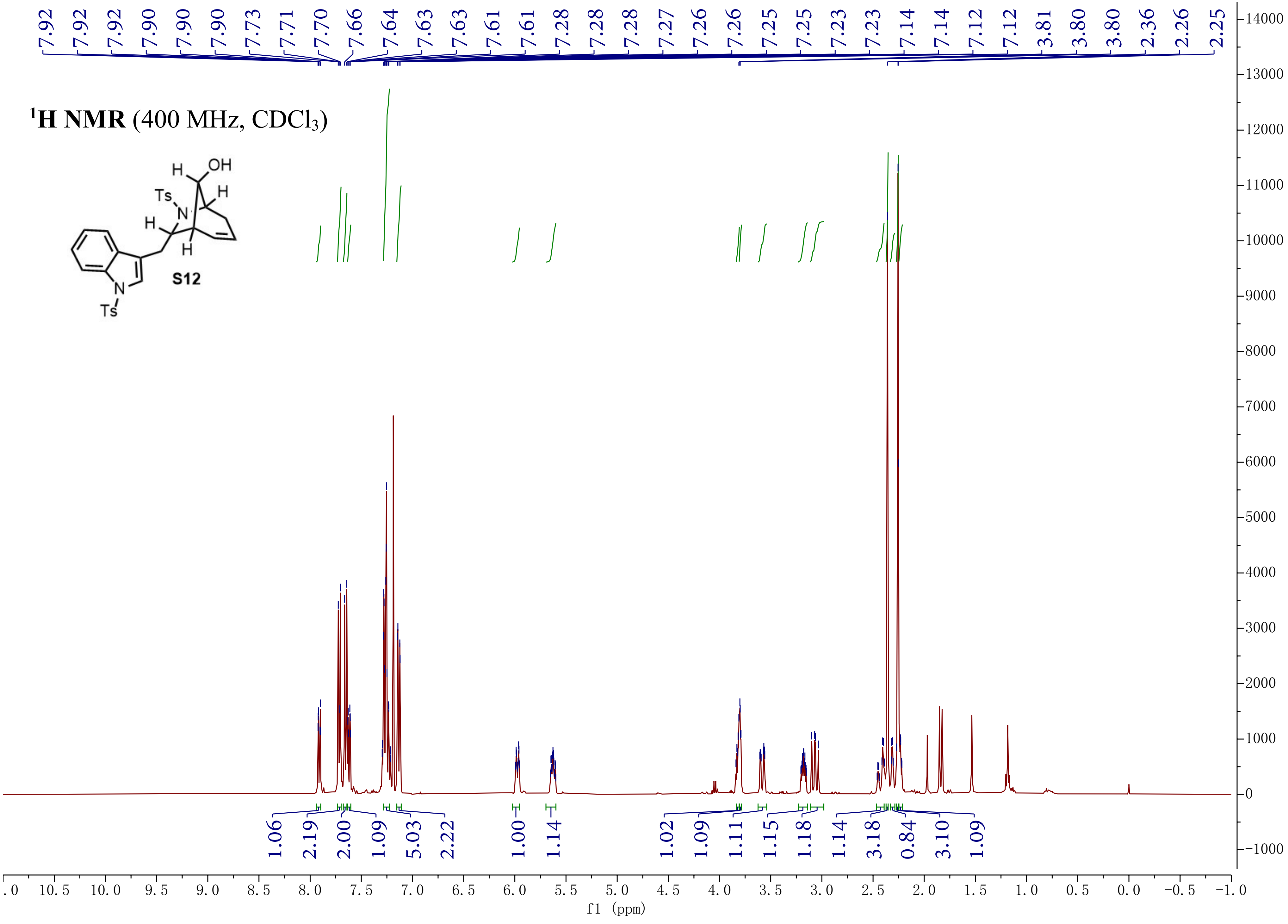

1H NMR (400 MHz, CDCl3)
OH
H
Ts
N
H
H
N
Ts
S12
7.92 7.92 7.92 7.90 7.90 7.90 7.73 7.71 7.70 7.66 7.64 7.63 7.63 7.61 7.61 7.28 7.28 7.28 7.27 7.26 7.26 7.25 7.25 7.23 7.23 7.14 7.14 7.12 7.12 3.81 3.80 3.80 2.36 2.26 2.25
1.06 2.19 2.00 1.09 5.03 2.22 1.00 1.14 1.02 1.09 1.11 1.15 1.18 1.14 3.18 0.84 3.10 1.09
f1 (ppm)

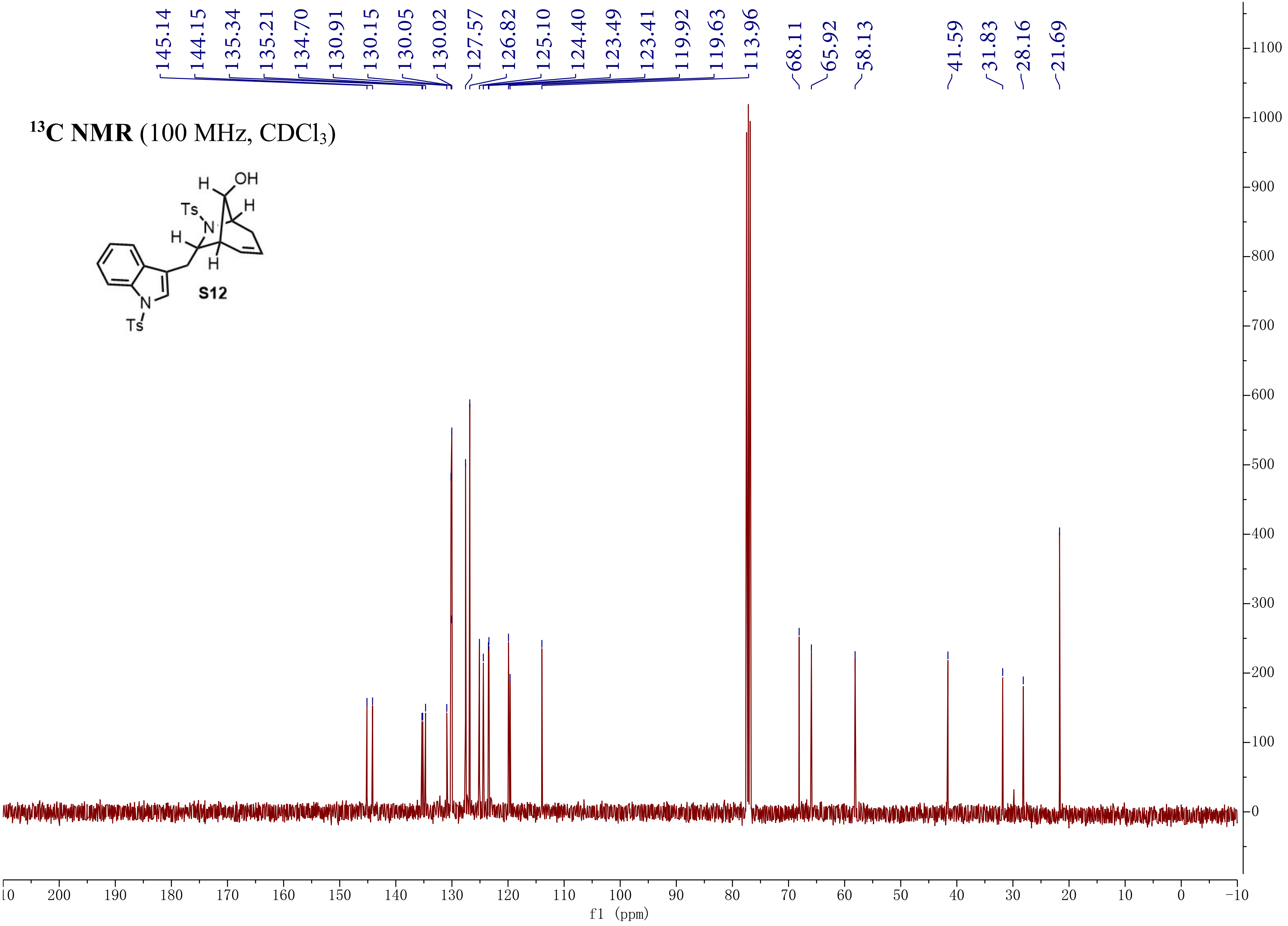

145.14
144.15
135.34
135.21
134.70
130.91
130.15
130.05
130.02
127.57
126.82
125.10
124.40
123.49
123.41
119.92
119.63
113.96
68.11
65.92
58.13
41.59
31.83
28.16
21.69
^{13}C NMR (100 MHz, $CDCl_3$)
OH
Ts
N
S12
f1 (ppm)

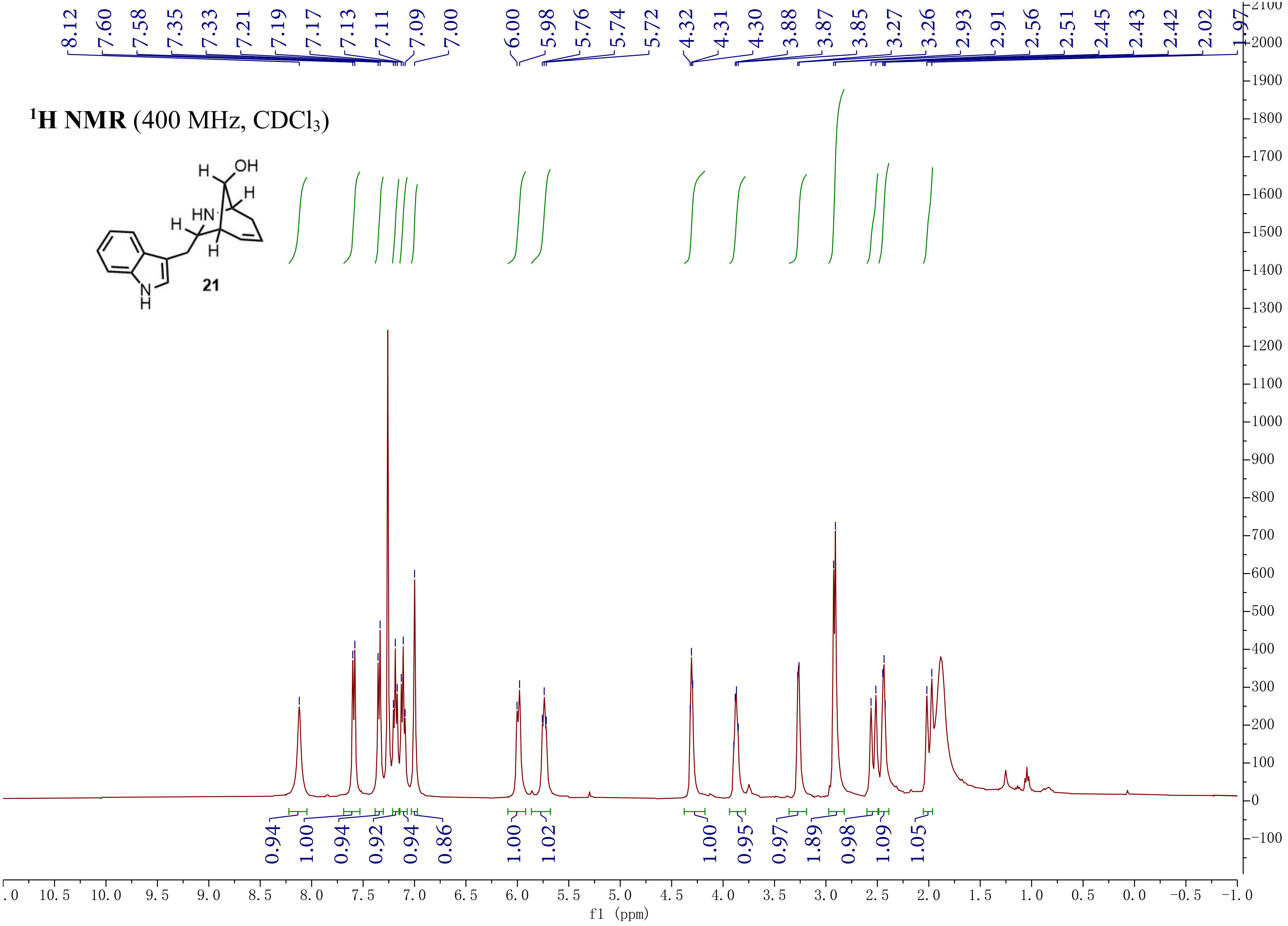

^{1}H NMR (400 MHz, $CDCl_3$)
21
8.12 7.60 7.58 7.35 7.33 7.21 7.19 7.17 7.13 7.11 7.09 7.00 6.00 5.98 5.76 5.74 5.72 4.32 4.31 4.30 3.88 3.87 3.85 3.27 3.26 2.93 2.91 2.56 2.51 2.45 2.43 2.42 2.02 1.97
0.94 1.00 0.94 0.92 0.94 0.86 1.00 1.02 1.00 0.95 0.97 1.89 0.98 1.09 1.05
f1 (ppm)

^{13}C NMR (100 MHz, $CDCl_3$)

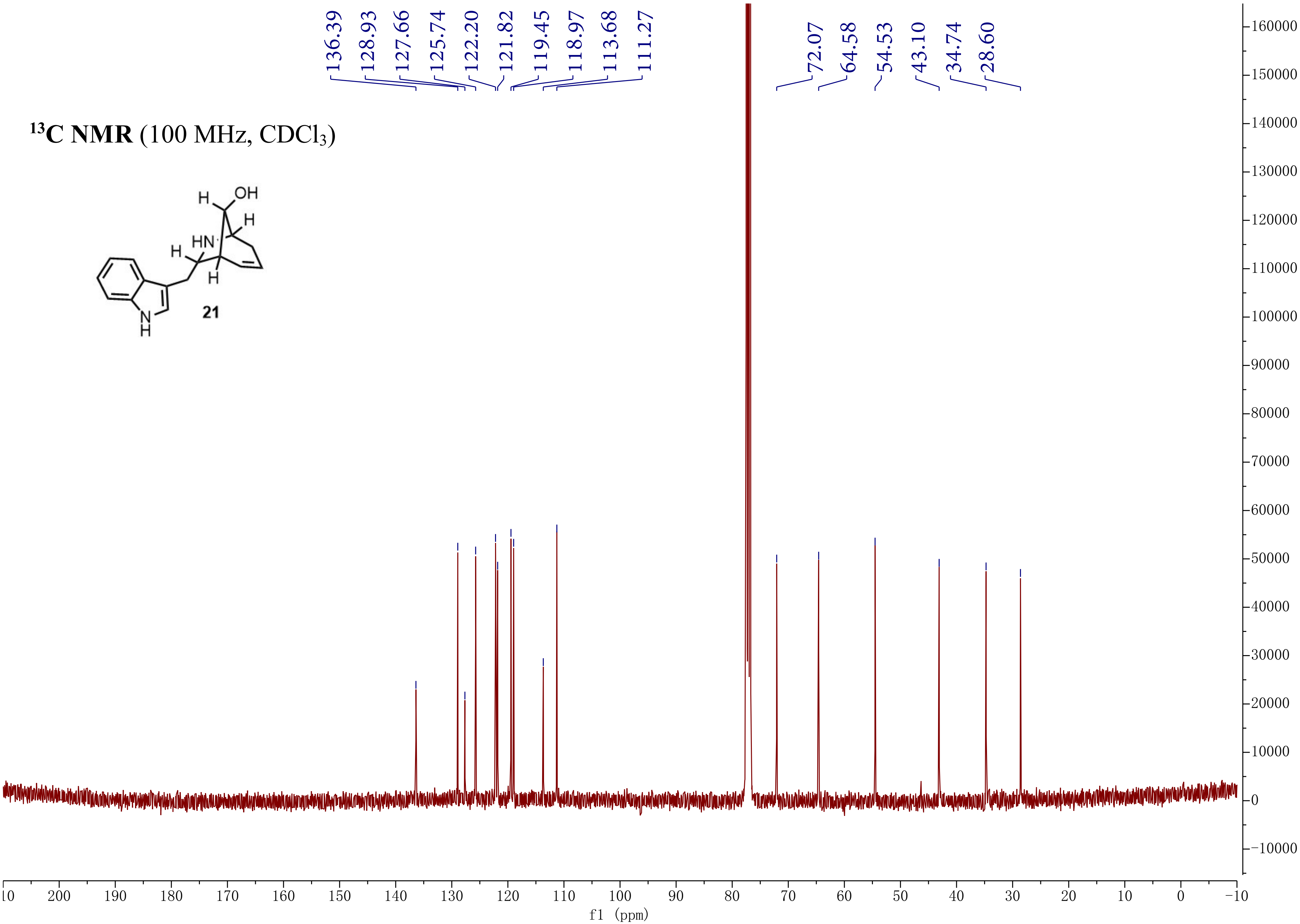

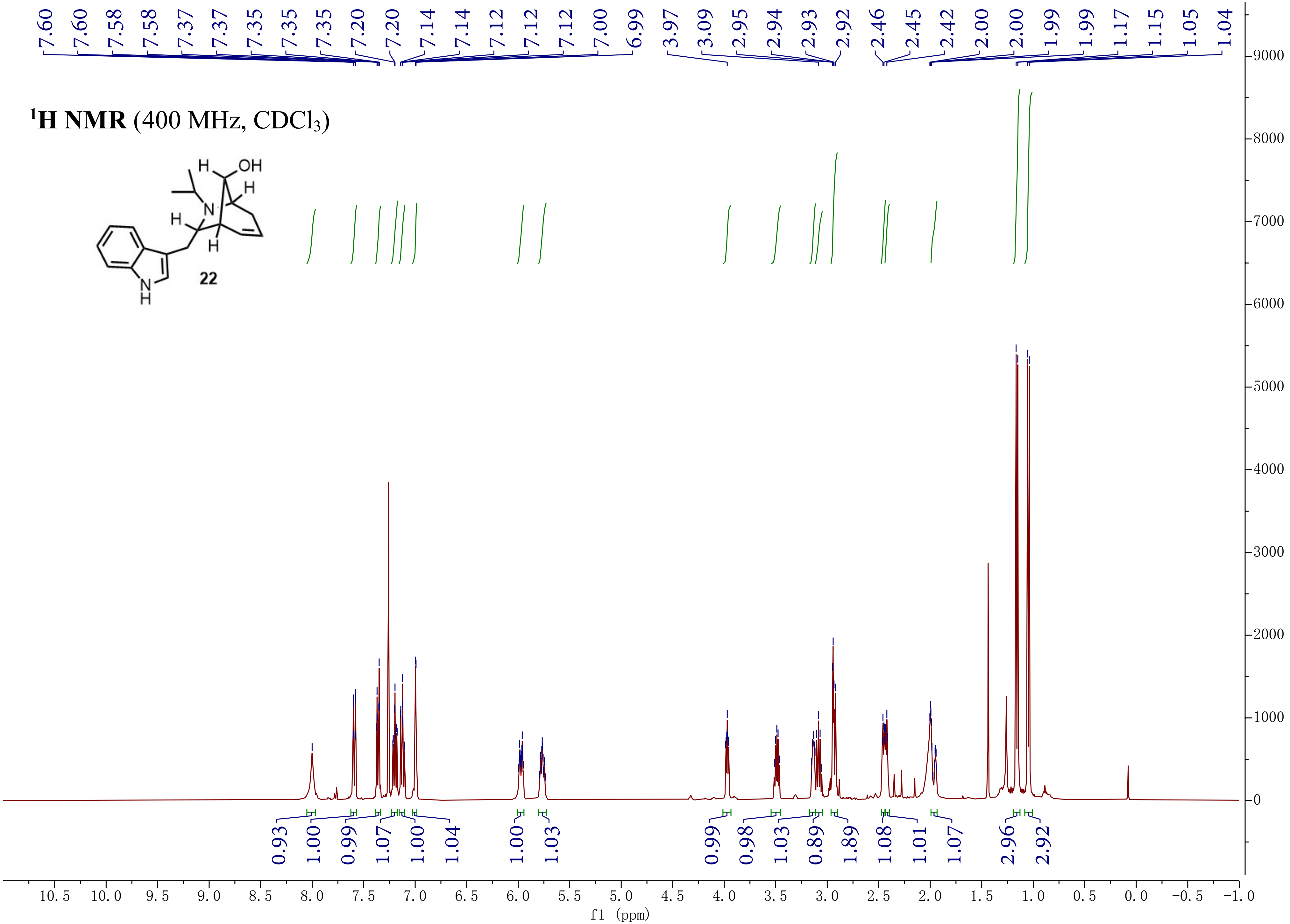

1H NMR (400 MHz, CDCl3)
H
OH
H
N
H
H
NH
22
7.60 7.60 7.58 7.58 7.37 7.37 7.35 7.35 7.35 7.20 7.20 7.14 7.14 7.12 7.12 7.12 7.00 6.99 3.97 3.09 2.95 2.94 2.93 2.92 2.46 2.45 2.42 2.00 2.00 1.99 1.99 1.17 1.15 1.05 1.04
0.93 1.00 0.99 1.07 1.00 1.04 1.00 1.03 0.99 0.98 1.03 0.89 1.89 1.08 1.01 1.07 2.96 2.92
9000 8000 7000 6000 5000 4000 3000 2000 1000 0
10.5 10.0 9.5 9.0 8.5 8.0 7.5 7.0 6.5 6.0 5.5 5.0 4.5 4.0 3.5 3.0 2.5 2.0 1.5 1.0 0.5 0.0 -0.5 -1.0
f1 (ppm)

^{13}C NMR (100 MHz, $CDCl_3$)

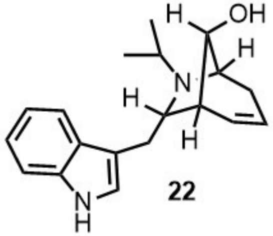

22

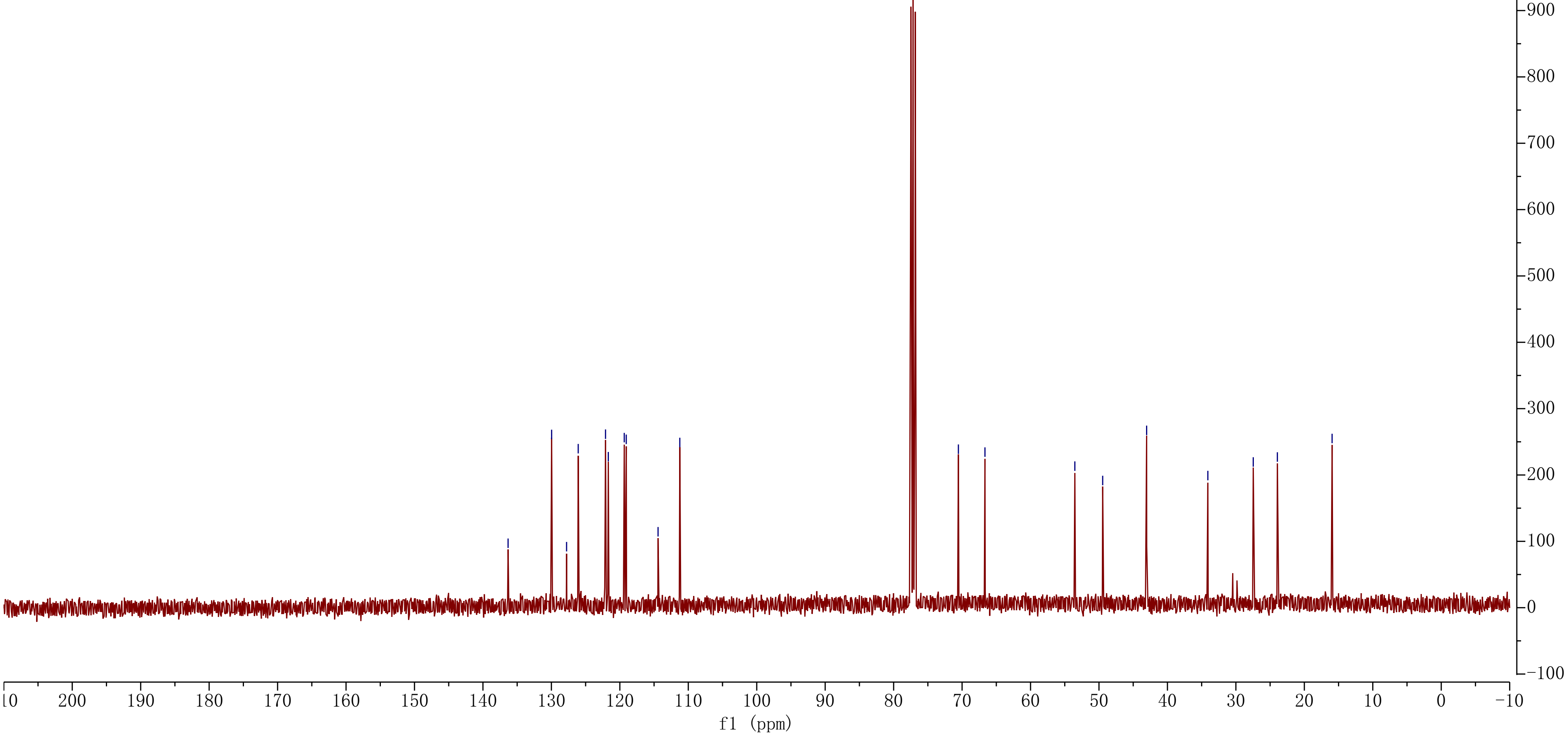

^{1}H NMR (500 MHz, $CDCl_3$)

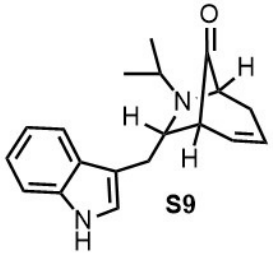

S9

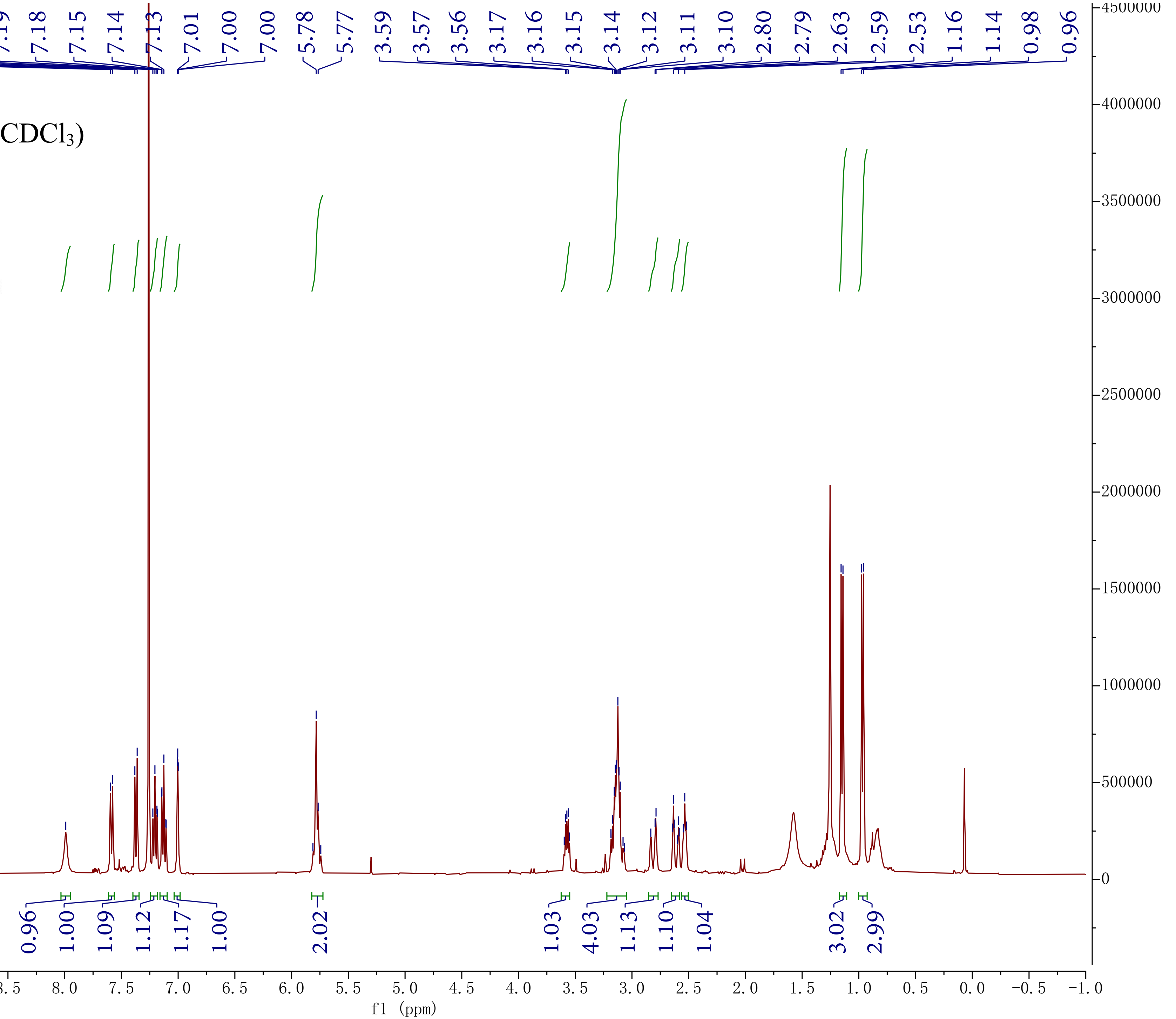

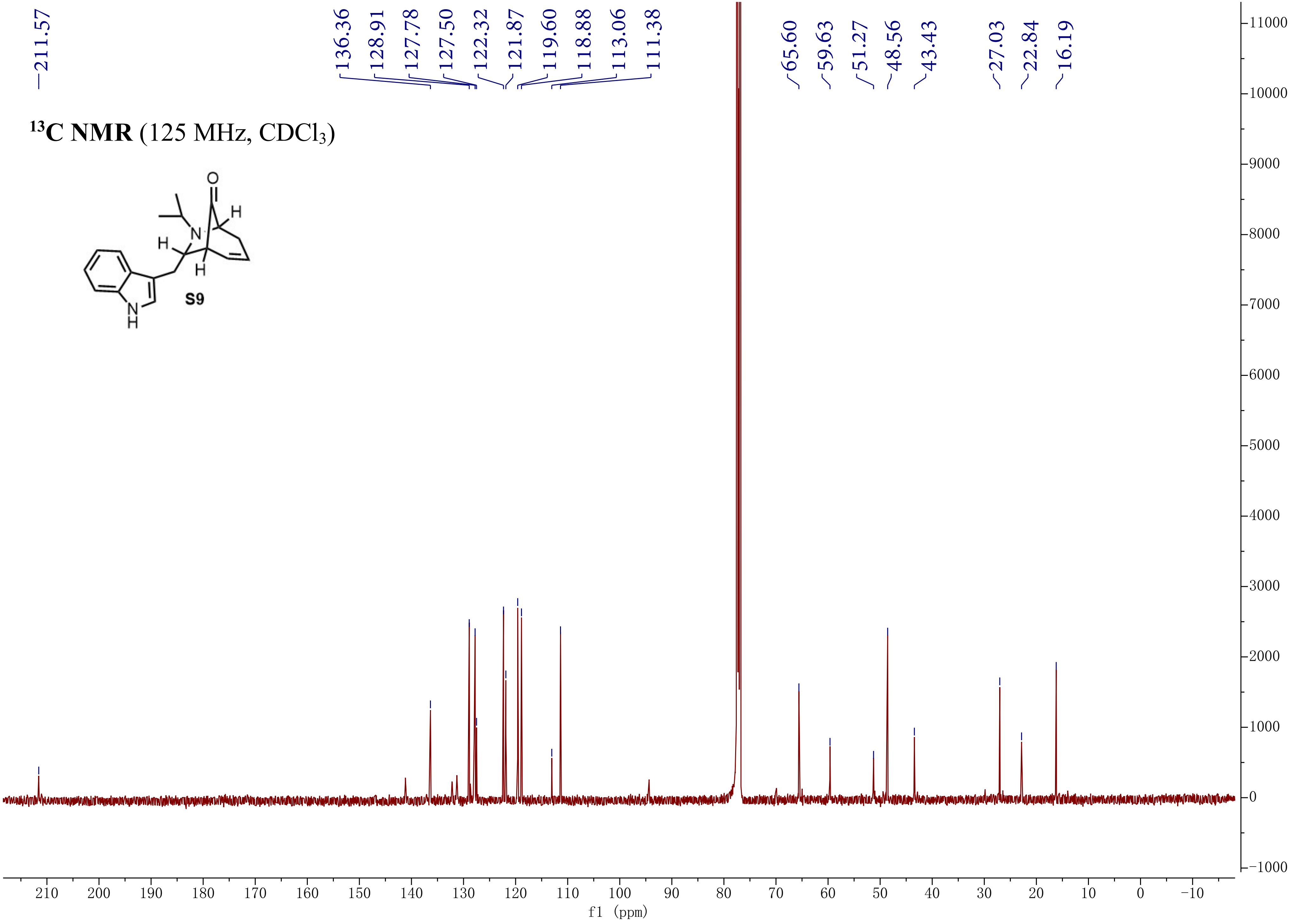

211.57
136.36
128.91
127.78
127.50
122.32
121.87
119.60
118.88
113.06
111.38
65.60
59.63
51.27
48.56
43.43
27.03
22.84
16.19
^{13}C NMR (125 MHz, $CDCl_3$)
O
H
N
H
H
N
H
S9
11000
10000
9000
8000
7000
6000
5000
4000
3000
2000
1000
0
−1000
210 200 190 180 170 160 150 140 130 120 110 100 90 80 70 60 50 40 30 20 10 0 −10
f1 (ppm)

^{1}H NMR (500 MHz, $CDCl_3$)

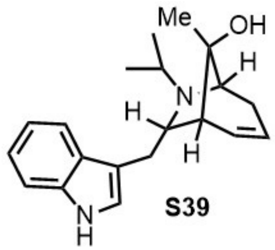

S39

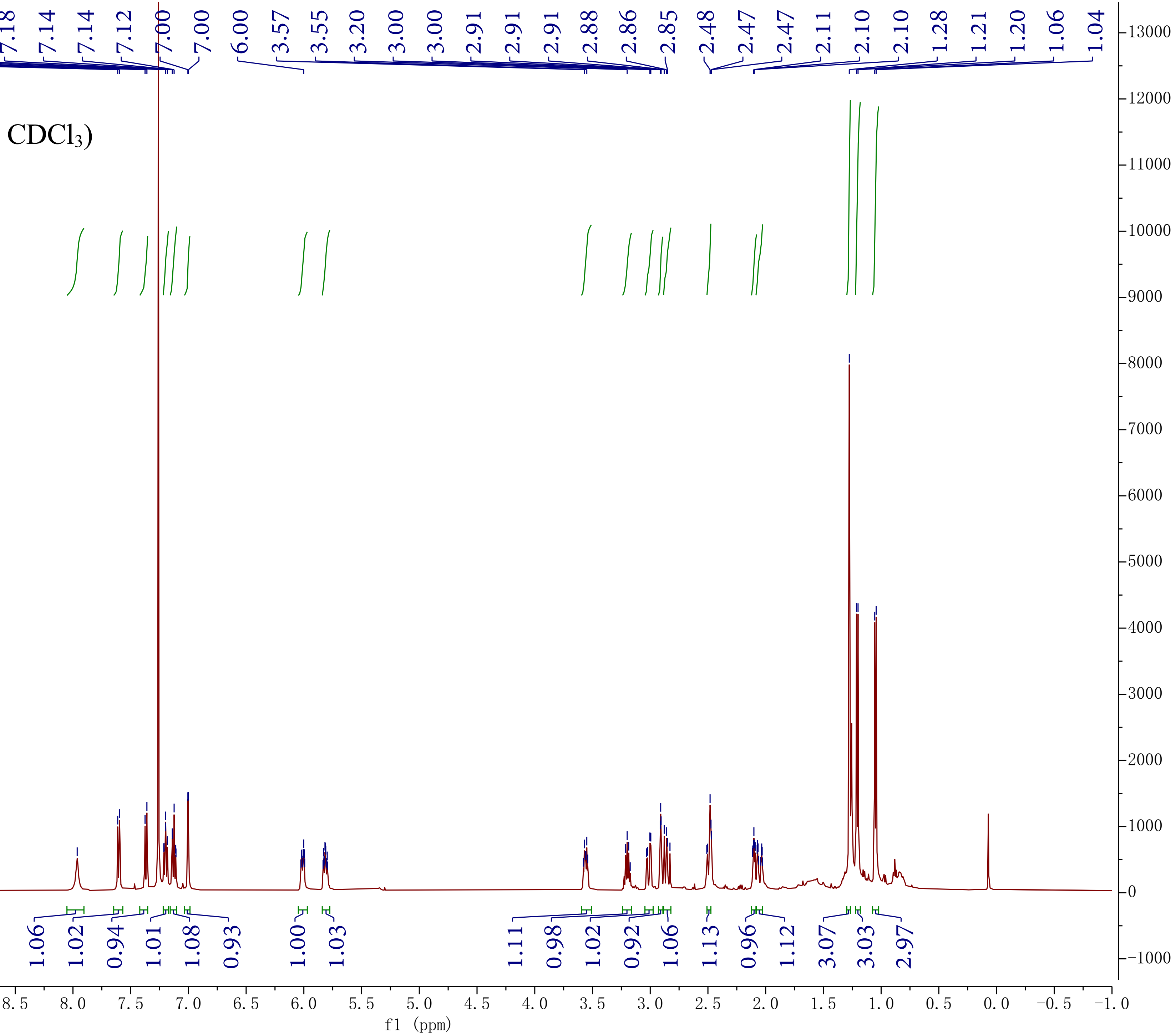

^{13}C NMR (125 MHz, $CDCl_3$)

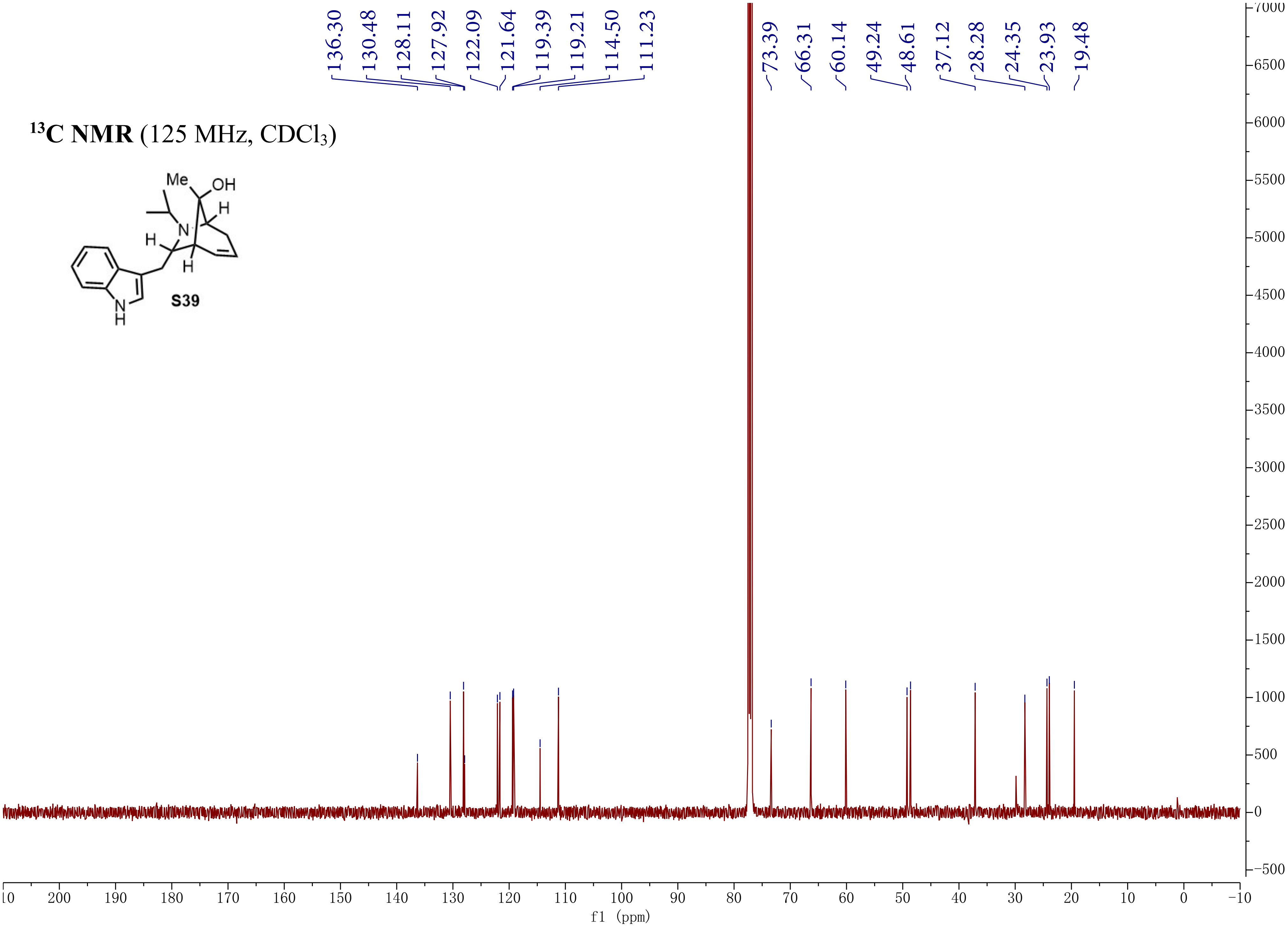

^{1}H NMR (400 MHz, $CDCl_3$)

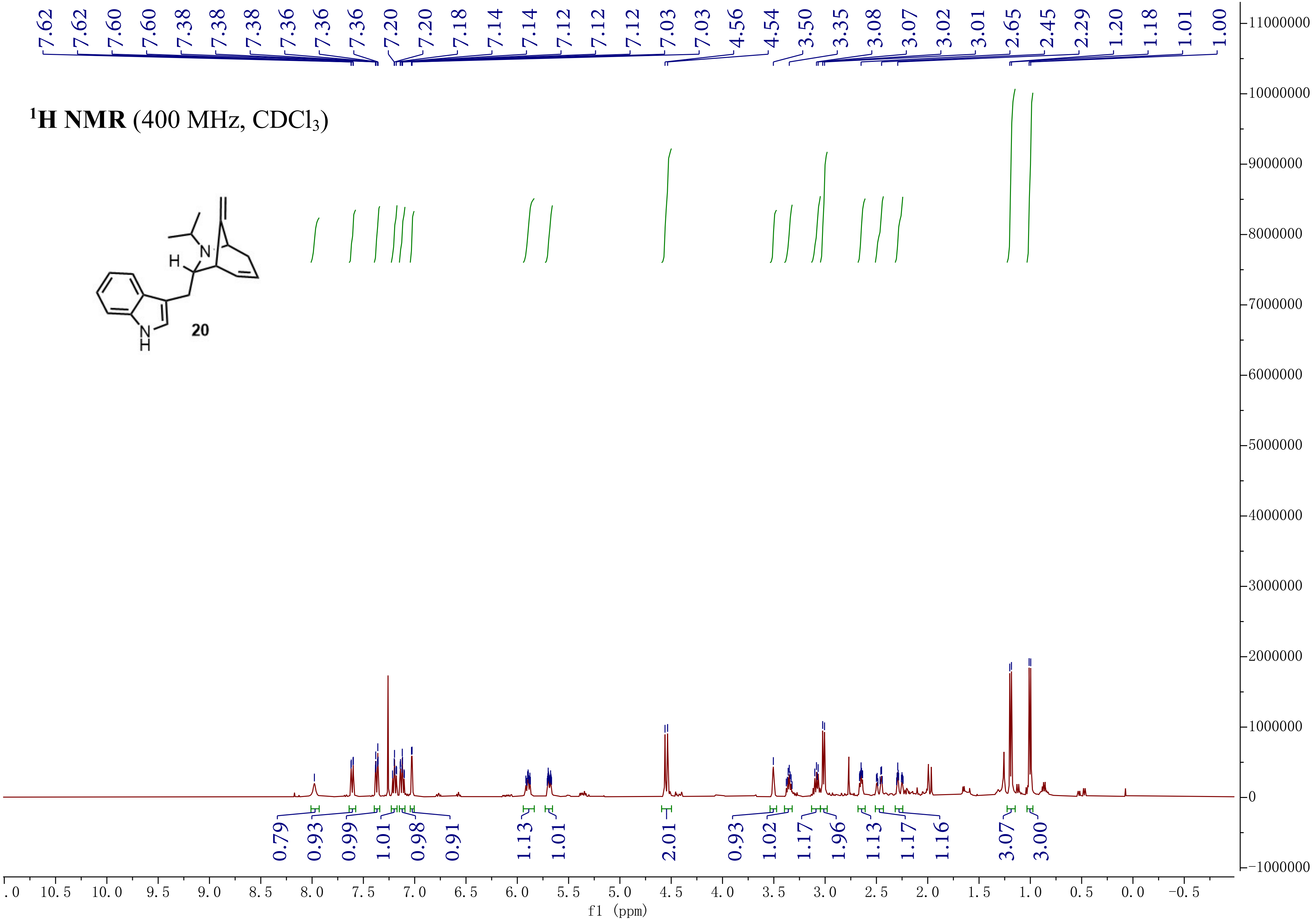

^{13}**C NMR** (100 MHz, $CDCl_3$)

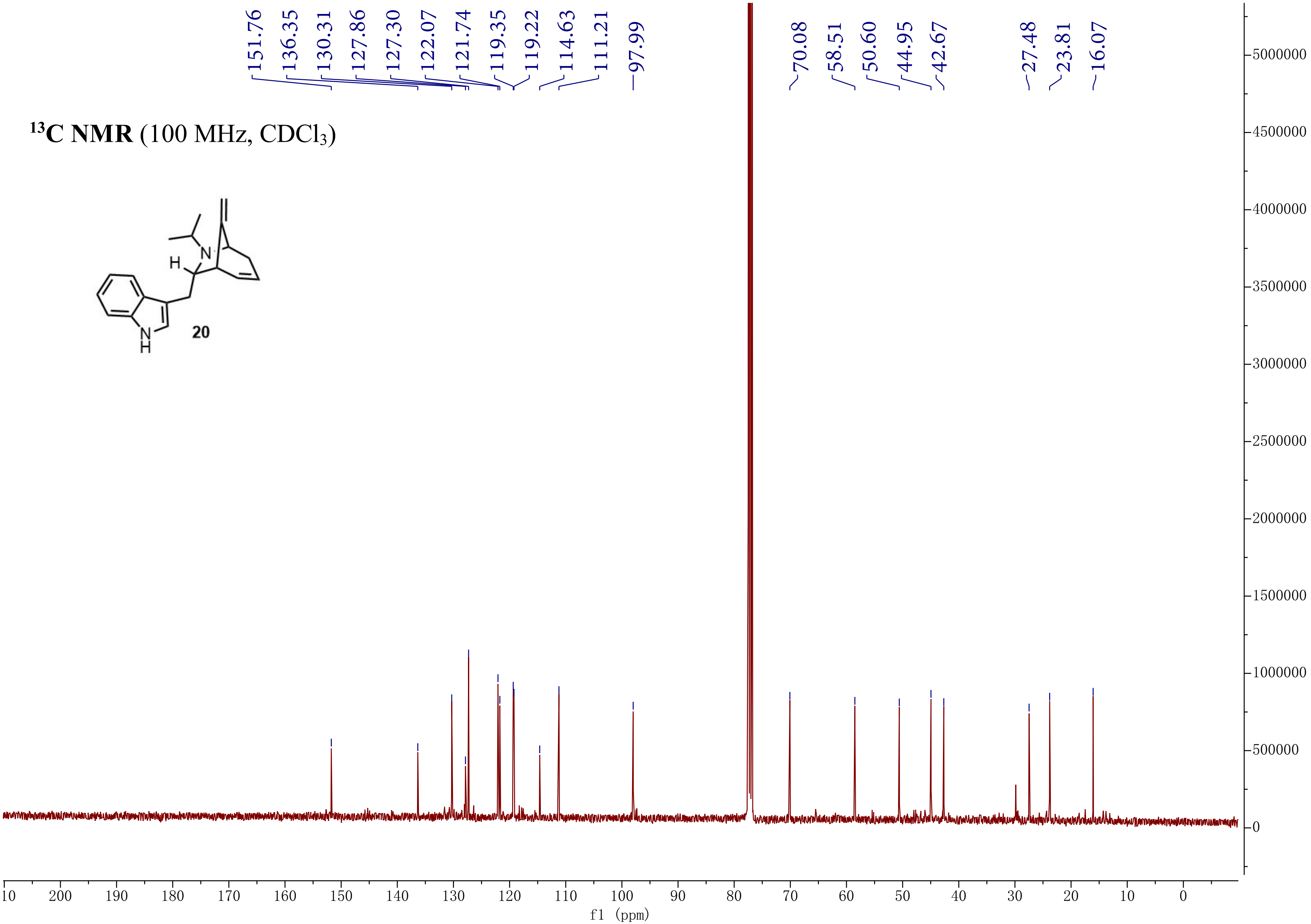

20

^{1}H NMR (500 MHz, $CDCl_3$)

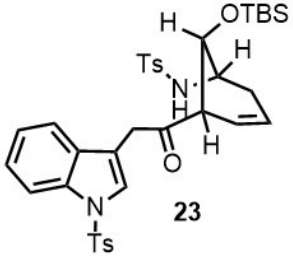

23

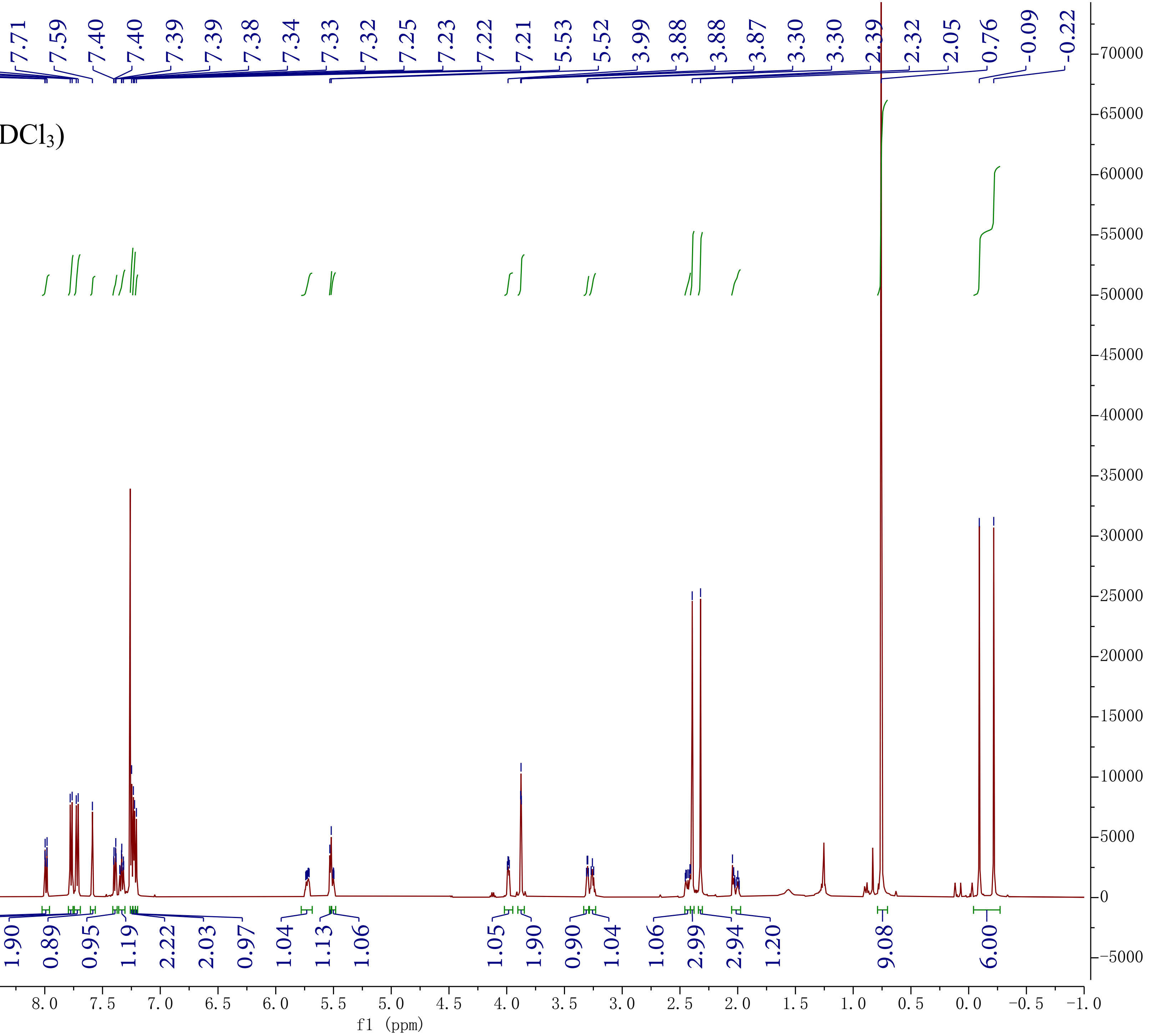

^{13}C NMR (125 MHz, $CDCl_3$)

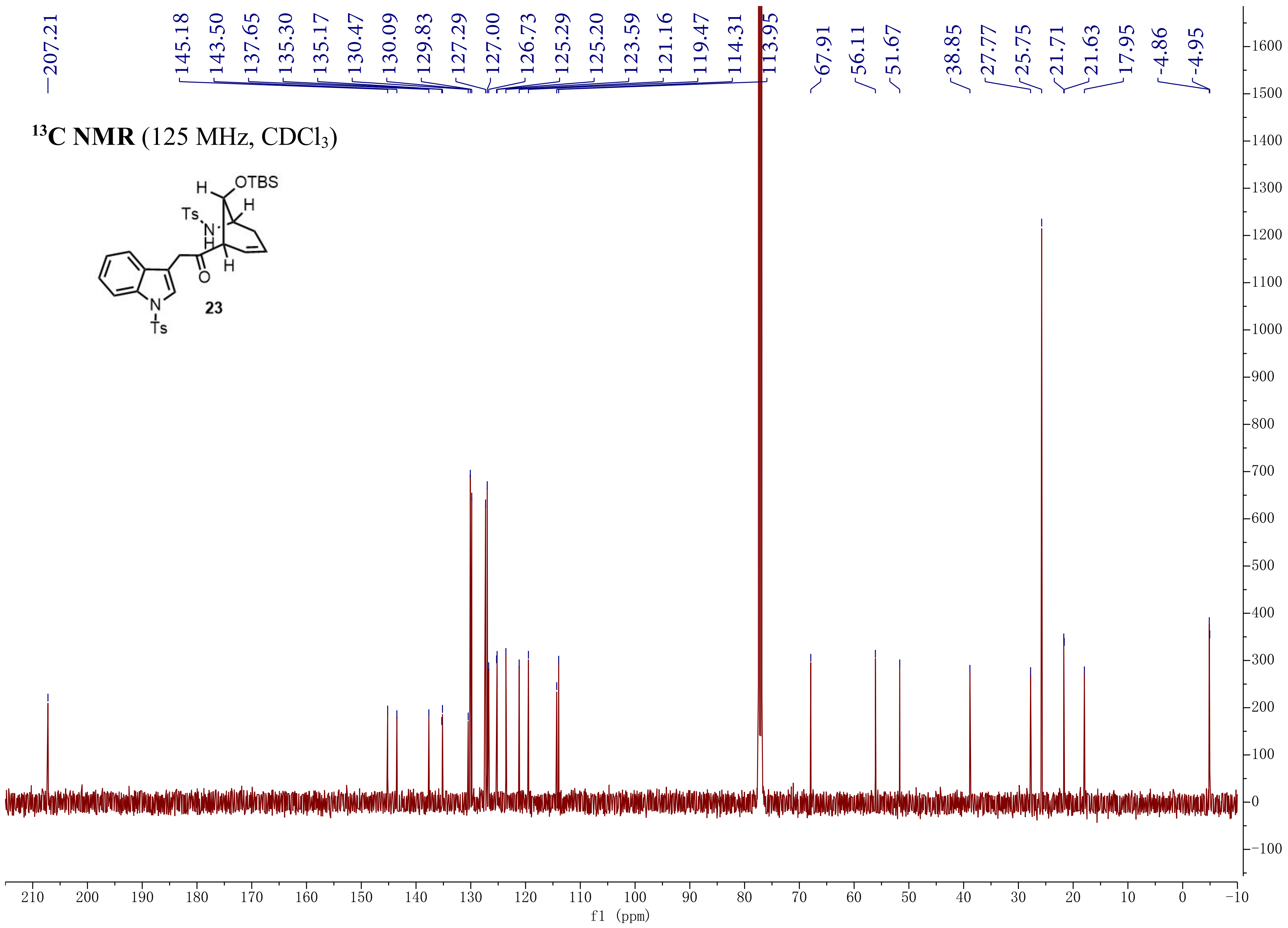

^{1}H NMR (400 MHz, $CDCl_3$)

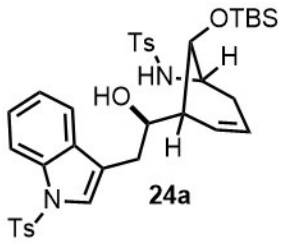

24a

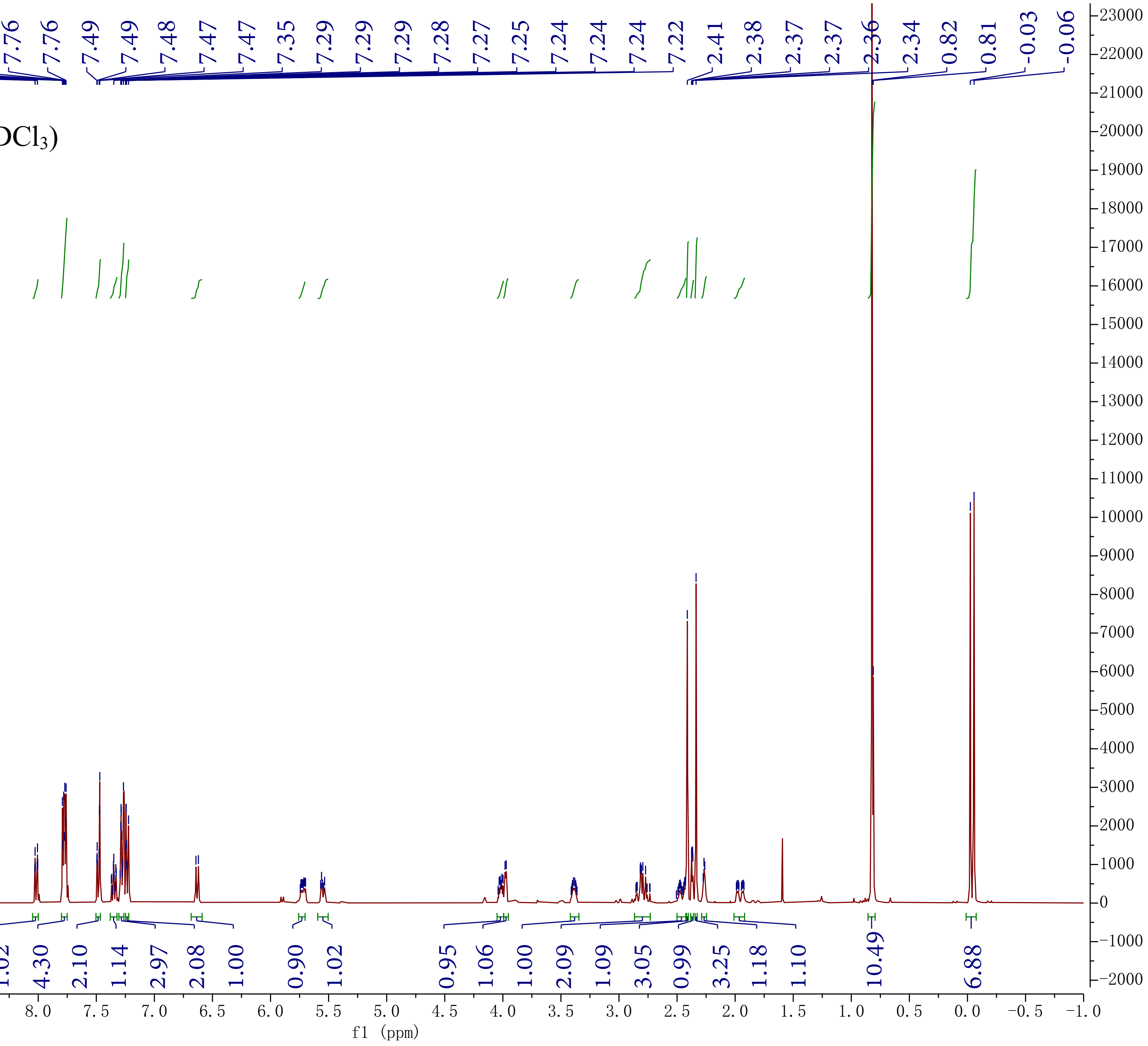

^{13}**C NMR** (100 MHz, $CDCl_3$)

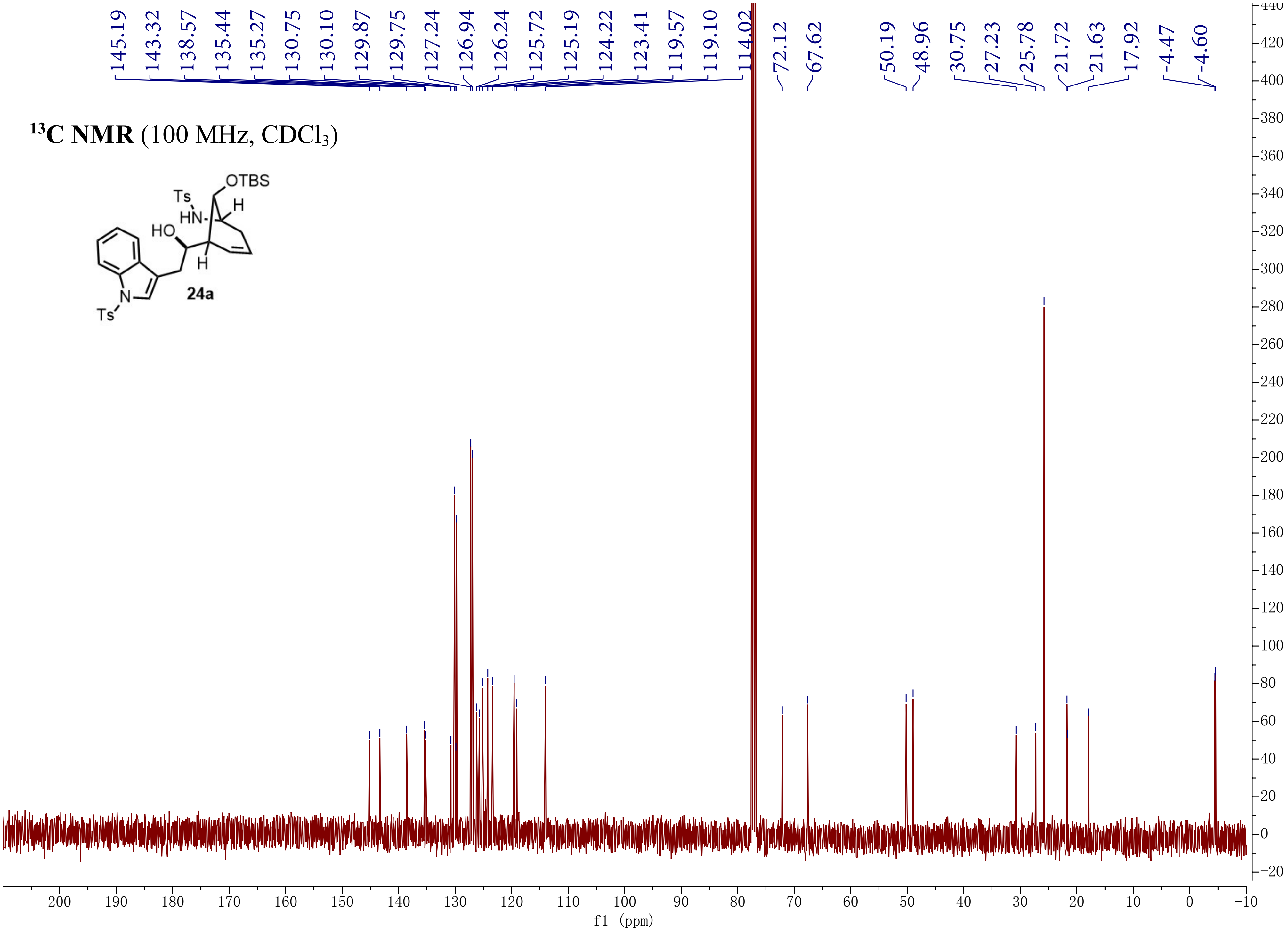

^{1}H NMR (500 MHz, $CDCl_3$)

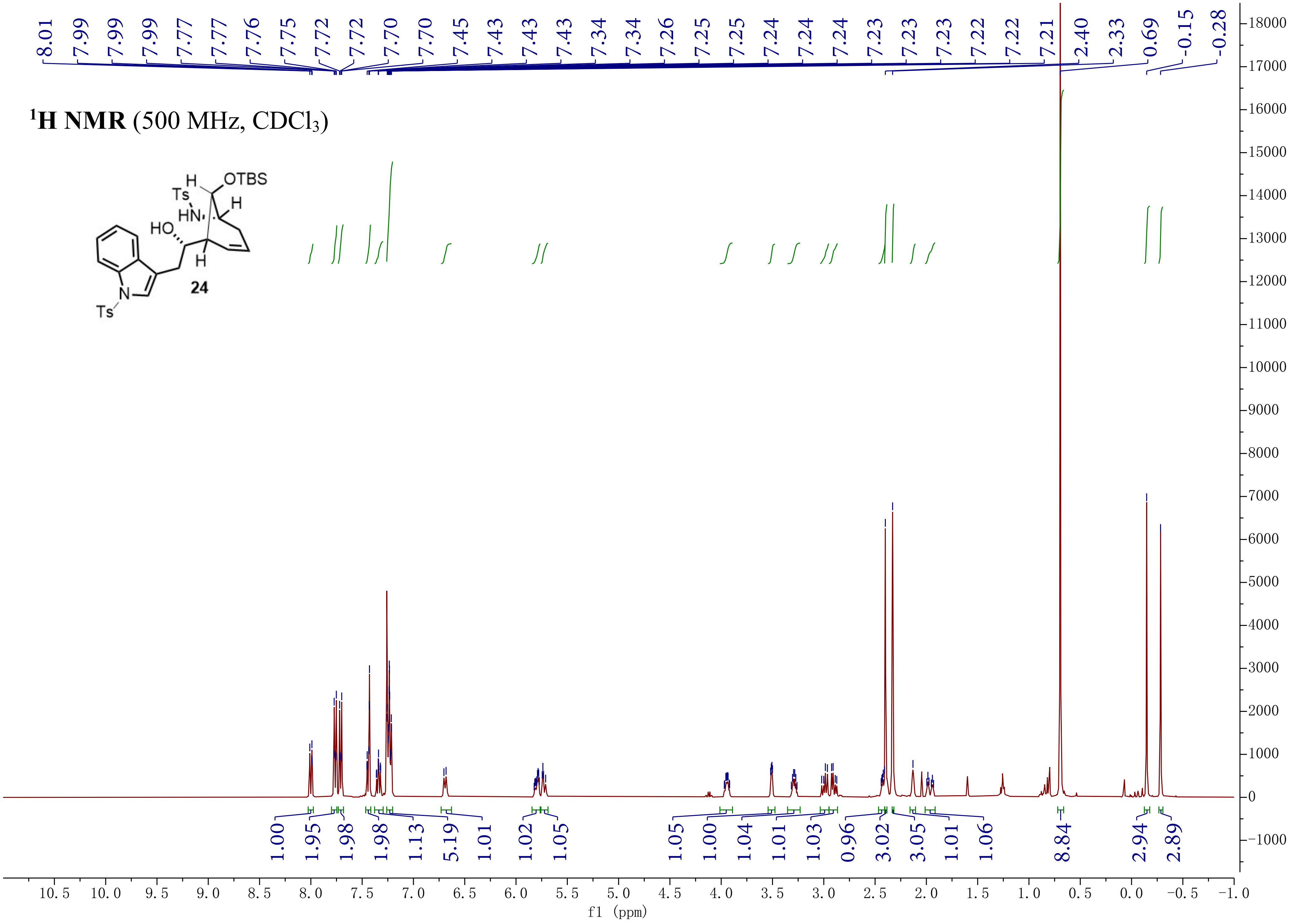

^{13}C NMR (125 MHz, $CDCl_3$)

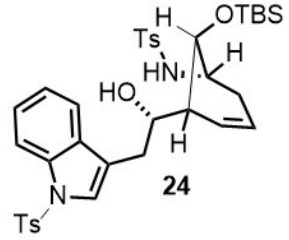

24

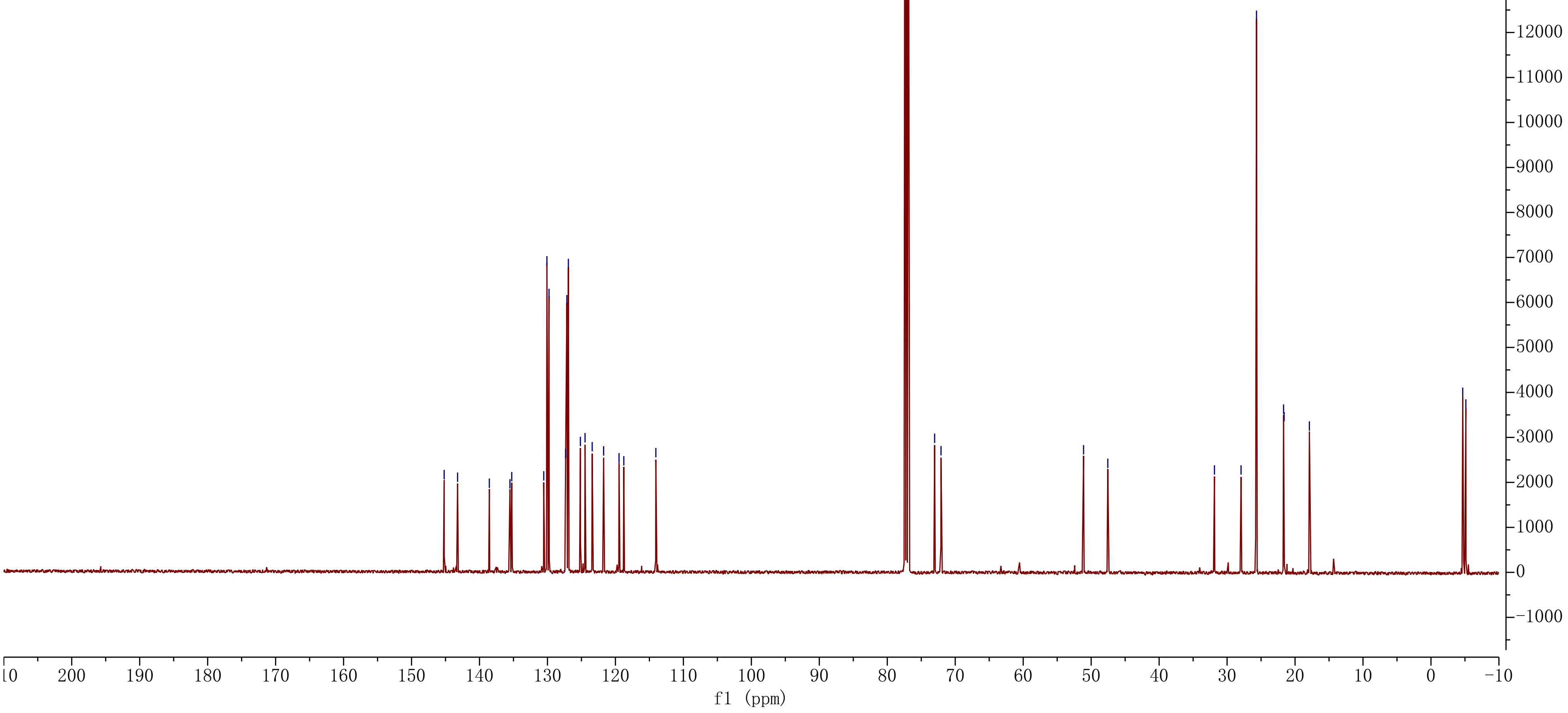

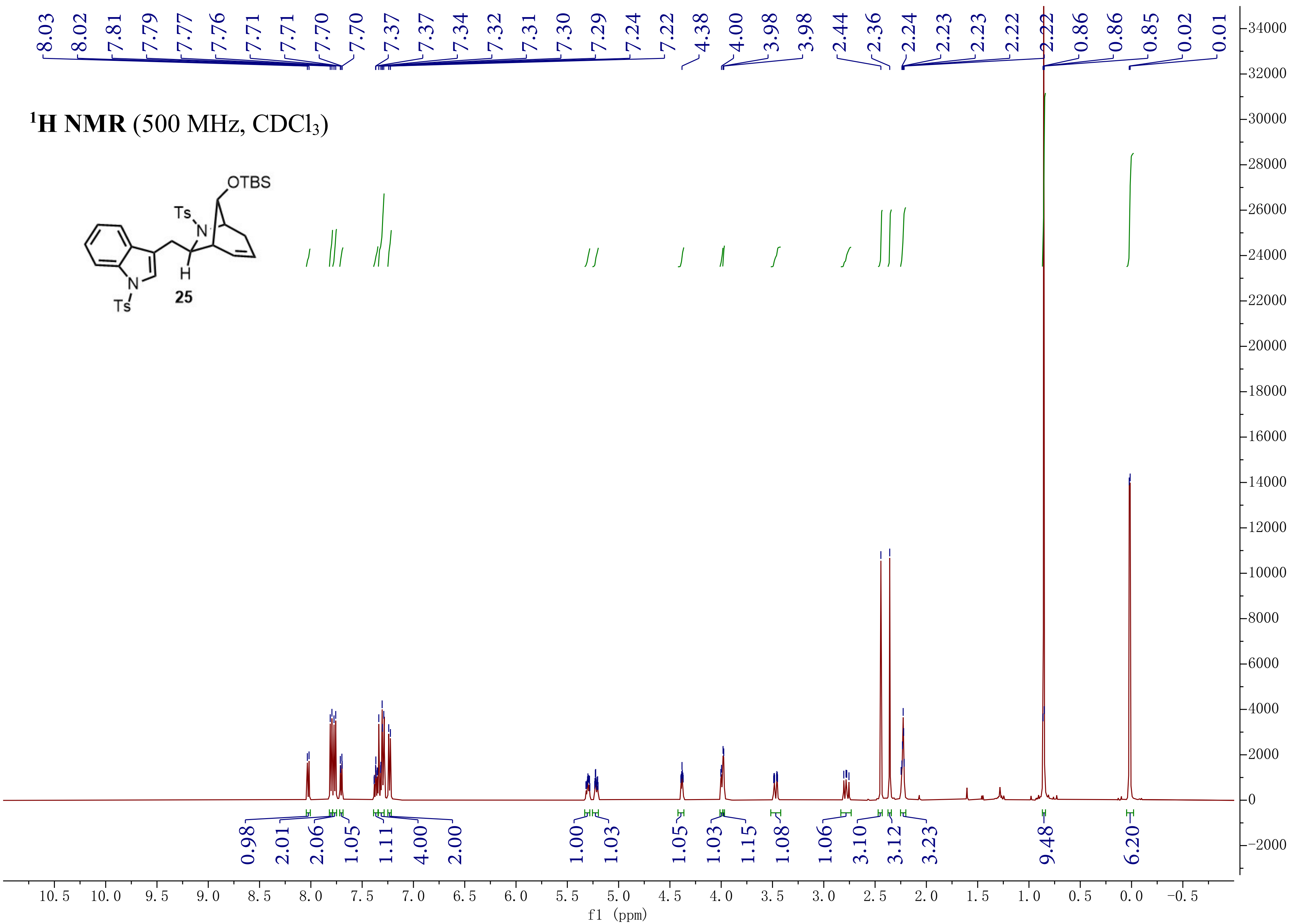

1H NMR (500 MHz, CDCl3)
OTBS
Ts
N
H
N
Ts
25
8.03 8.02 7.81 7.79 7.77 7.76 7.71 7.71 7.70 7.70 7.37 7.37 7.34 7.32 7.31 7.30 7.29 7.24 7.22 4.38 4.00 3.98 3.98 2.44 2.36 2.24 2.23 2.23 2.22 2.22 0.86 0.86 0.85 0.02 0.01
0.98 2.01 2.06 1.05 1.11 4.00 2.00 1.00 1.03 1.05 1.03 1.15 1.08 1.06 3.10 3.12 3.23 9.48 6.20
f1 (ppm)

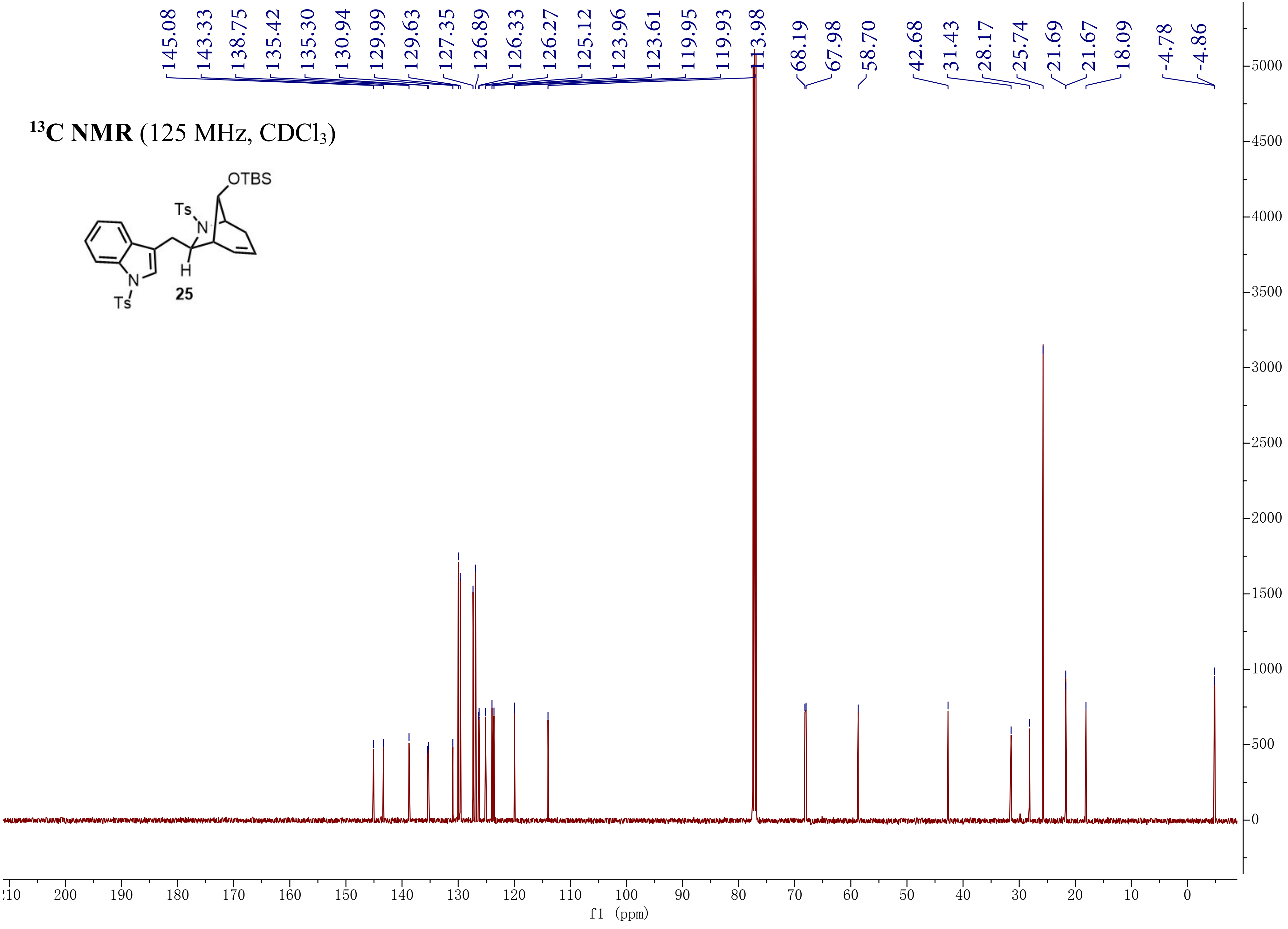

^{13}C NMR (125 MHz, $CDCl_3$)
OTBS
Ts
N
H
N
Ts
25
145.08
143.33
138.75
135.42
135.30
130.94
129.99
129.63
127.35
126.89
126.33
126.27
125.12
123.96
123.61
119.95
119.93
113.98
68.19
67.98
58.70
42.68
31.43
28.17
25.74
21.69
21.67
18.09
-4.78
-4.86
f1 (ppm)

^{1}H NMR (400 MHz, $CDCl_3$)

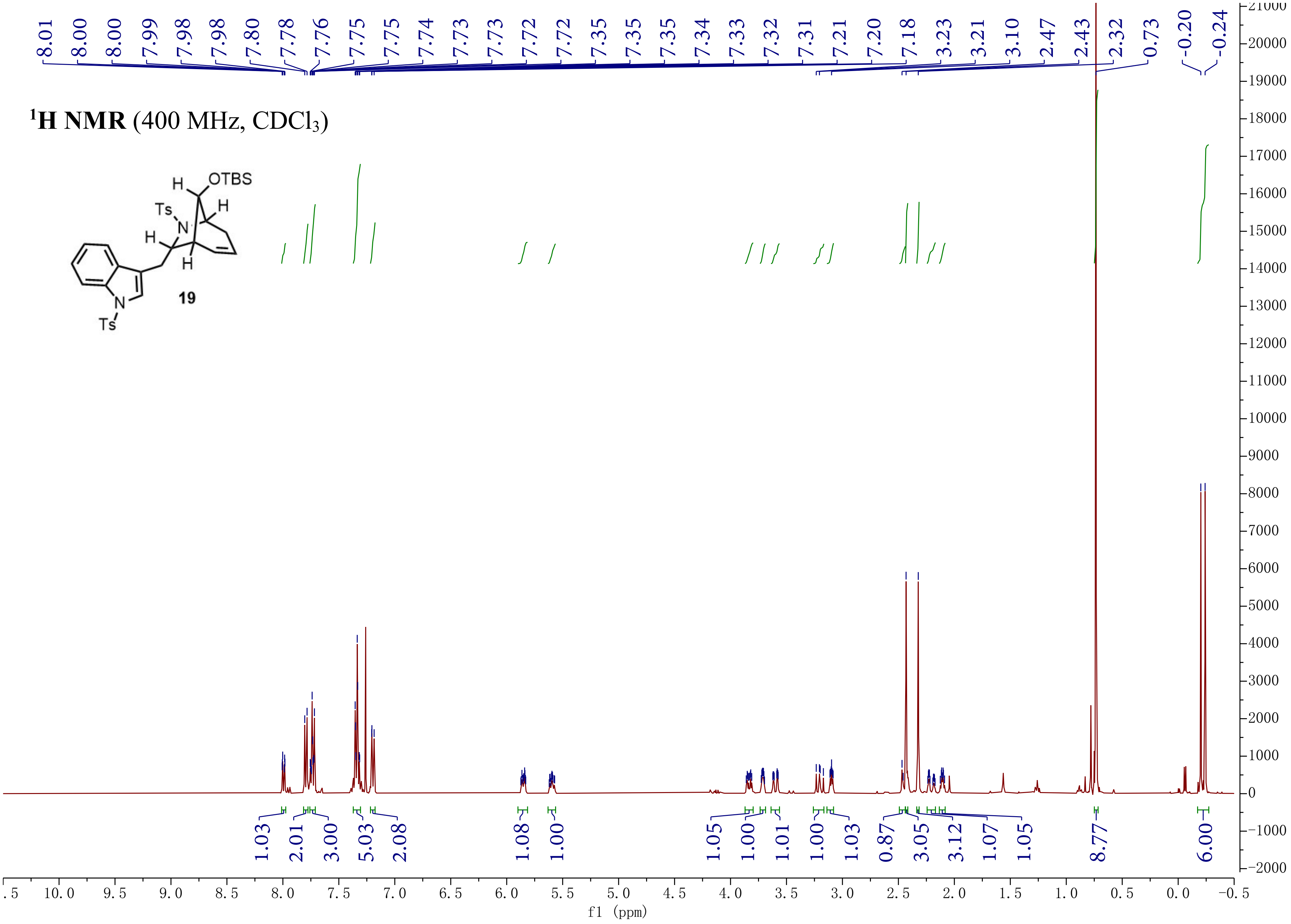

^{13}C NMR (100 MHz, $CDCl_3$)

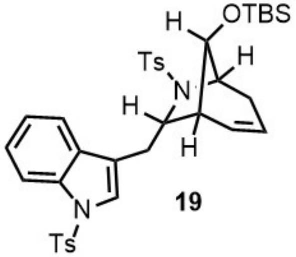

19

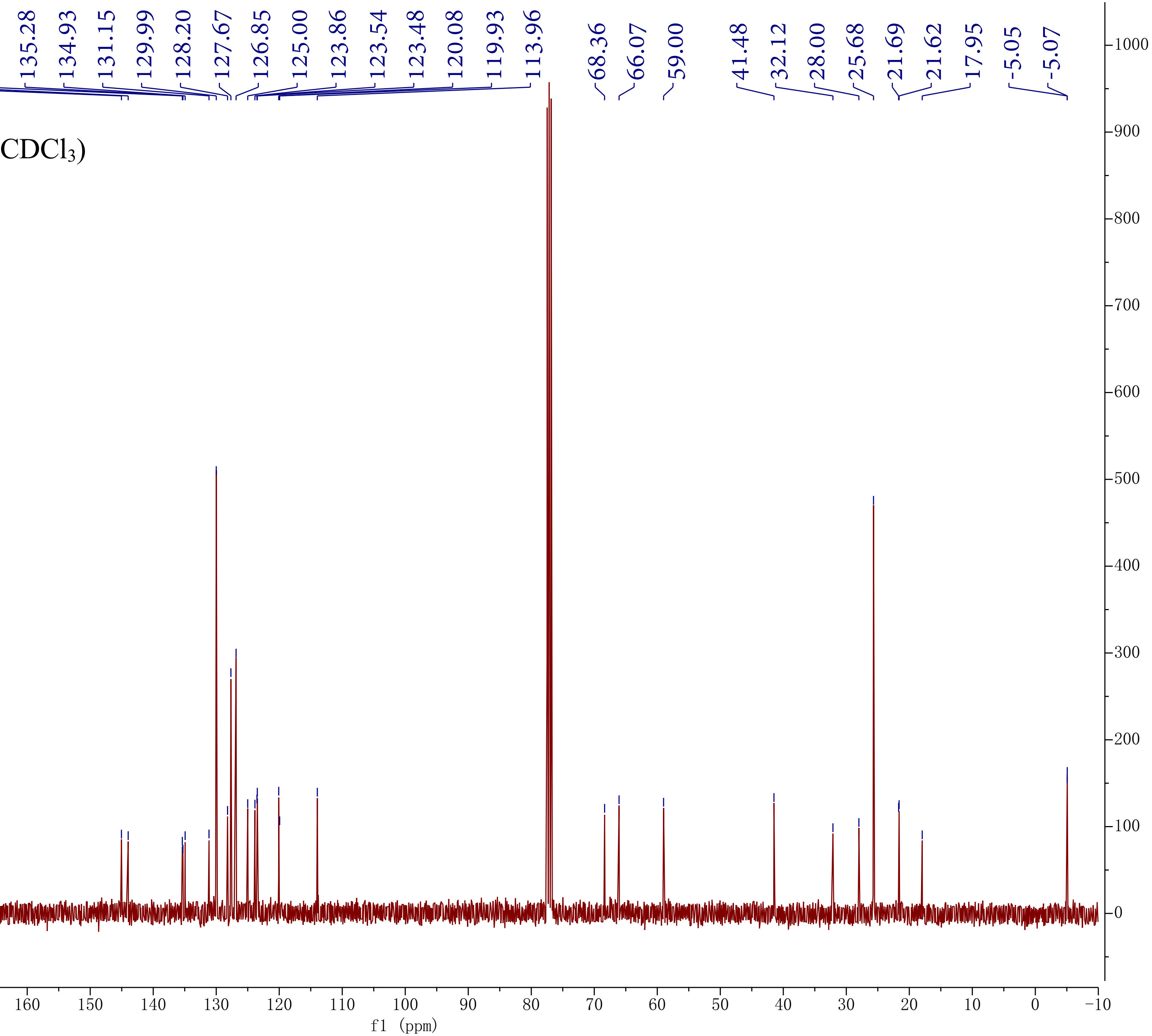

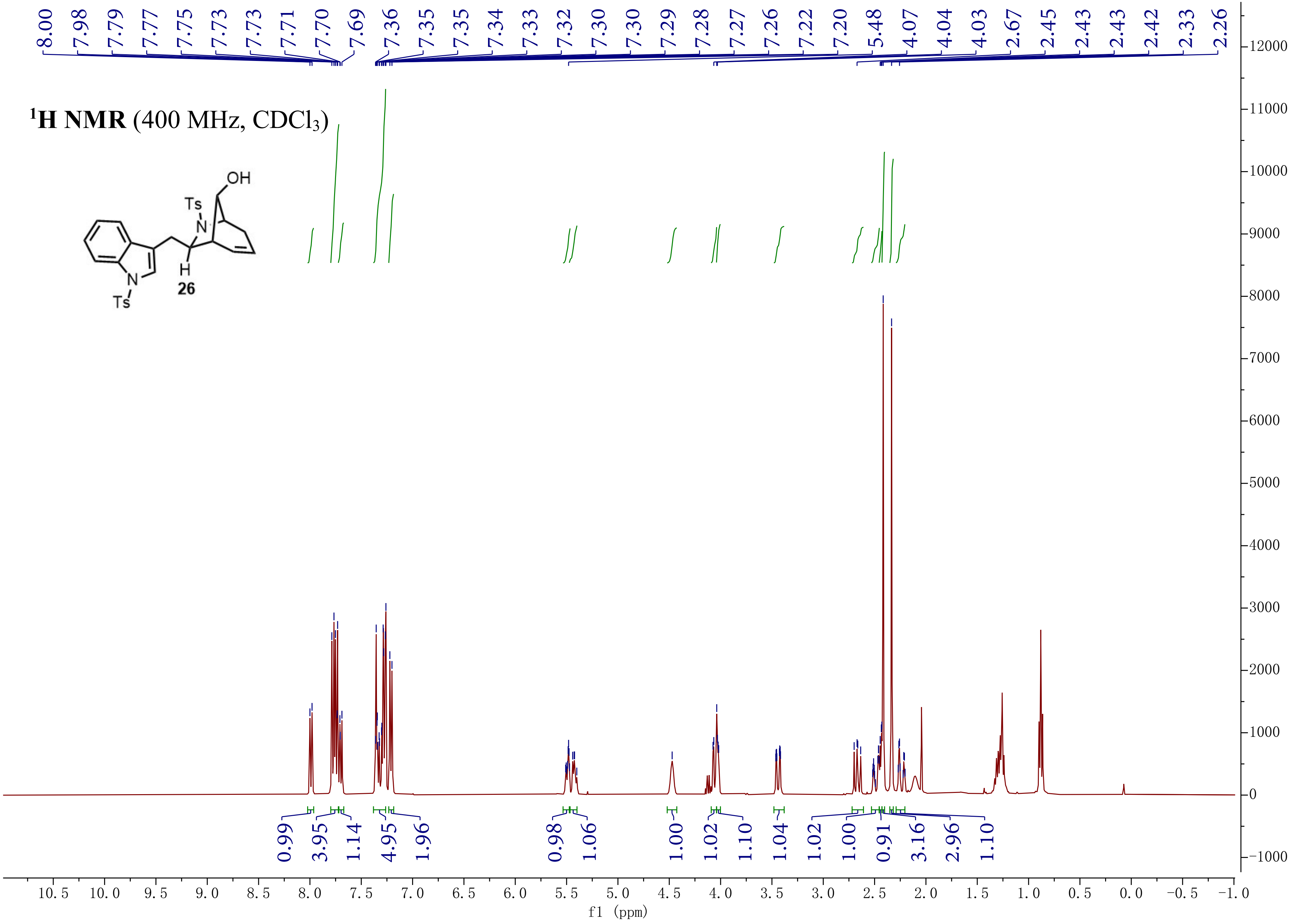

^{1}H NMR (400 MHz, $CDCl_3$)
26
8.00 7.98 7.79 7.77 7.75 7.73 7.73 7.71 7.70 7.69 7.36 7.35 7.35 7.34 7.33 7.32 7.30 7.30 7.29 7.28 7.27 7.26 7.22 7.20 5.48 4.07 4.04 4.03 2.67 2.45 2.43 2.43 2.42 2.33 2.26
0.99 3.95 1.14 4.95 1.96 0.98 1.06 1.00 1.02 1.10 1.04 1.02 1.00 0.91 3.16 2.96 1.10
f1 (ppm)

^{13}**C NMR** (100 MHz, $CDCl_3$)

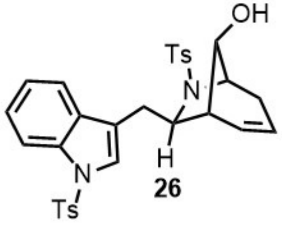

26

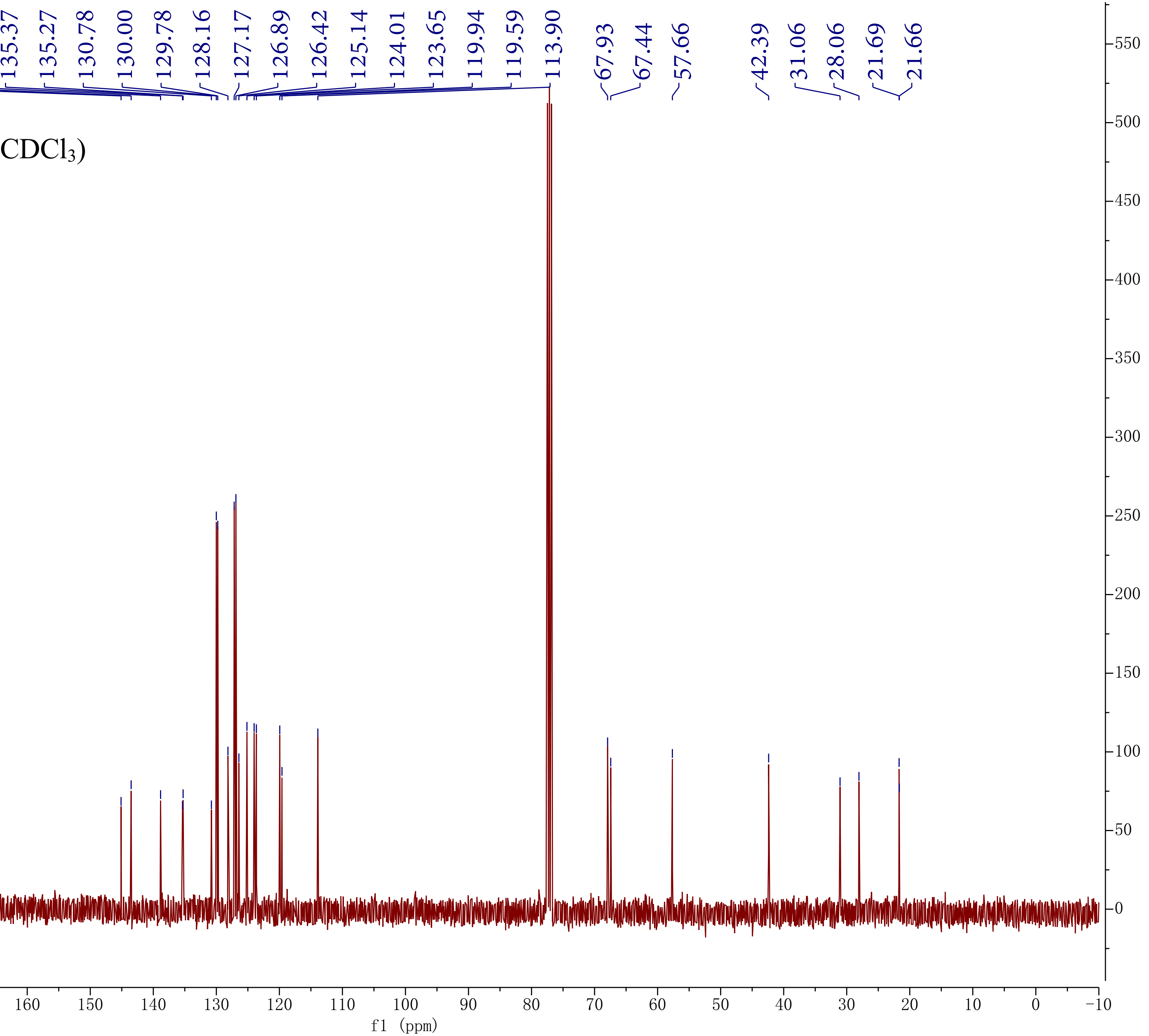

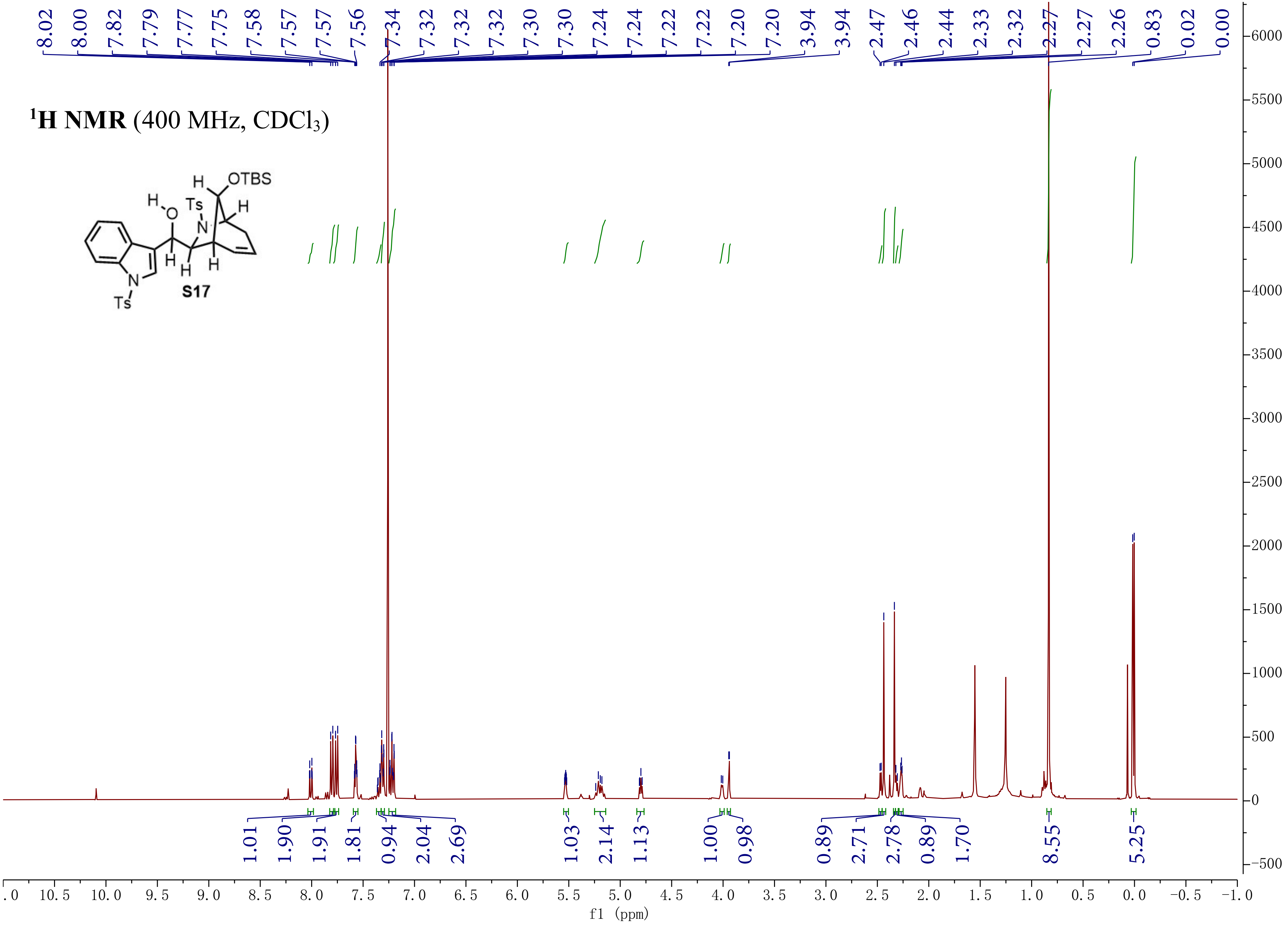

8.02 8.00 7.82 7.79 7.77 7.75 7.58 7.57 7.57 7.56 7.34 7.32 7.32 7.32 7.30 7.30 7.24 7.24 7.22 7.22 7.20 7.20 3.94 3.94 2.47 2.46 2.44 2.33 2.32 2.27 2.27 2.26 0.83 0.02 0.00
1H NMR (400 MHz, CDCl3)
OTBS
Ts
S17
1.01 1.90 1.91 1.81 0.94 2.04 2.69 1.03 2.14 1.13 1.00 0.98 0.89 2.71 2.78 0.89 1.70 8.55 5.25
f1 (ppm)

^{13}C NMR (100 MHz, $CDCl_3$)

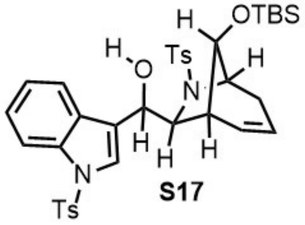

S17

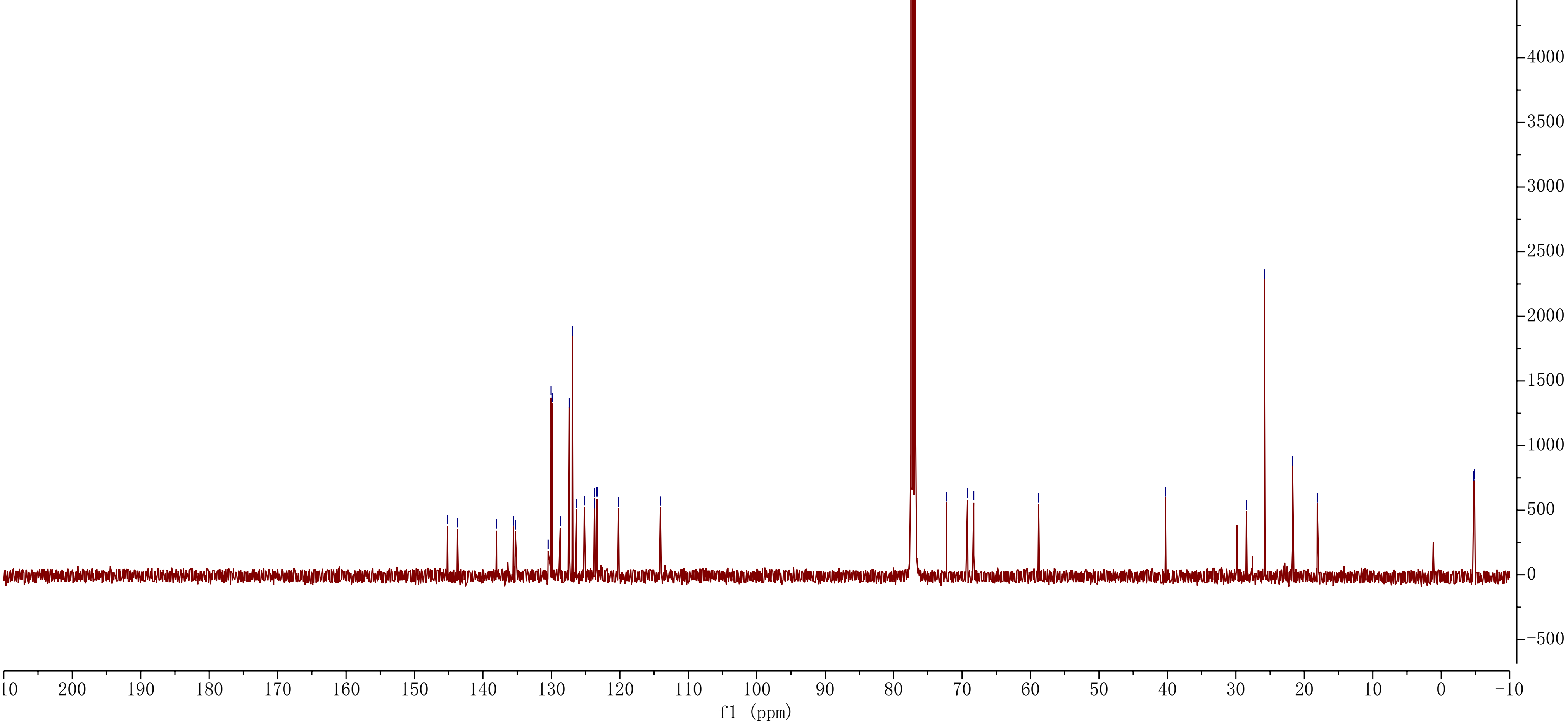

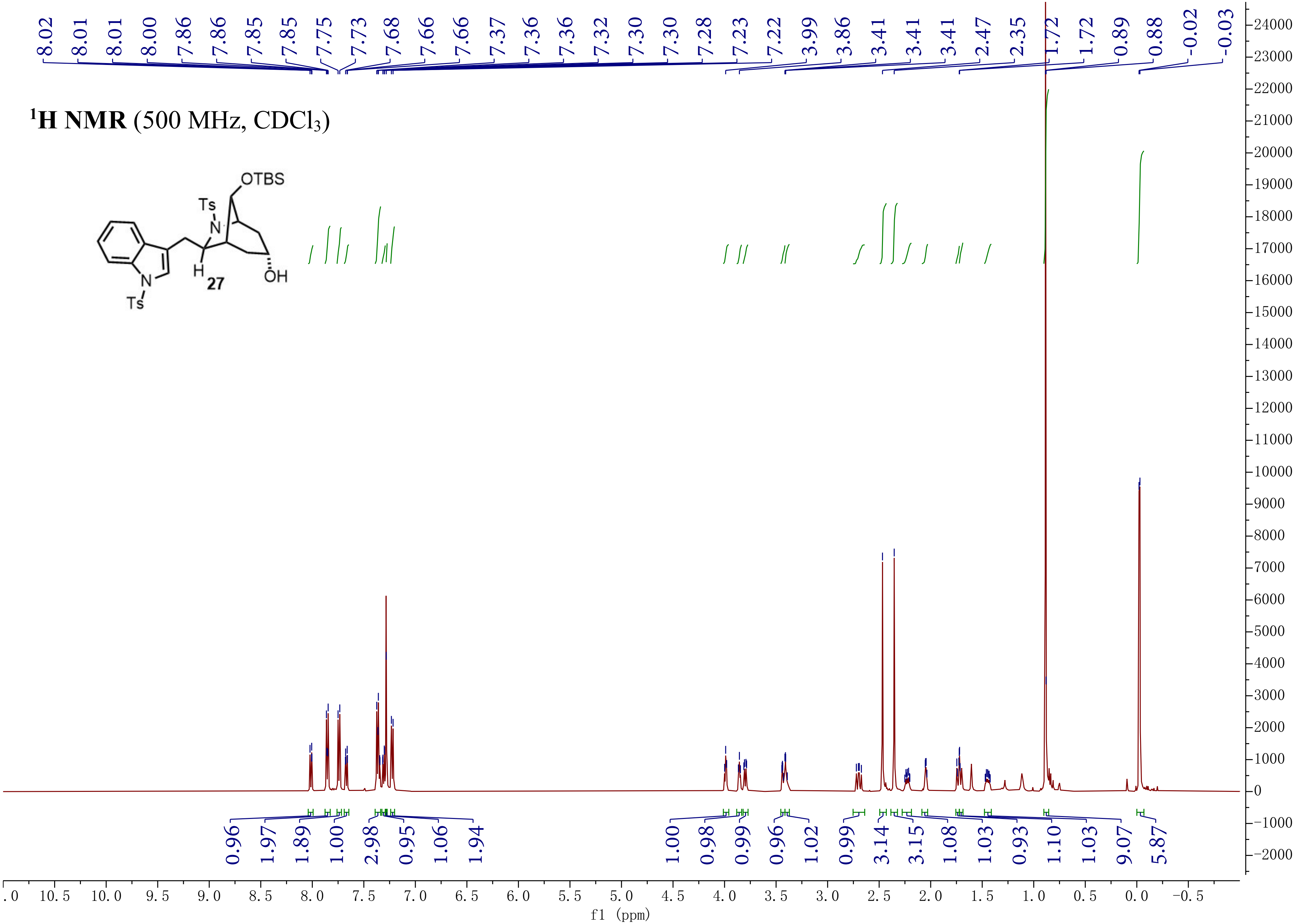

1H NMR (500 MHz, CDCl3)
OTBS
Ts
N
H
27
OH
N
Ts
8.02 8.01 8.01 8.00 7.86 7.86 7.85 7.85 7.75 7.73 7.68 7.66 7.66 7.37 7.36 7.36 7.32 7.30 7.30 7.28 7.23 7.22 3.99 3.86 3.41 3.41 3.41 2.47 2.35 1.72 1.72 0.89 0.88 -0.02 -0.03
0.96 1.97 1.89 1.00 2.98 0.95 1.06 1.94 1.00 0.98 0.99 0.96 1.02 0.99 3.14 3.15 1.08 1.03 0.93 1.10 1.03 9.07 5.87
f1 (ppm)

^{13}C NMR (125 MHz, $CDCl_3$)

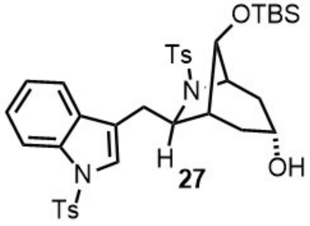

27

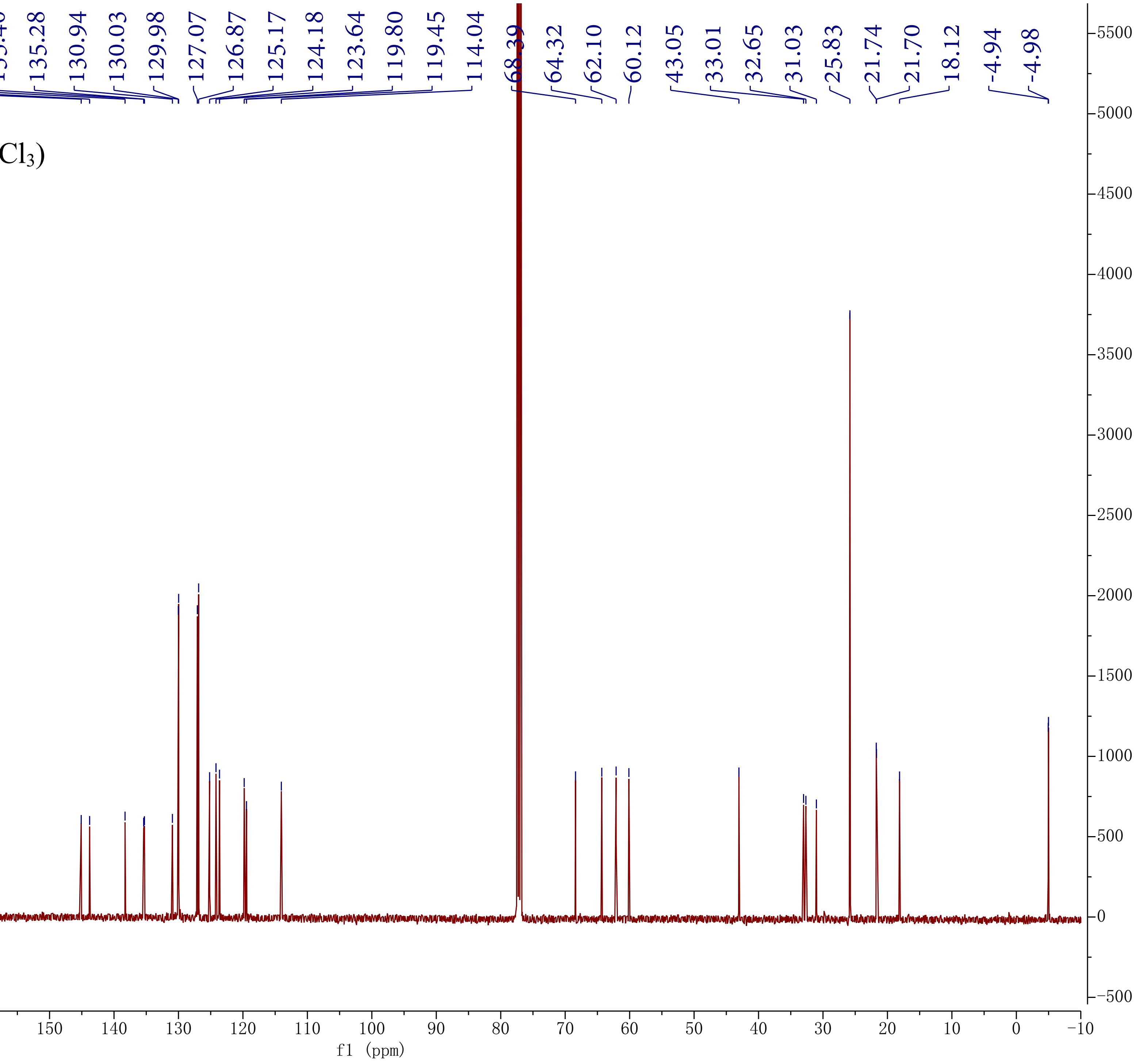

^{1}H NMR (400 MHz, $CDCl_3$)

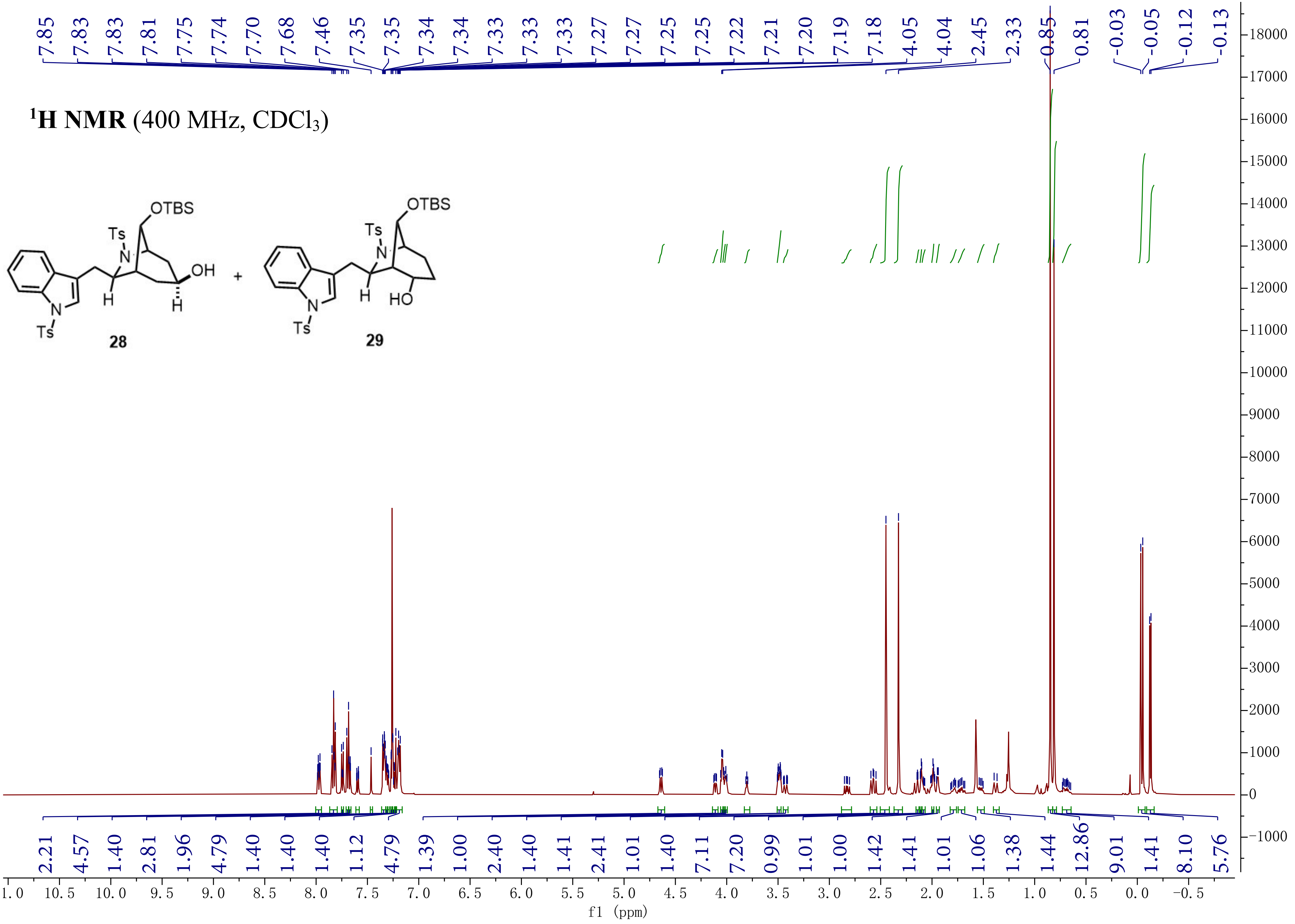

28 29

^{13}C NMR (100 MHz, $CDCl_3$)

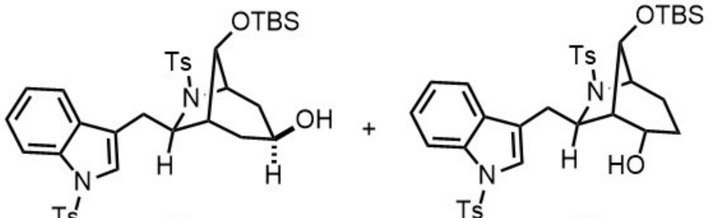

28 **29**

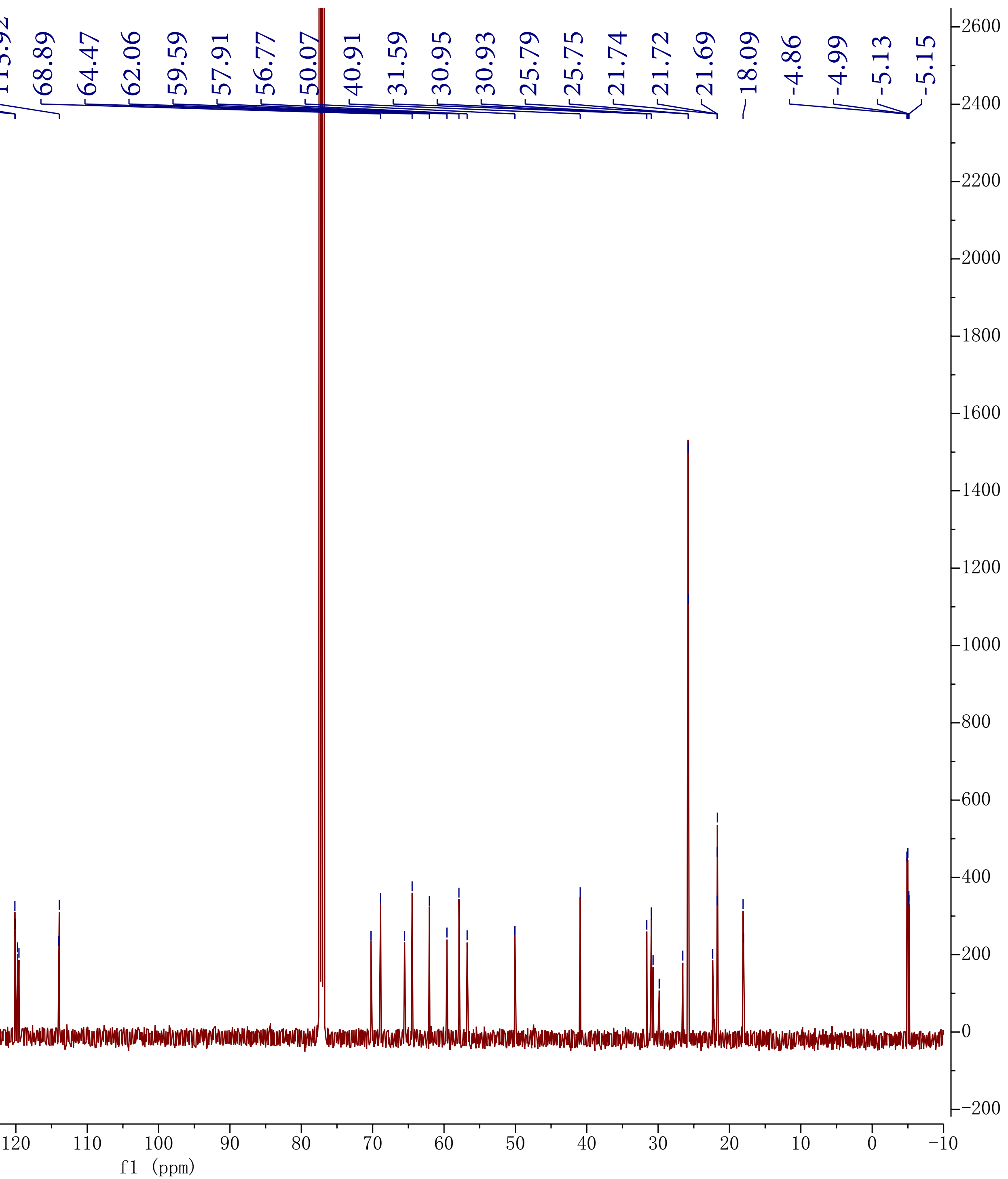

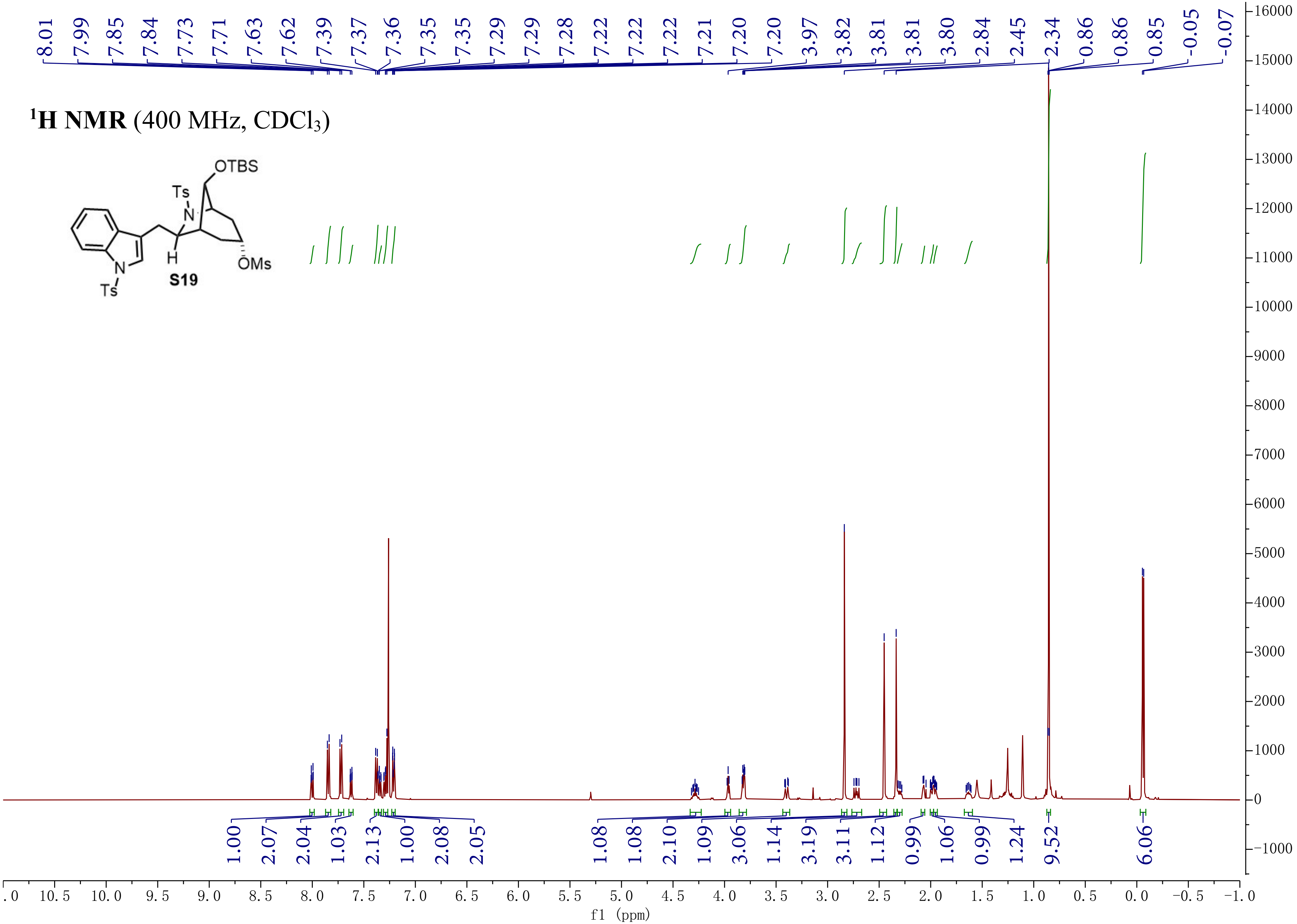

1H NMR (400 MHz, CDCl3)
OTBS
Ts
N
H
OMs
N
Ts
S19
8.01 7.99 7.85 7.84 7.73 7.71 7.63 7.62 7.39 7.37 7.36 7.35 7.35 7.29 7.29 7.28 7.22 7.22 7.22 7.21 7.20 7.20 3.97 3.82 3.81 3.81 3.80 2.84 2.45 2.34 0.86 0.86 0.85 -0.05 -0.07
1.00 2.07 2.04 1.03 2.13 1.00 2.08 2.05 1.08 1.08 2.10 1.09 3.06 1.14 3.19 3.11 1.12 0.99 1.06 0.99 1.24 9.52 6.06
f1 (ppm)

^{13}C NMR (100 MHz, $CDCl_3$)

S19

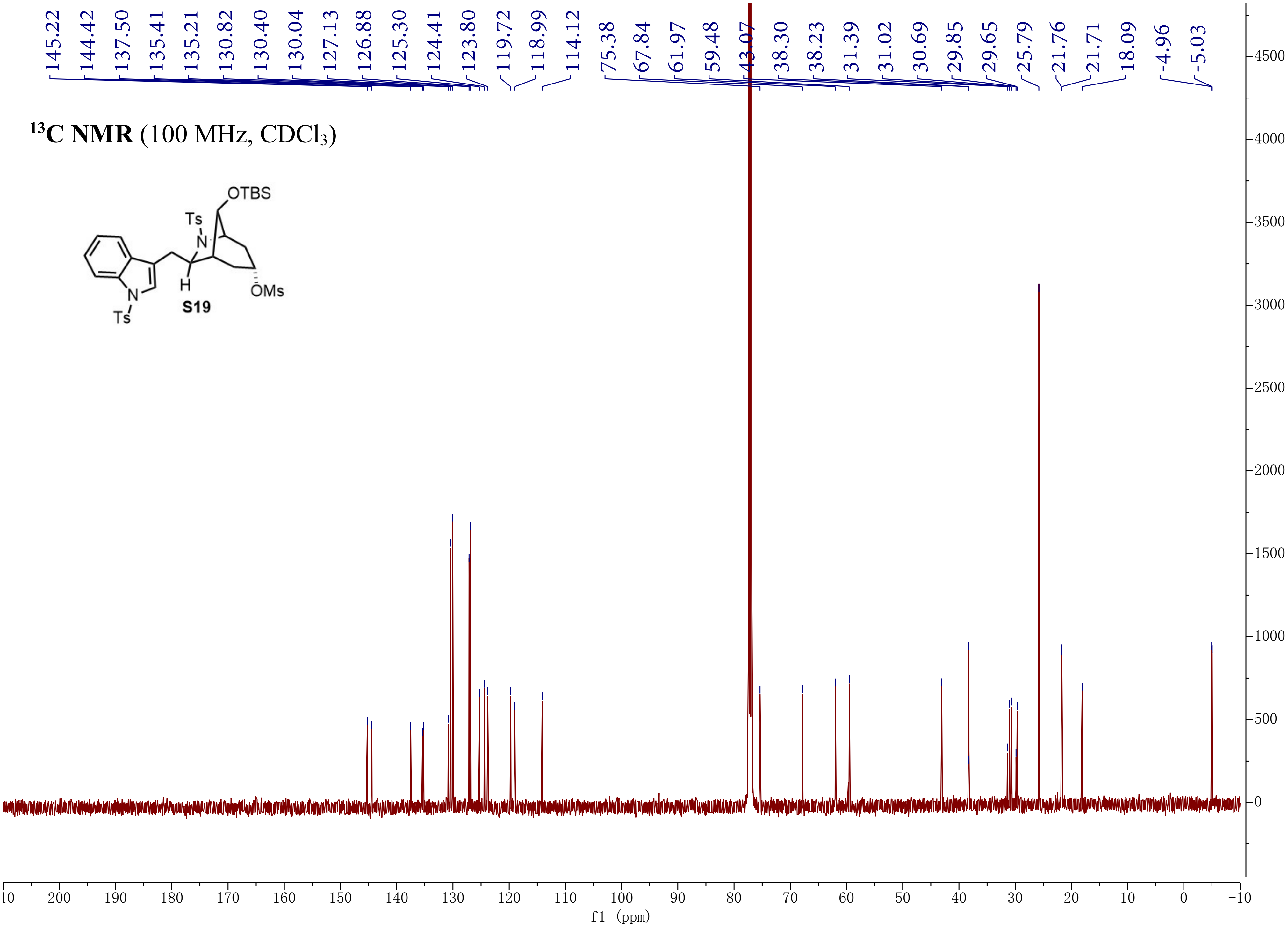

^{1}H NMR (400 MHz, $CDCl_3$)

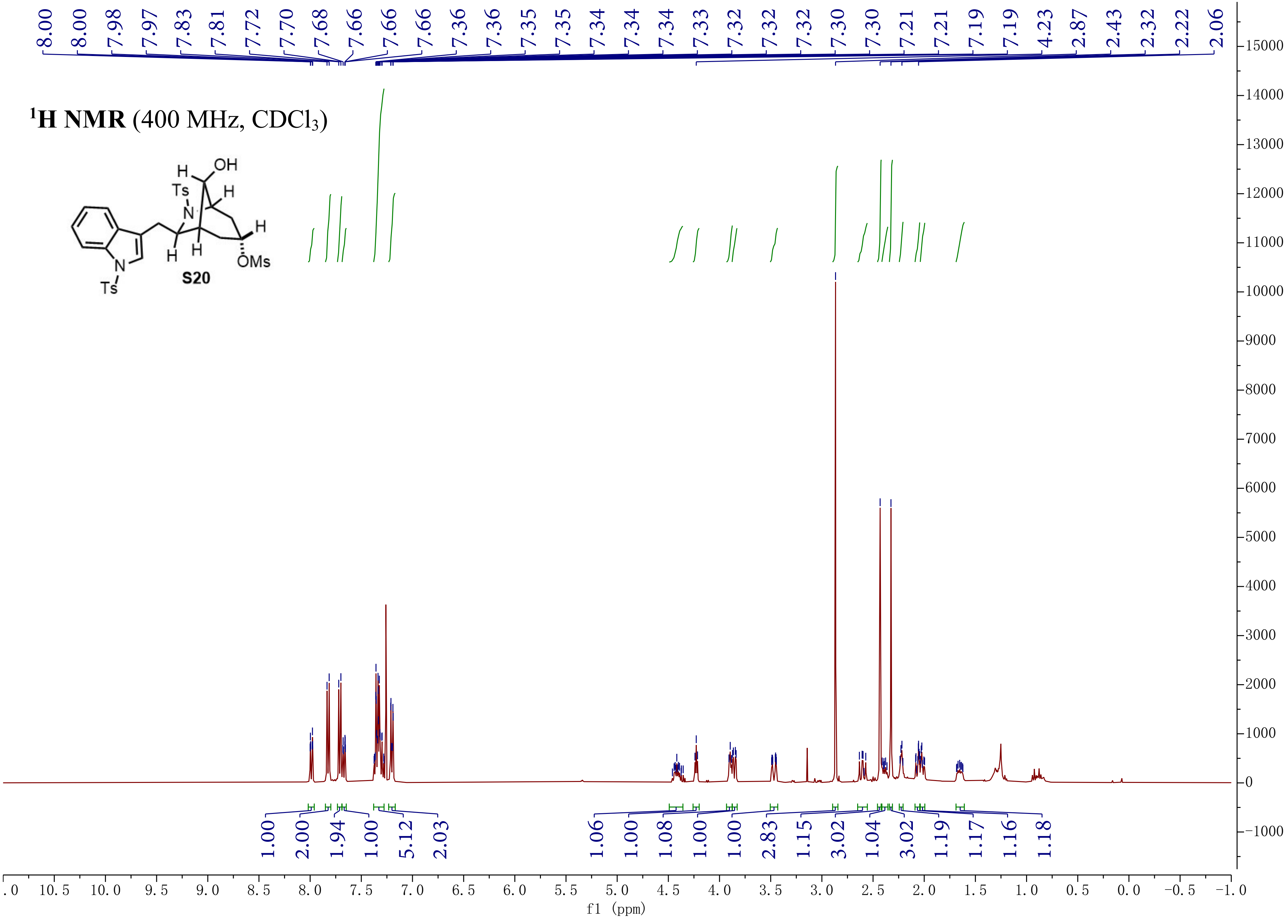

^{13}C NMR (100 MHz, $CDCl_3$)

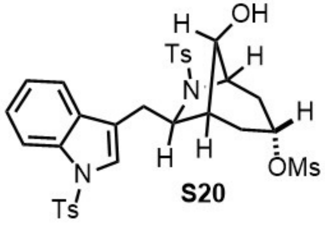

S20

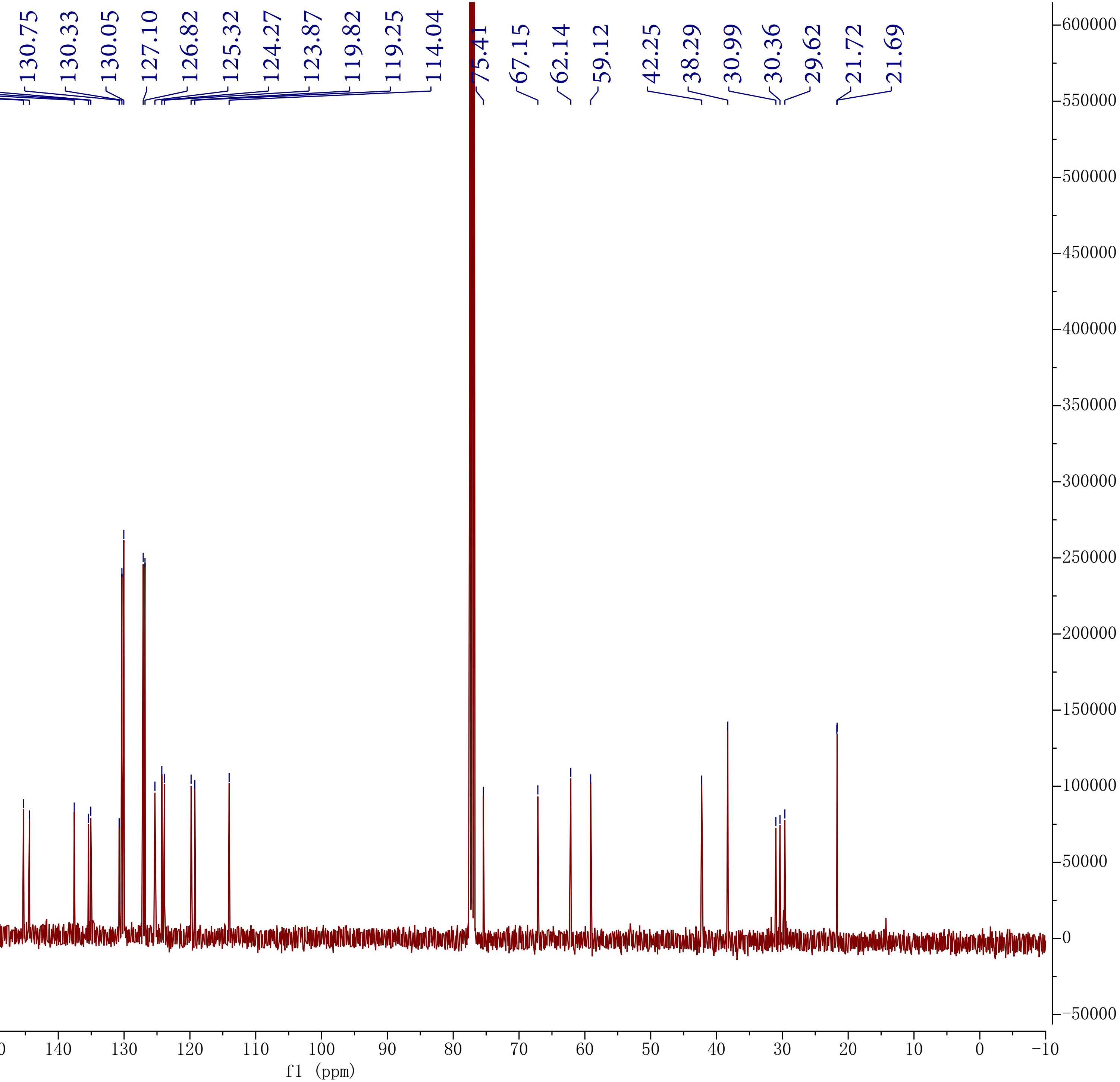

^{1}H NMR (400 MHz, $CDCl_3$)

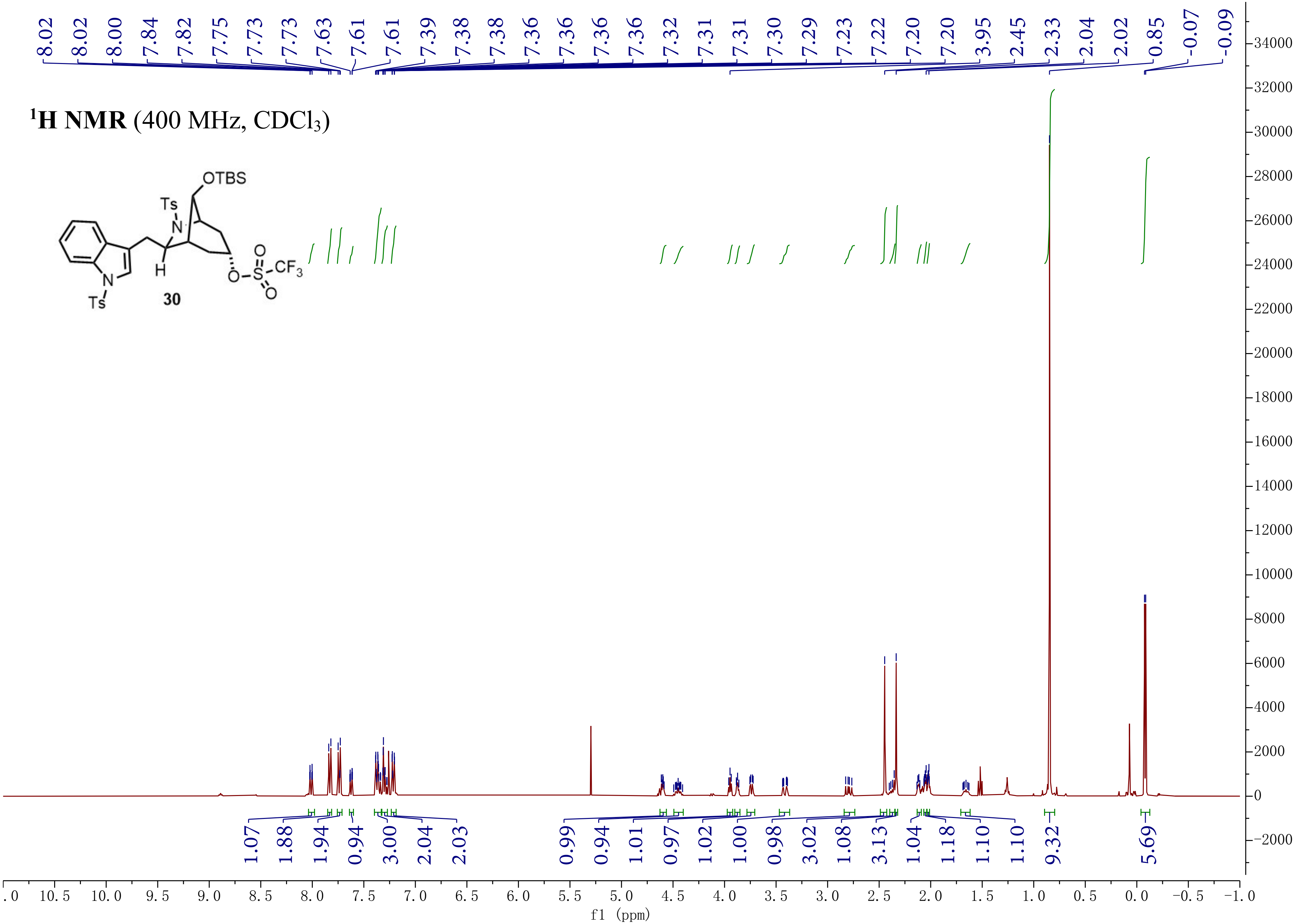

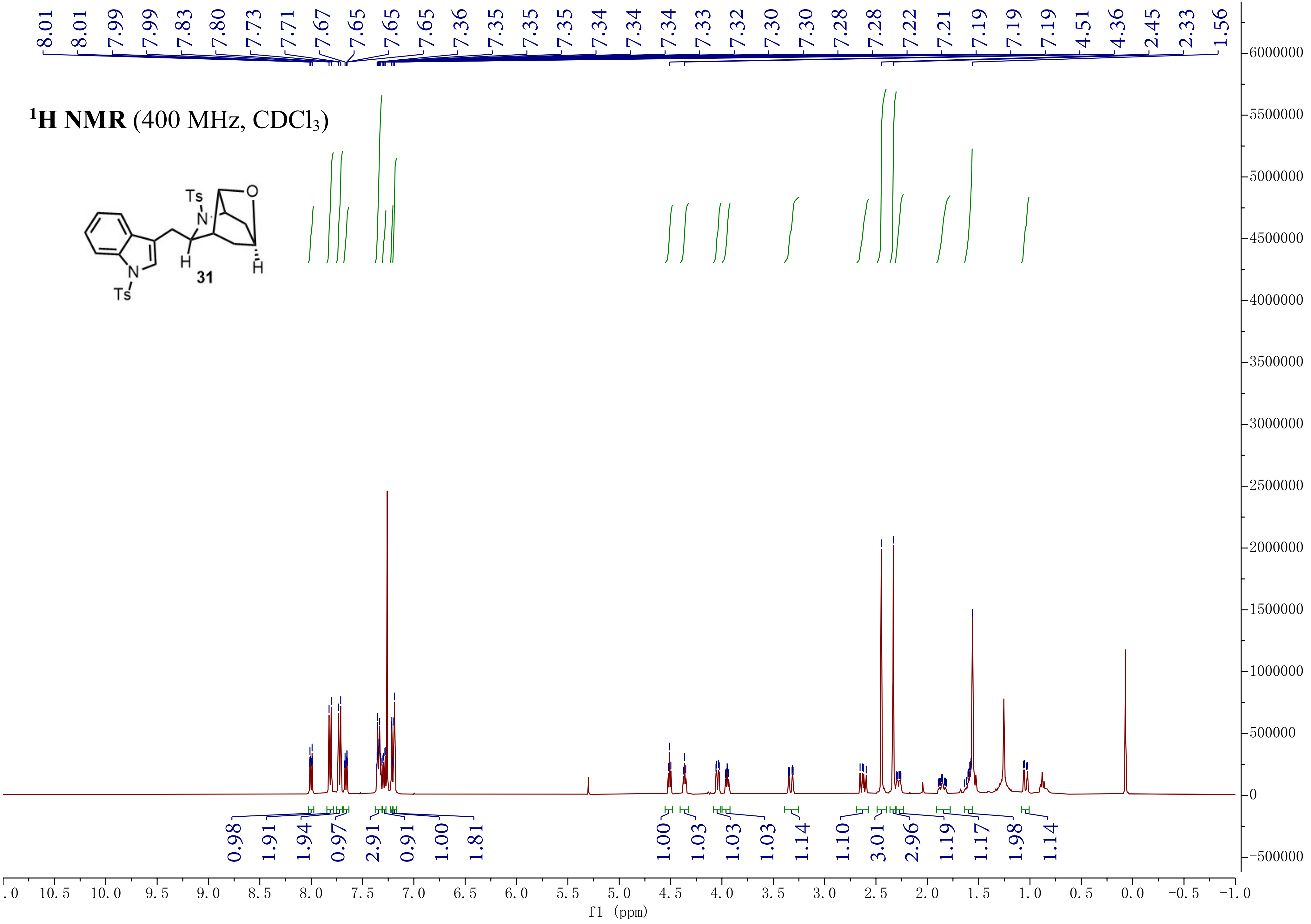

8.01
8.01
7.99
7.99
7.83
7.80
7.73
7.71
7.67
7.65
7.65
7.65
7.36
7.35
7.35
7.35
7.34
7.34
7.34
7.33
7.32
7.30
7.30
7.28
7.28
7.22
7.21
7.19
7.19
7.19
4.51
4.36
2.45
2.33
1.56
1H NMR (400 MHz, CDCl3)
Ts
N
O
H
H
N
Ts
31
0.98
1.91
1.94
0.97
2.91
0.91
1.00
1.81
1.00
1.03
1.03
1.03
1.14
1.10
3.01
2.96
1.19
1.17
1.98
1.14
f1 (ppm)

^{13}C NMR (100 MHz, $CDCl_3$)

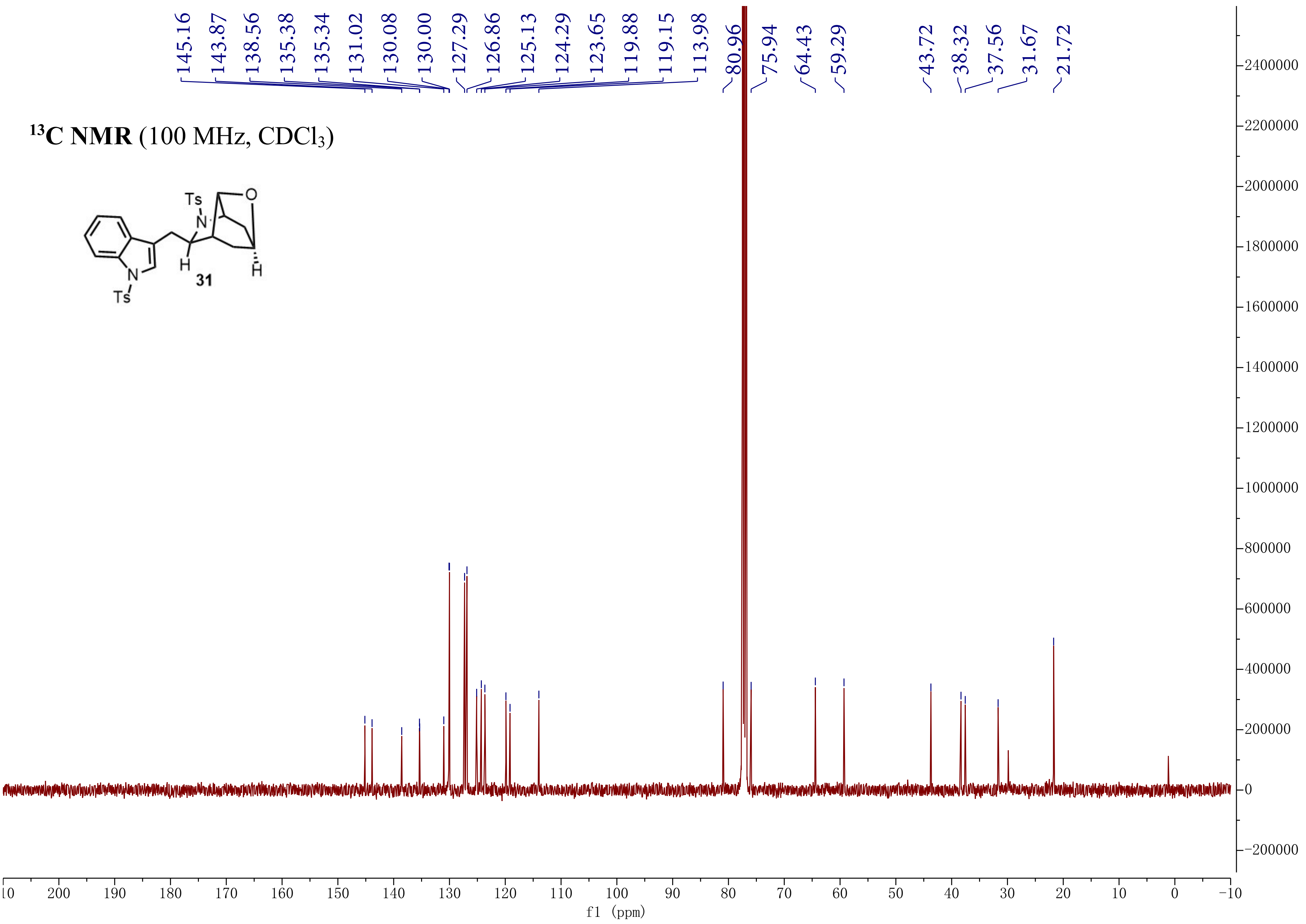

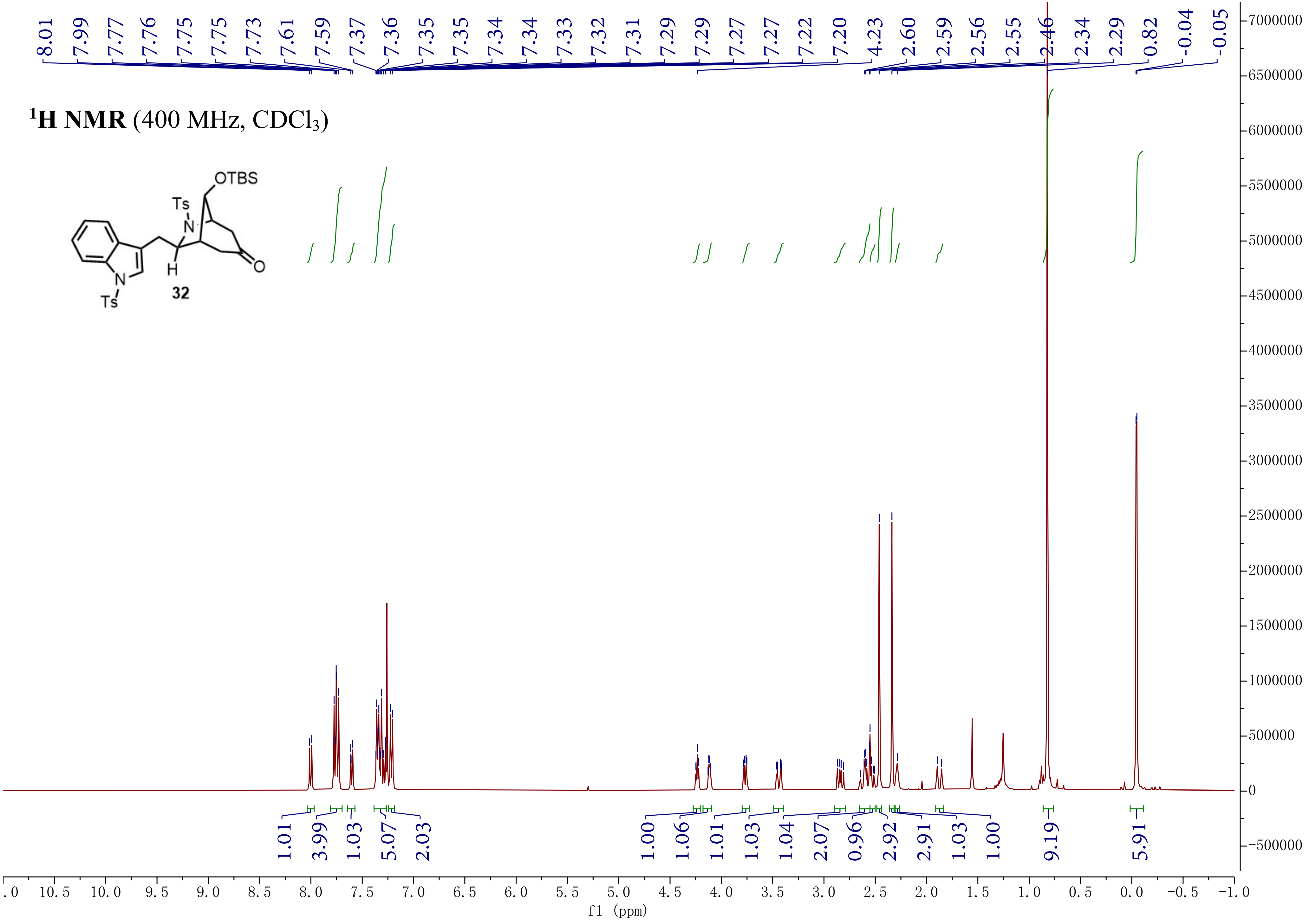

8.01
7.99
7.77
7.76
7.75
7.75
7.73
7.61
7.59
7.37
7.36
7.35
7.35
7.34
7.34
7.33
7.32
7.31
7.29
7.29
7.27
7.27
7.22
7.20
4.23
2.60
2.59
2.56
2.55
2.46
2.34
2.29
0.82
-0.04
-0.05
¹H NMR (400 MHz, CDCl₃)
OTBS
Ts
N
H
N
Ts
O
32
1.01
3.99
1.03
5.07
2.03
1.00
1.06
1.01
1.03
1.04
2.07
0.96
2.92
2.91
1.03
1.00
9.19
5.91
f1 (ppm)

^{13}C NMR (100 MHz, $CDCl_3$)

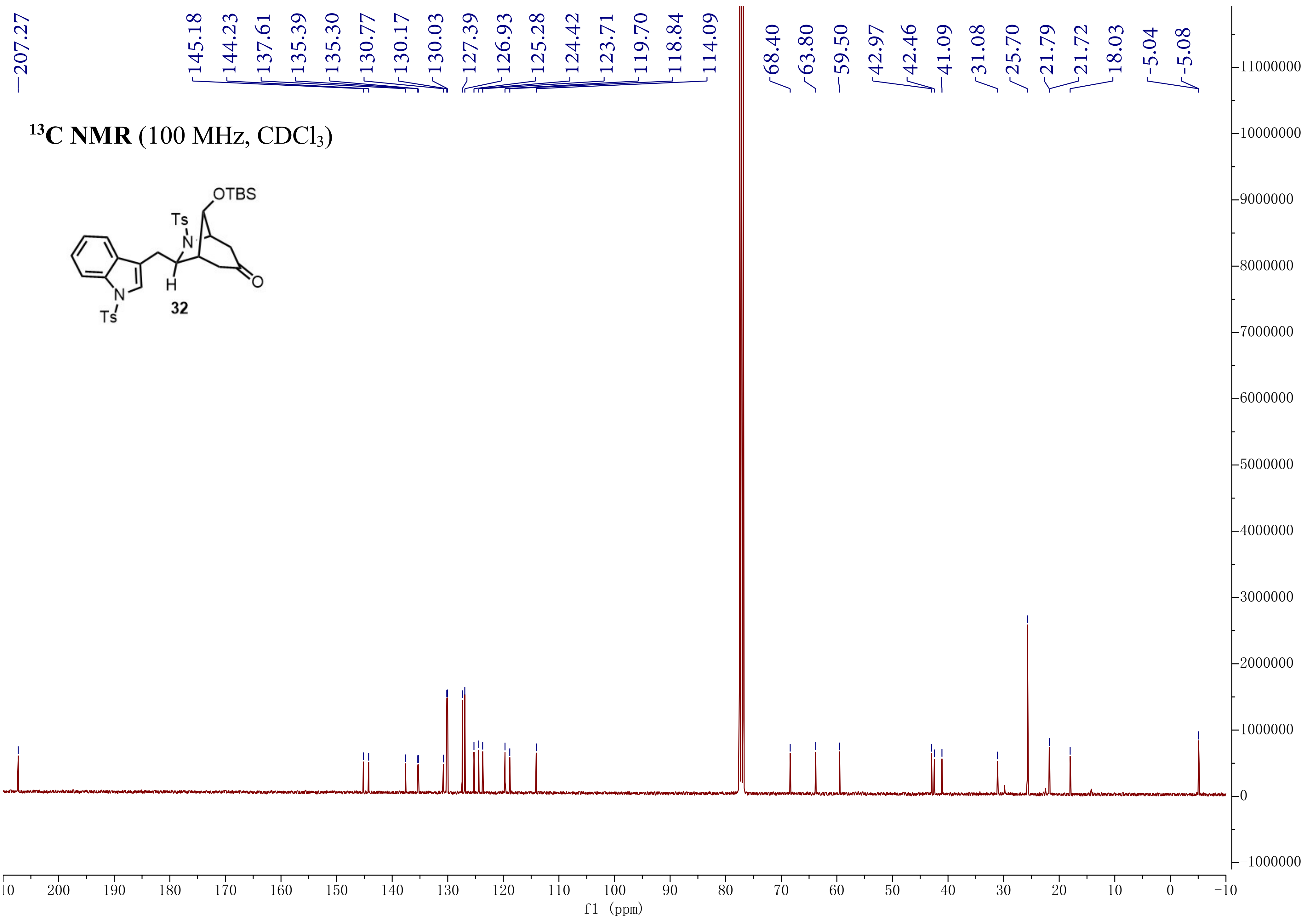

^{1}H NMR (400 MHz, $CDCl_3$)

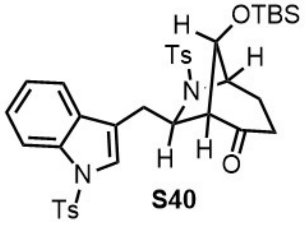

S40

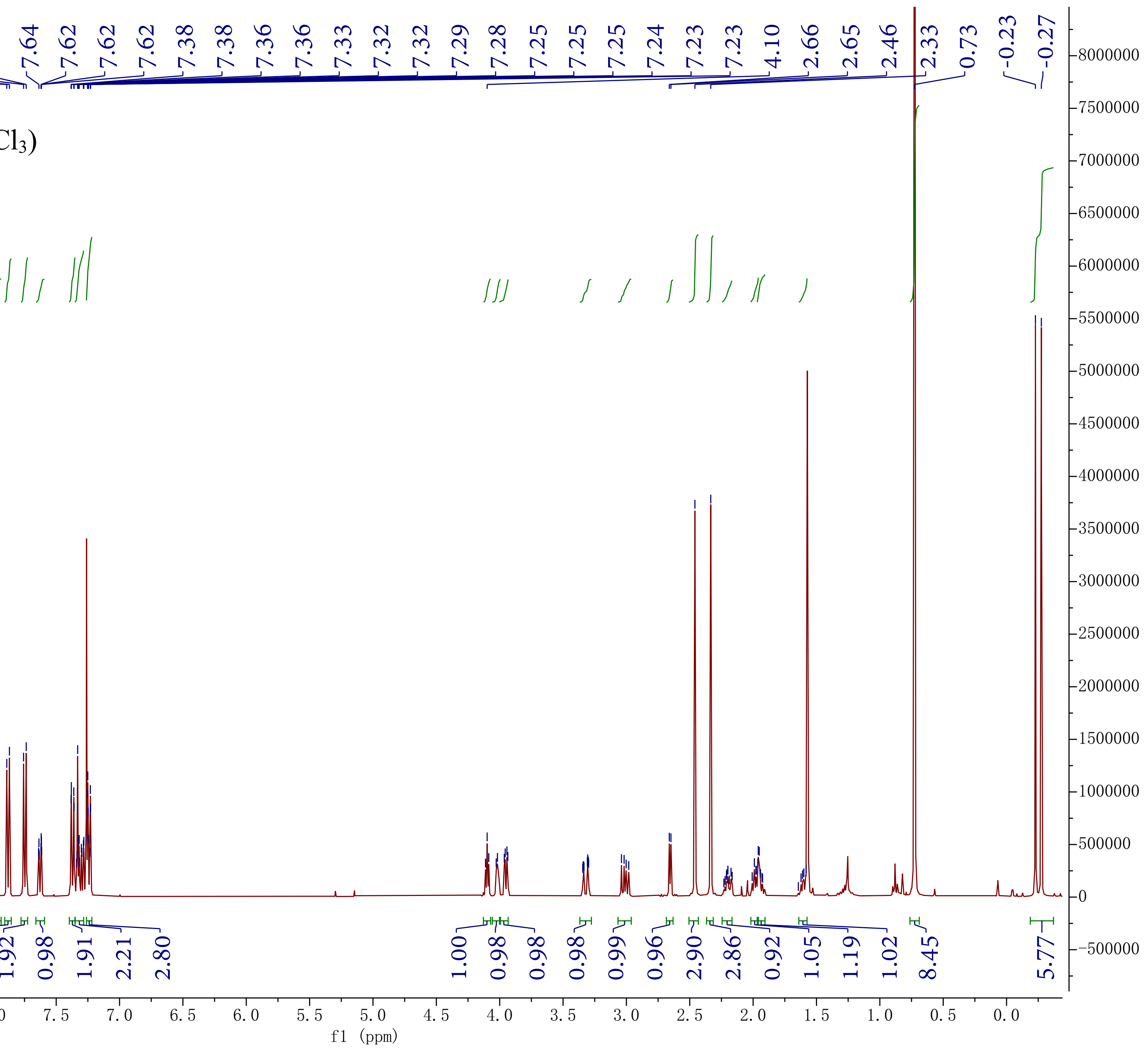

^{13}C NMR (100 MHz, $CDCl_3$)

S40

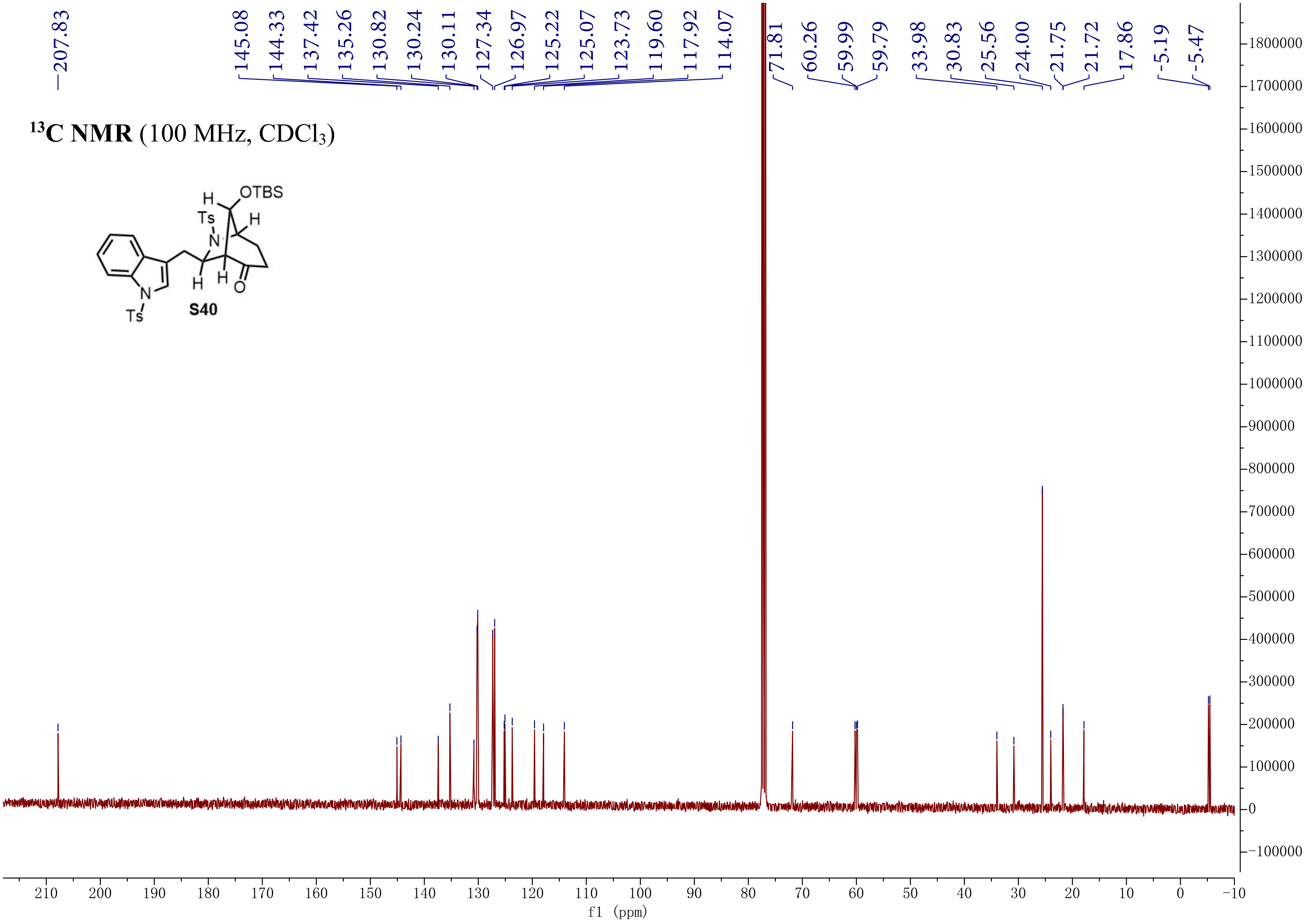

^{1}H NMR (400 MHz, $CDCl_3$)

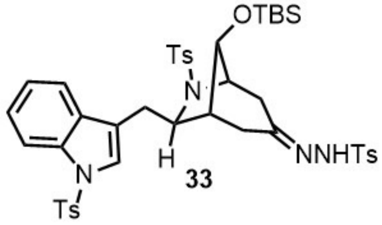

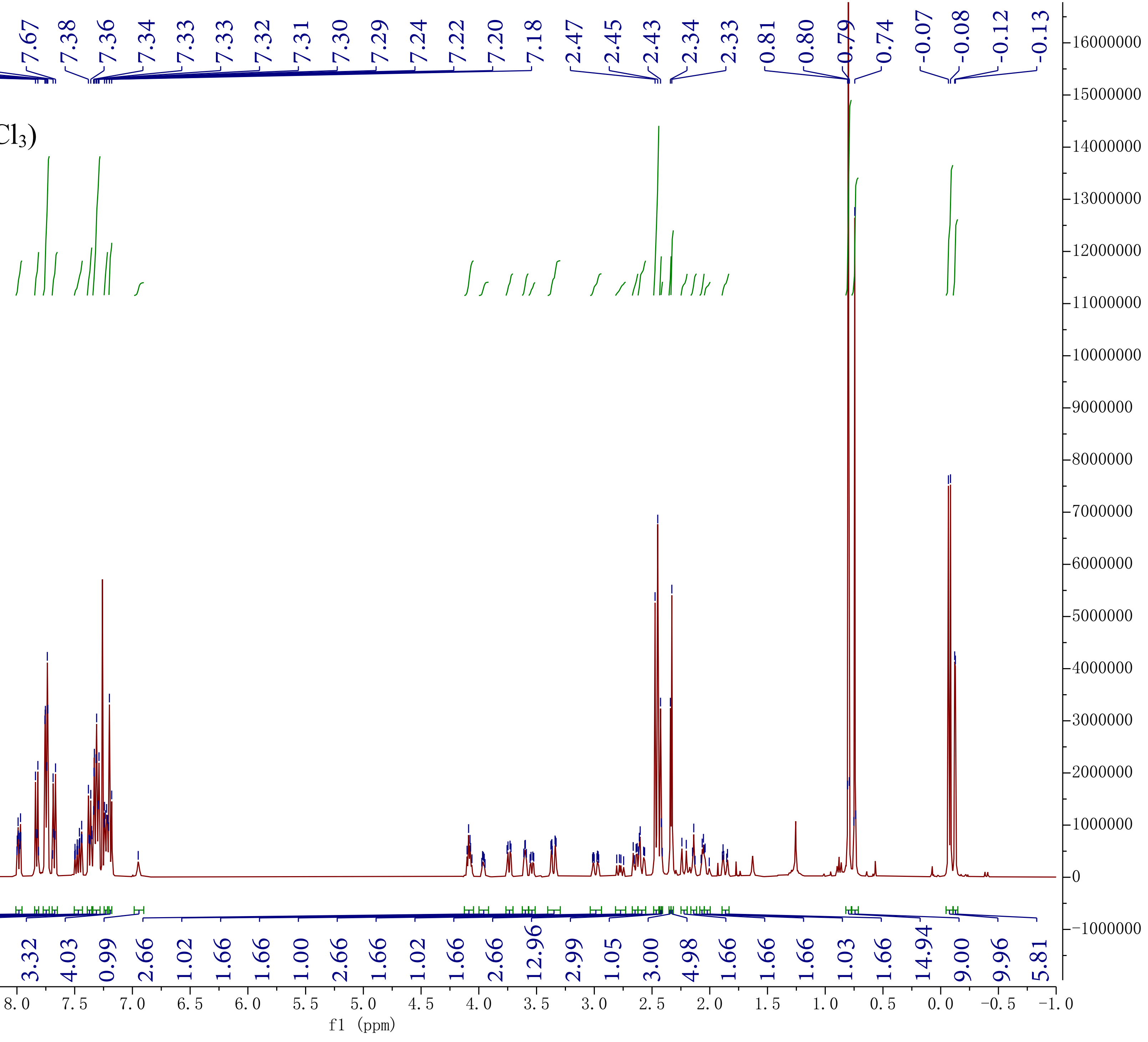

^{13}C NMR (100 MHz, $CDCl_3$)

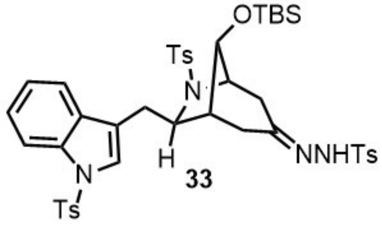

33

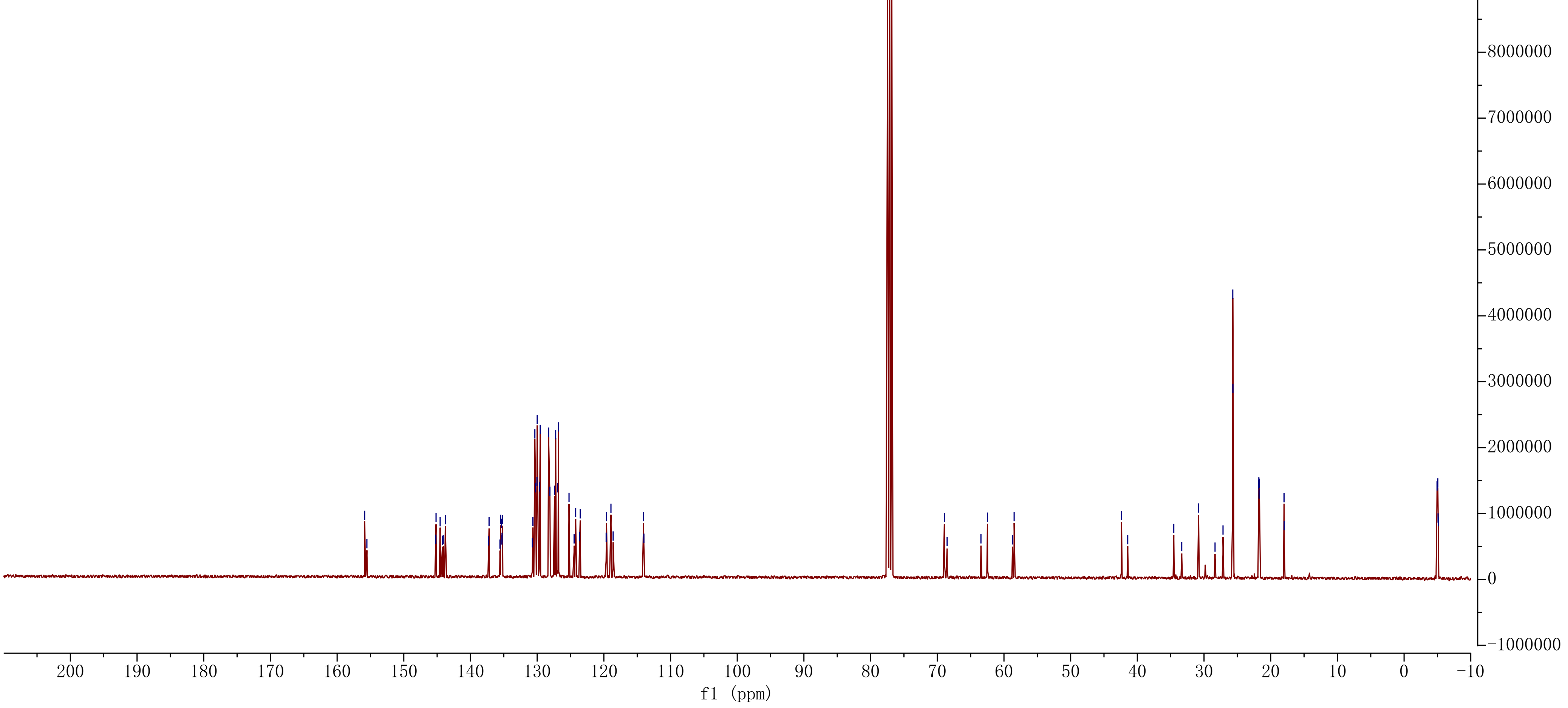

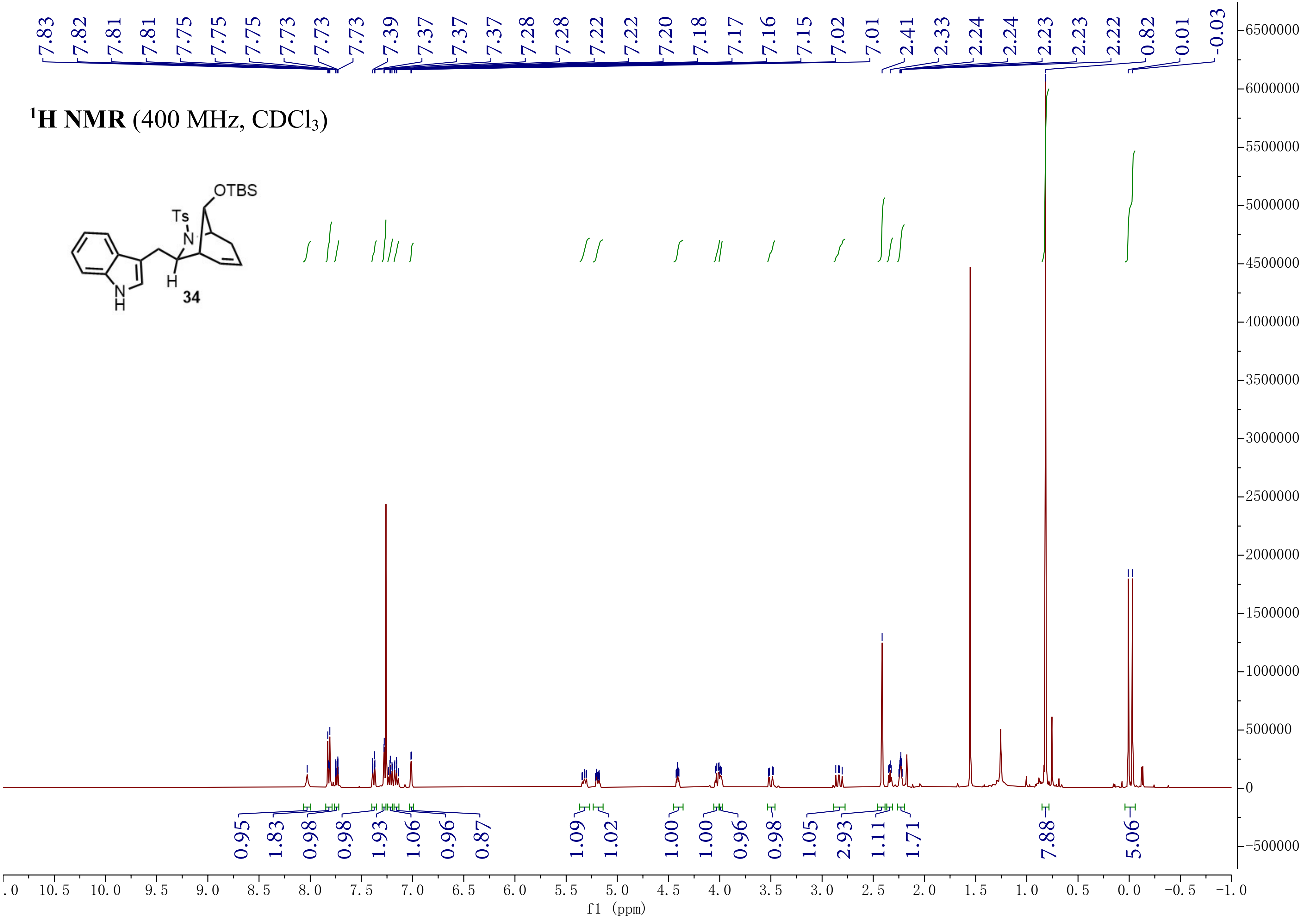

1H NMR (400 MHz, CDCl3)
OTBS
Ts
N
H
N
H
34
7.83 7.82 7.81 7.81 7.75 7.75 7.75 7.73 7.73 7.73 7.39 7.37 7.37 7.37 7.28 7.28 7.22 7.22 7.20 7.18 7.17 7.16 7.15 7.02 7.01 2.41 2.33 2.24 2.24 2.23 2.23 2.22 0.82 0.01 -0.03
0.95 1.83 0.98 0.98 1.93 1.06 0.96 0.87 1.09 1.02 1.00 1.00 0.96 0.98 1.05 2.93 1.11 1.71 7.88 5.06
f1 (ppm)

^{13}C NMR (100 MHz, $CDCl_3$)

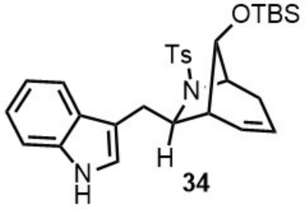

34

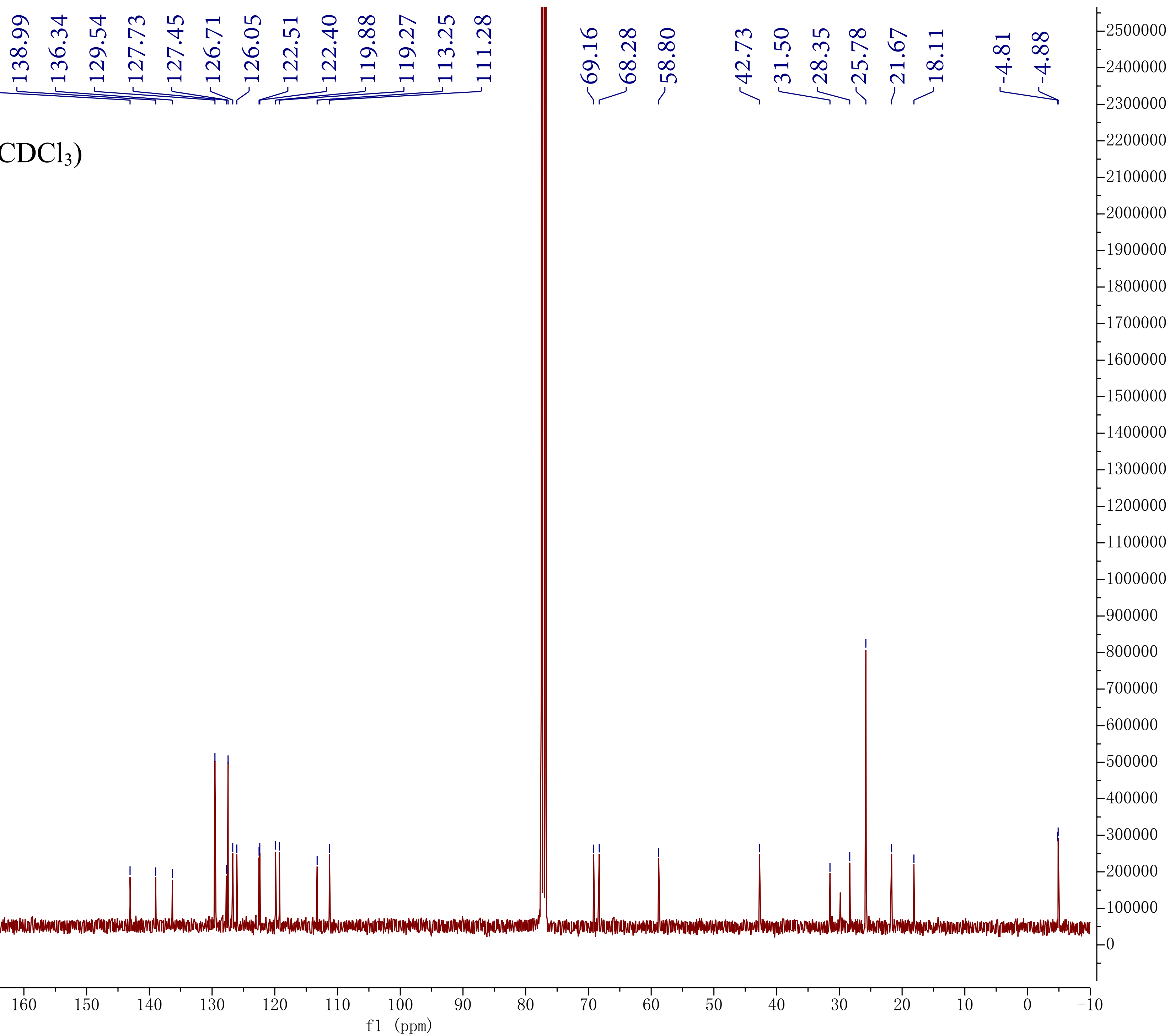

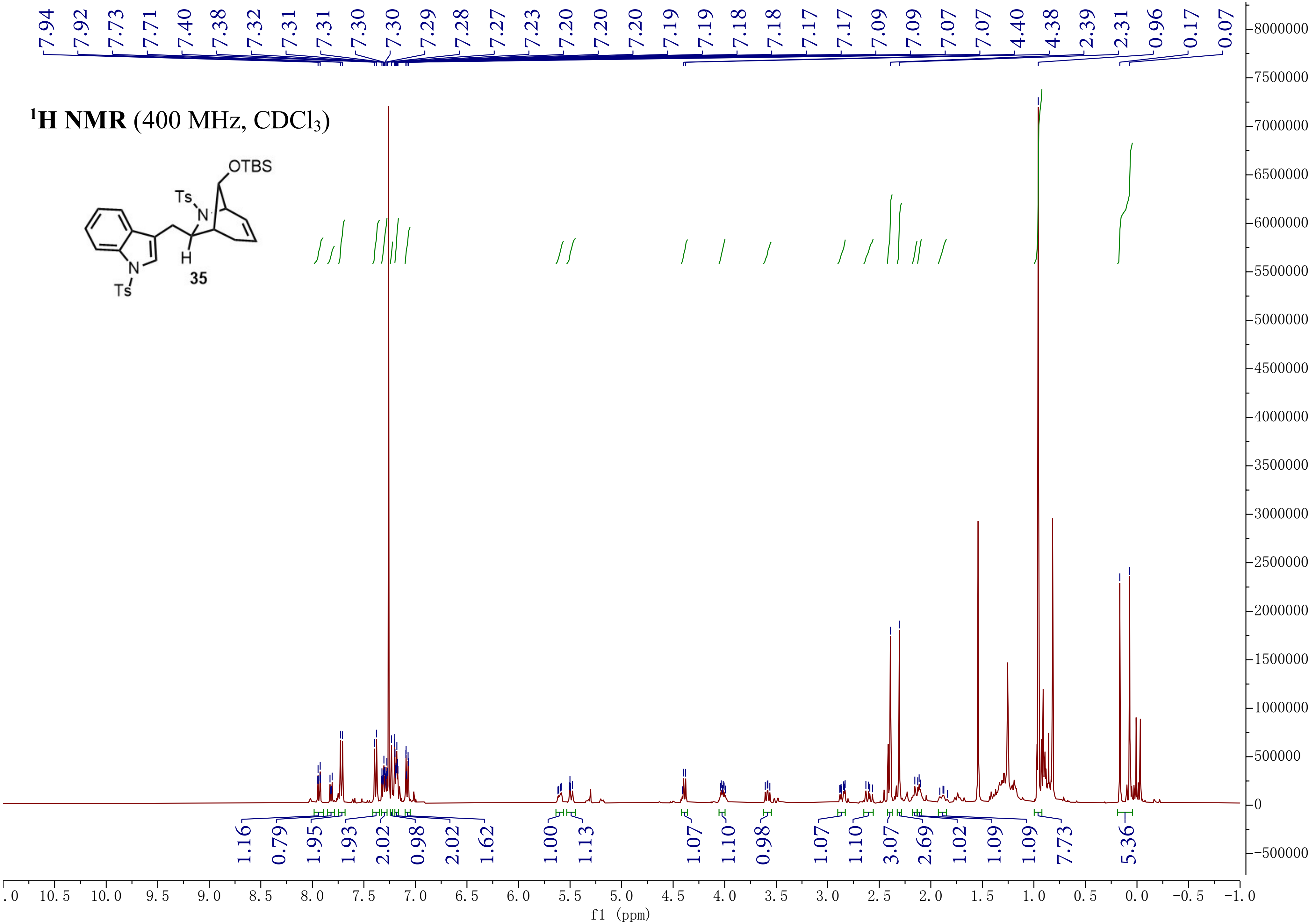

^{1}H NMR (400 MHz, $CDCl_3$)
OTBS
Ts
N
H
Ts
N
35
7.94 7.92 7.73 7.71 7.40 7.38 7.32 7.31 7.31 7.30 7.30 7.29 7.28 7.27 7.23 7.20 7.20 7.20 7.19 7.19 7.18 7.18 7.17 7.17 7.09 7.09 7.07 7.07 4.40 4.38 2.39 2.31 0.96 0.17 0.07
1.16 0.79 1.95 1.93 2.02 0.98 2.02 1.62 1.00 1.13 1.07 1.10 0.98 1.07 1.10 3.07 2.69 1.02 1.09 1.09 7.73 5.36
8000000 7500000 7000000 6500000 6000000 5500000 5000000 4500000 4000000 3500000 3000000 2500000 2000000 1500000 1000000 500000 0 -500000
.0 10.5 10.0 9.5 9.0 8.5 8.0 7.5 7.0 6.5 6.0 5.5 5.0 4.5 4.0 3.5 3.0 2.5 2.0 1.5 1.0 0.5 0.0 -0.5 -1.0
f1 (ppm)

^{13}C NMR (100 MHz, $CDCl_3$)

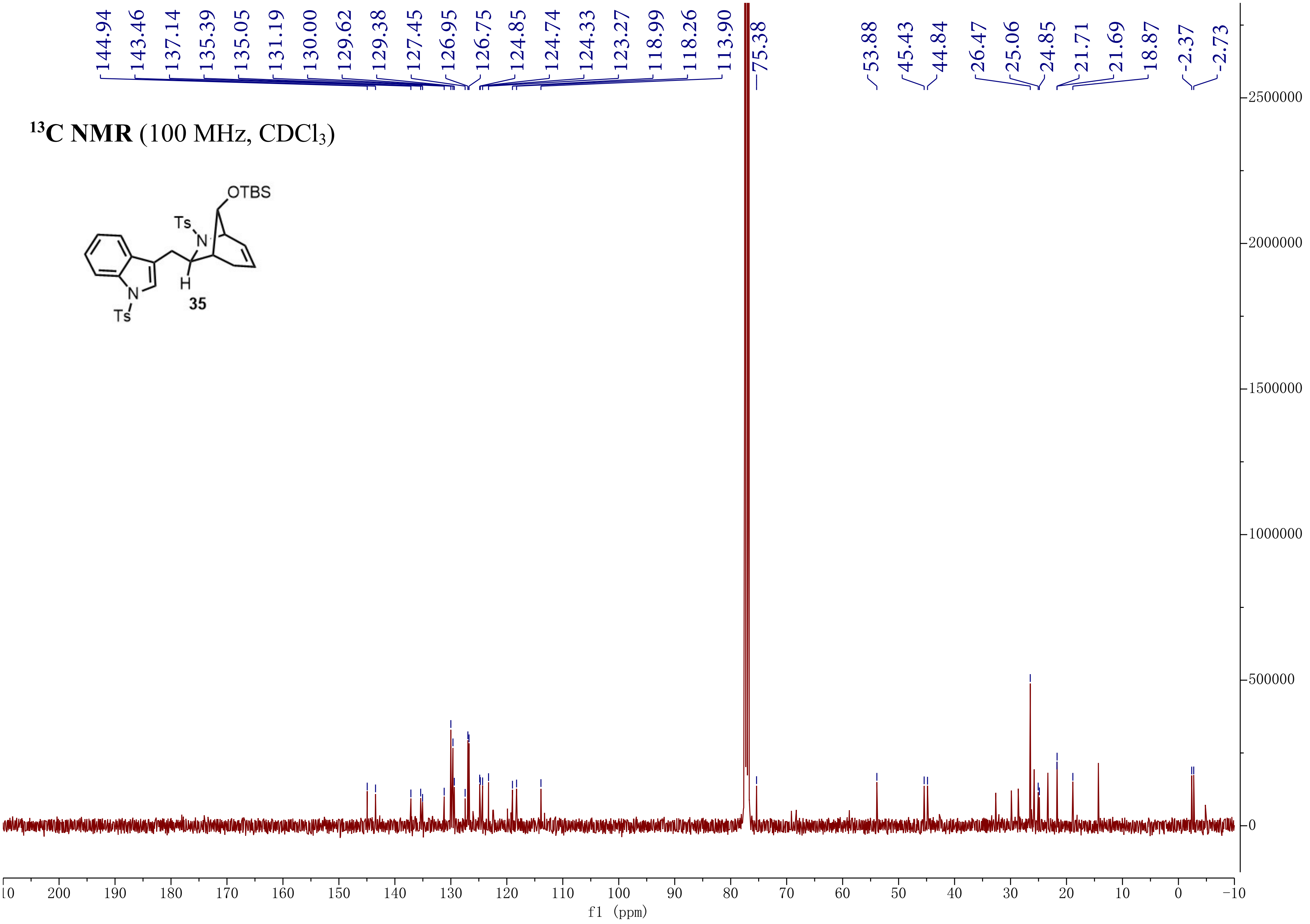

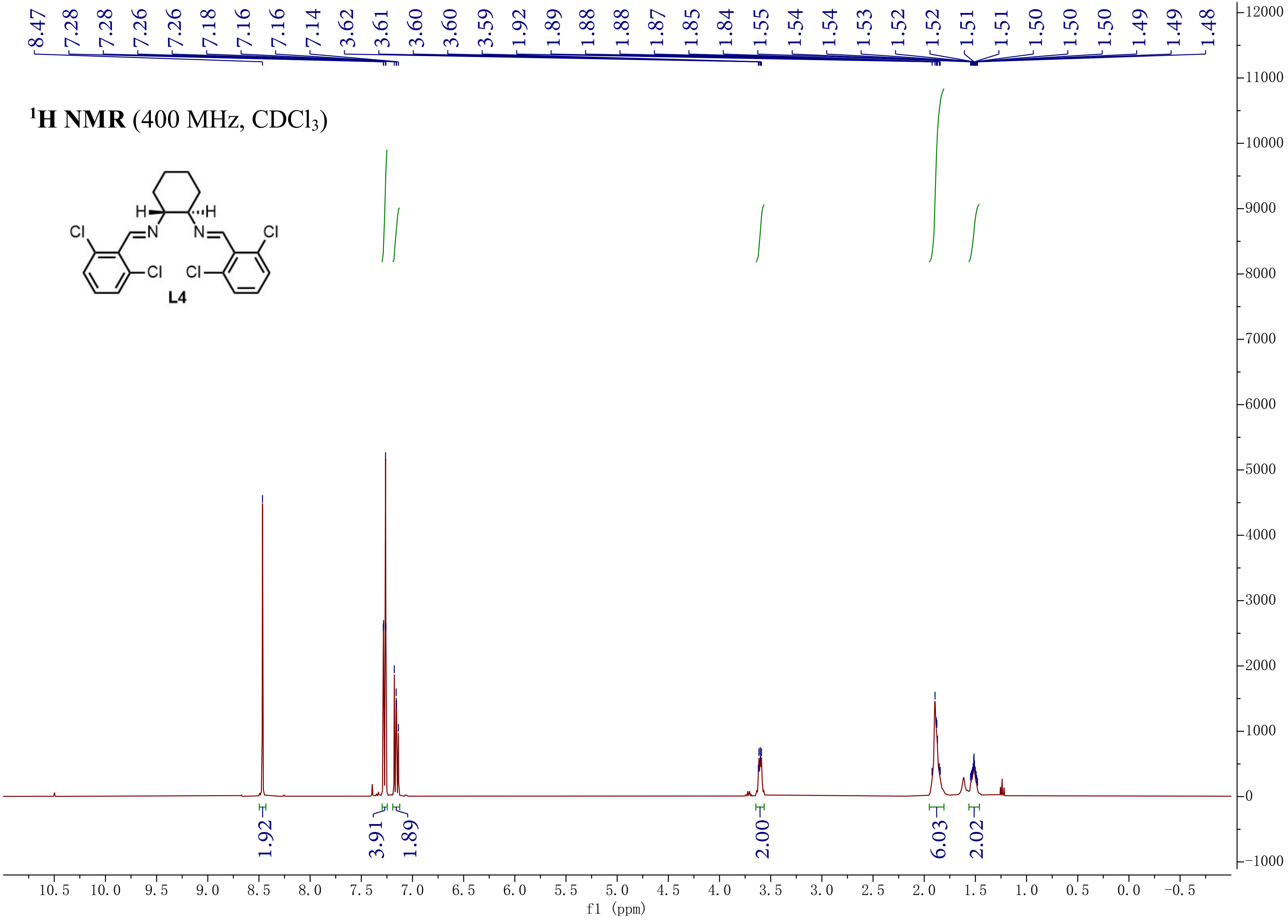

8.47 7.28 7.28 7.26 7.26 7.18 7.16 7.16 7.14 3.62 3.61 3.60 3.60 3.59 1.92 1.89 1.88 1.88 1.87 1.85 1.84 1.55 1.54 1.54 1.53 1.52 1.52 1.51 1.51 1.50 1.50 1.50 1.49 1.49 1.48
^{1}H NMR (400 MHz, $CDCl_3$)
Cl
H
N
Cl
L4
1.92
3.91
1.89
2.00
6.03
2.02
f1 (ppm)

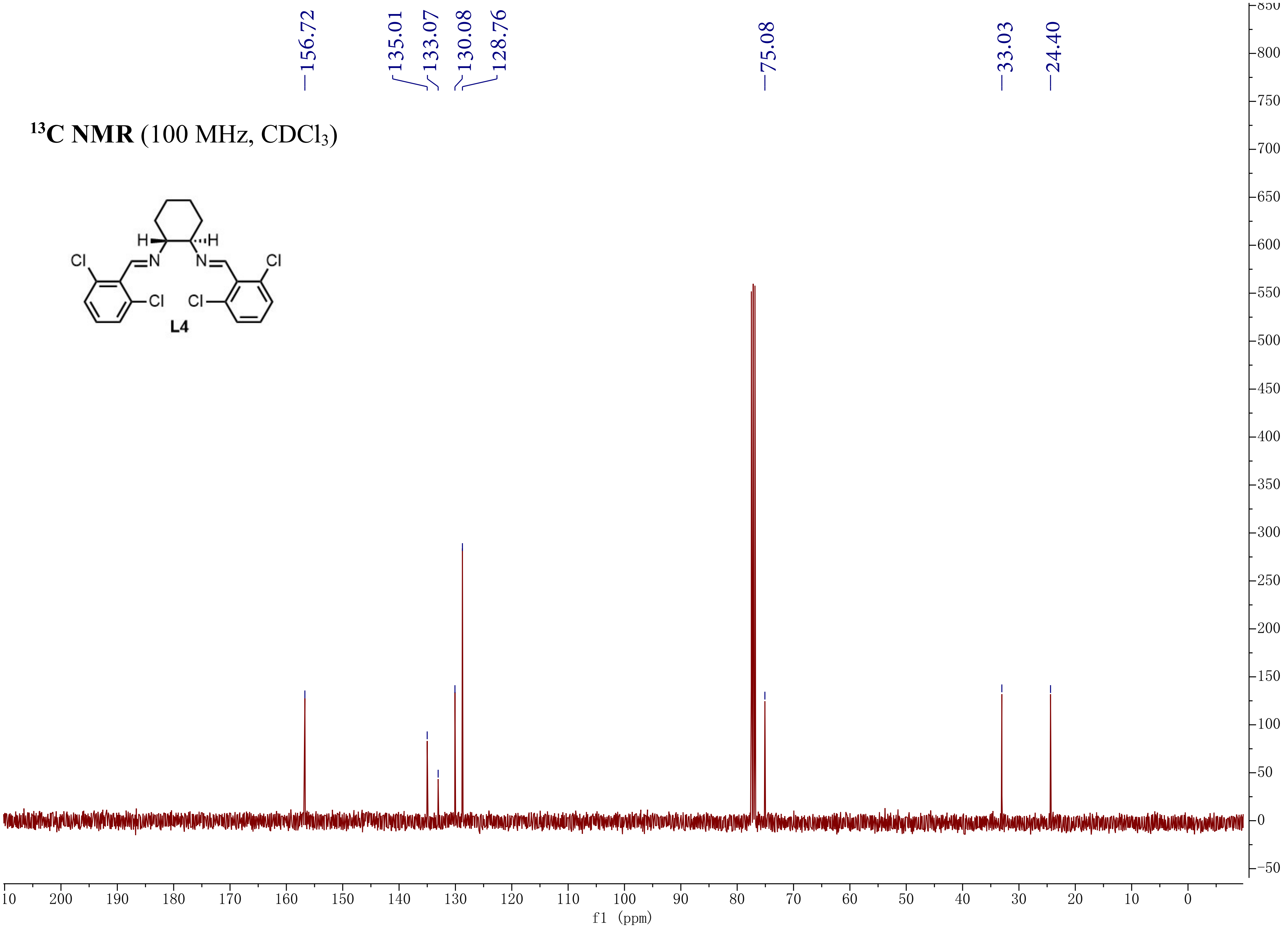

^{13}C NMR (100 MHz, $CDCl_3$)

L4

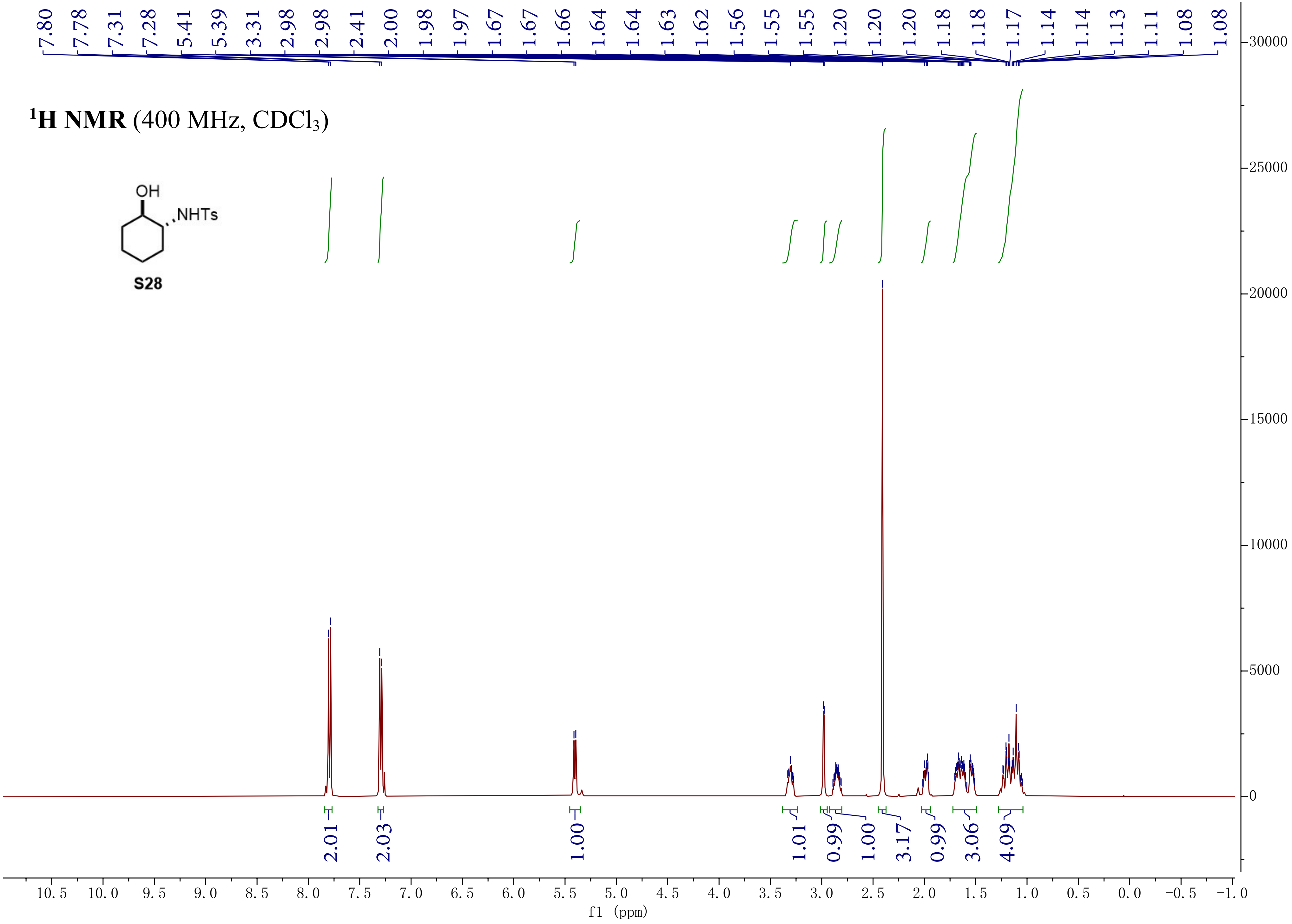

7.80 7.78 7.31 7.28 5.41 5.39 3.31 2.98 2.98 2.41 2.00 1.98 1.97 1.67 1.67 1.66 1.64 1.64 1.63 1.62 1.56 1.55 1.55 1.20 1.20 1.20 1.18 1.18 1.17 1.14 1.14 1.13 1.11 1.08 1.08
¹H NMR (400 MHz, CDCl₃)
2.01 2.03 1.00 1.01 0.99 1.00 3.17 0.99 3.06 4.09
10.5 10.0 9.5 9.0 8.5 8.0 7.5 7.0 6.5 6.0 5.5 5.0 4.5 4.0 3.5 3.0 2.5 2.0 1.5 1.0 0.5 0.0 -0.5 -1.0
f1 (ppm)
30000 25000 20000 15000 10000 5000 0
OH
NHTs

S28

^{13}C NMR (100 MHz, $CDCl_3$)

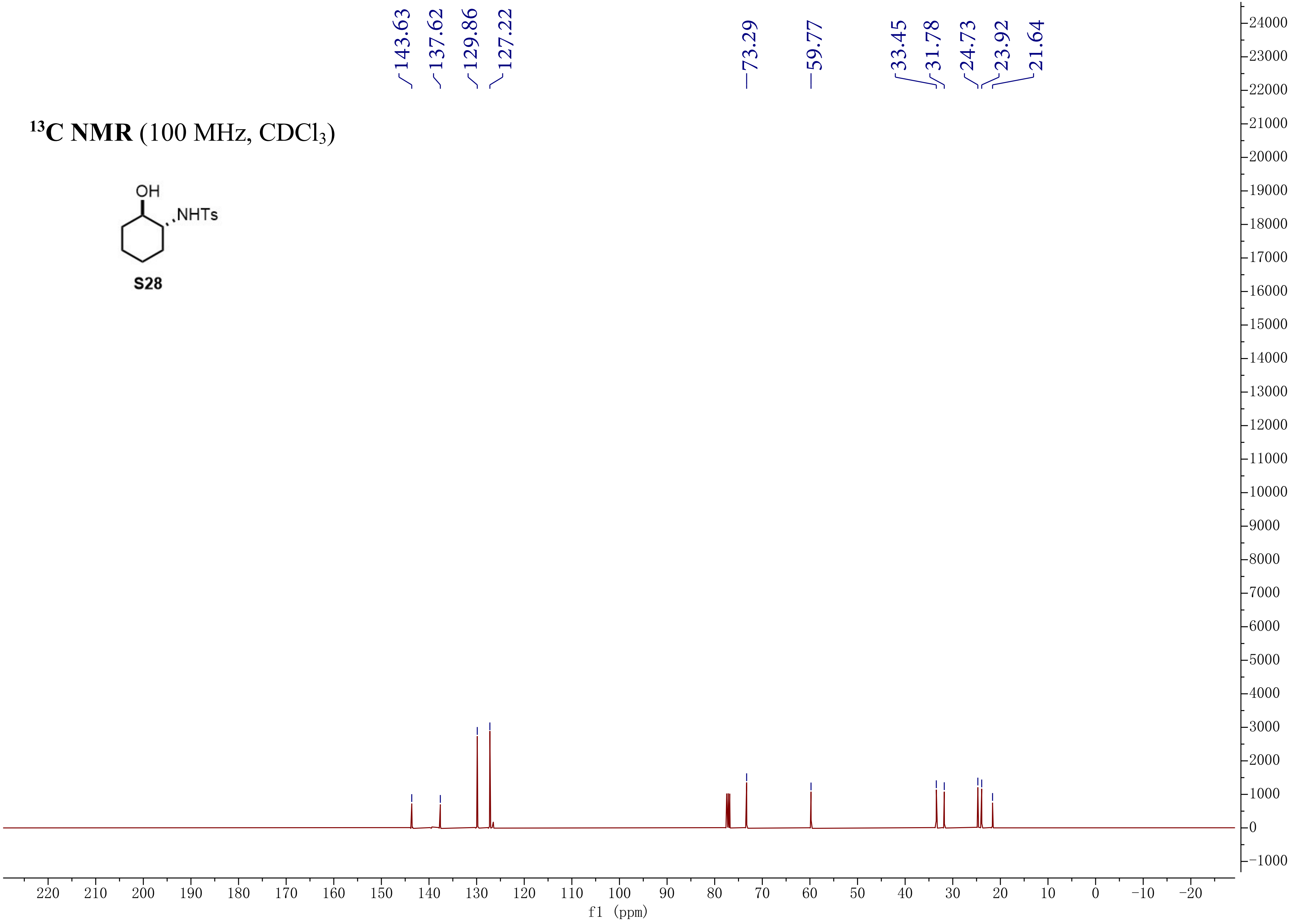

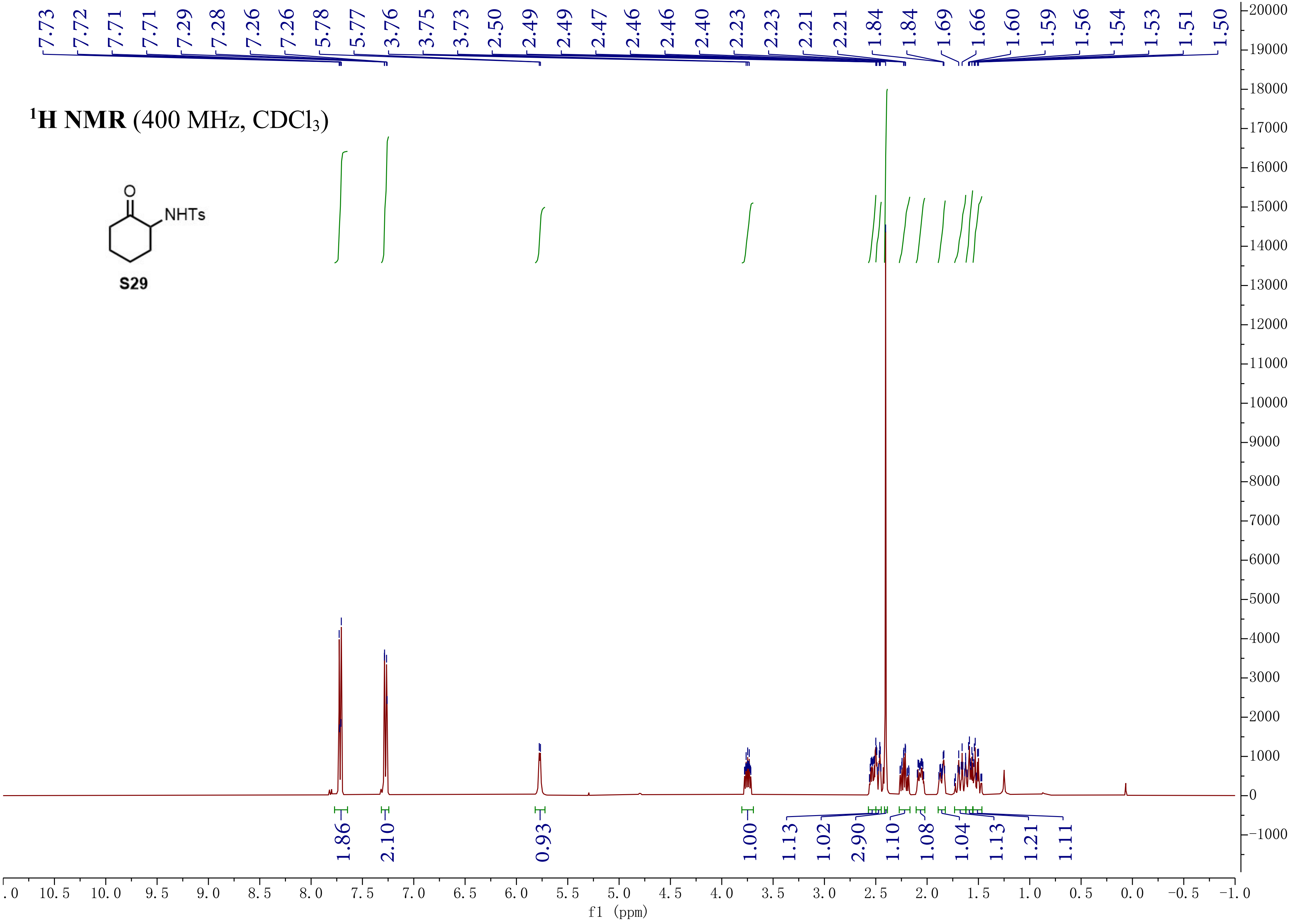

1H NMR (400 MHz, CDCl3)
NHTs
O
S29
7.73 7.72 7.71 7.71 7.29 7.28 7.26 7.26 5.78 5.77 3.76 3.75 3.73 2.50 2.49 2.49 2.47 2.46 2.46 2.40 2.23 2.23 2.21 2.21 1.84 1.84 1.69 1.66 1.60 1.59 1.56 1.54 1.53 1.51 1.50
1.86 2.10 0.93 1.00 1.13 1.02 2.90 1.10 1.08 1.04 1.13 1.21 1.11
f1 (ppm)

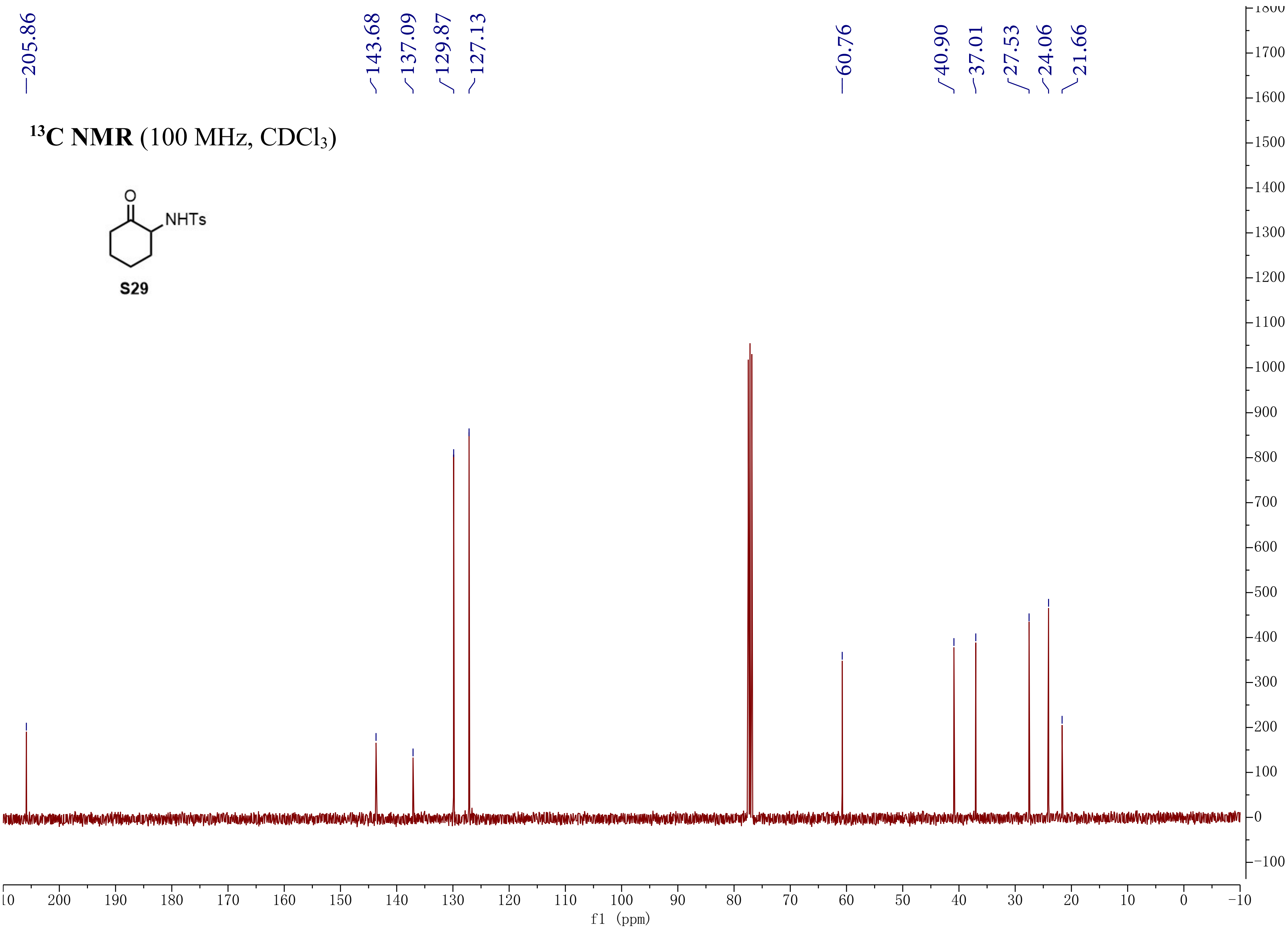

205.86
143.68
137.09
129.87
127.13
60.76
40.90
37.01
27.53
24.06
21.66
^{13}C NMR (100 MHz, CDCl$_3$)
O
NHTs
S29
f1 (ppm)

^{1}H NMR (400 MHz, $CDCl_3$)

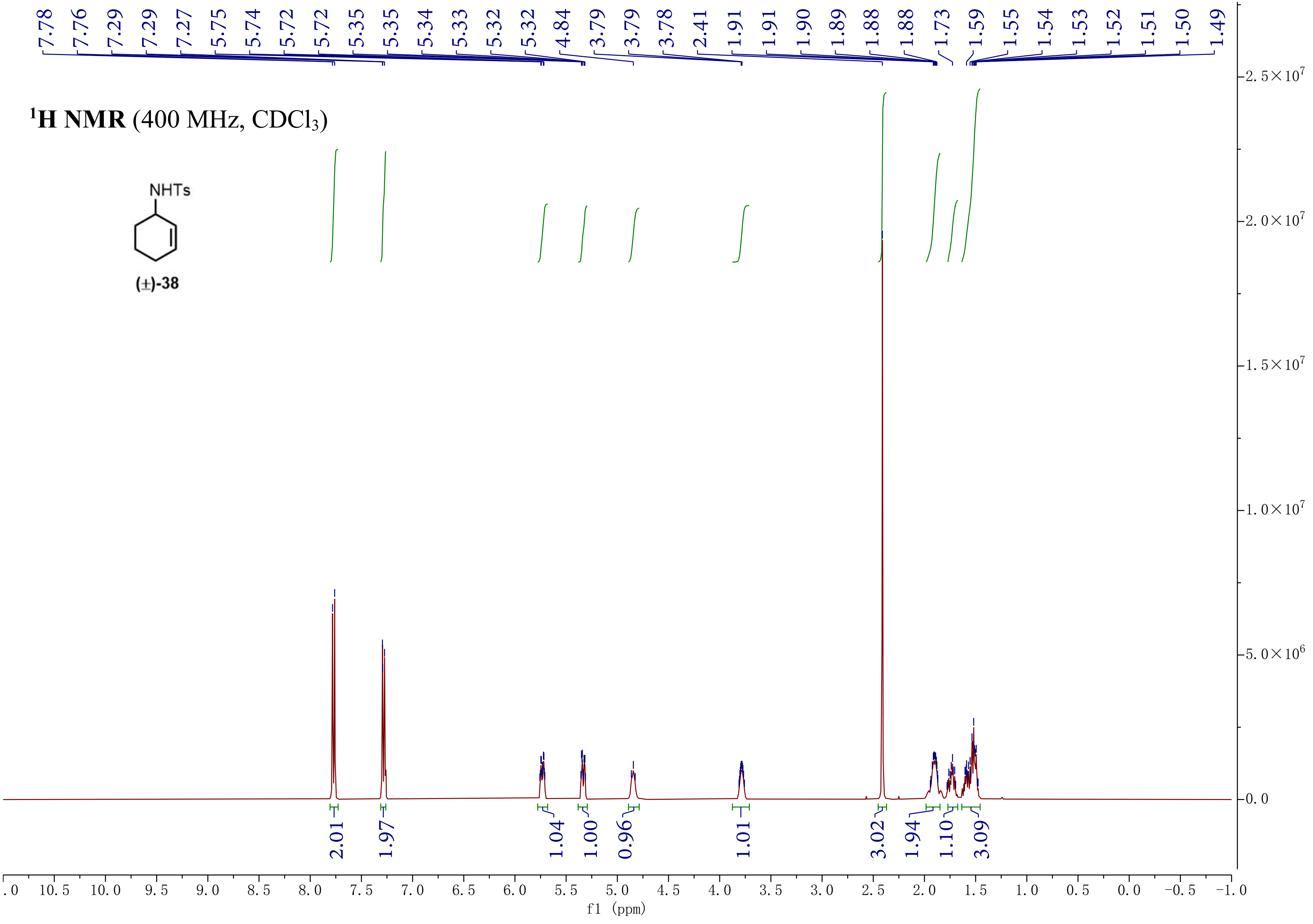

(±)-38

^{13}C NMR (100 MHz, $CDCl_3$)

(±)-38

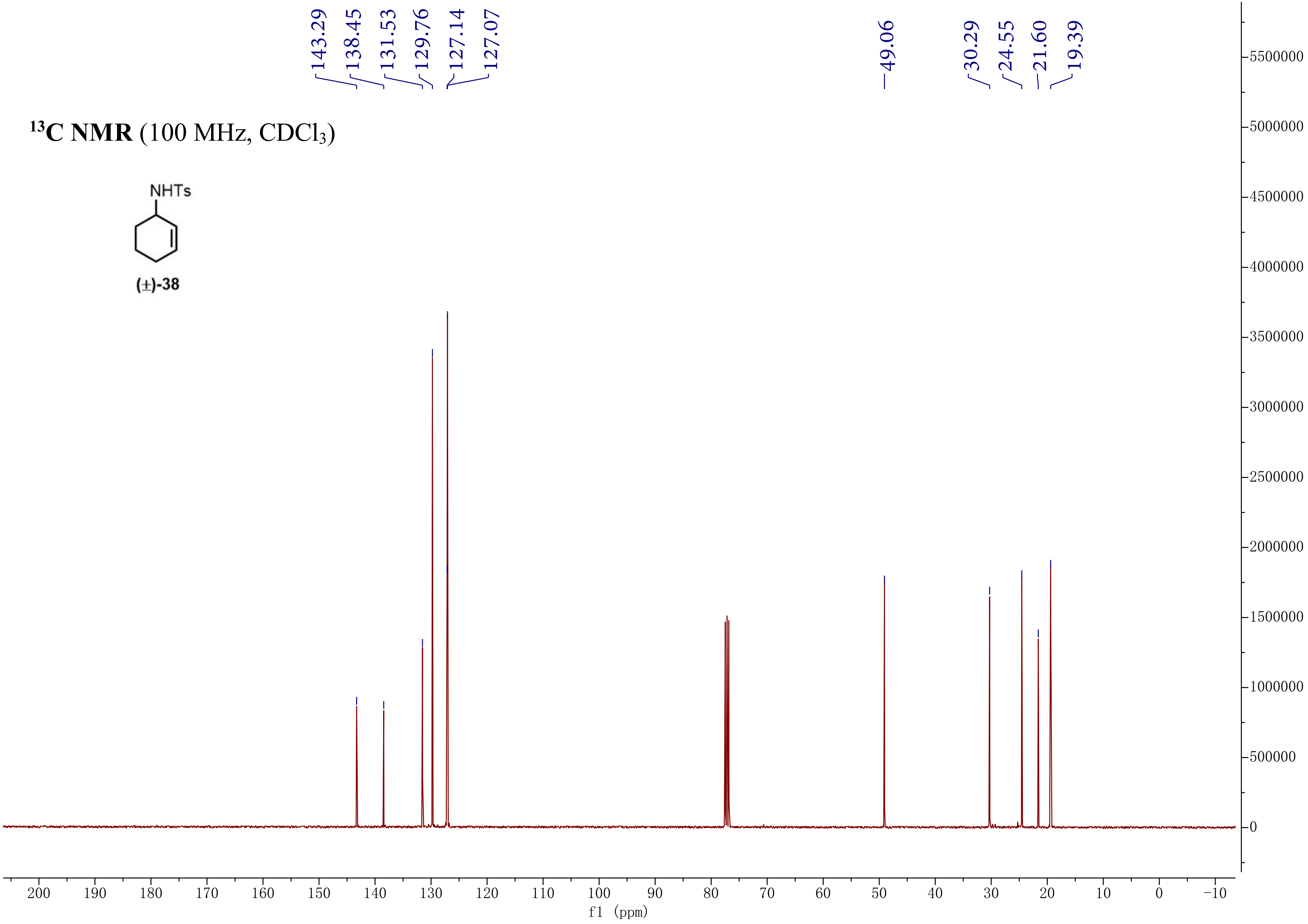

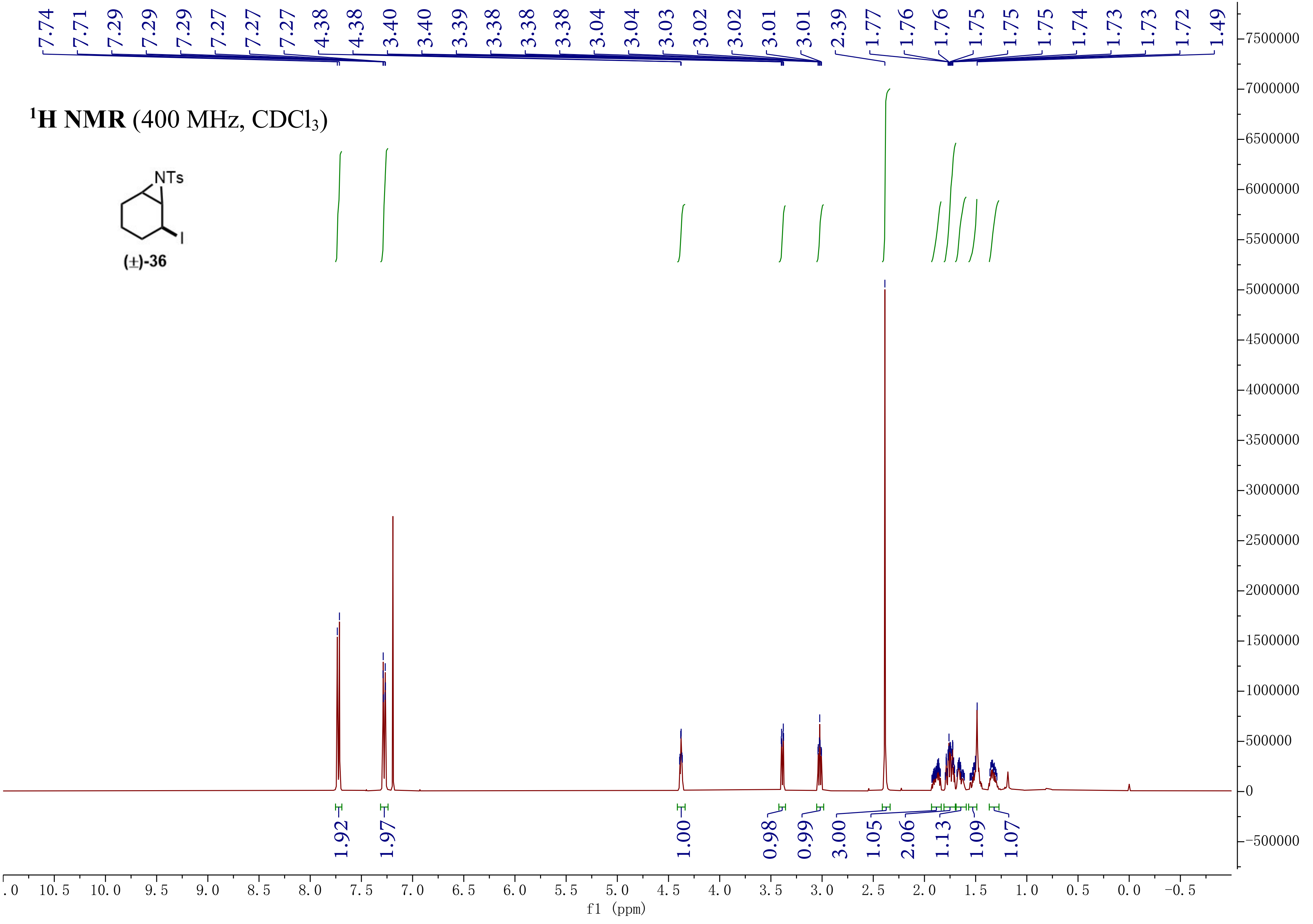

¹H NMR (400 MHz, CDCl3)
NTs
I
(±)-36
7.74 7.71 7.29 7.29 7.29 7.27 7.27 7.27 4.38 4.38 3.40 3.40 3.39 3.38 3.38 3.38 3.04 3.04 3.03 3.02 3.02 3.01 3.01 2.39 1.77 1.76 1.76 1.75 1.75 1.75 1.74 1.73 1.73 1.72 1.49
1.92 1.97 1.00 0.98 0.99 3.00 1.05 2.06 1.13 1.09 1.07
f1 (ppm)

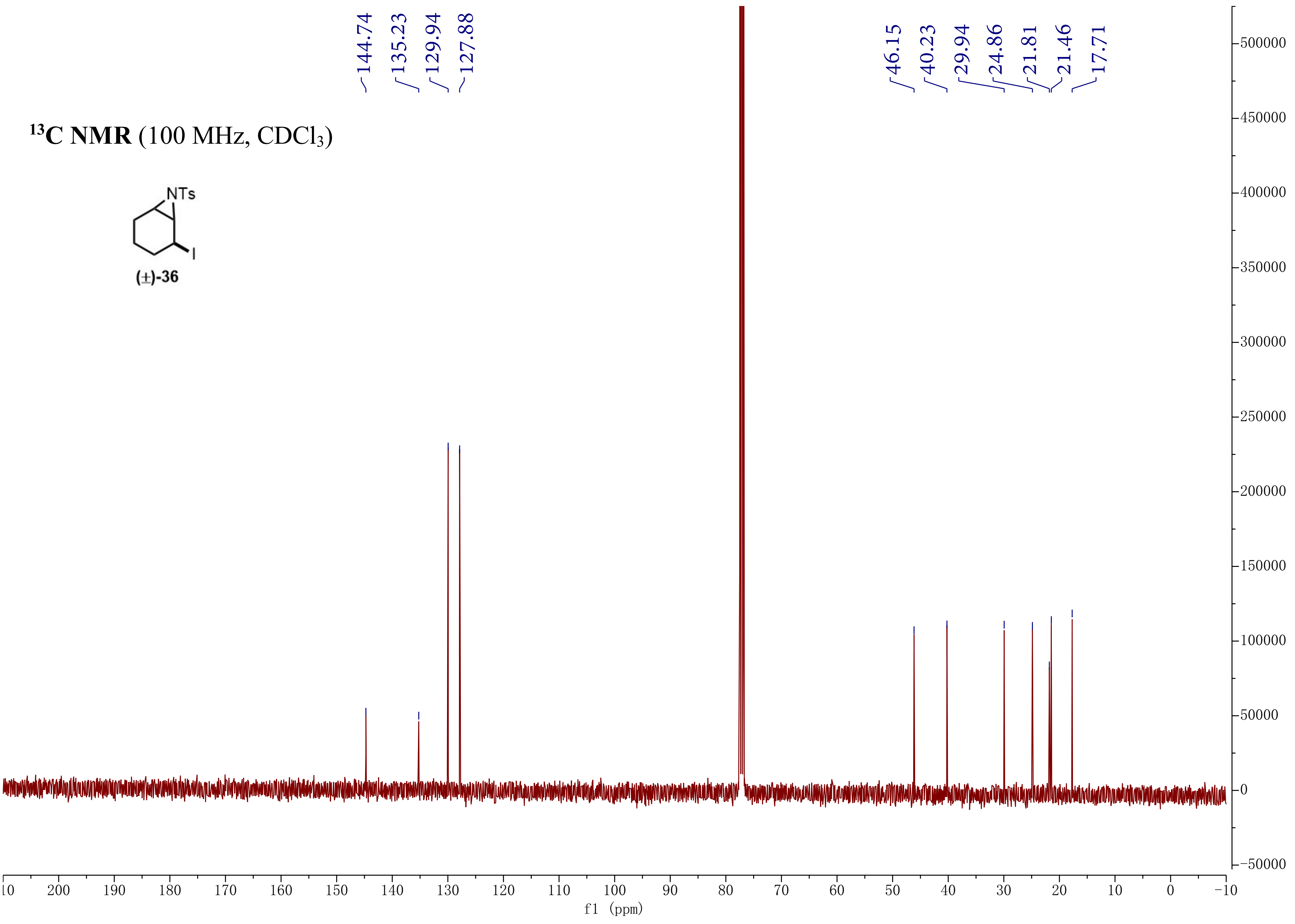

13C NMR (100 MHz, CDCl3)
NTs
I
(±)-36
144.74
135.23
129.94
127.88
46.15
40.23
29.94
24.86
21.81
21.46
17.71
f1 (ppm)

^{1}H NMR (400 MHz, $CDCl_3$)

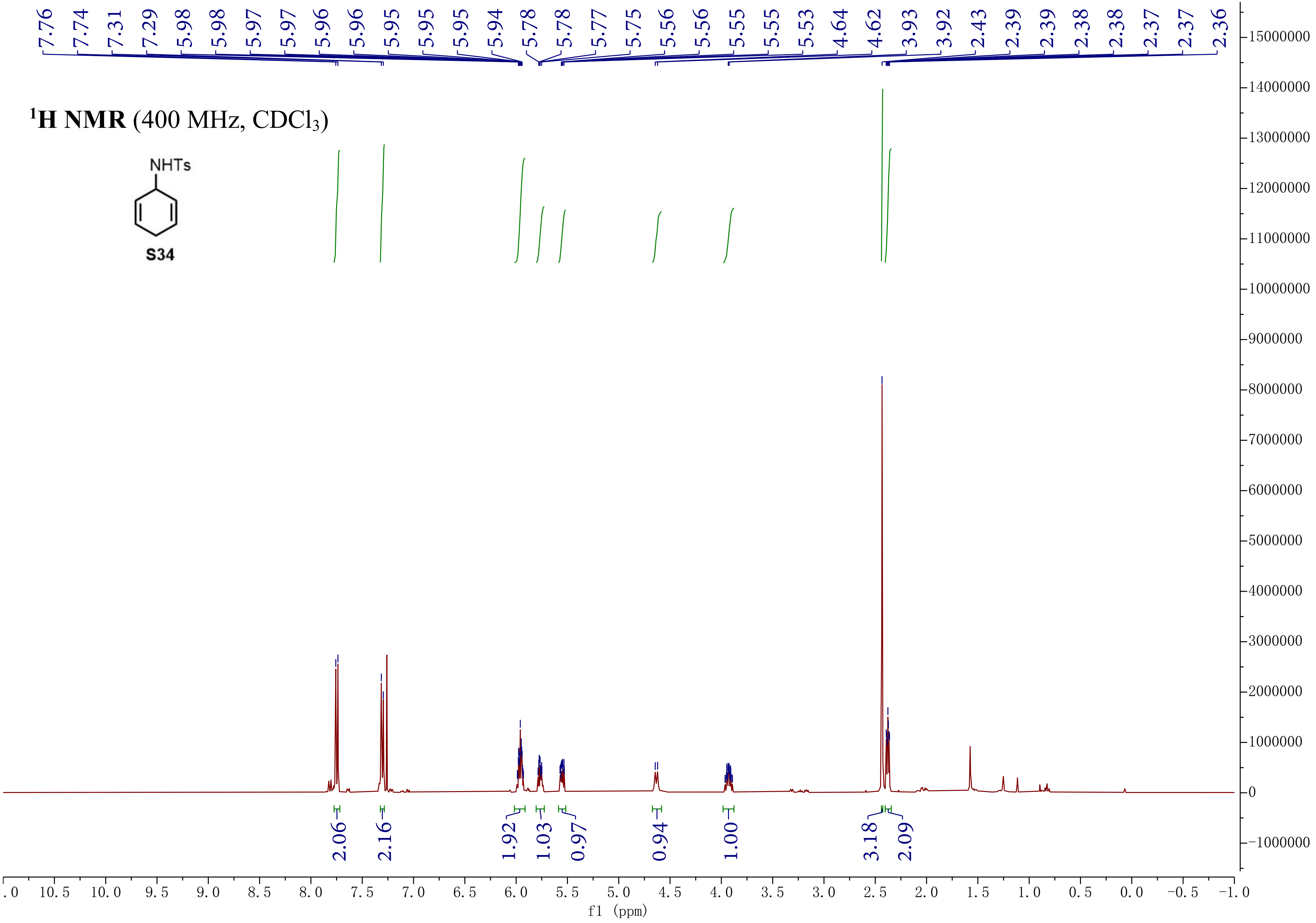

^{13}**C NMR** (100 MHz, $CDCl_3$)

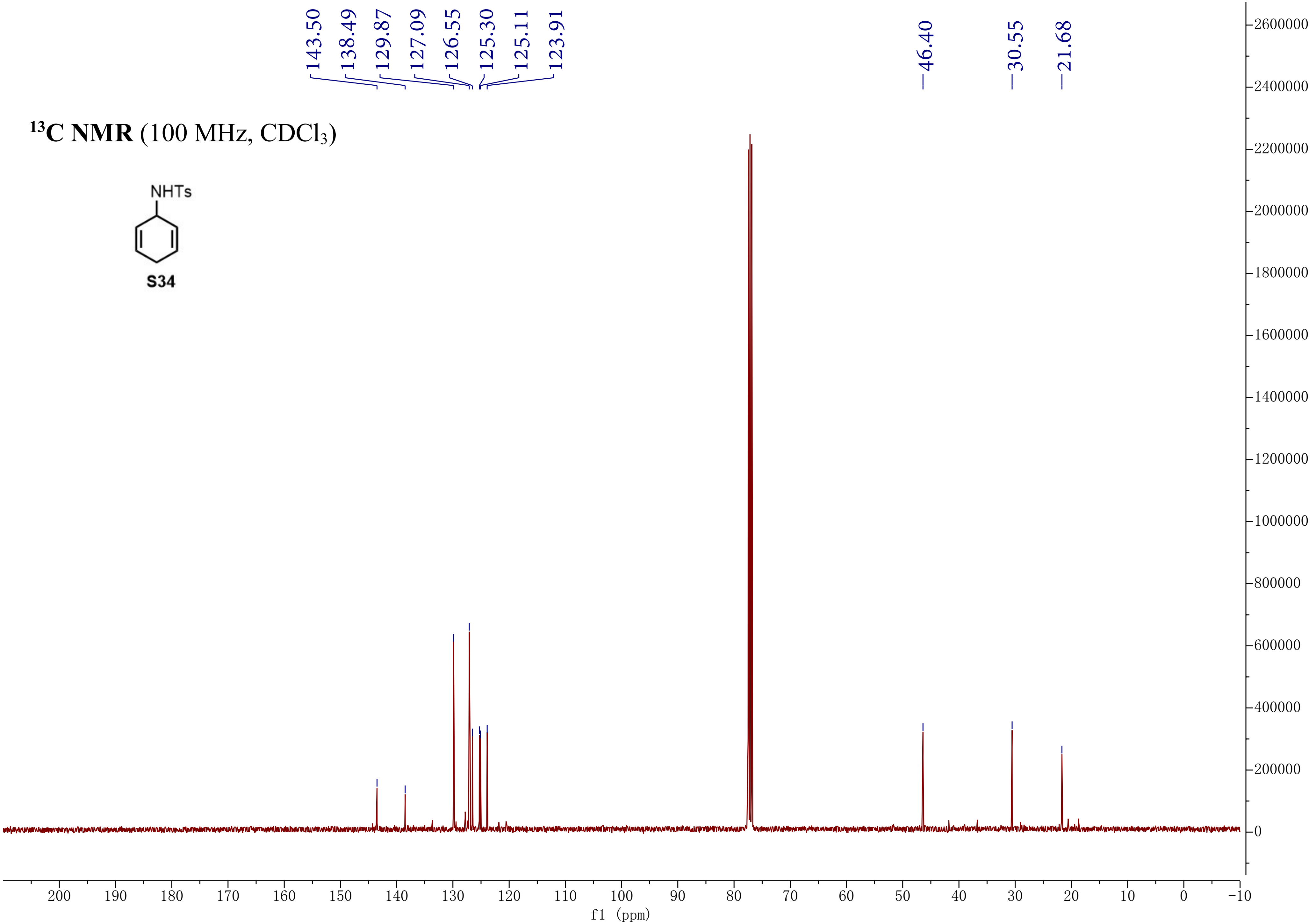

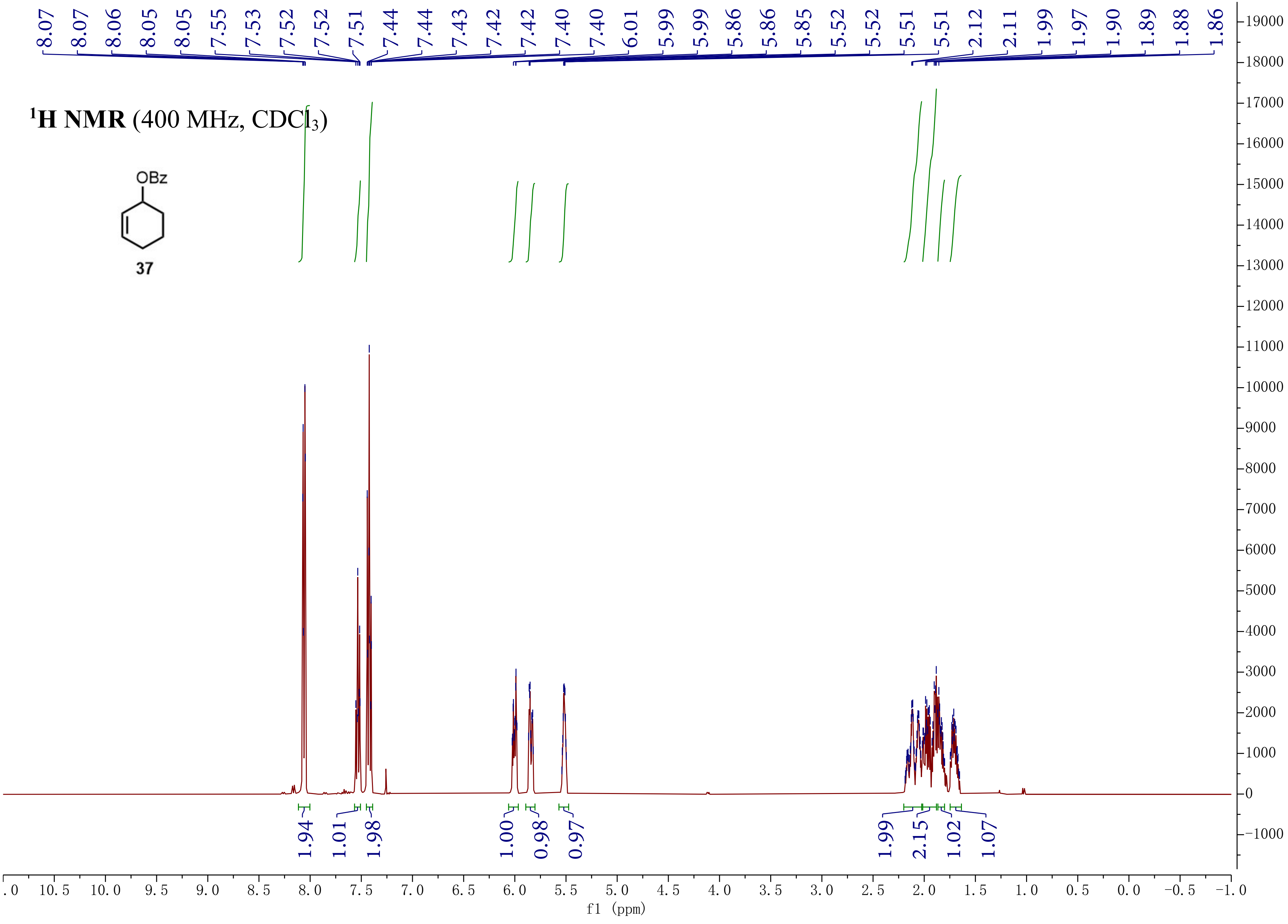

1H NMR (400 MHz, CDCl3)
OBz
37
8.07 8.07 8.06 8.05 8.05 7.55 7.53 7.52 7.52 7.51 7.44 7.44 7.43 7.42 7.42 7.40 7.40 6.01 5.99 5.99 5.86 5.86 5.85 5.52 5.52 5.51 5.51 2.12 2.11 1.99 1.97 1.90 1.89 1.88 1.86
1.94 1.01 1.98 1.00 0.98 0.97 1.99 2.15 1.02 1.07
f1 (ppm)

^{13}C NMR (100 MHz, $CDCl_3$)

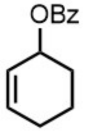

37

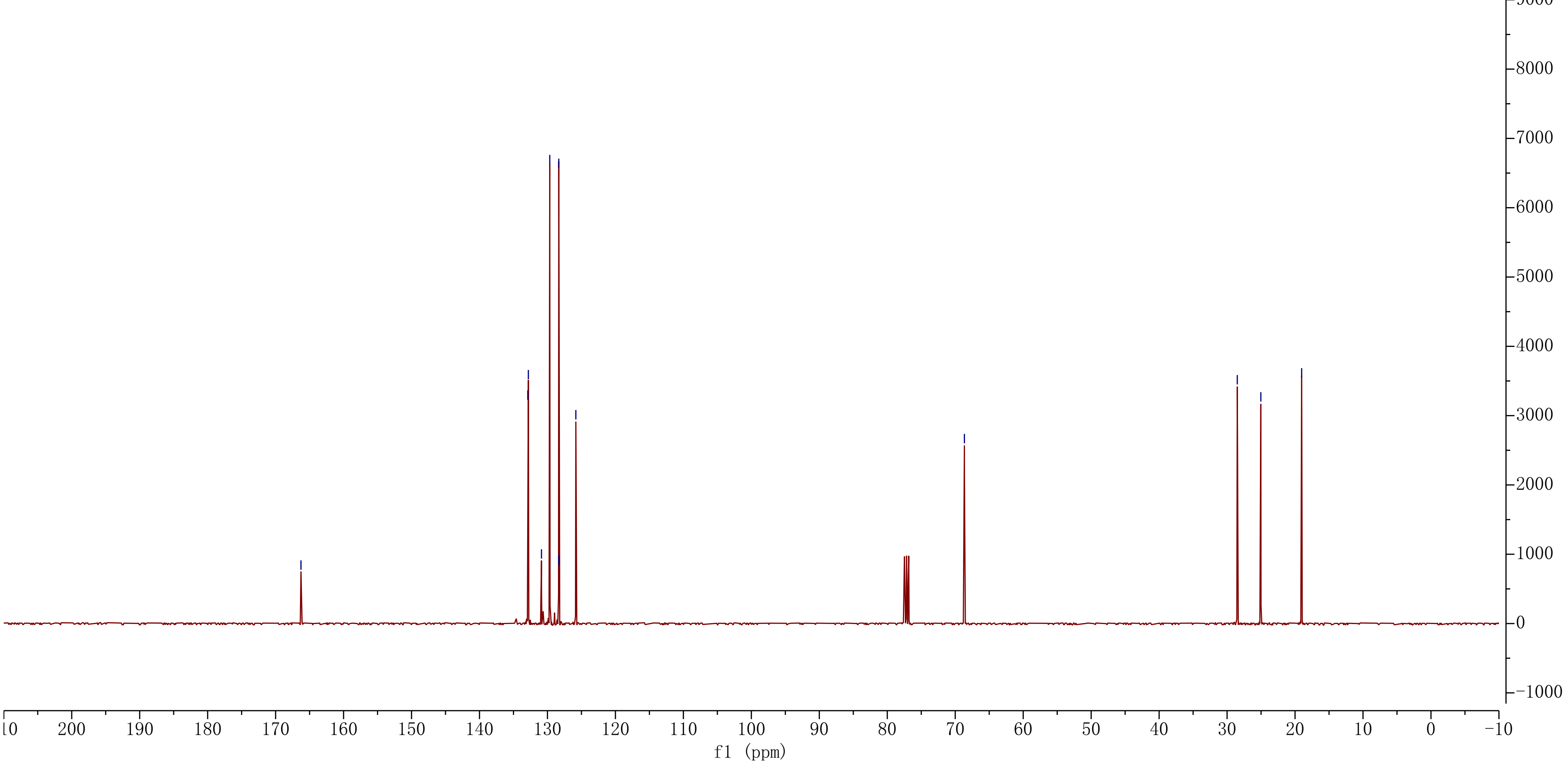

^{1}H NMR (400 MHz, $CDCl_3$)

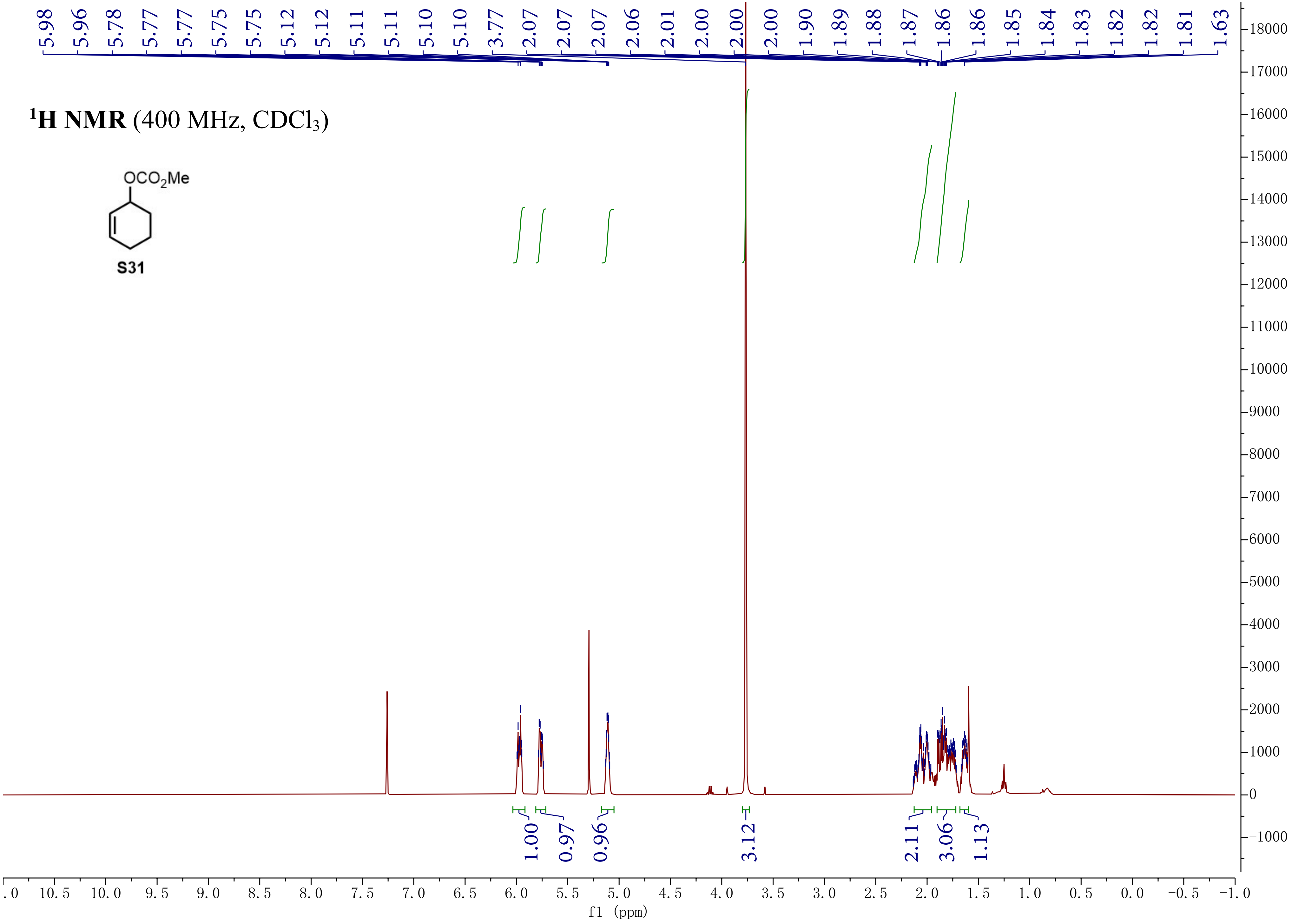

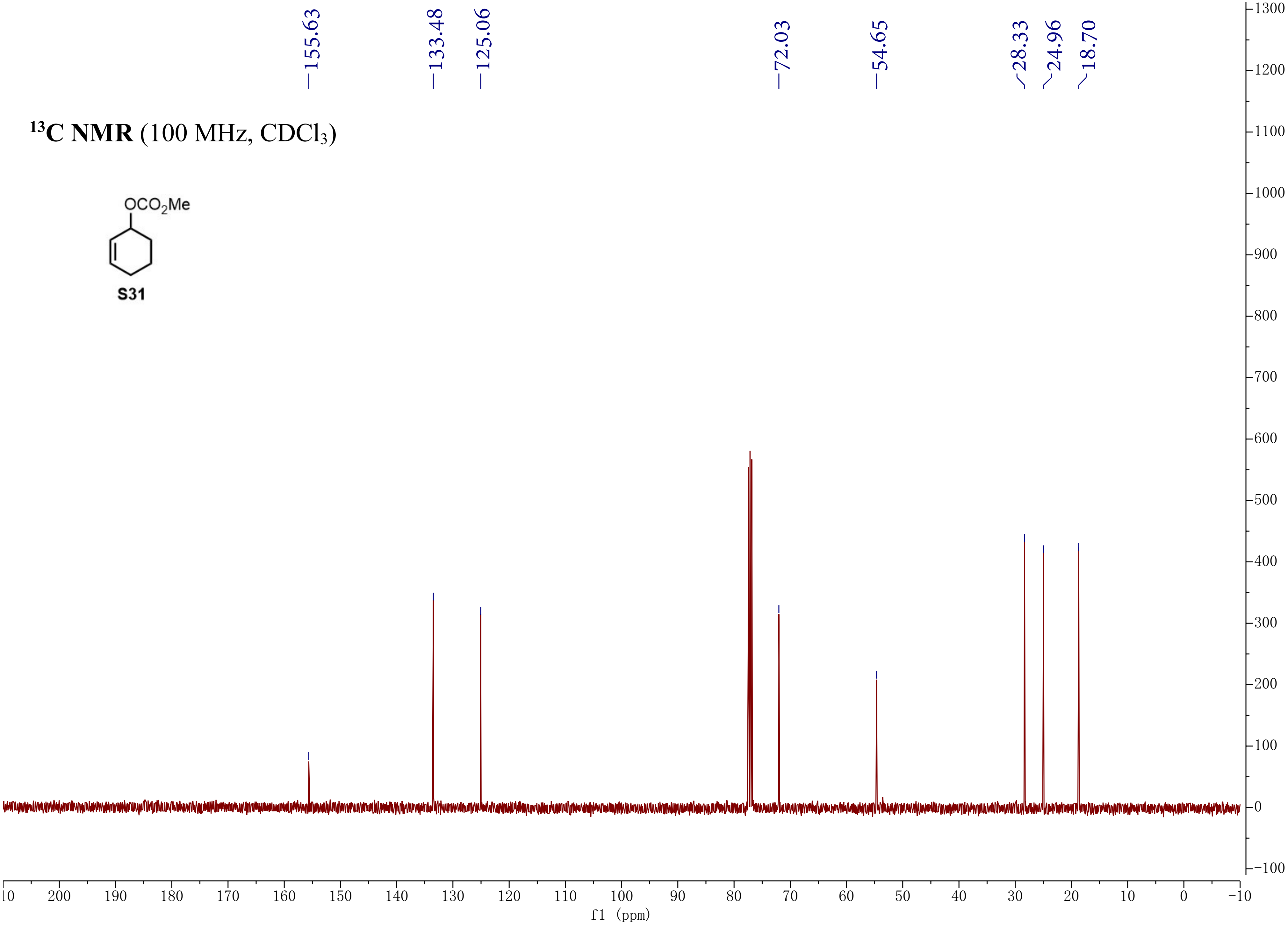

155.63
133.48
125.06
72.03
54.65
28.33
24.96
18.70
13C NMR (100 MHz, CDCl3)
OCO2Me
S31
f1 (ppm)

^{1}H NMR (400 MHz, $CDCl_3$)

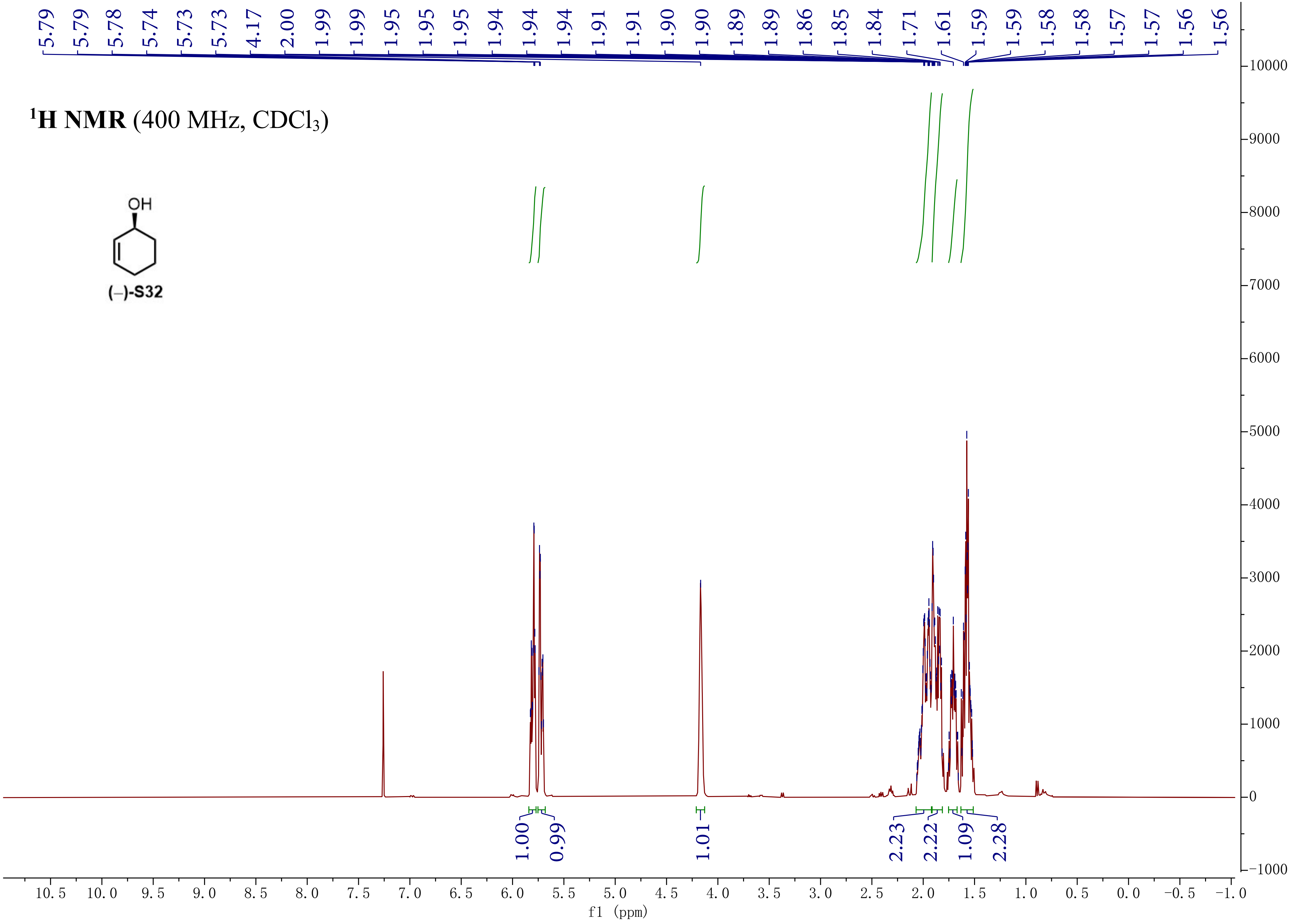

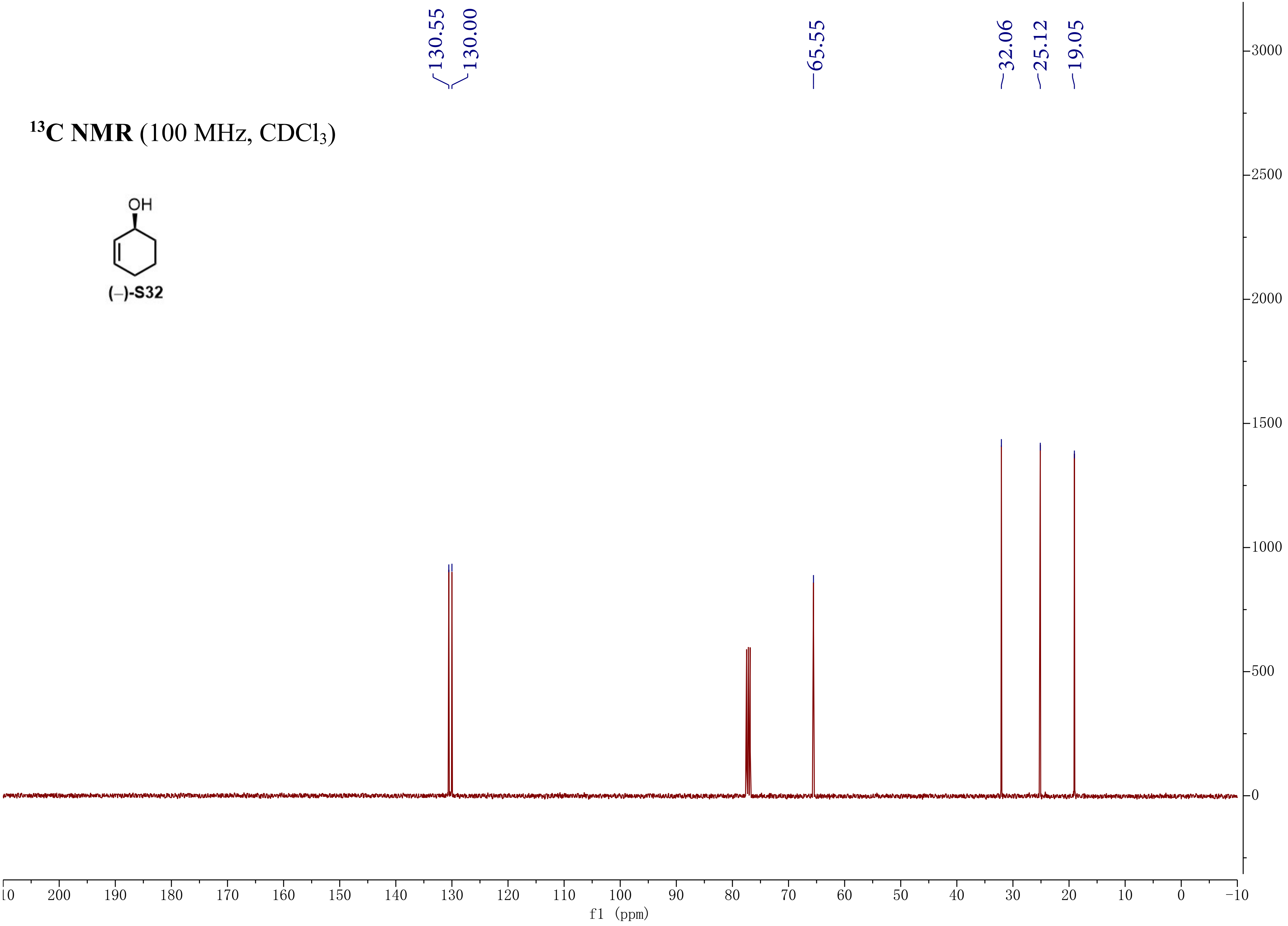

^{13}C NMR (100 MHz, $CDCl_3$)
OH
(−)-S32
130.55
130.00
65.55
32.06
25.12
19.05
3000
2500
2000
1500
1000
500
0
10
200
190
180
170
160
150
140
130
120
110
100
90
80
70
60
50
40
30
20
10
0
−10
f1 (ppm)

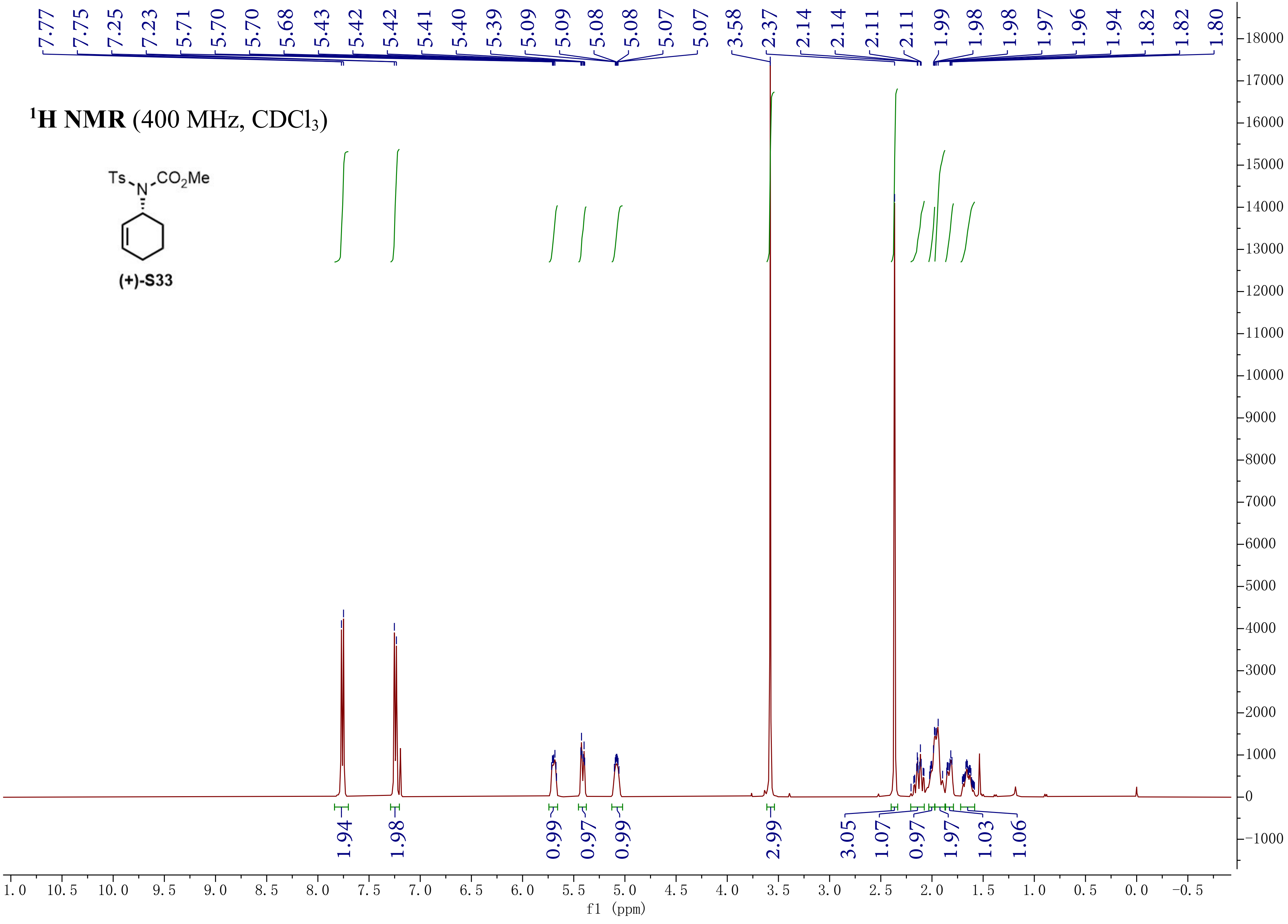

1H NMR (400 MHz, CDCl3)
Ts
N
CO2Me
(+)-S33
7.77 7.75 7.25 7.23 5.71 5.70 5.70 5.68 5.43 5.42 5.42 5.41 5.40 5.39 5.09 5.09 5.08 5.08 5.07 5.07 3.58 2.37 2.14 2.14 2.11 2.11 1.99 1.98 1.98 1.97 1.96 1.94 1.82 1.82 1.80
1.94 1.98 0.99 0.97 0.99 2.99 3.05 1.07 0.97 1.97 1.03 1.06
f1 (ppm)

^{13}C NMR (100 MHz, $CDCl_3$)

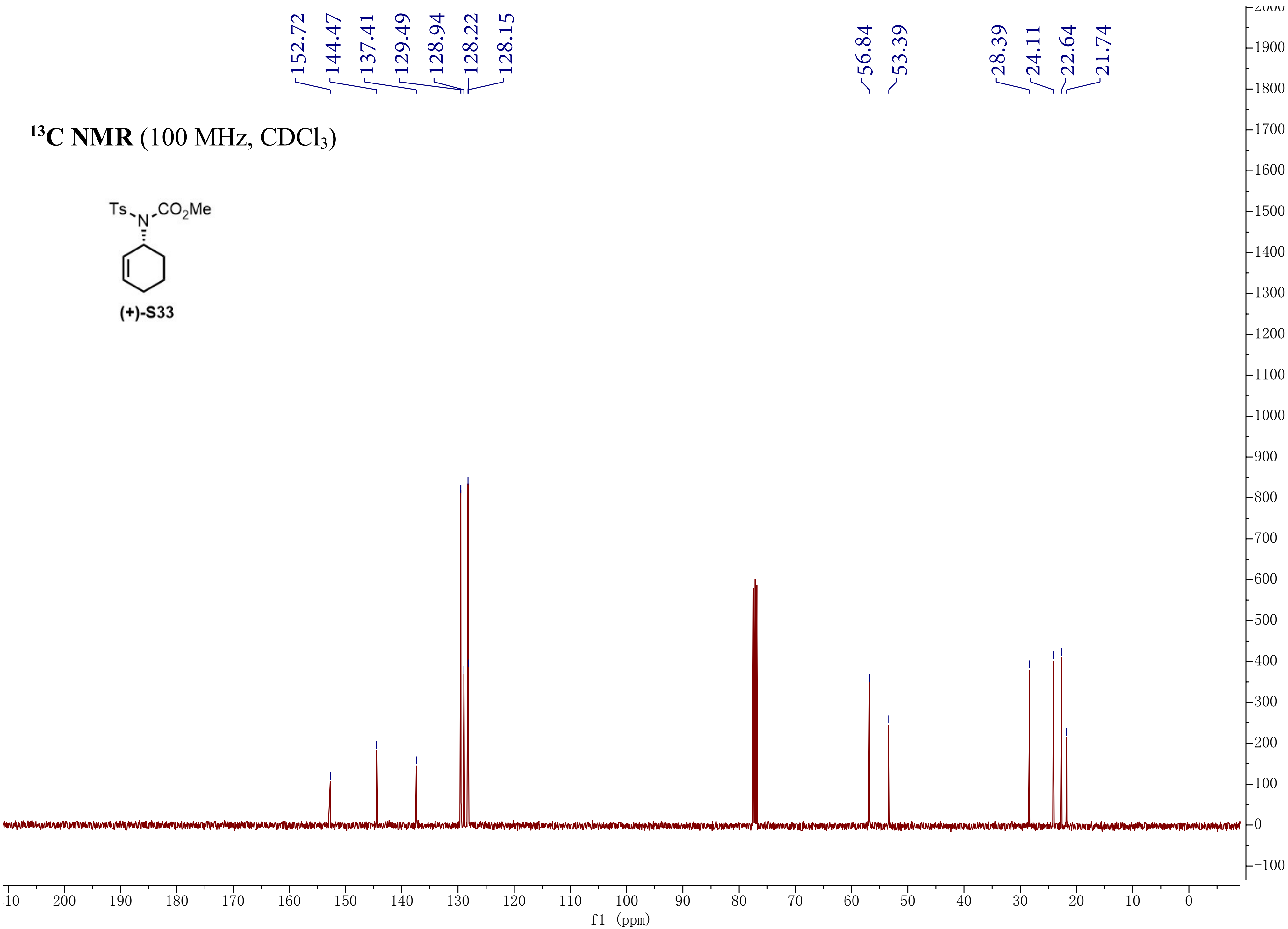

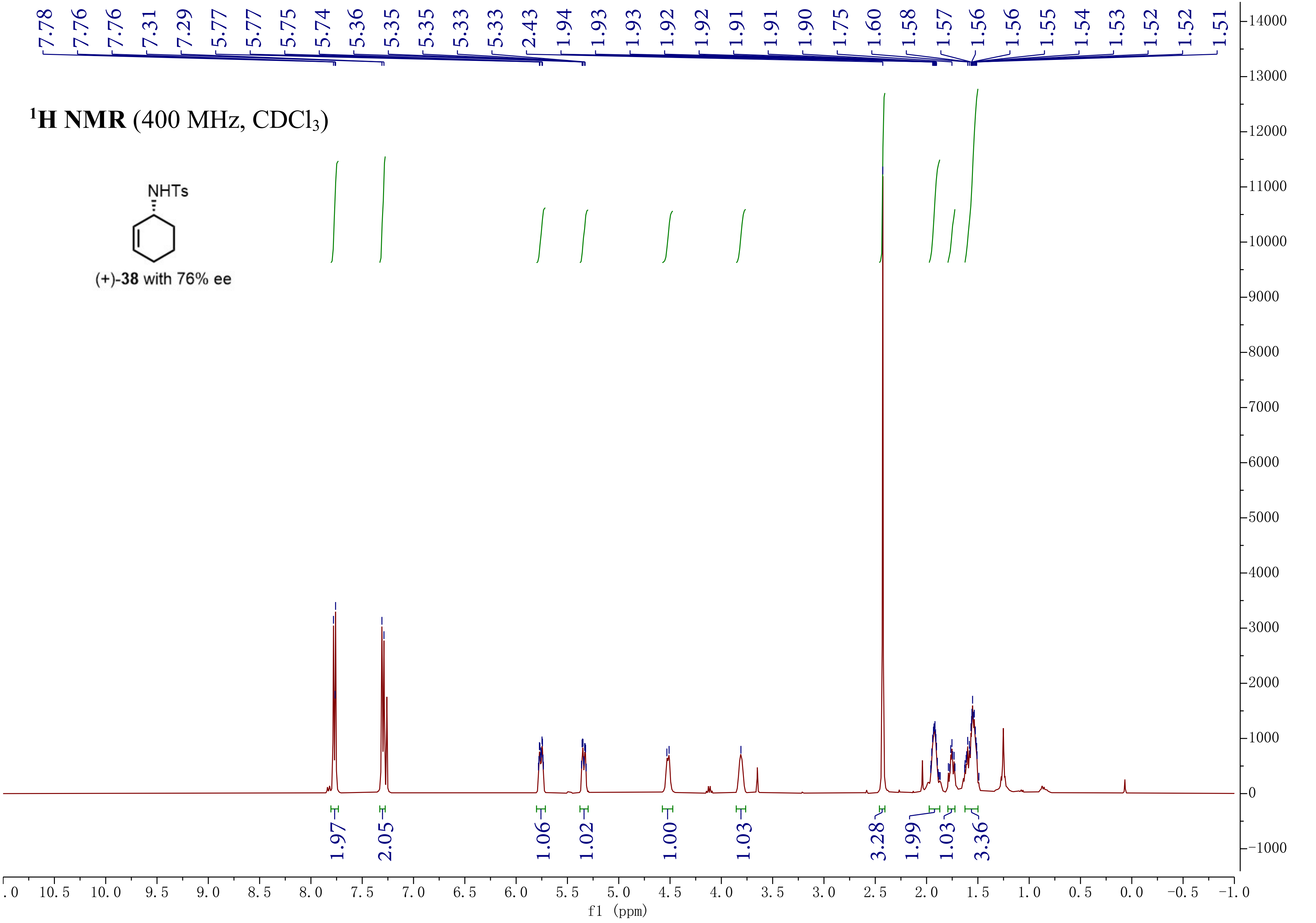

^{1}H NMR (400 MHz, CDCl$_3$)
NHTs
(+)-38 with 76% ee
7.78 7.76 7.76 7.31 7.29 5.77 5.77 5.75 5.74 5.36 5.35 5.35 5.33 5.33 2.43 1.94 1.93 1.93 1.92 1.92 1.91 1.91 1.90 1.75 1.60 1.58 1.57 1.56 1.56 1.55 1.54 1.53 1.52 1.52 1.51
1.97 2.05 1.06 1.02 1.00 1.03 3.28 1.99 1.03 3.36
f1 (ppm)

^{13}C NMR (100 MHz, $CDCl_3$)

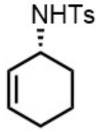
(+)-**38** with 76% ee

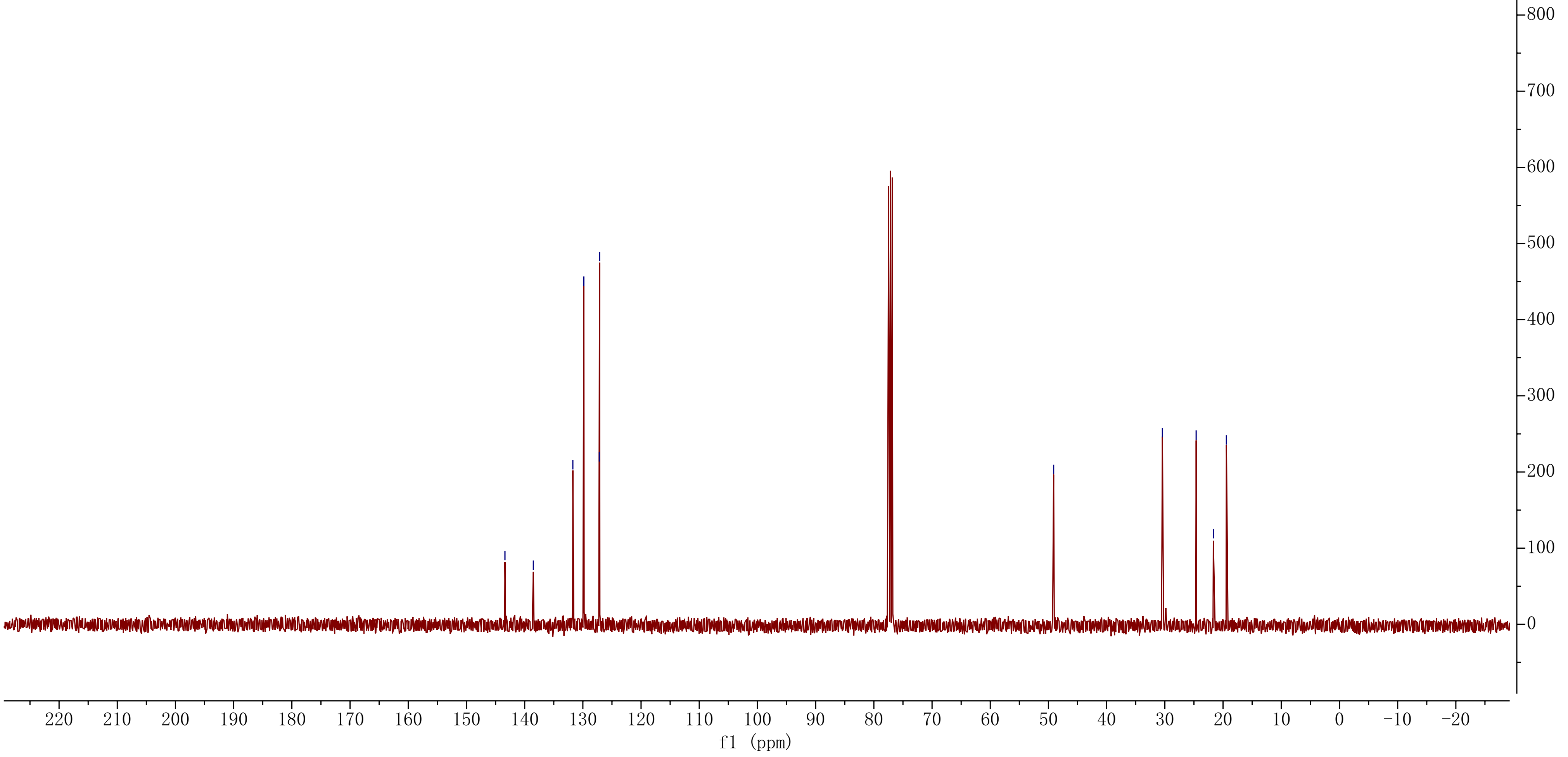

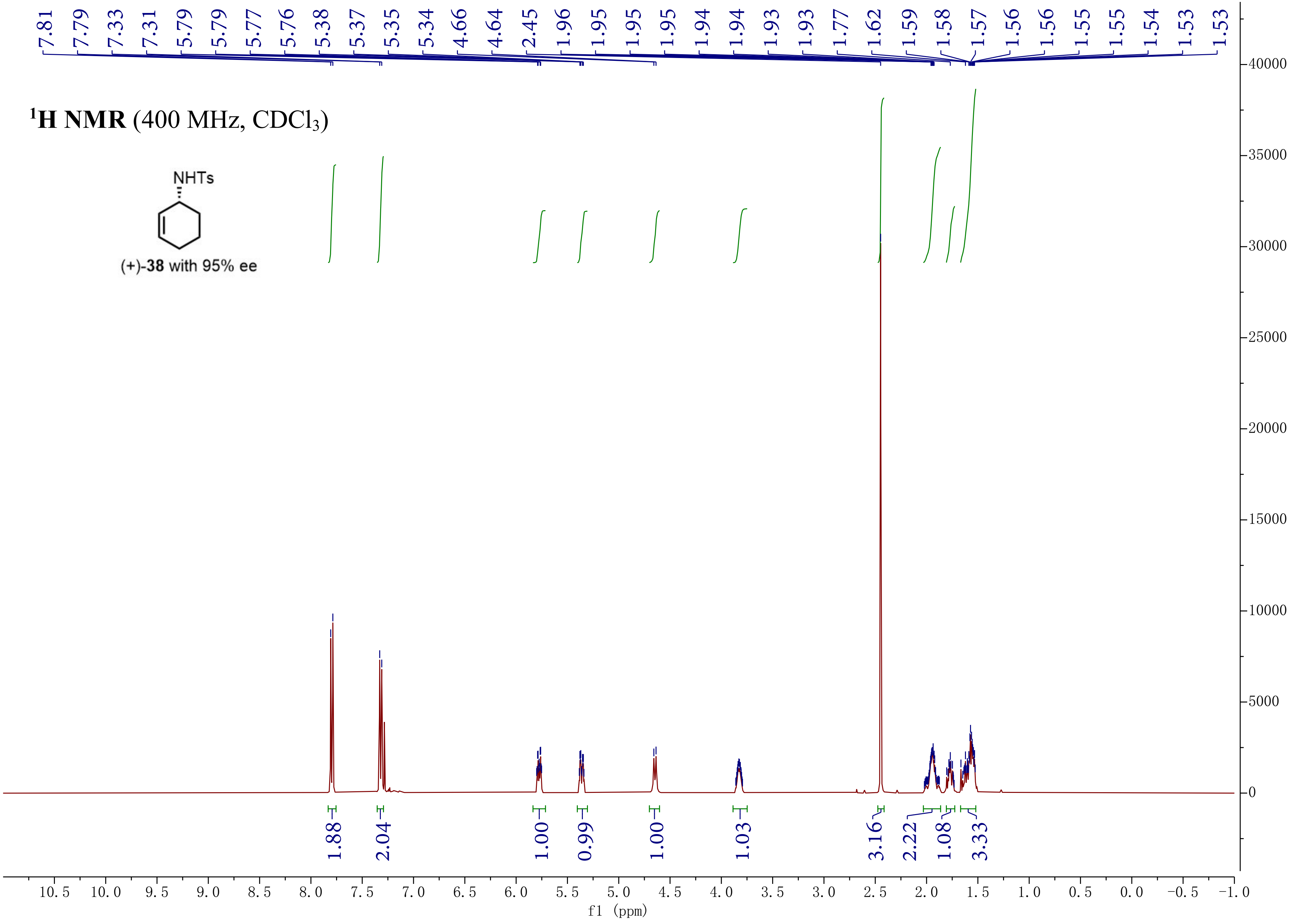

7.81 7.79 7.33 7.31 5.79 5.79 5.77 5.76 5.38 5.37 5.35 5.34 4.66 4.64 2.45 1.96 1.95 1.95 1.95 1.94 1.94 1.93 1.93 1.77 1.62 1.59 1.58 1.57 1.56 1.56 1.55 1.55 1.54 1.53 1.53
^{1}H NMR (400 MHz, $CDCl_3$)
NHTs
(+)-38 with 95% ee
1.88 2.04 1.00 0.99 1.00 1.03 3.16 2.22 1.08 3.33
f1 (ppm)

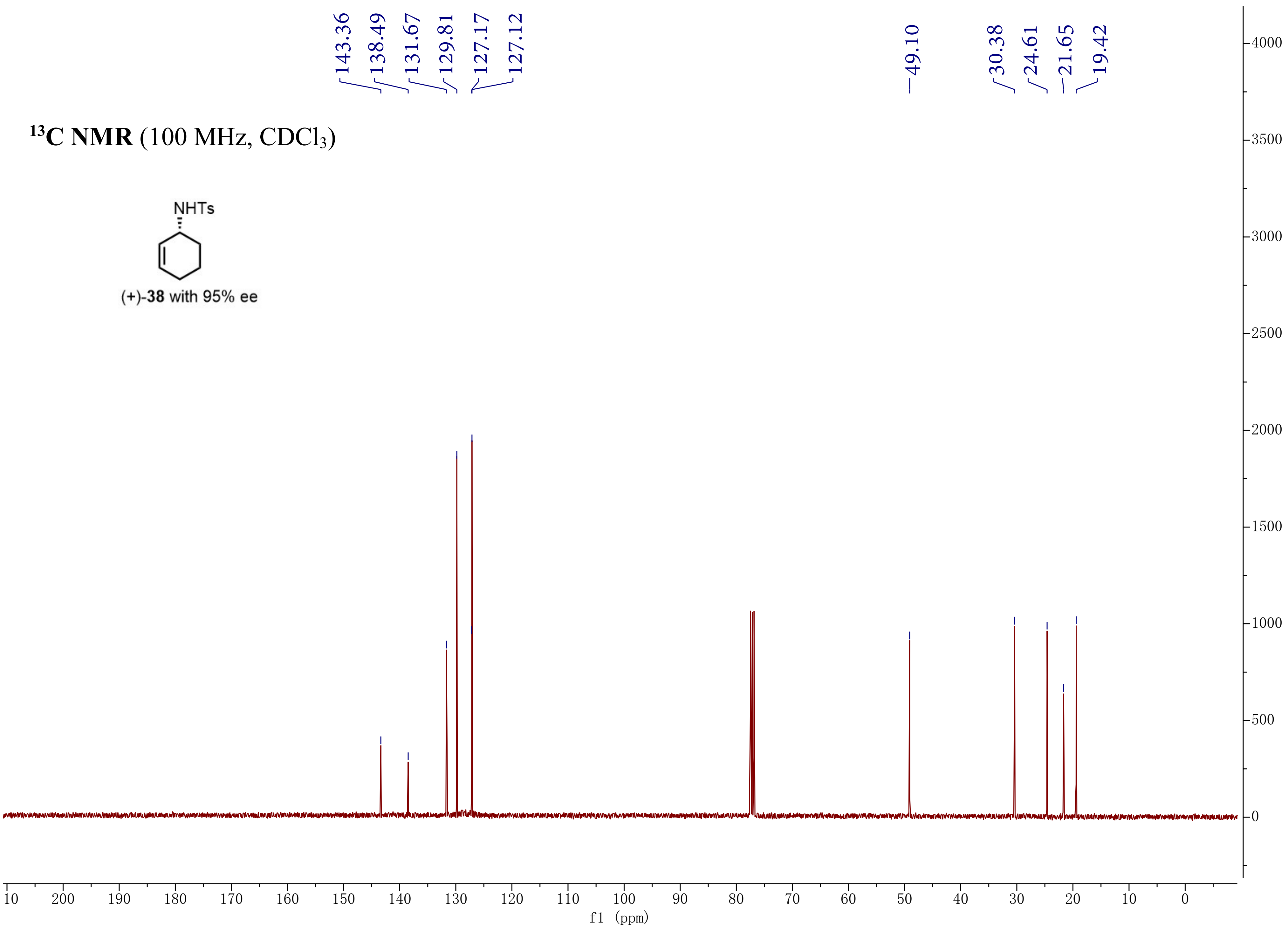

13C NMR (100 MHz, CDCl3)
NHTs
(+)-38 with 95% ee
143.36
138.49
131.67
129.81
127.17
127.12
49.10
30.38
24.61
21.65
19.42
f1 (ppm)

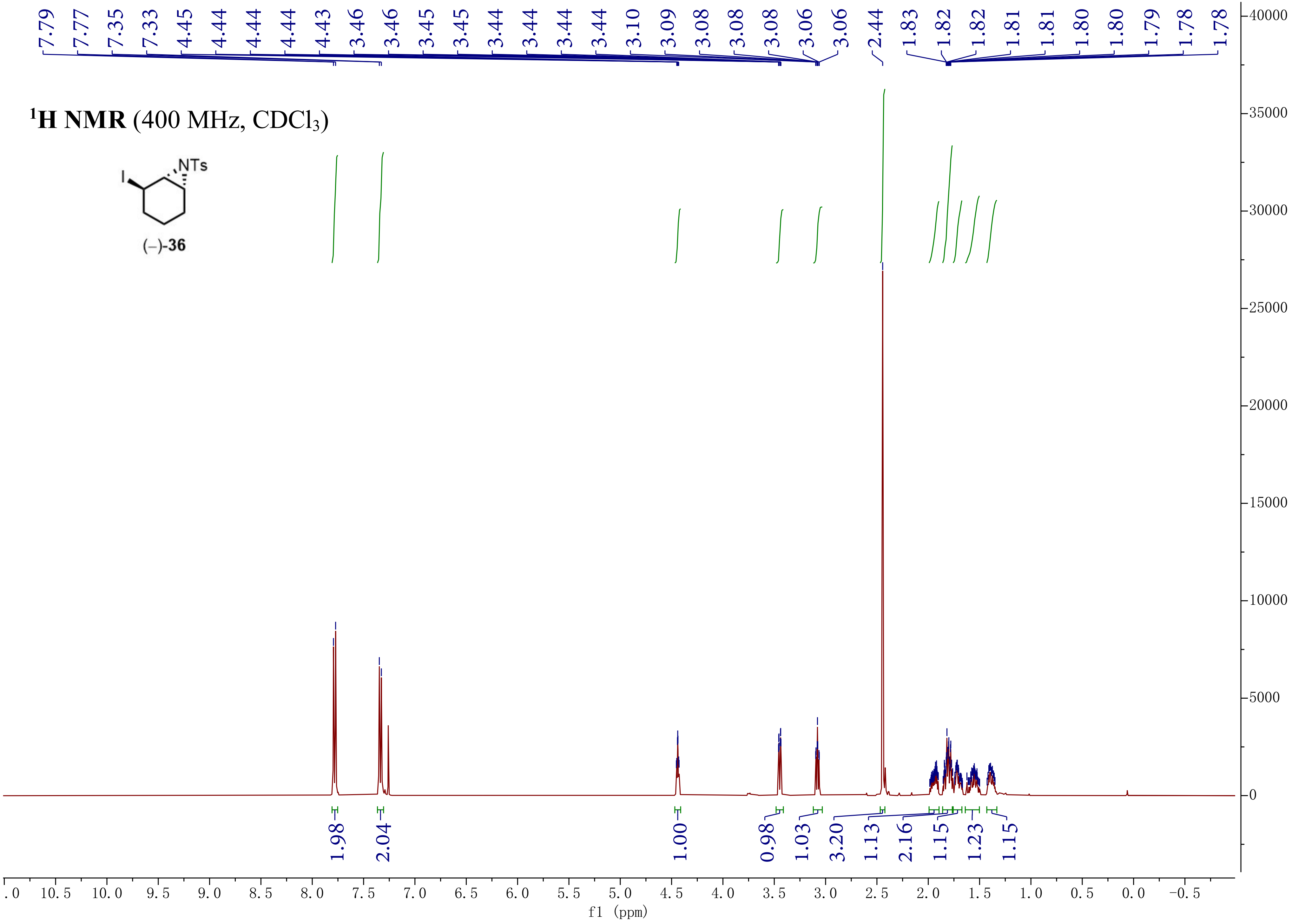

^{1}H NMR (400 MHz, $CDCl_3$)
NTs
I
(−)-36
7.79 7.77 7.35 7.33 4.45 4.44 4.44 4.44 4.43 3.46 3.46 3.45 3.45 3.44 3.44 3.44 3.44 3.10 3.09 3.08 3.08 3.08 3.06 3.06 2.44 1.83 1.82 1.82 1.81 1.81 1.80 1.80 1.79 1.78 1.78
1.98 2.04 1.00 0.98 1.03 3.20 1.13 2.16 1.15 1.23 1.15
40000 35000 30000 25000 20000 15000 10000 5000 0
.0 10.5 10.0 9.5 9.0 8.5 8.0 7.5 7.0 6.5 6.0 5.5 5.0 4.5 4.0 3.5 3.0 2.5 2.0 1.5 1.0 0.5 0.0 −0.5
f1 (ppm)

^{13}C **NMR** (100 MHz, $CDCl_3$)

(–)-**36**

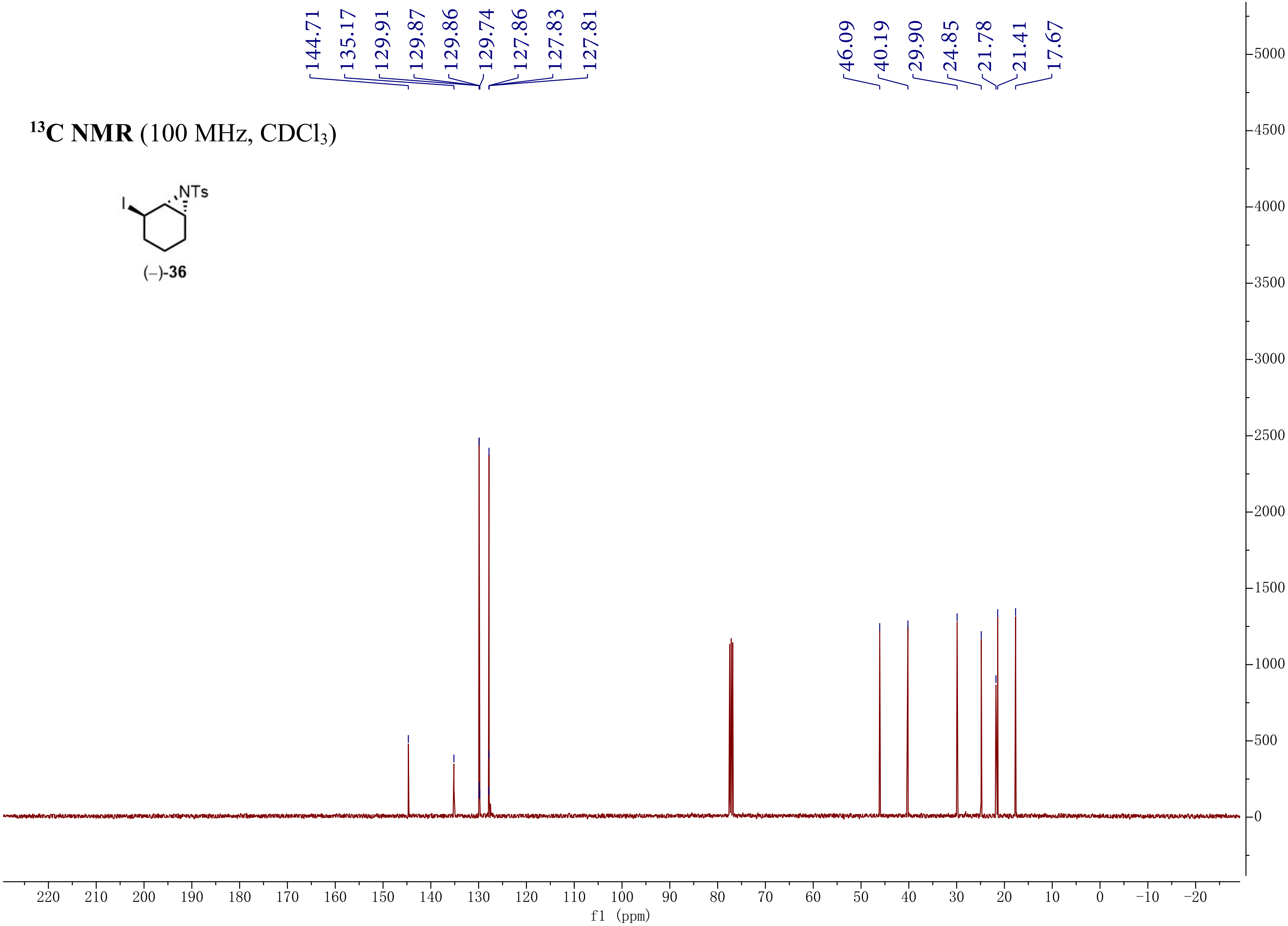

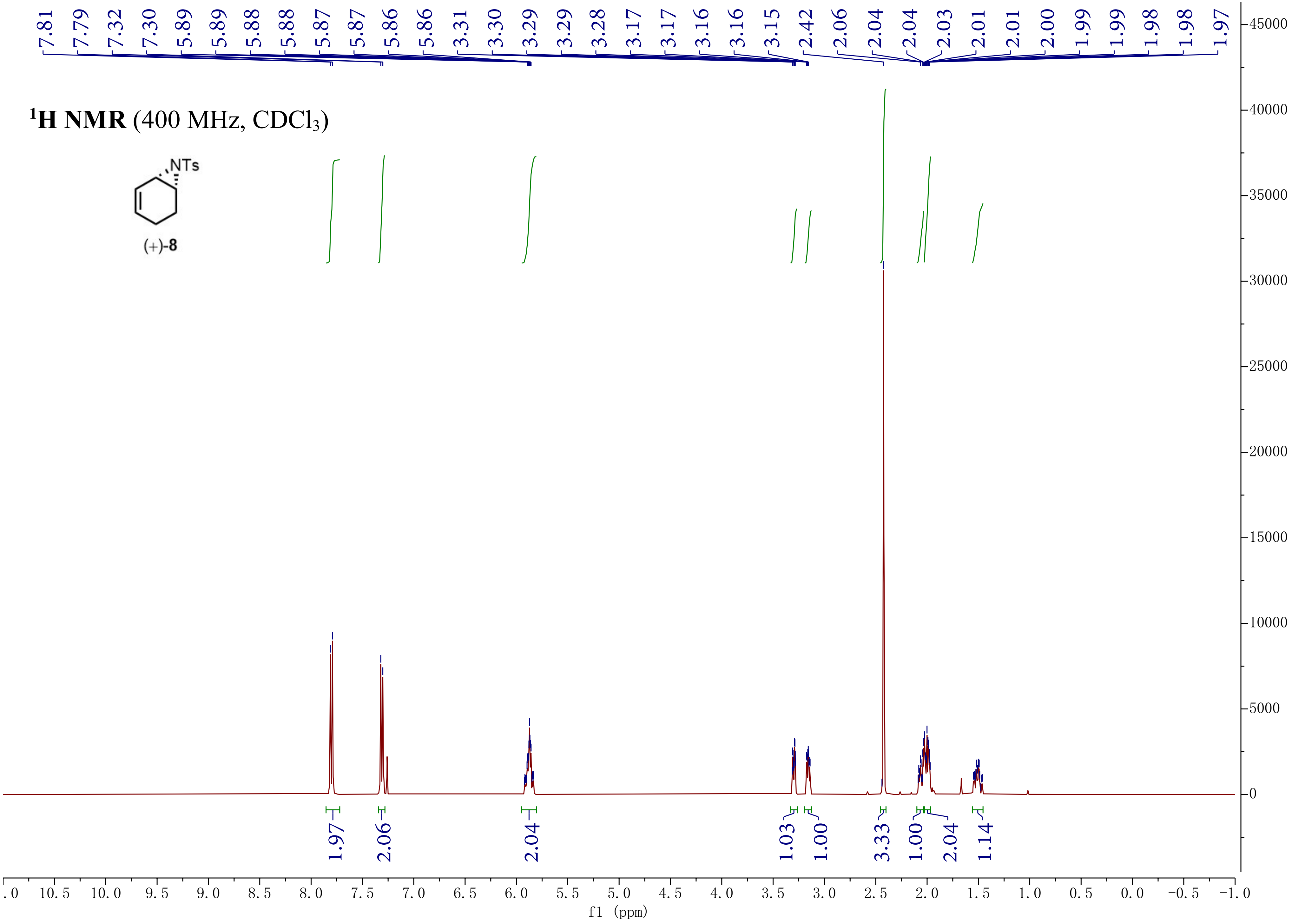

7.81 7.79 7.32 7.30 5.89 5.89 5.88 5.88 5.87 5.87 5.86 5.86 3.31 3.30 3.29 3.29 3.28 3.17 3.17 3.16 3.16 3.15 2.42 2.06 2.04 2.04 2.03 2.01 2.01 2.00 1.99 1.99 1.98 1.98 1.97
¹H NMR (400 MHz, CDCl₃)
NTs
(+)-8
1.97 2.06 2.04 1.03 1.00 3.33 1.00 2.04 1.14
f1 (ppm)

^{13}C **NMR** (100 MHz, $CDCl_3$)

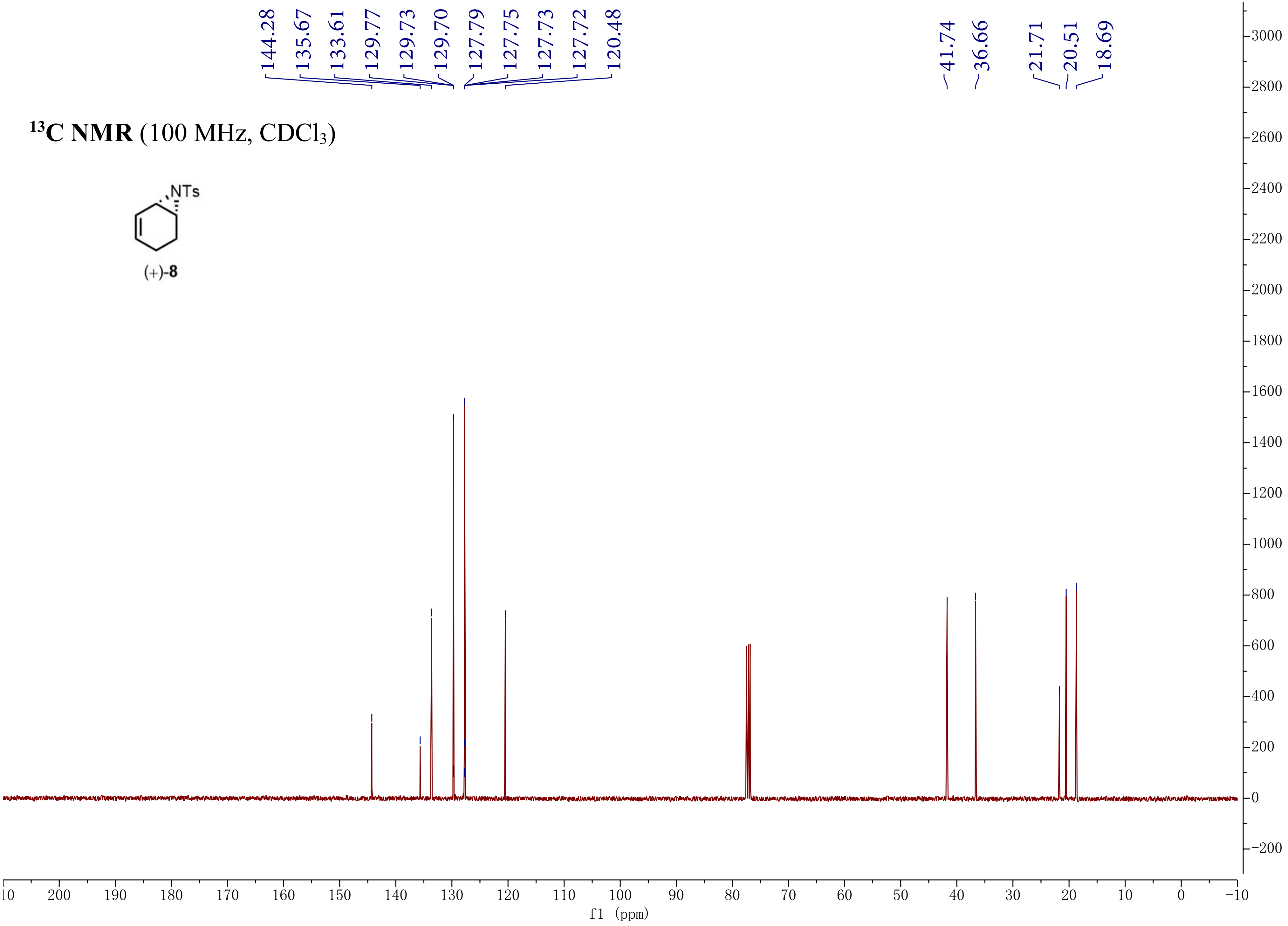

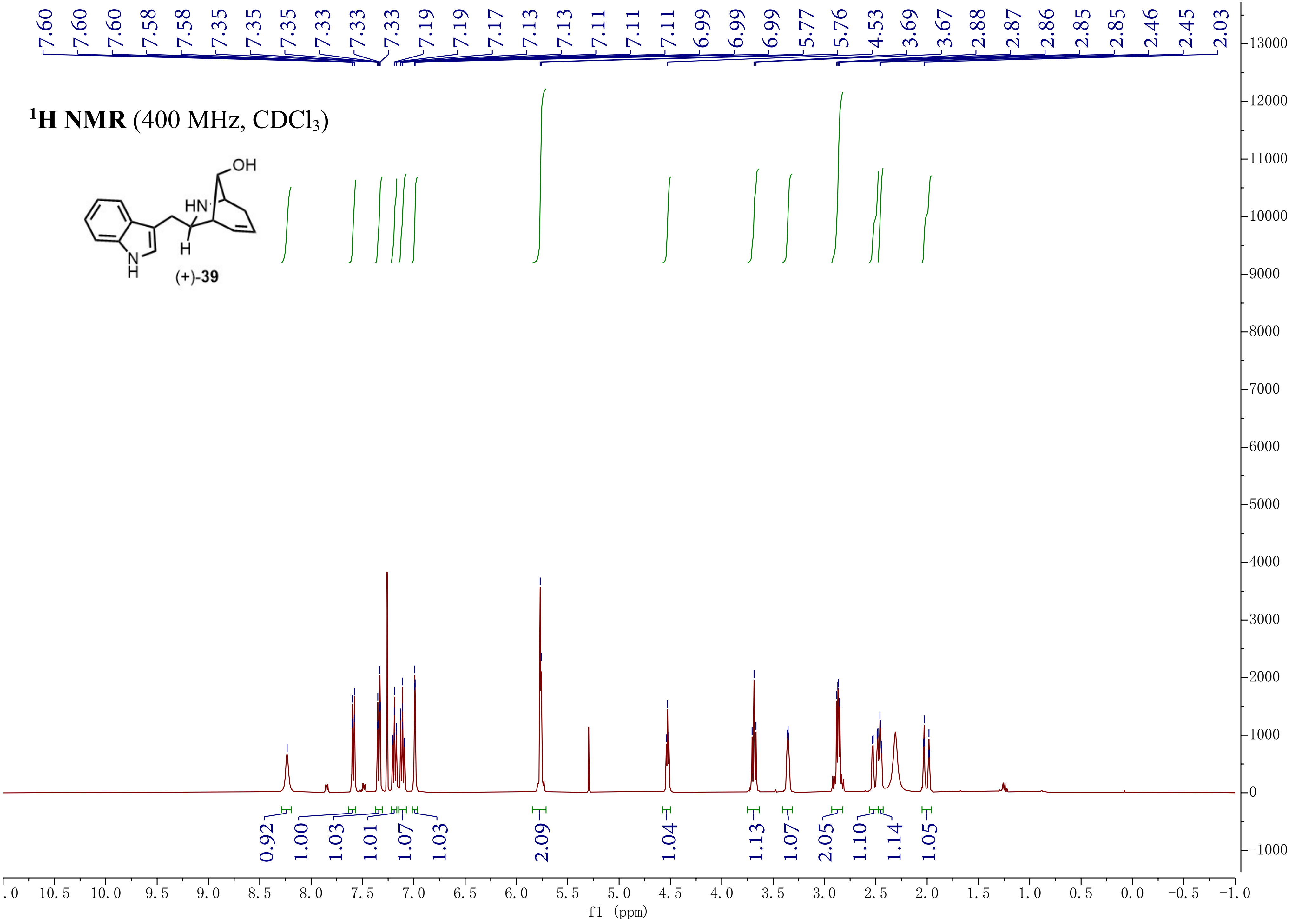

1H NMR (400 MHz, CDCl3)
(+)-39
OH
HN
H
N
H
7.60 7.60 7.60 7.58 7.58 7.35 7.35 7.35 7.33 7.33 7.33 7.19 7.19 7.17 7.13 7.13 7.11 7.11 7.11 6.99 6.99 6.99 5.77 5.76 4.53 3.69 3.67 2.88 2.87 2.86 2.85 2.85 2.46 2.45 2.03
0.92 1.00 1.03 1.01 1.07 1.03 2.09 1.04 1.13 1.07 2.05 1.10 1.14 1.05
f1 (ppm)

^{13}C NMR (100 MHz, $CDCl_3$)

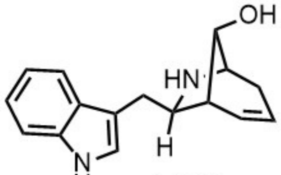

(+)-**39**

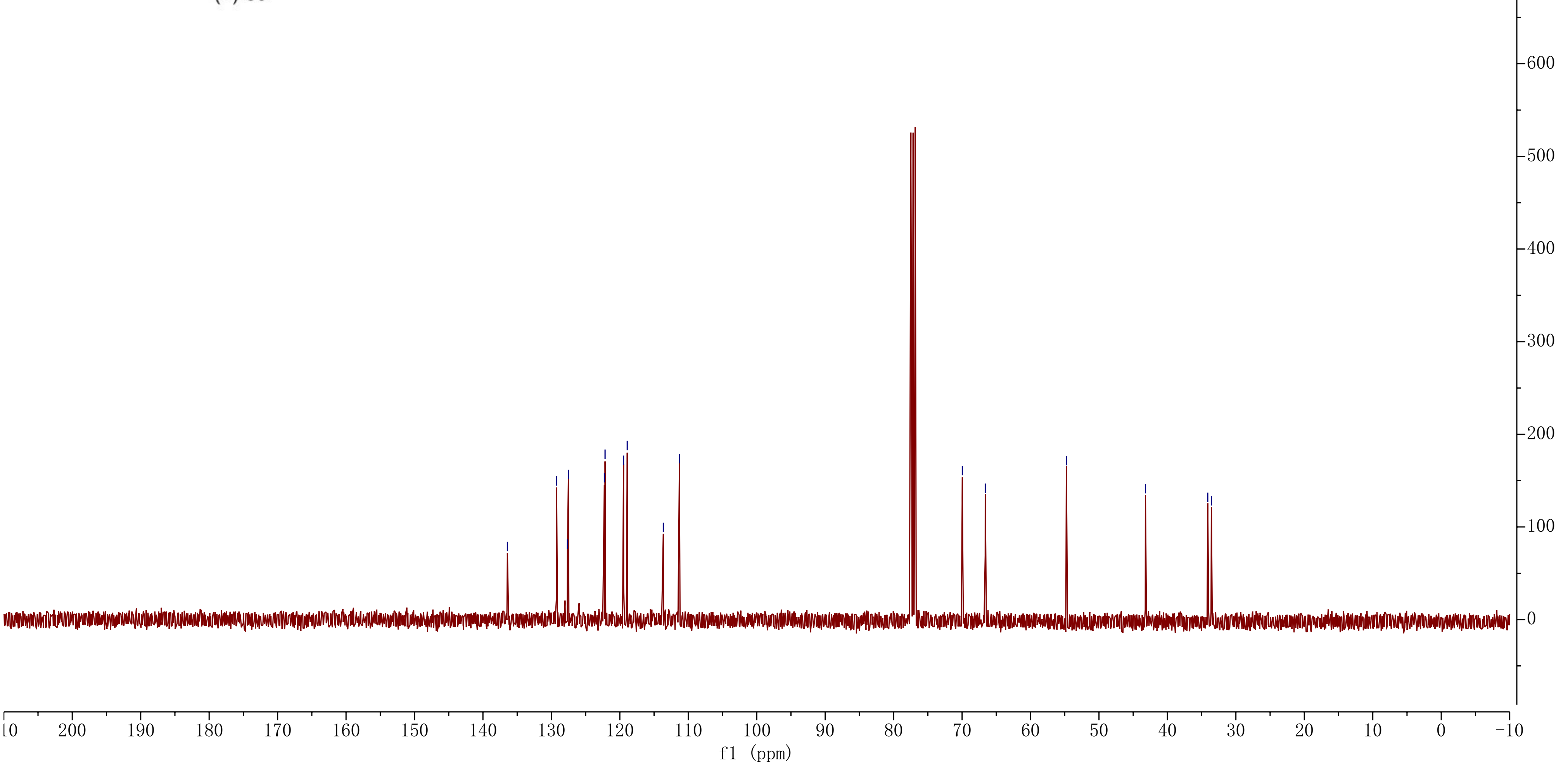

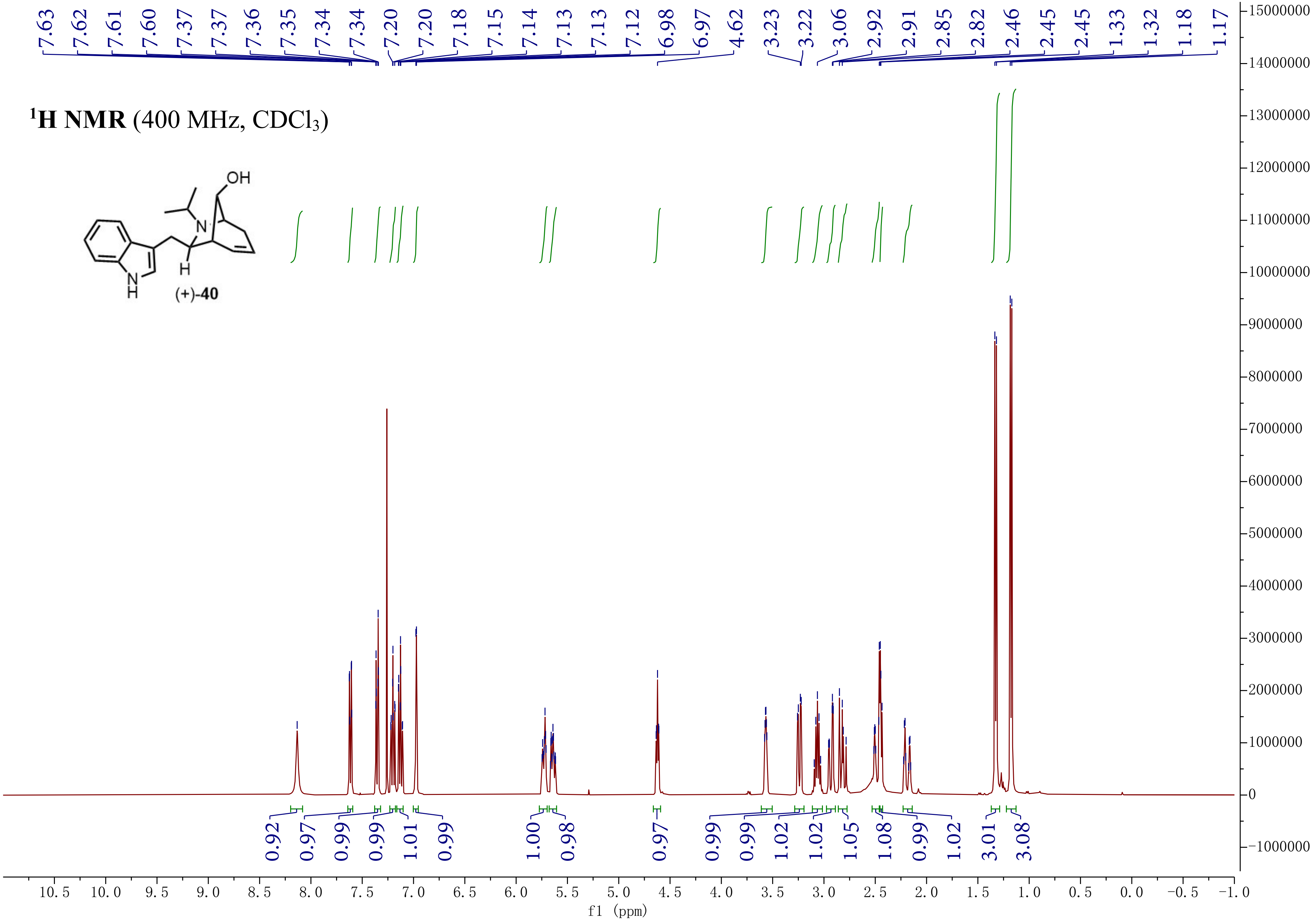

7.63
7.62
7.61
7.60
7.37
7.37
7.36
7.35
7.34
7.34
7.20
7.20
7.18
7.15
7.14
7.13
7.13
7.12
6.98
6.97
4.62
3.23
3.22
3.06
2.92
2.91
2.85
2.82
2.46
2.45
2.45
1.33
1.32
1.18
1.17
1H NMR (400 MHz, CDCl3)
OH
N
H
N
H
(+)-40
0.92
0.97
0.99
0.99
1.01
0.99
1.00
0.98
0.97
0.99
0.99
1.02
1.02
1.05
1.08
0.99
1.02
3.01
3.08
f1 (ppm)

^{13}C NMR (100 MHz, $CDCl_3$)

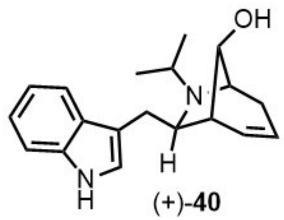

(+)-**40**

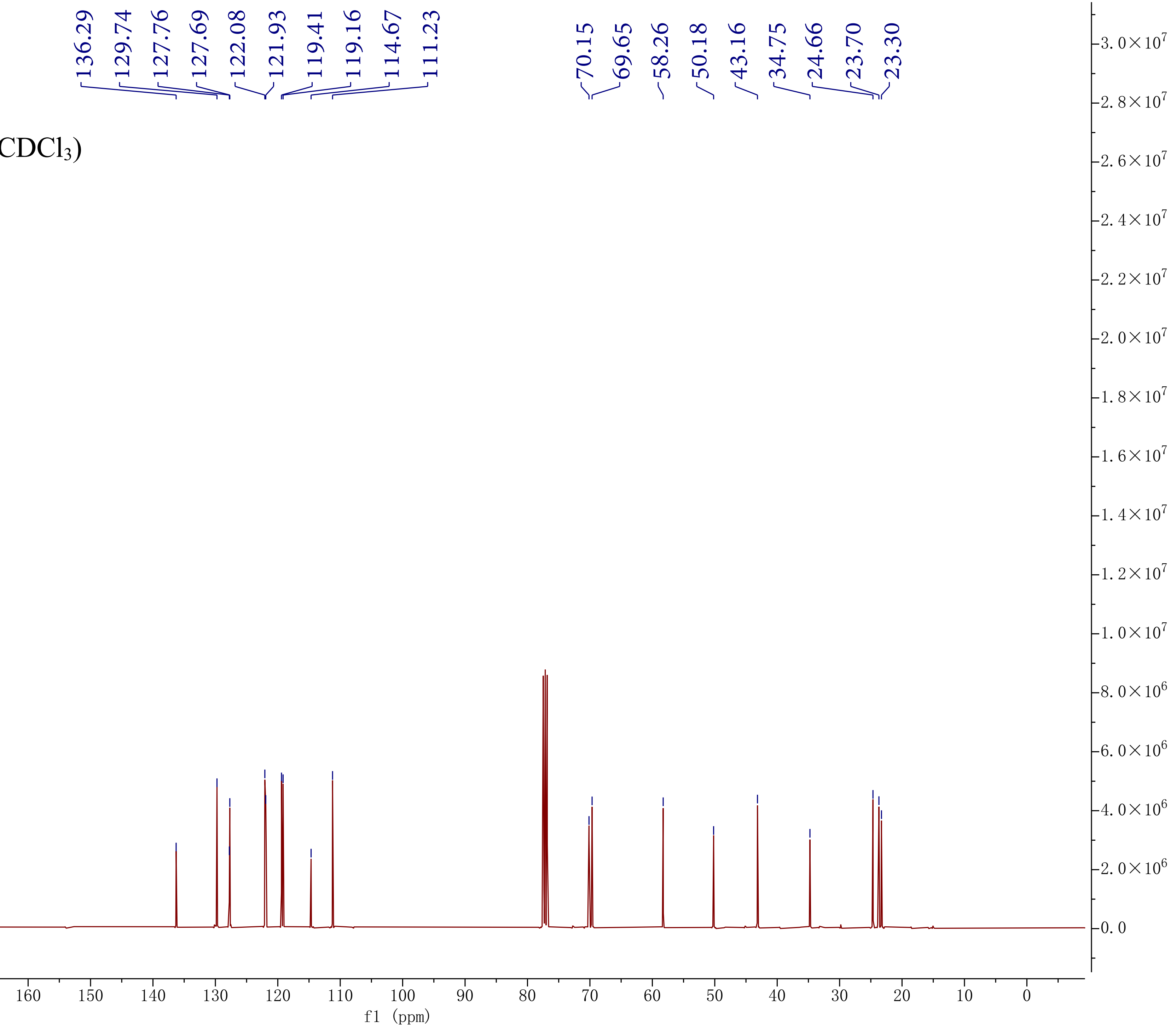

^{1}H NMR (400 MHz, $CDCl_3$)

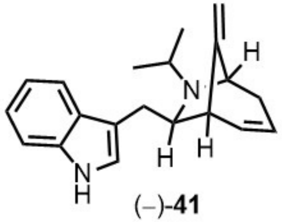

(−)-**41**

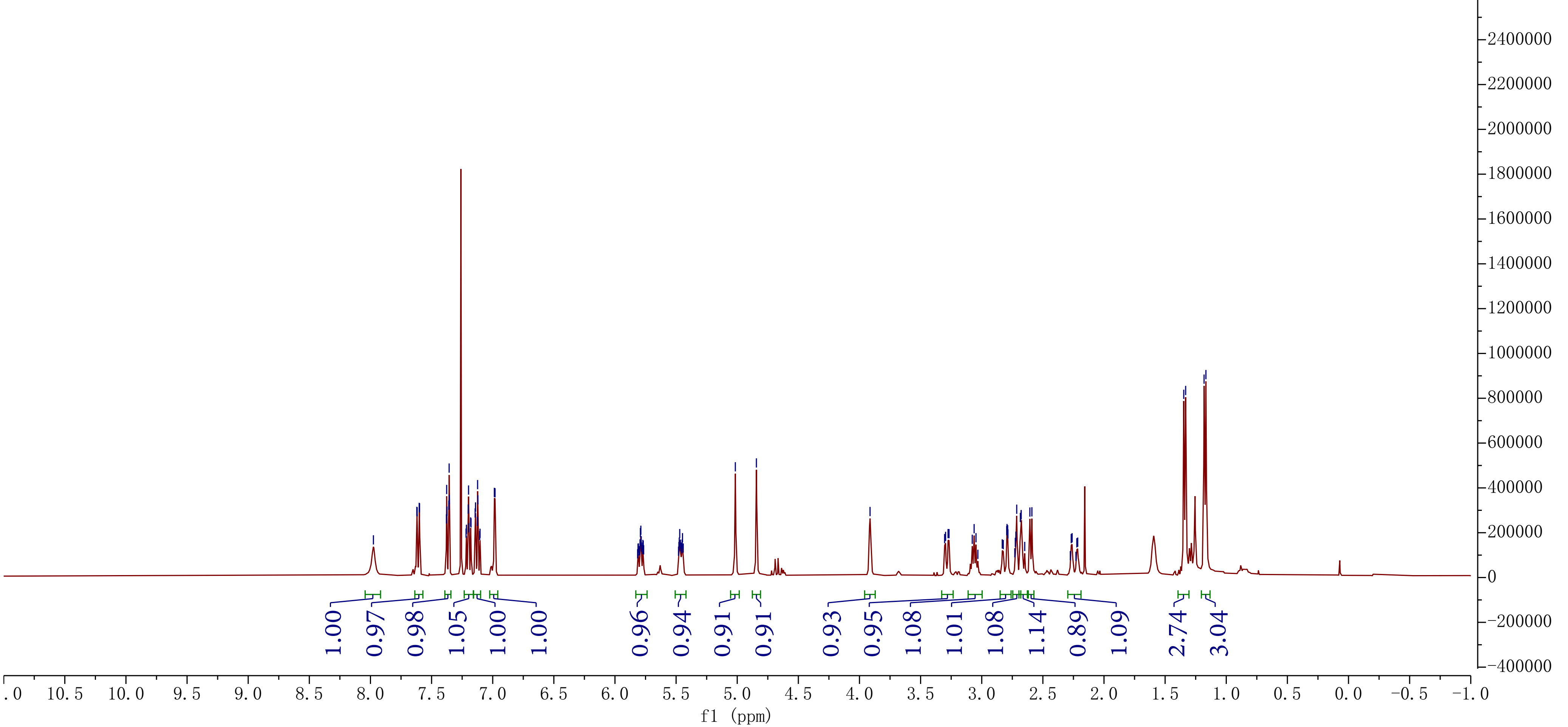

^{13}C NMR (100 MHz, CDCl$_3$)

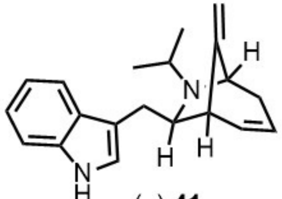

(–)-**41**

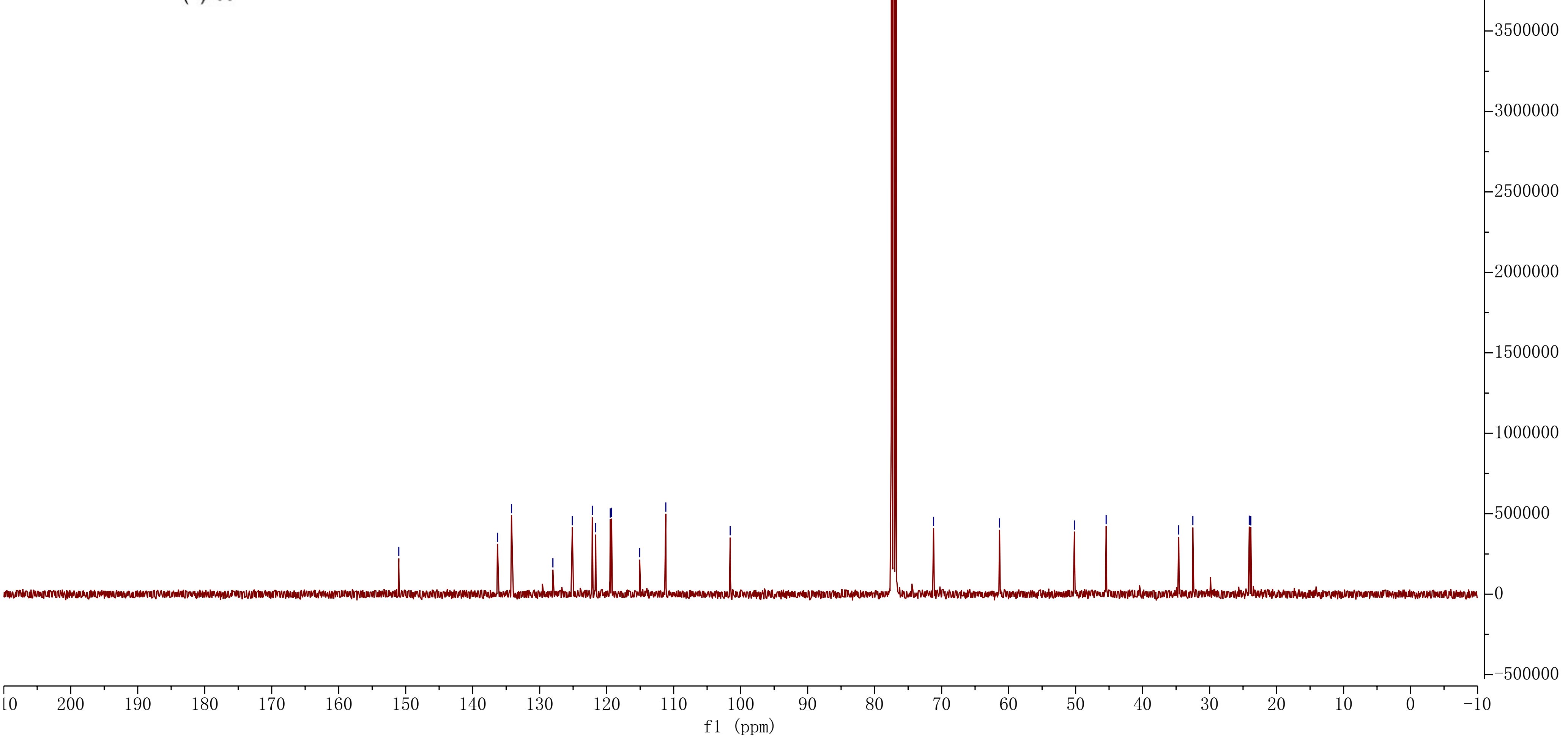

Supplement: Supplementary file 1 [file ol5c02062_si_001.pdf]
